# Bismuth Meets Olefins: Ethylene Activation and Reversible Alkene Insertion into Bi─N Bonds

**DOI:** 10.1002/anie.202505434

**Published:** 2025-05-08

**Authors:** Sangeetha Satheesh, Kai Oberdorf, Luis Roeck, Ahmed Fetoh, F. Matthias Bickelhaupt, Jordi Poater, Crispin Lichtenberg

**Affiliations:** ^1^ Department of Chemistry Philipps‐University Marburg Hans‐Meerwein‐Str. 4, 35032 Marburg Germany; ^2^ Department of Chemistry Faculty of Science Mansoura University El Gomhouria, Mansoura Qism 2, Dakahlia Governorate, 11432 Mansoura Egypt; ^3^ Theoretical Chemistry, Department of Chemistry and Pharmaceutical Sciences Vrije Universiteit Amsterdam De Boelelaan 1108, 1081 HZ Amsterdam The Netherlands; ^4^ Institute for Molecules and Materials Radboud University Heyendaalseweg 135, 6525 AJ Nijmegen The Netherlands; ^5^ Department of Chemical Sciences University of Johannesburg Auckland Park Johannesburg 2006 South Africa; ^6^ Department de Química Inorgànica i Orgànica & IQTCUB Universitat de Barcelona, ICREA Barcelona Spain

**Keywords:** Bismuth, Cationic species, Ethylene activation, Reversibility, Small molecule activation

## Abstract

Owing to its fundamental properties as the parent olefin, its outstanding industrial relevance, and the challenges associated with its activation, ethylene remains a benchmark substrate, especially in main group chemistry. Here we report the unprecedented activation of ethylene and related simple α‐olefins by well‐defined cationic bismuth complexes. The polarization of a substrate by a softly Lewis‐acidic central atom and a nucleophilic functional group positioned in a constrained geometry is for the first time exploited in the activation of ethylene and related olefins by compounds of a heavy p‐block element. Mechanistic investigations point toward a coordination‐insertion‐reaction mechanism. The bonding properties of the cationic bismuth species facilitate unprecedented reversible reactivity patterns and unusual characteristics in chemoselectivity.

## Graphical abstract

Well‐defined cationic bismuth compounds have been exposed to simple α‐olefins, including the benchmark substrate ethylene. Unprecedented and facile insertion, extrusion, and olefin exchange scenarios have been realized. The scope, mechanism, and functional group tolerance of these peerless transformations have been investigated in a combined experimental and theoretical approach.

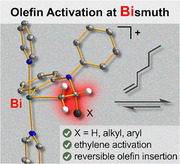

Ethylene and α‐olefins are of paramount importance as industrial carbon feedstocks for the production of pharmaceuticals, fine chemicals, and various polymers.^[^ ^1^, ^2^, ^3^, ^4^, ^5^, ^6^, ^7^, ^8^ ^]^ Traditionally, transition metals and their complexes are utilized to coordinate olefins, which results in the electronic activation of these substrates, facilitating the subsequent attack by external nucleophiles.^[^ ^9^, ^10^, ^11^, ^12^ ^]^ The ability of transition metals to (reversibly) bind and to insert alkenes is vital to catalytic cycles including hydrogenation, hydroelementation, isomerization, and polymerization.^[^ ^13^ ^]^ Over the past few decades, research in main‐group chemistry has shed light on the transition metal‐like reactivity of s‐ and p‐block elements.^[^ ^14^, ^15^, ^16^ ^]^ In recent years, major advances have been reported in the exploitation of well‐defined compounds of heavy p‐block elements for small molecule activation.^[^ ^17^ ^]^ Specifically, i) unusual coordination numbers and coordination geometries become accessible for compounds with heavy p‐block elements as central atoms,^[^ ^18^, ^19^, ^20^, ^21^, ^22^, ^23^ ^]^ ii) the bonding and electronic structure of low‐valent and multiply bonded species gradually changes when increasing the principle quantum number of the orbitals that are decisively involved in bond formation,^[^ ^24^, ^25^, ^26^, ^27^ ^]^ and iii) bonds involving heavy p‐block elements tend to be relatively weak,^[^ ^28^ ^]^ i.e., after initial small molecule activation, there is a rich potential for intriguing follow‐up reactions.^[^ ^17^, ^29^, ^30^, ^31^, ^32^ ^]^

The activation of simple alkenes bearing only alkyl substituents and the activation of ethylene in specific still pose a sizable challenge in main group chemistry. The first report of ethylene activation by a main group compound was reported by Power and co‐workers, who employed a distannyne to undergo a reversible addition reaction with ethylene.^[^ ^33^ ^]^ This triggered the development of intriguing strategies toward the activation of ethylene (and other simple olefins) by main group compounds.^[^ ^32^, ^33^, ^34^, ^35^, ^36^, ^37^, ^38^, ^39^, ^40^, ^41^, ^42^, ^43^, ^44^, ^45^, ^46^, ^47^, ^48^, ^49^, ^50^ ^]^ For compounds of heavy p‐block elements with a principal quantum number of *n* > 3, a very limited number of compounds is capable of ethylene activation. Three main strategies have been employed. First, multiply bonded species have been exploited following Power's pivotal study.^[^ ^39^, ^51^, ^52^ ^]^ Secondly, low‐valent compounds, such as germylenes and stannylenes, have allowed for ethylene activation covering modes of activation that lead to the formation of either metalla‐cyclopropane or 1,2‐dimetal‐ethylene structural motifs.^[^ ^31^, ^32^, ^53^, ^54^, ^55^, ^56^ ^]^ As a third approach, a cluster compound, namely a low‐valent tin cubane, has been exploited for ethylene insertion.^[^ ^57^ ^]^ All these approaches have heavily been focused on the chemistry of group 14 elements, with few notable exceptions.^[^ ^38^, ^41^, ^42^, ^43^, ^46^, ^48^, ^49^, ^51^ ^]^ Especially, compounds of heavy group 15 elements have only scarcely been utilized for ethylene activation. The addition of a low‐valent Sb(I) compound to ethylene has recently been reported.^[^ ^43^ ^]^ Recent studies in antimony chemistry have also evidenced that C═C bond insertions of olefins into the Sb─H bond are feasible.^[^ ^58^ ^]^ For bismuth compounds, rare examples of insertion reactions with olefins have been witnessed but were strictly limited to activated substrates such as acrylonitrile.^[^ ^59^, ^60^ ^]^ Cationic bismuth compounds have granted access to an unprecedented Lewis‐acid activation of simple olefins but did not proceed to the stage of olefin insertion.^[^ ^61^ ^]^ The activation of electron‐poor olefinic C═C bonds by simple bismuth halide salts, prior to an attack by soft nucleophiles leading to the formation of alkylbismuth species was also proposed.^[^ ^62^ ^]^ Very recently, the addition of a bismuth hydride to ethylene was suggested to proceed in 4% spectroscopic yield with in situ ^1^H NMR spectroscopy as the only means of experimental analysis, granting a first glimpse at the potential of bismuth compounds to be exploited in the transformation of ethylene and related simple olefins.^[^ ^44^ ^]^

Differing from previous concepts for ethylene activation by multiply‐bonded and low‐valent compounds of heavy p‐block elements, we present the reversible insertion of simple olefins H_2_C═CHR (R = H, alkyl, aryl) into the Bi─N bond of a Lewis acidic, hypercoordinate cationic bismuth amide.

Aiming at the activation of simple olefins with well‐defined bismuth compounds, the cationic bismuth amides **1‐Ph** ^[^ ^63^, ^64^ ^]^ and **1‐*i*Pr** ^[^ ^65^ ^]^ were identified as promising candidates in view of their pronounced reactivity, resulting from their Lewis‐acidic and ring‐strained properties (Scheme 1). As a benchmark reaction for C═C bond activation, compound **1‐Ph** was exposed to ethylene (1 bar) in pyridine at 60 °C. In initial experiments, a conversion of **1‐Ph** was indicated by a color change of the solution from deep red to yellow. ^1^H NMR spectroscopic studies showed that the formation of a potential ethylene insertion product (with two triplets for the two CH_2_ groups as characteristic signals) was competing with the formation of the previously reported product of a CH‐activation reaction (**A**,^[^ ^63^ ^]^ Scheme 1b). Striving to favor the ethylene activation pathway, the reaction was performed at higher pressure of 20 bar H_2_C═CH_2_ in an autoclave. After work‐up and re‐crystallization from difluorobenzene/pentane, compound **2‐Ph** could be isolated as a pale‐yellow solid in 56% yield (Scheme 1a). This is the first example of a bismuth compound to show unambiguous ethylene activation and the first example of ethylene activation with a compound of a heavy p‐block element, which does not exploit low‐valent or multiply bonded species. The NMR spectra of **2‐Ph** in THF‐*d*_8_ support the formation of a well‐defined mononuclear species and show two distinct triplet signals for the inserted CH_2_ groups at 2.45 and 5.81 ppm (^1^H) and singlets at 60.5 and 47.4 ppm (^13^C). Two‐dimensional correlation NMR spectroscopy confirms that the CH_2_ group with its resonances at 5.81/ 47.4 ppm is bound to the N‐atom, while the other CH_2_ group shows a spatial proximity to bismuth‐bound pyridine ligands, giving evidence of ethylene insertion into the Bi─N bond. Single‐crystal X‐ray diffraction analysis of **2‐Ph** (monoclinic space group C2/c, *Z* = 8; Figure 1a) confirmed the structural assignment based on solution NMR spectroscopy. In the solid state, compound **2‐Ph** forms a typical molecular structure without significant directional intermolecular interactions. The insertion of the C═C double bond of ethylene into the Bi─N bond of the four‐membered BiC_2_N cycle in **1‐Ph** generates release of ring strain to give a six‐membered, puckered BiC_4_N cycle. The low tendency of carbon‐based ligands to adopt bridging coordination modes with bismuth central atoms results in a mononuclear structural motif. The central atom Bi1 shows main bonding interactions with two carbon atoms of the dianionic ligand framework and two pyridine ligands, the bond lengths of Bi1─C1 2.246 Å, Bi1─C10 2.245 Å and Bi1─N2 2.498 Å, Bi1─N3 2.543 Å being typical for cationic bismuth species.^[^ ^60^ ^]^ In addition, geometric parameters suggest a very weak Bi⋯OTf interaction with pronounced ionic contributions (Bi1⋯O1, 3.558 Å, just below the sum of the van der Waals radii (3.59 Å); O1···Bi1─C10, 147.46°). Overall, this results in a distorted square pyramidal coordination geometry around Bi1 (*τ*_5_ = 0.39) with C1 in the apical position and the pyridine ligands in a *trans*‐configuration. The transformation of the former C═C double bond of ethylene into a C1─C2 single bond in compound **2‐Ph** is well in line with the interatomic C1─C2 distance of 1.520 Å.

**Scheme 1:**
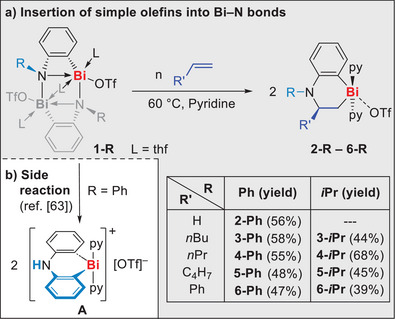
a) Reactions of **1‐R** ethylene and simple α‐olefins to give insertion products **2‐R** (*n* = 20 bar for R’ = H; *n* = ca. 100 equiv for the remaining cases). b) Side reaction of **1‐Ph** to give compound **A**.^[^ ^63^ ^].^

**Figure 1:**
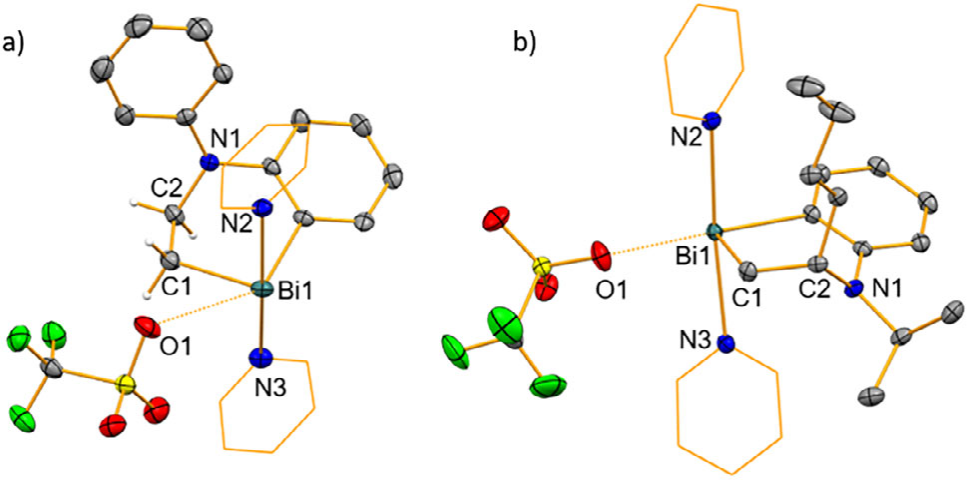
Molecular structure of compound a) **2‐Ph** and b) **3‐*i*Pr** in the solid state. Displacement ellipsoids are shown at the 50% probability level, C atoms of pyridine ligands are shown as a wireframe. For **2‐Ph**, a lattice‐bound C_6_H_4_F_2_ molecule and for **2‐Ph** and **3‐iPr**, hydrogen atoms (except for those in C_2_H_4_) are omitted for clarity. Selected bond lengths [Å] for **2‐Ph**: Bi1─C1 2.246(5), Bi1─C10 2.245(4), Bi1─N2 2.498(3), Bi1─N3 2.543(3), C1─C2 1.520(7), C2─N1 1.458(6). Selected bond angles for **2‐Ph** (°): C1─Bi1─C10 91.58(2), N2─Bi1─N3 171.05(1). Selected bond lengths [Å] for **3‐*i*Pr**: Bi1─C1 2.247(2), Bi1─C10 2.228(2), Bi1─N2 2.576(2), Bi1─N3 2.491(2), C1─C2 1.526(3), C2─N1 1.484(3). Selected bond angles for **3‐*i*Pr** (°): C1─Bi1─C10 90.99(8), N2─Bi1─N3 172.07(7).

Aiming to expand the unprecedented insertion of ethylene into a Bi─N bond to related challenging substrates, reactions of **1‐Ph** and **1‐*i*Pr** with nonactivated olefins were performed. Reactions with 1‐hexene and 1‐pentene readily gave the desired insertion products **3‐R** and **4‐R** (R = Ph, *i*Pr). 1,5‐Hexadiene as a substrate allowed for the selective formation of the mono‐insertion product **5‐R** in both cases. For the activated substrate styrene, insertion was favored over polymerization,^[^ ^66^, ^67^, ^68^ ^]^ allowing for the isolation of compounds **6‐R**. Compounds **3‐R** – **6‐R** were isolated in yields of 44%–68% and characterized in detail (Supporting Information). Note that for acceptable isolated yields, an excess of the olefin had to be employed, and that bulky terminal olefins or internal olefins did not undergo insertion (vide infra and Supporting Information). In successful insertion events, the bismuth atom is added to the terminal carbon atom of the substrate, in agreement with the polarity of the Bi^δ+^─N^δ−^ bond. The ^1^H NMR spectroscopic signal for CH_2_ group appears upfield as two doublets of doublets at 2.45 to 3.31 ppm, while the signals for CHR group are located downfield at 5.57 to 7.46 ppm. Selected representatives were subjected to single‐crystal X‐ray diffraction analysis, revealing qualitatively identical coordination geometries and bonding parameters. A representative example (**3‐*i*Pr**) is shown in Figure 1b, additional examples (**3‐Ph**, **5‐*i*Pr**) can be found in the Supporting Information. All cases show a (slightly to significantly) distorted square pyramidal coordination geometry around bismuth, with weak Bi···O^OTf^ interactions (according to distance criteria), a *trans*‐arrangement of the pyridine ligands, and the CH_2_ group in the apical position. The puckering of the six‐membered BiC_4_N ring can be tracked down to the carbon atoms of the former olefin units being located above the plane defined by the Bi─C_6_H_4_─N unit.

**Figure 2:**
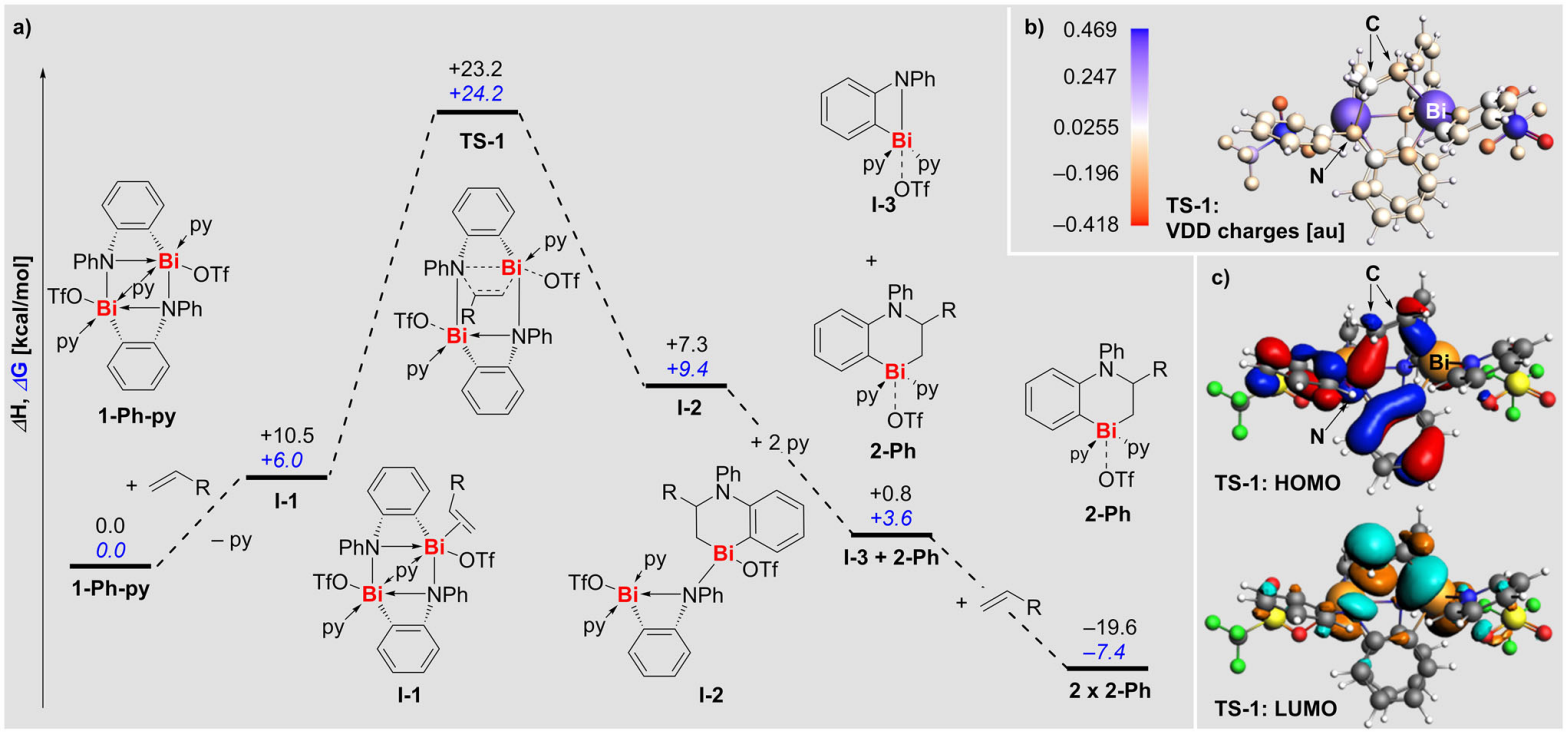
a) Calculated mechanism for the olefin insertion reaction with **1‐Ph**. ∆*G* values are shown in italics blue font and **∆** *H* values are shown in black regular font, calculated with respect to reactant **1‐Ph‐py**. The energies in the figure corresponds to R = H. For other olefins, see Table S5. Computed at ZORA‐BLYP‐D3(BJ)/TZP in pyridine. Diagram not in scale. b) VDD charges in **TS‐1**. c) Frontier orbitals of **TS‐1** at an isovalue of 0.03.

The mechanism of olefin insertion into the Bi─N bond was investigated by DFT calculations (Figure 2a; for details, see Supporting Information). All enthalpies and Gibbs free energies are reported relative to the original reactant **1‐Ph‐py**. For the discussion, we will focus on ∆*G* values. The initiating step is mildly endergonic (+6.0 kcal mol^−1^) and represents the substitution of one pyridine ligand in **1‐Ph‐py** for ethylene to give the intermediate **I‐1**. The insertion occurs in a concerted manner through the transition state **TS‐1** (+24.2 kcal mol^−1^), in which a new C─N bond and a new C─Bi bond between the reactants are forming while the C═C double bond of the ethylene converts into a single C─C bond and the Bi─N bond of **1‐Ph‐py** dissociates. The polar nature of the RBi^+^─NR_2_ structural motif in **I‐1** translates into a considerable polarization of the olefin in **TS‐1**. This is reflected by Voronoi deformation density (VDD) charges of +0.020 au and −0.136 au for the olefinic carbon atoms, along with a positive charge of +0.413 au at the neighboring bismuth atom and a negative charge of −0.152 au at the adjacent nitrogen atom (Figure 2b and Supporting Information). A frontier orbital analysis reveals that the HOMO reflects σ(Bi─C) and σ(N─C) bond formation via p‐type atomic orbitals, while the LUMO shows significant contributions by p‐type atomic orbitals of the positively charged carbon atom and the bismuth atom (Figure 2c). The reaction proceeds to give the intermediate **I‐2** (+9.4 kcal mol^−1^). At this stage, one olefin insertion per two bismuth atoms has been completed. The dinuclear intermediate liberates one equivalent of the product **2‐Ph** by pyridine coordination, along with the formation of the mononuclear intermediate **I‐3** (+3.6 kcal mol^−1^). From a mechanistic point of view, intermediate **I‐3** can dimerize according to the exergonic reaction: **I‐3** → 0.5 **1‐Ph‐py** + 0.5 py (−7.3 kcal mol^−1^), forming the initial starting material **1‐Ph‐py**, which can be fed back into this synthetic cycle (not shown in Figure 2). The direct formation of **2‐Ph** from **I‐3** is also possible but likely kinetically disfavored (Supporting Information). It should be noted that the overall olefin insertion reaction **1‐Ph‐py** + 2 H_2_C═CHR + py → 2 **2‐Ph** is moderately exergonic for ethylene (−7.4 kcal mol^−1^; R  =  H), but shows a Δ*G* value close to zero for 1‐hexene (+0.5 kcal mol^−1^; R  =  *n*Bu), in agreement with experimental observations. The mechanistic investigations reveal that the insertion of i) internal and ii) bulky olefins is not hampered from an overall thermodynamic point of view, but due to the instability of **I‐2** (in case of i)) and due to the relative stability of **I‐1** (in the case of ii), for details see Supporting Information).

The reversible binding of olefins to metal centers plays a crucial role in catalytic cycles.^[^ ^69^ ^]^ The equilibrium reaction of olefins binding to metal centers is a key step in catalytic cycles such as olefin metathesis,^[^ ^70^ ^]^ polymerization,^[^ ^71^ ^]^ and isomerization.^[^ ^72^ ^]^ The mildly exergonic character and moderate kinetic barriers of the olefin insertion reactions prompted us to investigate the reversibility of these transformations. Looking at the kinetic and thermodynamic data obtained from DFT studies, the reaction **1‐Ph‐py** + 2 styrene + py ⇌ 2 **6‐Ph** was most promising in this respect (**TS‐1**, ∆*H*  =  +19.4, ∆*G*  =  +26.0; overall reaction ∆*H*  =  −12.5, ∆*G*  =  +4.7). Long‐term investigations of the behavior of **6‐Ph** in pyridine‐*d*_5_ indicated a color change of the solution after one day at room temperature from colorless to red. ^1^H NMR spectroscopic analyses revealed that, indeed, styrene (5%), **1‐Ph** (3%), and compound **A** (2%) were present in the reaction mixture along with **6‐Ph** (90%) (Scheme 2a). This indicates a reversible reaction where **6‐Ph** was being converted back into starting the materials styrene and **1‐Ph**. Under the given reaction conditions, **1‐Ph** tautomerizes to give compound **A**, thus depleting the concentration of **1‐Ph** over time. After three weeks, a full conversion of **6‐Ph** into free styrene and compound **A** was observed.

**Scheme 2:**
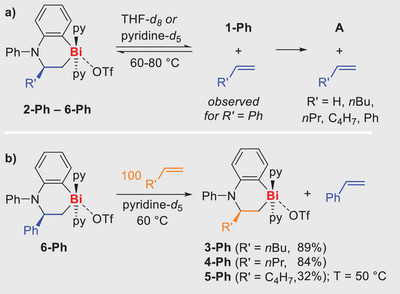
a) The isolated olefin insertion products **2‐Ph**–**6‐Ph** eliminate the hydrocarbons CH_2_═CHR’, showcasing reversible olefin insertion into Bi─N bonds. b) Olefin exchange reactions between **6‐Ph** (R’ = Ph) and CH_2_═CHR’ to give **3‐Ph**–**5‐Ph** (R’ = *n*Bu, *n*Pr, C_4_H_7_).

At room temperature under an argon atmosphere, pyridine‐*d*_5_ solutions of **2‐Ph**, **3‐Ph, 4‐Ph,** and **5‐Ph** were stable for several days. When the temperature was raised to 80 °C, compounds **3‐Ph**, **4‐Ph**, and **5‐Ph** released 1‐hexene, 1‐pentene, and 1,5‐hexadiene, respectively. The release of olefin was accompanied by the formation of compound **A** in all three cases, with a full conversion being accomplished after 11 days. Contrary to the observations with the styrene inserted product **6‐Ph**, the starting material **1‐Ph** was not observed in the ^1^H NMR spectra. In comparison to its alkyl‐substituted congeners, the ethylene insertion product **2‐Ph** was remarkably stable in pyridine‐*d*_5_ at 80 °C. No release of olefin nor any change in the spectrum was observed even after heating for 14 days. However, changing the solvent from pyridine to THF allowed for ethylene extrusion: heating **2‐Ph** to 80 °C in THF‐d_8_ in a closed vessel facilitated ethylene release (17%), along with the formation of compound **A** (36%) and other, yet unidentified decomposition products.

To transfer the reversible character of Bi─N olefin insertion to productive intermolecular reactions, a 100‐fold excess of 1‐hexene was added to a solution of the styrene insertion product **6‐Ph** in pyridine‐*d*_5_ (Scheme 2b). Heating the solution to 60 °C for 16 h, allowed for the smooth conversion into the hexene insertion product **3‐Ph** with 89% yield according to NMR spectroscopic analyses. Minor quantities (11%) of compound **A** were also detected. The analogous reaction of **6‐Ph** with excess 1‐pentene and 1,5‐hexadiene were also successful.

The chemoselective differentiation between unsaturated functional groups is of paramount importance in method development and synthetic applications. While transformations with late transition metals such as Ni, Cu, Rh, and Pd can favor the activation of olefins over carbonyl functional groups,^[^ ^73^, ^74^, ^75^, ^76^, ^77^ ^]^ the opposite tends to be true for compounds based on s‐block and lighter p‐block metals.^[^ ^78^, ^79^, ^80^, ^81^, ^82^, ^83^, ^84^ ^]^ To gain first insights into the chemoselectivity of carbonyl versus olefin insertion into Bi─N bonds, **1‐Ph** was reacted with equimolar amounts of 1‐hexene and aldehydes, ketones, amides, or esters in competition experiments (Scheme 3). While the highly reactive aldehyde outperformed the alkene as a substrate, in all the remaining cases, the formation of **3‐Ph** was clearly favored, which can in part be ascribed to the softly Lewis‐acidic nature of the cationic bismuth center, showing a preference for the activation of the softly Lewis‐basic olefin substrate.

**Scheme 3:**
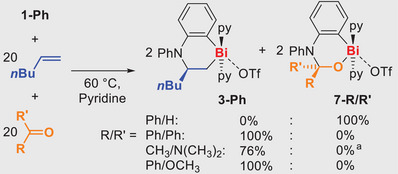
Competition reactions of **1‐Ph** with 1‐hexene versus a range of carbonyl compounds. a) the formation of **A** with 24% spectroscopic yield was observed.

In conclusion, we have unambiguously demonstrated the peerless activation of ethylene by a well‐defined bismuth complex. The reactivity of a softly Lewis‐acidic central atom in spatial proximity to a nucleophilic functional group has for the first time successfully been exploited for ethylene activation by a compound of a heavy p‐block element. This mode of action was equally effective for a range of activated and nonactivated α‐olefins. Remarkably, these insertion reactions of olefins into Bi─N bonds can be guided into reversible scenarios, allowing for olefin release. The dynamic bonding scenario also enables olefin exchange reactions, mimicking olefin coordination chemistry that is well‐established for transition metal complexes.

## Supporting Information

The authors have cited additional references within the Supporting Information.^[^ ^85^, ^86^, ^87^, ^88^, ^89^, ^90^, ^91^, ^92^, ^93^, ^94^, ^95^, ^96^, ^97^, ^98^, ^99^, ^100^, ^101^ ^]^

## Conflict of Interests

The authors declare no conflict of interest.

## Supporting information



Supporting Information

2‐Ph.cif

3‐iPr.cif

3‐Ph.cif

5‐iPr.cif

## Data Availability

The data that support the findings of this study are available in the Supporting Information of this article.
