# Supplementary material for: Bismuth Meets Olefins: Ethylene Activation and Reversible Alkene Insertion into Bi─N Bonds
Source: Angew Chem Int Ed Engl. 2025 May 8;64(26):e202505434. doi: 10.1002/anie.202505434 (PMC12184319; doi:10.1002/anie.202505434)
Supplement: Supplementary file 1 — Supporting Information [file ANIE-64-e202505434-s005.pdf]

**Table of Contents**

Experimental…… 2

NMR Spectra of Isolated Compounds…… 12

Test reactions with additional olefins…… 28

Test reactions for the release of olefins…… 29

Exchange/competition experiments…… 41

DFT calculations…… 47

Single-Crystal X-ray Analyses…… 113

Acknowledgements…… 118

## Experimental

### General considerations

All air and moisture-sensitive manipulations were carried out using standard Schlenk techniques or in a glovebox containing purified argon. Solvents were purified by distillation using the appropriate drying agents, degassed and stored over molecular sieves prior to use (3 Å for acetonitrile, 4 Å for other solvents used here). Deuterated solvents used for NMR spectroscopy were dried, degassed and stored over molecular sieves (3 Å or 4 Å, see above) under dry argon prior to use. $BiCl_3$ was sublimed prior to use. All liquid olefins were degassed by three freeze-pump-thaw cycles and stored over molecular sieves (4 Å) under dry argon. Compounds **1-Ph**[63] and **1-*i*Pr**[65] were prepared according to the literature.

All NMR spectra were acquired either on a Bruker Avance 300 spectrometer or on Bruker Avance I/III 500 spectrometer. $^{1}H$ and $^{13}C$ chemical shifts are reported relative to $SiMe_4$ using the residual solvent peak of the solvent as a secondary standard. $^{19}F$ chemical shifts are reported relative to $CFCl_3$ as an external standard. Elemental analyses (C, H, N, S) were conducted on Vario Micro Cube instruments by Elementar Analysesysteme GmbH. HR-ESI mass spectra were acquired with an Orbitrap Q Exactive plus mass spectrometer (Thermo Fischer Scientific), with a resolution set to 140,000. HR-FD/FI/LIFDI mass spectra were acquired with an AccuTOF GCv 4G (JEOL) Time of Flight (TOF) mass spectrometer. An internal or external standard was used for drift time correction. The LIFDI ion source and FD emitters were purchased from Linden ChroMasSpec GmbH (Bremen, Germany). Single-crystals suitable for X-ray diffraction analysis were coated with perfluorinated polyether oil in a glovebox, transferred to a nylon loop and then transferred to the goniometer of a diffractometer (Bruker D8 Quest or Bruker D8 Venture) equipped with a molybdenum X-ray tube (λ = 0.71073 Å). The structures were solved using Olex2[85], with XT structure solution program[86] and refined with the XL refinement package.[87],[101]

**Synthesis of ethene insertion product - [Bi(N($C_6H_5$)($C_6H_4$)($CH_2CH_2$))(OTf)($NC_5H_5$)$_2$] (2-Ph)**

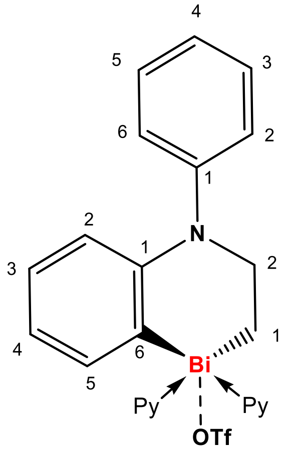


[$Bi_2$(NPh($C_6H_4$))$_2$(OTf)$_2$(thf)$_3$] (**1-Ph**) (275 mg, 0.30 mmol) was dissolved in pyridine (7 mL) in an autoclave (volume: 100 mL). An atmosphere of ethylene (20 bar) was applied, and the reaction mixture was heated to 60 °C for 3 hours. The reaction mixture was allowed to stir at room temperature for another 18 hours in the autoclave. All volatiles were removed under reduced pressure and a yellow oil was obtained. It was dissolved in THF (1 mL), and excess of pentane (7 mL) was added to precipitate a yellow residue. The yellow oil was washed with pentane (2 × 5 mL) and dried *in vacuo* for 3 hours to give a yellow powder. The powder was dissolved in difluorobenzene (3 mL), layered with an equal volume of pentane, and stored at –30 °C for 72 hours to afford yellow crystals. The crystals were isolated by filtration, washed with pentane (2 × 3 mL), and dried in vacuum for 3 hours. Compound **2-Ph** was obtained as a yellow powder.

Yield = 172 mg, 0.24 mmol, 56%.

**$^1$H NMR** (298 K, 500 MHz, THF-*d$_8$*): $\delta$ = 2.45 (t, 2H, $^3J_{HH}$ = 5.8 Hz, 1-$CH_2$), 5.81 (t, 2H, $^3J_{HH}$ = 5.9 Hz, 2-$CH_2$), 6.67-6.72 (m, 3H, 2,4,6-$C_6H_5$), 6.95-7.00 (m, 2H, 3,5-$C_6H_5$), 7.23-7.27 (m, 2H, 3,4-$C_6H_4$), 7.30-7.34 (m, 4H, 3,5-pyridine), 7.59-7.64 (m, 1H, 2-$C_6H_4$), 7.77-7.82 (m, 2H, 4-pyridine), 8.48-8.51 (m, 1H, 5-$C_6H_4$), 8.51-8.55 (m, 4H, 2,6-pyridine) ppm.

**$^{13}$C NMR** (298 K, 126 MHz, THF-*d$_8$*): $\delta$ = 47.4 (s, 2-*C*$H_2$), 60.5 (s, 1-$CH_2$), 119.3 (s, 2,6-$C_6H_5$), 120.8 (s, 4-$C_6H_5$), 121.5 (q, $^1J$CF = 321 Hz, OTf), 125.6 (s, 4-$C_6H_4$), 125.8 (s, 3,5-pyridine), 125.9 (s, 2-$C_6H_4$), 130.0 (s, 3,5-$C_6H_5$), 131.4 (s, 3-$C_6H_4$), 138.6 (s, 4-pyridine), 140.2 (s, 5- $C_6H_4$), 147.8 (s, 1-$C_6H_4$), 150.4 (s, 2,6-pyridine), 154.1 (1-$C_6H_5$), 180.5 (s, 6-$C_6H_4$) ppm.

**$^{19}$F NMR** (298 K, 471 MHz, THF-*d$_8$*): $\delta$ = –78.94 (s, OTf) ppm.

**$^1$H NMR** (298 K, 300 MHz, Pyridine-*d$_5$*): $\delta$ = 2.56 (t, 2H, $^3J_{HH}$ = 5.9 Hz, 1-$CH_2$), 5.57 (t, 2H, $^3J_{HH}$ = 5.9 Hz, 2-$CH_2$), 6.68-6.72 (m, 2H, 2,6-$C_6H_5$), 6.85 (t, 1H, $^3J_{HH}$ = 7.28 Hz, 4-$C_6H_5$), 7.03-7.09 (m, 2H, 3,5-$C_6H_5$), 7.16-7.21 (m, 4 H, 3,5-pyridine), 7.29-7.39 (m, 2H, 3,4-$C_6H_4$), 7.52-7.59 (m, 2H, 4-pyridine), 7.67-7.70 (m, 1H, 2-$C_6H_4$), 8.60-8.63 (m, 1H, 6-$C_6H_4$), 8.68-8.70 (m, 4H, 2,6- pyridine).

**LIFDI-MS** (positive mode, DCM): found m/z = 553.037, calculated for $BiNC_{15}H_{13}F_3O_3S^+$ ([M-2 $C_5H_5N$]$^+$) m/z = 553.037.

**Elemental analysis.** Anal. calc. for $C_{25}H_{21}BiF_3N_3O_3S$ (709.11 g/mol): C, 42.20; H, 3.26; N, 5.91; found: C, 42.25; H, 3.58; N, 6.07.

**Synthesis of hexene insertion product - [$Bi(N(C_6H_5)(C_6H_4)(CH(C_4H_9)CH_2))(OTf)(NC_5H_5)_2$] (3-Ph)**

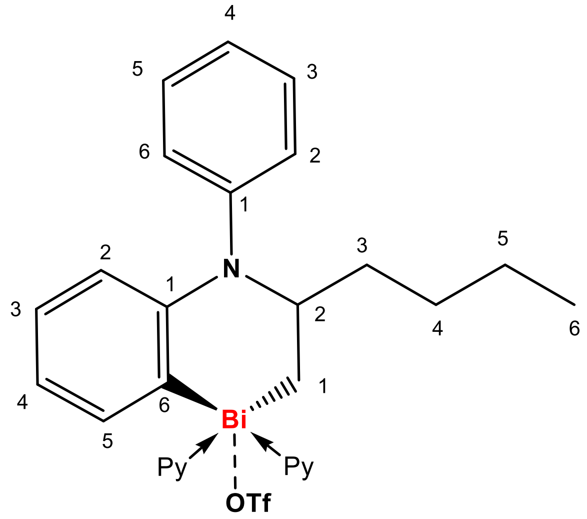


[$Bi_2(NPh(C_6H_4))_2(OTf)_2(thf)_3$] (**1-Ph**) (50 mg, 0.039 mmol) was dissolved in pyridine (2 mL). While stirring, an excess of 1-hexene (0.5 mL) was added. The reaction mixture was heated to 60 °C for 16 hours and a color change from red to pale yellow occurred. All volatiles were removed under vacuum and a yellow oil was obtained. It was dissolved in THF (2 mL), filtered to get a lime-yellow solution and further layered with hexane (1 mL) and stored at –30 °C. After 2 days, white block-like crystals formed from the solution and were characterised using SC-XRD. The crystals were isolated by filtration, washed with hexane (2 × 2 mL) and dried under vacuum for 2 hours. Compound **3-Ph** was obtained as a white powder.

Yield = 34.6 mg, 0.045 mmol, 58%.

**$^1$H NMR** (298 K, 500 MHz, THF-*d$_8$*): $\delta$ = 0.77 (t, 3H, $^3J_{HH}$ = 7.3 Hz, 6-$CH_3$), 1.08-1.24 (m, 2H, 5-$CH_2$), 1.26-1.44 (m, 2H, 4-$CH_2$), 1.45-1.64 (m, 2H, 3-$CH_2$), 2.25 (dd, 1H, $^2J_{HH}$ = 13.0 Hz, $^3J_{HH}$ = 2.1 Hz, 1-$CH_2$(a)), 2.88 (dd, 1H, $^2J_{HH}$ = 13.0 Hz, $^3J_{HH}$ = 6.1 Hz, 1-$CH_2$(b)), 6.64-6.70 (m, 3H, 2,4,6-$C_6H_5$), 6.74-6.80 (m, 1H, 2-CH), 6.92-6.96 (m, 2H, 3,5-$C_6H_5$), 7.26-7.34 (m, 6H, 3,4-$C_6H_4$, 3,5-pyridine), 7.72 (dd, 1H, $^3J_{HH}$ = 7.3 Hz, $^4J_{HH}$ = 2.0 Hz, 2-$C_6H_4$), 7.79 (tt, $^3J_{HH}$ = 7.7 Hz, $^4J_{HH}$ = 1.7 Hz, 4-pyridine), 8.54-8.56 (m, 4H, 2,6-pyridine), 8.62 (dd, 1H, $^3J_{HH}$ = 6.9 Hz, $^4J_{HH}$ = 2.2 Hz, 5-$C_6H_4$) ppm.

**$^{13}$C NMR** (298 K, 126 MHz, THF-*d$_8$*): $\delta$ = 14.40 (s, 6-$CH_3$), 23.45 (s, 5-$CH_2$), 30.02 (s, 4-$CH_2$), 35.39 (s, 3-$CH_2$), 54.54 (s, 2-CH), 68.09 (s, 1-$CH_2$), 118.93 (s, 2,6-$C_6H_5$), 120.22 (s, 4-$C_6H_5$), 120.0 (q, $^1J_{CF}$ = 321 Hz, OTf), 125.58 (s, 3,5-pyridine), 126.28 (s, 3-$C_6H_4$), 129.18 (s, 2-$C_6H_4$), 129.85 (s, 3,5-$C_6H_5$), 131.82 (s, 4-$C_6H_4$), 138.26 (s, 4-pyridine), 139.48 (s, 5-$C_6H_4$), 149.67 (s, 1-$C_6H_5$), 149.84 (s, 1-$C_6H_4$), 150.53 (s, 2,6-pyridine), 181.34 (s, 6-$C_6H_4$) ppm.

**$^{19}$F NMR** (298 K, 471 MHz, THF-*d$_8$*): $\delta$ = –78.94 (s, OTf) ppm.

**$^1$H NMR** (298 K, 300 MHz, pyridine-*d$_5$*): $\delta$ = 0.46 (t, 3H, $^3J_{HH}$= 7.3 Hz, 6-$CH_3$), 0.67-1.33 (m, 6H, 3,4,5-$CH_2$), 1.59 (m, THF), 2.49 (dd, 1H, $^2J_{HH}$ = 13.3 Hz, $^3J_{HH}$ = 1.7 Hz, 1-$CH_2$(a)), 3.15 (dd, 1H, , $^2J_{HH}$ = 13.3 Hz, $^3J_{HH}$ = 6.3 Hz, 1-$CH_2$(b)), 3.63 (m, THF), 6.35-6.41 (m, 1H, 2-CH), 6.62 (d, 2H, $^3J_{HH}$ = 7.9 Hz, 2,6-$C_6H_5$), 6.81 (t, 1H, $^3J_{HH}$ = 7.3 Hz, 4-$C_6H_5$), 7.02 (t, 2H, $^3J_{HH}$ = 7.9 Hz, 3,5- $C_6H_5$), 7.18 (m, 3,5-pyridine), 7.31-7.44 (m, 2H, 3,4-$C_6H_4$), 7.55 (m, 4-pyridine), 7.72 (dd, 1H, $^3J_{HH}$ = 8.0 Hz, $^4J_{HH}$ = 1.8 Hz, 2-$C_6H_4$), 8.70 (m, 2,6-pyridine), 8.75 (dd, 1H, $^3J_{HH}$ = 7.1 Hz, $^4J_{HH}$ = 1.7 Hz, 5-$C_6H_4$) ppm.

**$^{13}$C NMR** (298 K, 75 MHz, pyridine-*d$_5$*): $\delta$ = 14.21 (s, 6-$CH_3$), 22.97 (s, 5-$CH_3$), 26.26 (s, THF), 29.29 (s, 4-$CH_3$), 35.39 (s, 3-$CH_3$), 54.53 (s, 2-CH), 63.62 (s, 1-$CH_2$), 68.28 (s, THF), 119.22 (s, 2,6-$C_6H_5$), 120.45 (q,

$^{1}J_{CF}$ = 321 Hz, OTf), 120.91 (s, 4- $C_6H_5$), 126.25 (s, 4-$C_6H_4$), 129.05 (s, 1-$C_6H_4$), 130.49 (s, 3,5-$C_6H_5$), 132.43 (s, 3,5-$C_6H_5$), 138.99 (s, 5-$C_6H_4$), 149.11 (s, 1-$C_6H_5$), 174.47 (s, 6-$C_6H_4$) ppm.

**$^{19}$F NMR** (298 K, 282.39 MHz, pyridine-*d₅*): $\delta$ = -77.26 (s, OTf) ppm.

**ESI-MS** (positive mode, THF): found m/z: 460.147, calculated for $BiNC_{18}H_{21}^+$ [M-($O_3SCF_3^-$ + 2 $C_5H_5N$)]$^+$ m/z: 460.147.

**Elemental analysis.** Anal. calc. for $C_{29}H_{31}BiF_3N_3O_3S$ (767.62 g/mol): C, 45.38; H, 4.07; N, 5.47; S, 4.18; found: C, 45.30; H, 3.99; N, 5.47; S, 4.19.

**Synthesis of pentene insertion product - [Bi(N($C_6H_5$)($C_6H_4$)(CH($C_3H_7$)$CH_2$))(OTf)($NC_5H_5$)$_2$] (4-Ph)**

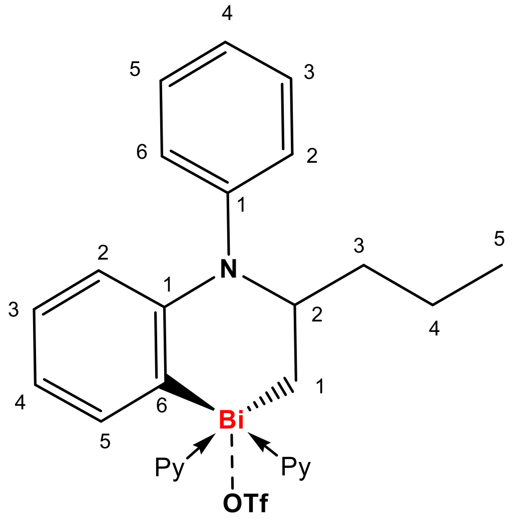


[$Bi_2$(NPh($C_6H_4$))$_2$(OTf)$_2$(thf)$_3$] **(1-Ph)** (50 mg, 0.039 mmol) was dissolved in pyridine (2 mL). While stirring, an excess of 1-pentene (0.5 mL) was added. The reaction mixture was heated to 60 °C for 16 hours and a color change from red to pale yellow occurred. All volatiles were removed under vacuum and a yellow oil was obtained. It was dissolved in THF (2 mL), filtered to get a lime-yellow solution and further layered with hexane (1 mL) and stored at –30 °C. After 2 days, white block-like crystals formed in the solution and were characterized using SC-XRD. The crystals were isolated by filtration, washed with hexane (2 × 2 mL), and dried under vacuum for 2 hours. Compound **4-Ph** was obtained as a white powder.

Yield = 32.6 mg, 0.043 mmol, 55%.

**$^{1}$H NMR** (298 K, 300 MHz, Pyridine-*d₅*): $\delta$ = 0.38 (t, 3H, $^{3}J_{HH}$= 7.1 Hz, 5-$CH_3$), 0.88-1.29 (m, 4H, 3,4-$CH_3$), 2.49 (dd, 1H, $^{2}J_{HH}$ = 13.4 Hz, $^{3}J_{HH}$ = 1.8 Hz, 1-$CH_2$(a)), 3.13 (dd, 1H, , $^{2}J_{HH}$ = 13.4 Hz, $^{3}J_{HH}$ = 6.2 Hz, 1-$CH_2$(b)), 6.34-6.40 (m, 1H, 2-CH), 6.61 (d, 2H, $^{3}J_{HH}$ = 7.9 Hz, 2,6-$C_6H_5$), 6.81 (t, 1H, $^{3}J_{HH}$ = 7.3 Hz, 4-$C_6H_5$), 7.01 (t, 2H, $^{3}J_{HH}$ = 7.9 Hz, 3,5- $C_6H_5$), 7.18 (m, 3,5-pyridine), 7.55 (m, 4-pyridine), 8.70 (m, 2,6-pyridine), 7.31-7.44 (m, 2H, 3,4-$C_6H_4$), 7.72 (dd, 1H, $^{3}J_{HH}$ = 7.9 Hz, $^{4}J_{HH}$ = 1.7 Hz, 2-$C_6H_4$), 8.75 (dd, 1H, $^{3}J_{HH}$ = 7.1 Hz, $^{4}J_{HH}$ = 1.7 Hz, 5-$C_6H_4$) ppm.

**$^{13}$C NMR** (298 K, 125 MHz, Pyridine-*d₅*): $\delta$ = 13.77 (s, 5-$CH_3$), 20.01 (s, 4-$CH_3$), 25.81 (s, THF), 37.40 (s, 3-$CH_3$), 53.83 (s, 2-CH), 63.00 (s, 1-$CH_2$), 67.84 (s, THF), 118.75 (s, 2,6-$C_6H_5$), 120.44 (q, $^{1}J_{CF}$ = 321 Hz, OTf), 120.45 (s, 4- $C_6H_5$), 125.78 (s, 4-$C_6H_4$), 128.50 (s, 1-$C_6H_4$), 129.59 (s, 2,6-C6H5), 131.98 (s, 3,5-$C_6H_5$), 138.61 (s, 5-$C_6H_4$), 148.62 (s, 1-$C_6H_5$), 174.10 (s, 6-$C_6H_4$) ppm.

**$^{19}$F NMR** (298 K, 282.39 MHz, Pyridine-*d*$_5$): $\delta$ = -77.28 (s, OTf) ppm.

**ESI-MS** (positive mode, THF): found m/z: 446.131, calculated for $[M-(O_3SCF_3 + 2\ C_5H_5N)]^+$ m/z: 460.132.

**Elemental analysis.** Anal. calc. for $C_{28}H_{29}BiF_3N_3O_3S$ (753.59 g/mol): C, 44.63; H, 3.88; N, 5.58; S, 4.25; found: C, 44.63; H, 3.94; N, 5.31; S, 4.24.

**Synthesis of 1,5-hexadiene insertion product - $[Bi(N(C_6H_5)(C_6H_4)(CH(C_4H_7)CH_2))(OTf)(NC_5H_5)_2]$ (5-Ph)**

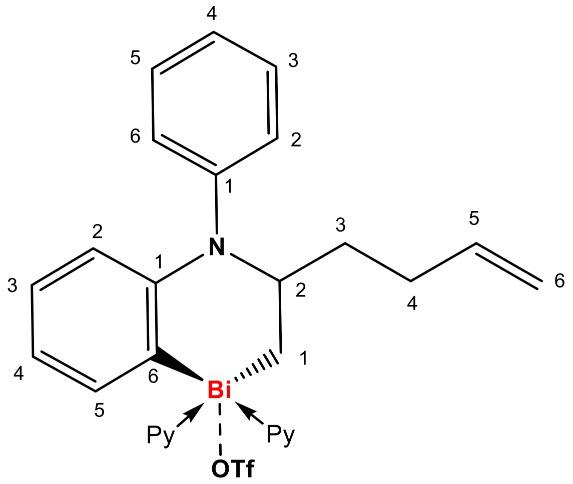


$[Bi_2(NPh(C_6H_4))_2(OTf)_2(thf)_3]$ **(1-Ph)** (50 mg, 0.039 mmol) was dissolved in pyridine (3 mL). While stirring, an excess of 1,5-hexadiene (0.5 mL) was added. The reaction mixture was heated to 60 °C for 16 hours and a colour change from red to light yellow occurred. All volatiles were removed under vacuum and a dark yellow oil was obtained. The oil was re-dissolved in 1 mL THF and an excess of hexane (6 mL) was added to it and stirred for 30 minutes. On stopping the stirring a dirty grey precipitate settled down, which was isolated and washed with (3 × 2 mL) pentane and dried for 3 hours in a vacuum. Compound **5-Ph** was obtained as cream-coloured powder.

Yield = 28.7 mg, 0.037 mmol, 48%.

**$^1$H NMR** (298 K, 300 MHz, Pyridine-*d*$_5$): $\delta$ = 1.07-1.19 (m, 1H, 3-CH$_2$(a)), 1.35-1.48 (m, 1H, 3-CH$_2$(b)), 1.85-1.91 (m, 2H, 4-CH$_2$), 2.50 (dd, 1H, $^2J_{HH}$ = 13.24 Hz, $^3J_{HH}$ = 1.74 Hz, 1-CH$_2$(a)), 3.15 (dd, 1H, $^2J_{HH}$ = 13.37 Hz, $^3J_{HH}$ = 6.29 Hz, 1-CH$_2$(b)), 4.65 (dd, 1H, $^2J_{HH}$ = 17.18 Hz, $^3J_{HH}$ = 1.72 Hz, 6-CH$_2$(a)), 4.73-4.77 (m, 1H, 6-CH$_2$(b)), 5.36-5.50 (m, 1H, 5-CH), 6.38-6.45 (m, 1H, 2-CH), 6.60 (d, 2H, $^3J_{HH}$ = 8.07 Hz, 2,6-C$_6$H$_5$), 6.80 (t, 1H, $^3J_{HH}$ = 7.23 Hz, 4- C$_6$H$_5$), 7.00(t, 2H, $^3J_{HH}$ = 7.00 Hz, 3,5- C$_6$H$_5$), 7.18 (m, 3,5-pyridine), 7.32-7.35 (m, 2H, 3,4- C$_6$H$_4$), 7.55 (m, 4-pyridine), 7.74 (dd, 1H, $^3J_{HH}$ = 7.88 Hz, $^4J_{HH}$ = 1.10 Hz, 2-C$_6$H$_4$), 8.70 (m, 2,6-pyridine), 8.76 (dd, 1H, $^3J_{HH}$ = 7.10 Hz, $^4J_{HH}$ = 1.67 Hz, 5-C$_6$H$_4$).

**$^{13}$C NMR** (298 K, 75 MHz, Pyridine-*d*$_5$): $\delta$ = 25.81 (s, THF), 30.69 (s, 4-CH$_3$), 34.31 (s, 3-CH$_3$), 53.29 (s, 2-CH), 62.95 (s, 1-CH$_2$), 67.83 (s, THF), 115.01 (s, 6-CH$_2$), 118.47 (s, 2,6- C$_6$H$_5$), 120.15 (q, $^1J_{CF}$ = 321 Hz, OTf), 120.35 (s, 4-C$_6$H$_5$), 126.04 (s, 4-C$_6$H$_4$), 128.60 (s, 5-C$_6$H$_4$), 129.56 (s, 3,5-C$_6$H$_5$), 132.02 (s, 3-C$_6$H$_4$), 137.87 (s, 5-CH), 138.58 (s, 2-C$_6$H$_4$), 148.51 (s, 1-C$_6$H$_5$), 174.17 (s, 6-C$_6$H$_4$) ppm.

**$^{19}$F NMR** (298 K, 282.39 MHz, Pyridine-*d*$_5$): $\delta$ = -77.27 (s, OTf) ppm.

**LIFDI-MS** (positive mode, THF): found m/z: 607.080, calculated for $[M-2\ C_5H_5N]^+$ m/z: 607.084.

**Elemental analysis.** Anal. calc. for $C_{29}H_{29}BiF_3N_3O_3S$ (765.60 g/mol): C, 45.49; H, 3.82; N, 5.49; found: C, 45.66; H, 3.98; N, 5.65.

**Synthesis of styrene insertion product - $[Bi(N(C_6H_5)(C_6H_4)(CH(C_6H_5)CH_2))(OTf)(NC_5H_5)_2]$ (6-Ph)**

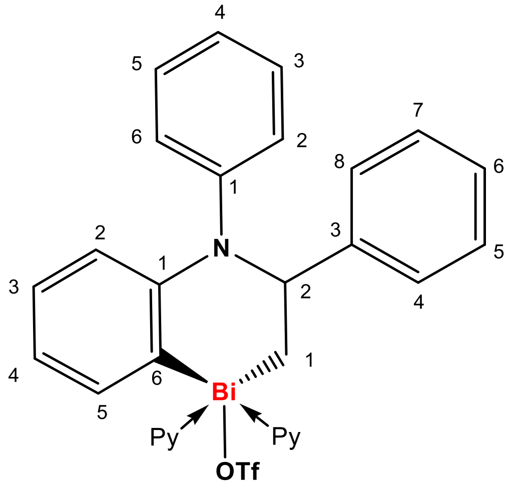


$[Bi_2(NPh(C_6H_4))_2(OTf)_2(thf)_3]$ **(1-Ph)** (50 mg, 0.039 mmol) was dissolved in pyridine (3 mL). While stirring, an excess of styrene (0.5 mL) was added. The reaction mixture was heated to 60 °C for 16 hours and a color change from red to bright orange occurred. All volatiles were removed under vacuum and an orange powder was obtained. The powder was re-dissolved in THF (1 mL) and an excess of hexane (6 mL) was added, and the resulting suspension was stirred for 30 minutes. On stopping the stirring a white precipitate settled down, which was isolated by filtration, washed with (3 × 2 mL) pentane and dried for 3 hours in vacuum. Compound **6-Ph** was obtained as white powder.

Yield = 29.5 mg, 0.037 mmol, 47%.

$^{1}$**H NMR** (298 K, 500 MHz, Pyridine-*d5*): *δ* = 3.07 (dd, 1H, $^{2}J_{HH}$ = 13.35 Hz, $^{3}J_{HH}$ = 5.85 Hz, 1-$CH_2$ (a)-styrene), 3.31 (dd, 1H, $^{2}J_{HH}$ = 13.35 Hz, $^{3}J_{HH}$ = 5.10 Hz, 1-$CH_2$ $CH_2$ (b)- styrene), 6.65 (d, 2H, 2,6-$C_6H_5$), 6.88-6.95 (m, 4H, 4-$C_6H_5$, 5,6,7-styrene), 7.05 (t, 2H, $^{3}J_{HH}$ = 7.85 Hz, 3,5-$C_6H_5$), 7.17-7.19 (m, 3,5-pyridine), 7.19-7.24 (m, 2H, 4,8-styrene), 7.29-7.35 (m, 3H, 2-CH-styrene, 3,4-$C_6H_4$), 7.53-7.57 (m, 4-Pyridine), 7.83 (d, 1H, $^{3}J_{HH}$ = 8.23 Hz, 2-$C_6H_4$), 8.45 (dd, 1H, 5-$C_6H_4$, $^{3}J_{HH}$ = 7.19 Hz, $^{4}J_{HH}$ = 1.54 Hz), 8.70 (m, 2,6-pyridine) ppm.

$^{13}$**C NMR** (298 K, 126 MHz, Pyridine-*d5*): *δ* = 60.56 (s, 2-CH-styrene), 61.58 (s, 1-$CH_2$-styrene), 119.2 (q, $^{1}J_{CF}$ = 268.6 Hz, OTf), 122.90 (s, 2,6- C6H5), 123.15 (s, 4-$C_6H_5$), 124.52 (s, 2-$C_6H_4$), 126.06 (s, 6-$C_6H_5$-Styrene), 127.44 (s, 4-$C_6H_4$), 128.44 (s, 5,7-$C_6H_5$- styrene), 129.00 (s, 1-$C_6H_4$), 130.14 (s, 3,5-$C_6H_5$), 132.68 (s, 3-$C_6H_4$), 136.49 (s, 4,8-$C_6H_5$-styrene), 139.76 (s, 5-$C_6H_4$), 144.05 (s, 3-$C_6H_5$-styrene), 149.46 (s, 1-$C_6H_5$), 153.03 (s, 1-$C_6H_5$), 171.90 (s, 6-$C_6H_4$).

$^{19}$**F NMR** (298 K, 282.51 MHz, Pyridine-*d5*): *δ* = -77.28 (s, OTf) ppm.

**LIFDI-MS** (positive mode, pyridine): found m/z: 629.090, calculated for $[M\text{-}2\ C_5H_5N]^+$ m/z: 629.068.

**Elemental analysis.** Anal. calc. for $C_{31}H_{27}BiF_3N_3O_3S$ (787.60 g/mol): C, 47.27; H, 3.45; N, 5.33; S, 4.07; found: C, 47.05; H, 3.68; N, 5.31; S, 3.71.

**Synthesis of hexene insertion product- [Bi(N($C_3H_7$)($C_6H_4$)(CH($C_4H_9$)$CH_2$))(OTf)($NC_5H_5$)$_2$] (3-*i*Pr)**

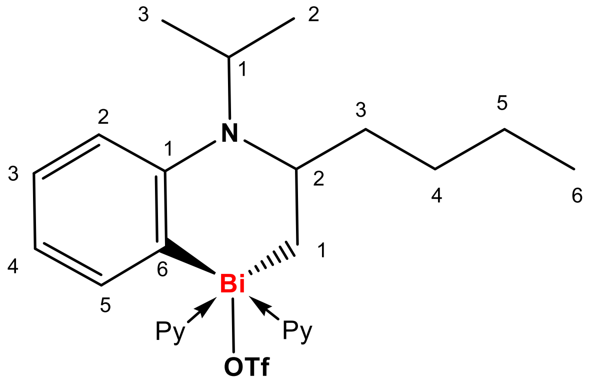


[$Bi_2$($NC_3H_7$($C_6H_4$))$_2$(OTf)$_2$(thf)$_3$] (**1-*i*Pr**) (70 mg, 0.071 mmol) was dissolved in pyridine (5 mL) and an excess of 1-hexene (1 mL) was added. The reaction mixture was then heated to 60 °C for 18 hours. The reaction mixture had changed color from red to yellow. All volatiles were removed in vacuo and the remaining residue was dissolved in pyridine (2 mL). The solution was filtered and layered with $Et_2O$ for crystallization. After 3 days yellow block-like crystals had formed and were characterized using SC-XRD. The crystals were isolated by filtration, washed with $Et_2O$ (2 × 2 mL), and dried under vacuum for 2 hours. Compound **3-*i*Pr** was obtained as a light-yellow powder.

Yield = 35 mg (0.048 mmol, 44%)

**$^1$H NMR** (298 K, 300 MHz, Pyridine-*d$_5$*): $\delta$ = 0.41 (t, 3H, $^3J_{HH}$= 2.3 Hz, 6-$CH_3$), 0.52 (d, 3H, $^3J_{HH}$= 6.6 Hz, 2-$CH_3$-iPr), 0.64-1.12 (m, 6H, 3,4,5-$CH_2$), 1.17 (d, 3H, $^3J_{HH}$ = 6.5 Hz, 3-$CH_3$-iPr), 2.60 (dd, 1H, , $^2J_{HH}$ = 12.9 Hz, $^3J_{HH}$ = 1.7 Hz, 1-$CH_2$(a)), ), 2.70 (dd, 1H, $^2J_{HH}$ = 12.9 Hz, $^3J_{HH}$ = 5.7 Hz, 1-$CH_2$(b)), 3.55 (sept, 1H, $^3J_{HH}$ = 6.5 Hz, 1-CH-iPr), 5.57-5.64 (m, 1H, 2-CH), 6.61 (d, 2H, $^3J_{HH}$ = 7.9 Hz, 2,6-$C_6H_5$), 7.13-7.18 (m, 1H, 4-$C_6H_4$), 7.18 (m, 3,5-pyridine), 7.47 (td, 1H, $^3J_{HH}$ = 11.4 Hz, $^4J_{HH}$ = 1.7 Hz, 3-$C_6H_4$), 7.55 (m, 4-pyridine), 7.72 (d, 1H, $^3J_{HH}$ = 8.1Hz, 2-$C_6H_4$), 8.50 (dd, $^3J_{HH}$ = 7.1Hz, $^4J_{HH}$ = 1.6Hz, 5-$C_6H_4$), 8.70 (m, 2,6-pyridine).

**$^1$H NMR** (298 K, 500 MHz, $CD_3CN$): $\delta$ = 0.66 (t, 3H, $^3J_{HH}$ = 7.1 Hz, 6-$CH_3$), 0.72 (d, 3H, $^3J_{HH}$ = 6.6 Hz, 2-$CH_3$-iPr), 0.81-1.17 (m, 6H, 3,4,5-$CH_2$), 1.25 (d, 3H, $^3J_{HH}$ = 6.5 Hz, 8-$CH_3$-iPr), 2.32 (dd, 1H, $^2J_{HH}$ = 12.5 Hz, $^3J_{HH}$ 2.5 Hz, 1-$CH_2$(a)), 2.47 (dd, $^2J_{HH}$ = 12.6 Hz, $^3J_{HH}$ = 6.0 Hz, 1-$CH_2$(b)), 3.74 (sept, 1H, $^3J_{HH}$ = 6.5 Hz, 1-CH-iPr), 5.75-5.80 (m, 1H, 2-CH), 7.04 (td, 1H, $^3J_{HH}$ = 7.2 Hz, $^4J_{HH}$ 1.1 Hz, 4-$C_6H_4$), 7.38 (td, 1H, $^3J_{HH}$ = 7.7 Hz, $^3J_{HH}$ = .7 Hz, 3-$C_6H_4$), 7.46 (m, 4H, 3,5-pyridine), 7.76 (d, 1H, $^3J_{HH}$ = 8.3 Hz, 2-$C_6H_4$), 7.94 (tt, 2H, $^3J_{HH}$ = 7.7 Hz, $^4J_{HH}$ = 1.8 Hz, 4-pyridine), 8.31 (dd, 1H, $^3J_{HH}$ = 7.2 Hz, $^4J_{HH}$ = 1.6 Hz, 5-$C_6H_4$), 8.47 (dt, 4H, $^3J_{HH}$ = 4.6 Hz, $^4J_{HH}$ =1.7 Hz, 2,6-pyridine) ppm.

**$^{13}$C NMR** (298 K, 125 MHz, $CD_3CN$): $\delta$ = 14.21 (s, 6-$CH_3$), 20.36 (s, 3-$CH_3$-iPr), 22.20 (s, 2-$CH_3$-iPr), 23.30 (s, 5-$CH_3$), 30.02 (s, 4-$CH_3$), 36.60 (s, 3-$CH_3$), 48.83 (s, 2-CH), 51.76 (s, 1-CH-iPr), 67.60 (s, 1-$CH_2$), 118.45 (q, $^1J_{CF}$ = 321 Hz, OTf), 120.71 (s, 2-$C_6H_4$), 121.97 (s, 4- $C_6H_4$), 126.63 (s, 3,5-pyridine), 133.14 (s, 3-$C_6H_4$), 139.52 (s, 5-$C_6H_4$), 140.08 (s, 4-pyridine), 149.95 (s, 2,6-pyridine), 152.6 (s, 1-$C_6H_4$), 171.9 (s, 6-$C_6H_4$) ppm.

**$^{19}$F NMR** (298 K, 282 MHz, Pyridine-*d$_5$*): $\delta$ = -77.26 (s, OTf) ppm.

**$^{19}$F NMR** (298 K, 282 MHz, $CD_3CN$): $\delta$ = -79.29 (s, OTf) ppm.

**Elemental analysis.** Anal. calc. for $C_{26}H_{33}BiF_3N_3O_3S$ (731.18 g/mol): C, 42.57; H, 4.53; N, 5.73; S, 4.37; found: C, 42.12; H, 4.35; N, 5.74; S, 4.03.

**Synthesis of pentene insertion product- $[Bi(N(C_3H_7)(C_6H_4)(CH(C_3H_7)CH_2))(OTf)(NC_5H_5)_2]$ (4-*i*Pr)**

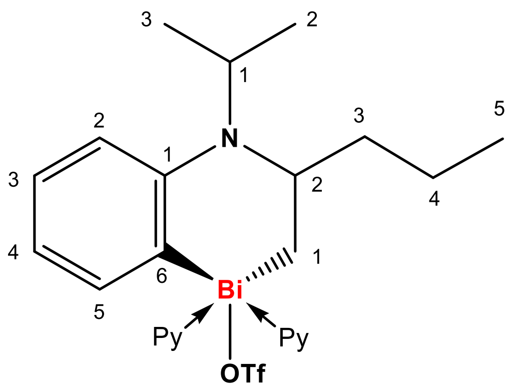


$[Bi_2(NC_3H_7(C_6H_4))_2(OTf)_2(thf)_3]$ (**1-*i*Pr**) (60 mg, 0.071 mmol) was dissolved in pyridine (3 mL) and an excess of 1-pentene (0.5 mL) was added. The reaction mixture was then heated to 60 °C for 18 hours. The reaction mixture had changed color from red to yellow. All volatiles were removed in vacuo and a yellow oil was obtained. The oil was re-dissolved in THF (1 mL) and an excess of pentane (8 mL) was added to it and stirred for 30 minutes. On stopping the stirring a dark yellow precipitate settled down, which was isolated, washed with pentane (3 × 2 mL), and dried for 3 hours in vacuum. Compound **4-*i*Pr** was obtained as cream-coloured powder.

Yield = 34.5 mg (0.048 mmol, 68%).

**$^1$H NMR** (298 K, 300 MHz, Pyridine-*d*$_5$): $\delta$ = 0.21 (t, 3H, $^3J_{HH}$= 7.1 Hz, 5-$CH_3$), 0.52 (d, 3H, $^3J_{HH}$= 6.6 Hz, 2-$CH_3$-iPr), 0.64-1.12 (m, 6H, 3,4,5-$CH_2$), 1.17 (d, 3H, $^3J_{HH}$ = 6.5 Hz, 3-$CH_3$-iPr), 2.60 (dd, 1H, , $^2J_{HH}$ = 12.9 Hz, $^3J_{HH}$ = 1.7 Hz, 1-$CH_2$(a)), ), 2.70 (dd, 1H , $^2J_{HH}$ = 12.9 Hz, $^3J_{HH}$ = 5.7 Hz, 1-$CH_2$(b)), 3.55 (sept, 1H, $^3J_{HH}$ = 6.5 Hz, 1-CH-iPr), 5.57-5.64 (m, 1H, 2-CH), 6.61 (d, 2H, $^3J_{HH}$ = 7.9 Hz, 2,6-$C_6H_5$), 7.13-7.18 (m, 1H, 4-$C_6H_4$), 7.18 (m, 3,5-pyridine), 7.47 (td, 1H, $^3J_{HH}$ = 11.4 Hz, $^4J_{HH}$ = 1.7 Hz, 3-$C_6H_4$), 7.55 (m, 4-pyridine), 7.72 (d, 1H, $^3J_{HH}$ = 8.1Hz, 2-$C_6H_4$), 8.50 (dd, $^3J_{HH}$ = 7.1Hz, $^4J_{HH}$ = 1.6Hz, 5-$C_6H_4$), 8.70 (m, 2,6-pyridine) ppm.

**$^1$H NMR** (298 K, 500 MHz, $CD_3CN$): $\delta$ = 0.56 (t, 3H, $^3J_{HH}$ = 7.0 Hz, 5-$CH_3$), 0.70 (d, 3H, $^3J_{HH}$ = 6.6 Hz, 2-$CH_3$-iPr), 0.99-1.16 (m, 4H, 3,4-$CH_2$), 1.25 (d, 3H, $^3J_{HH}$ = 6.5 Hz, 8-$CH_3$-iPr), 2.32 (dd, 1H, $^2J_{HH}$ = 12.6 Hz, $^3J_{HH}$ 2.5 Hz, 1-$CH_2$(a)), 2.47 (dd, $^2J_{HH}$ = 12.6 Hz, $^3J_{HH}$ = 6.0 Hz, 1-$CH_2$(b)), 3.74 (sept, 1H, $^3J_{HH}$ = 6.6 Hz, 1-CH-iPr), 5.79-5.86 (m, 1H, 2-CH), 7.04 (td, 1H, $^3J_{HH}$ = 10.8 Hz, $^4J_{HH}$ 1.1 Hz, 4-$C_6H_4$), 7.38 (td, 1H, $^3J_{HH}$ = 7.7 Hz, $^4J_{HH}$ = .7 Hz, 3-$C_6H_4$), 7.46 (m, 4H, 3,5-pyridine), 7.76 (d, 1H, $^3J_{HH}$ = 8.3 Hz, 2-$C_6H_4$), 7.94 (tt, 2H, $^3J_{HH}$ = 7.7 Hz, $^4J_{HH}$ = 1.8 Hz, 4-pyridine), 8.31 (dd, 1H, $^3J_{HH}$ = 7.2 Hz, $^4J_{HH}$ = 1.7 Hz, 5-$C_6H_4$), 8.47 (dt, 4H, $^3J_{HH}$ = 4.6 Hz, $^4J_{HH}$ =1.7 Hz, 2,6-pyridine) ppm.

**$^{13}$C NMR** (298 K, 125 MHz, $CD_3CN$): $\delta$ = 14.11 (s, 5-$CH_3$), 20.25 (s, 3-$CH_3$-iPr), 20.91 (s, 2-$CH_3$-iPr), 22.14 (s, 4-$CH_3$), 38.94 (s, 3-$CH_3$), 48.50 (s, 2-CH), 51.74 (s, 1-CH-iPr), 67.58 (s, 1-$CH_2$), 118.45 (q, $^1J_{CF}$ = 321 Hz, OTf), 120.48 (s, 2-$C_6H_4$), 121.82 (s, 4- $C_6H_4$), 126.84 (s, 3,5-pyridine), 133.14 (s, 3-$C_6H_4$), 139.60 (s, 5-$C_6H_4$), 140.42 (s, 4-pyridine), 149.86 (s, 2,6-pyridine), 153.66 (s, 1-$C_6H_4$), 172.99 (s, 6-$C_6H_4$) ppm.

**$^{19}$F NMR** (298 K, 282 MHz, $CD_3CN$): $\delta$ = –77.26 (s, OTf) ppm.

**LIFDI-MS** (positive mode, DCM): found m/z: 412.148, calculated for $[M-(2\ C_5H_5N+OTf)]^+$ m/z: 412.148.

**Elemental analysis.** Anal. calc. for $C_{25}H_{31}BiF_3N_3O_3S$ (717.56 g/mol): C, 41.73; H, 4.34; N, 5.84; S, 4.46; found: C, 41.68; H, 4.52; N, 6.01; S, 4.49.

**Synthesis of 1,5-hexadiene insertion product - [Bi(N($C_3H_7$)($C_6H_4$)(CH($C_4H_7$)$CH_2$))(OTf)($NC_5H_5$)$_2$] (5-*i*Pr)**

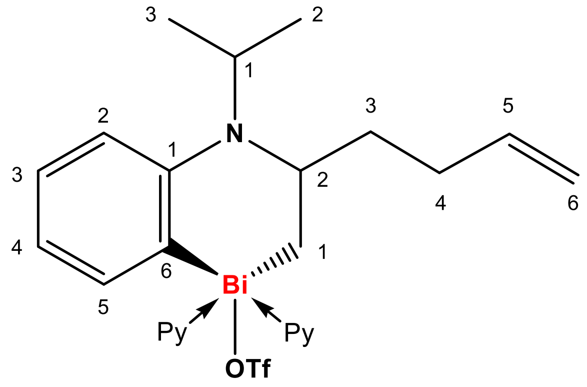


[$Bi_2$($NC_3H_7$($C_6H_4$))$_2$(OTf)$_2$(thf)$_3$] (**1-*i*Pr**) (60 mg, 0.071 mmol) was dissolved in pyridine (3 mL) and an excess of 1,5-hexadiene (0.6 mL) was added. The reaction mixture was then heated to 60 °C for 18 hours. The reaction mixture had changed color from red to yellow. All volatiles were removed in vacuo and the remaining residue was dissolved in pyridine (1 mL). The solution was filtered and layered with $Et_2O$ (1 mL) for crystallization. After 3 days yellow block-like crystals had formed from the solution and were characterized using SC-XRD. The crystals were isolated by filtration, washed with $Et_2O$ (2 × 2 mL), and dried under vacuum for 2 hours. Compound **5-*i*Pr** was obtained as a light-yellow powder.

Yield = 30.5 mg, (0.041 mmol, 45%).

**$^1$H NMR** (298 K, 500 MHz, Pyridine-*d₅*): $\delta$ = 0.51 (d, 3H, $^3J_{HH}$= 6.5 Hz, 2-$CH_3$-iPr), 1.06-1.13 (m, 1H, 3-$CH_2$), 1.15 (d, 3H, $^3J_{HH}$ = 6.5 Hz, 3-$CH_3$-iPr), 1.18-1.28 (m, 1H, 3-$CH_2$), 1.50-1.59 (m, 1H, 4-CH2), 1.64-1.73 (m, 1H, 4-CH3), 2.63 (dd, 1H, , $^2J_{HH}$ = 13.0 Hz, $^3J_{HH}$ = 1.8 Hz, 1-$CH_2$(a)), 2.70 (dd, 1H, , $^2J_{HH}$ = 13.1 Hz, $^3J_{HH}$ = 5.6 Hz, 1-$CH_2$(b)), 3.55 (sept, 1H, $^3J_{HH}$ = 6.5 Hz, 1-CH-iPr), 4.54 (dd, 1H, $^2J_{HH}$ = 17.1 Hz, $^3J_{HH}$ = 1.7 Hz, 6-CH2(a)), 4.66 (dd, 1H, $^2J_{HH}$ = 10.2 Hz, $^3J_{HH}$ = 1.3 Hz, 6-CH2(b)), 5.24 (m, 1H, 5-CH2), 5.64-5.72 (m, 1H, 2-CH), 7.13-7.18 (m, 1H, 4-$C_6H_4$), 7.18-7.20 (m, 3,5-pyridine), 7.47 (td, 1H, $^3J_{HH}$ = 7.8 Hz, $^4J_{HH}$ = 1.7 Hz, 3-$C_6H_4$), 7.55 (m, 4-pyridine), 7.72 (d, 1H, $^3J_{HH}$ = 8.3 Hz, 2-$C_6H_4$), 8.50 (dd, $^3J_{HH}$ = 7.1Hz, $^4J_{HH}$ = 1.6Hz, 5-$C_6H_4$), 8.70 (m, 2,6-pyridine) ppm.

**$^{13}$C NMR** (298 K, 125 MHz, Pyridine-*d₅*): $\delta$ = 20.16 (s, 3-$CH_3$-iPr), 21.55 (s, 2-$CH_3$-iPr), 31.35 (s, 4-$CH_3$), 35.13 (s, 3-$CH_3$), 47.51 (s, 2-CH), 51.45 (s, 1-CH-iPr), 67.05 (s, 1-$CH_2$), 118.45 (q, $^1J_{CF}$ = 321 Hz, OTf), 114.71 (s, 6-$CH_2$), 119.10 (s, 2-$C_6H_4$), 124.06 (s, 4-$C_6H_4$), 132.90 (s, 3-$C_6H_4$), 137.85 (s, 5-CH), 139.62 (s, 5-$C_6H_4$), 153.37 (s, 1-$C_6H_4$), 171.55 (s, 6-$C_6H_4$) ppm.

**$^{19}$F NMR** (298 K, 282 MHz, Pyridine-*d₅*): $\delta$ = –77.26 (s, OTf) ppm.

**Elemental analysis.** Anal. calc. for $C_{26}H_{31}BiF_3N_3O_3S$ (729.57 g/mol): C, 42.69; H, 4.27; N, 5.74; S, 4.38; found: C, 42.30; H, 4.36; N, 5.73; S, 4.24.

**Synthesis of styrene insertion product - [Bi(N($C_3H_7$)($C_6H_4$)(CH($C_6H_5$)$CH_2$))(OTf)($NC_5H_5$)$_2$] (6-*i*Pr)**

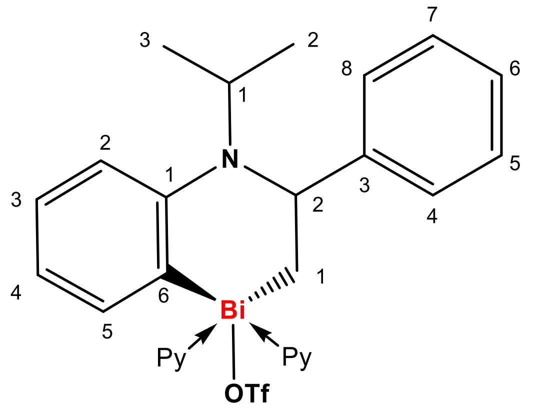


[$Bi_2$($NC_3H_7$($C_6H_4$))$_2$(OTf)$_2$(thf)$_3$] (**1-*i*Pr**) (60 mg, 0.071 mmol, 1.00eq.) was dissolved in pyridine (1.5 mL) and an excess of styrene (0.6 mL) was added. The reaction mixture was then heated to 60 °C for 18 hours. The reaction mixture had changed color from red to yellow. All volatiles were removed in vacuo and the remaining oily residue was dissolved in pyridine (1 mL) and layered with $Et_2O$ (2 mL) for crystallization. After one day, a white polycrystalline powder had precipitated from the solution, which was isolated by filtration, washed with $Et_2O$ (2 × 2 mL), and dried under vacuum for 2 hours. Compound **6-*i*Pr** was obtained as a white powder.

Yield = 42 mg (0.056 mmol, 39 %).

**$^1$H NMR** (298 K, 500 MHz, Pyridine-*d*$_5$): $\delta$ = 0.67 (d, 3H, $^3J_{HH}$= 6.7 Hz, 2-$CH_3$-*i*Pr), 1.09 (d, 3H, $^3J_{HH}$ = 6.6 Hz, 3-$CH_3$-*i*Pr), 2.90 (dd, 1H, $^2J_{HH}$ = 13.2 Hz, $^3J_{HH}$ = 6.3 Hz, 1-$CH_2$(a)-styrene), 3.31 (dd, 1H , $^2J_{HH}$ = 13.1 Hz, $^3J_{HH}$ = 2.3 Hz, 1-$CH_2$(b)-styrene), 3.93 (sept, 1H, $^3J_{HH}$ = 6.6 Hz, 1-CH-*i*Pr), 6.68-6.78 (m, 3H, 2,4,6-$C_6H_5$-styrene), 7.03-7.05 (m, 2H, 3,5-$C_6H_5$-styrene), 7.07 (t, 1H, $^3J_{HH}$ = 6.8 Hz, 4-$C_6H_4$), 7.18 (m, 3,5-pyridine), 7.45-7.46 (m, 1H, 2-CH-styrene), 7.55 (m, 4-pyridine,3-$C_6H_4$), 7.96 (d, 1H, $^3J_{HH}$ = 8.5 Hz, 2-$C_6H_4$), 8.23 (dd, $^3J_{HH}$ = 7.1Hz, $^4J_{HH}$ = 1.7 Hz, 5-$C_6H_4$), 8.70 (m, 2,6-pyridine) ppm.

**$^{13}$C NMR** (298 K, 125 MHz, Pyridine-*d*$_5$): $\delta$ = 20.16 (s, 3-$CH_3$-iPr), 21.68 (s, 2-$CH_3$-iPr), 49.72 (s, 2-CH-styrene), 52.19 (s, 1-CH-iPr), 65.27 (s, 1-$CH_2$-styrene), 118.28 (s, 2-$C_6H_4$), 118.45 (q, $^1J_{CF}$ = 321 Hz, OTf), 119.95 (s, 4-$C_6H_4$), 123.80 (s, pyridine), 126.07 (s, 4-$C_6H_5$-styrene), 127.64 (s, 3,5-$C_6H_5$-styrene), 128.31 (s, 2,6- $C_6H_5$-styrene), 133.10 (s, 3-$C_6H_4$), 135.76 (s, pyridine), 140.09 (s, 5-$C_6H_4$), 143.45 (s, 1- $C_6H_5$-styrene), 153.80 (s, 1-$C_6H_4$), 167.27 (s, 6-$C_6H_4$) ppm.

**ESI-MS** (positive mode, THF): found m/z: 446.132, calculated for $BiNC_{17}H_{19}^+$ [M-($O_3SCF_3^-$ + 2 $C_5H_5N$)]$^+$ m/z: 446.132.

**NMR Spectra**

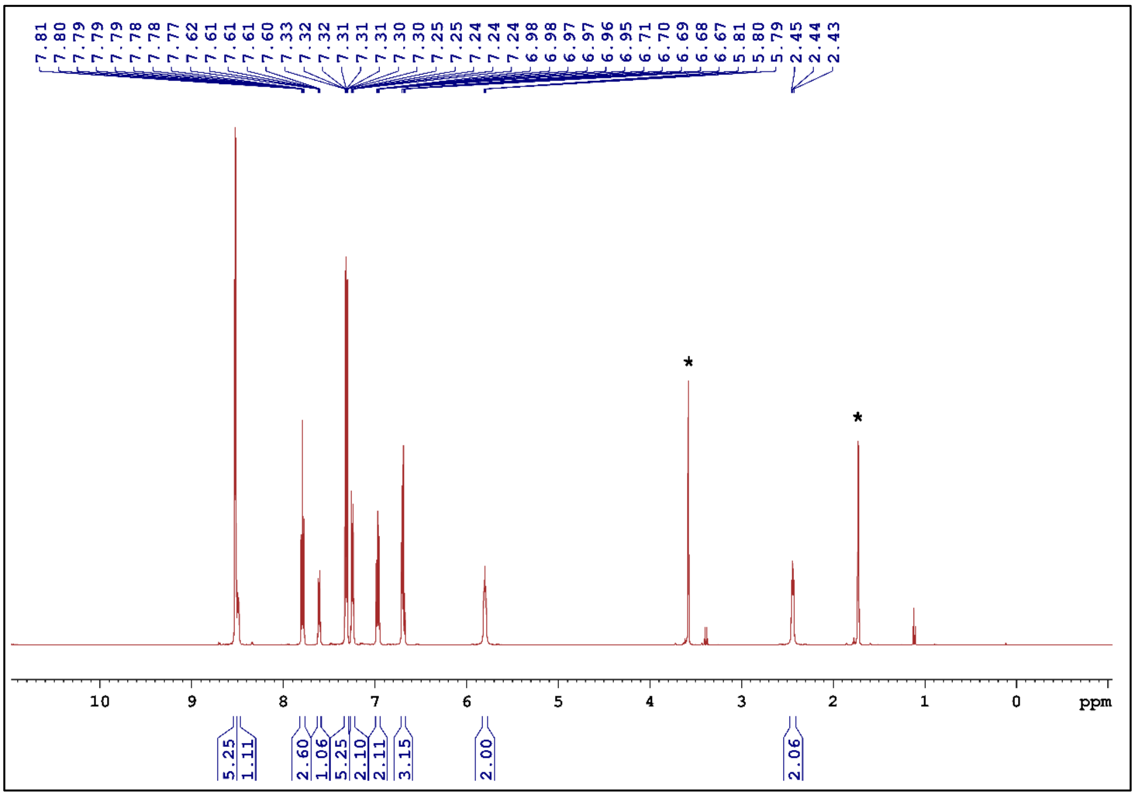


**Figure S1**: $^{1}H$ NMR spectrum of **2-Ph** in THF-$d_8$ (solvent signals: *). Traces of diethyl ether were detected.

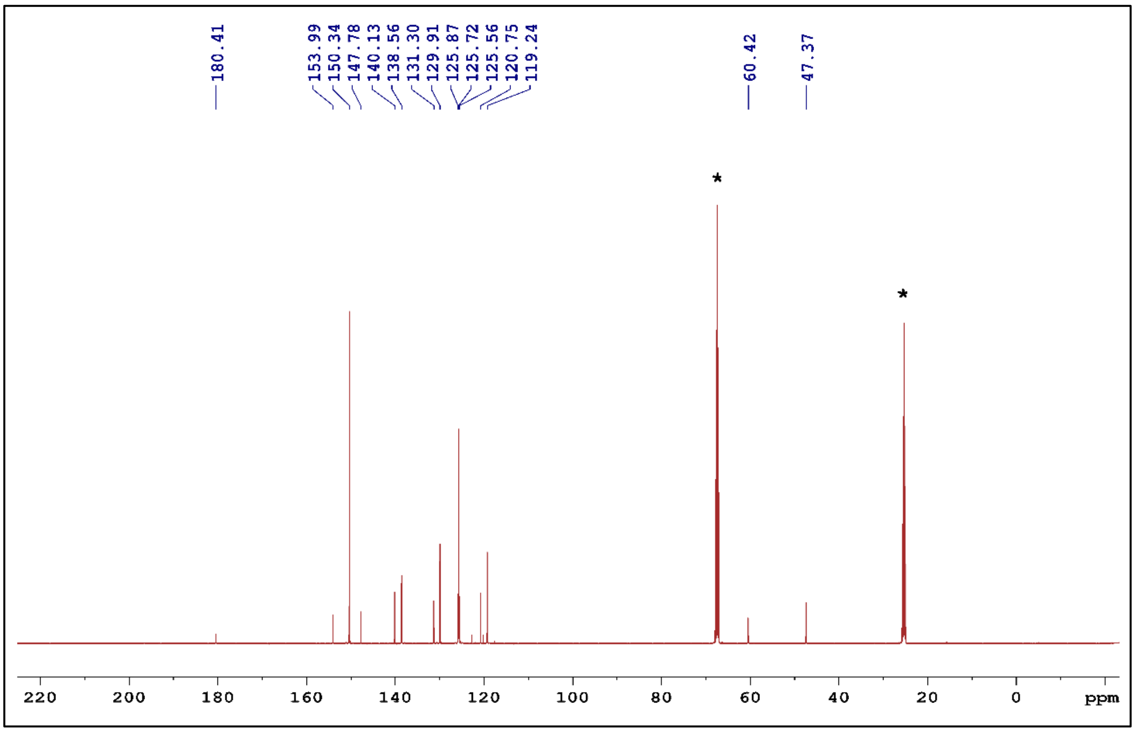


**Figure S2**: $^{13}C$ NMR spectrum of **2-Ph** in THF-$d_8$ (solvent signals: *).

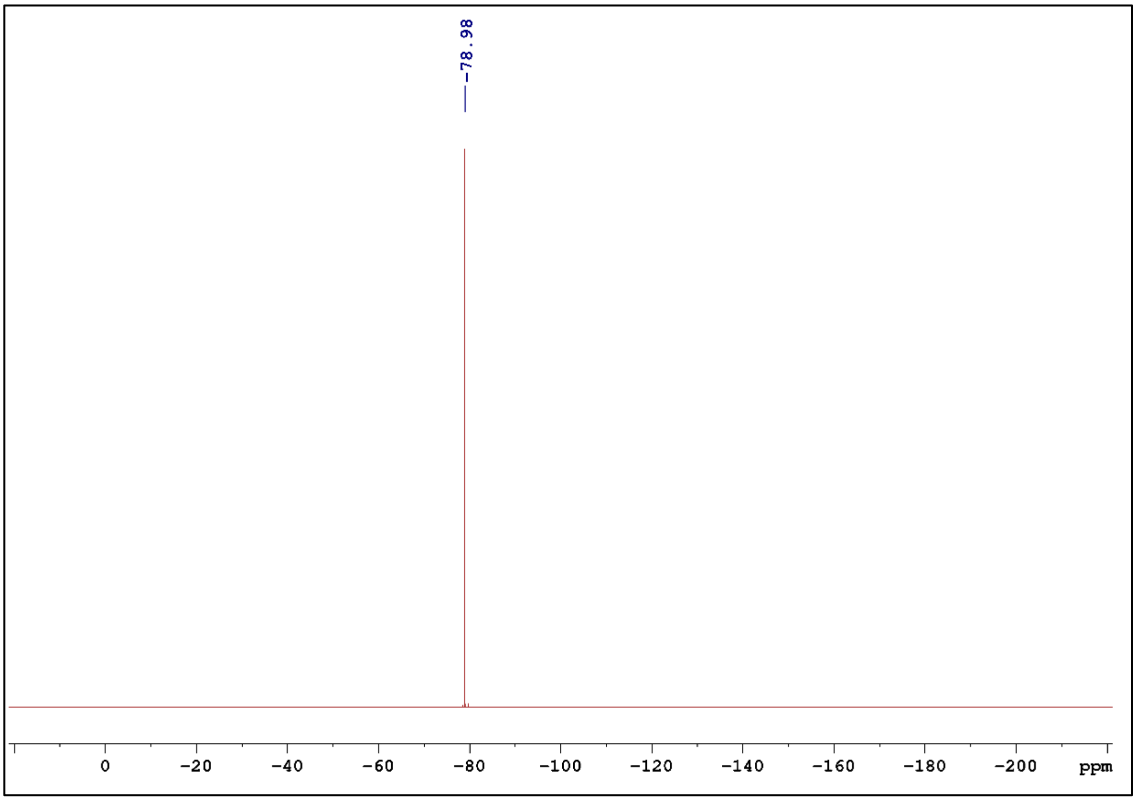


**Figure S3**: $^{19}$F NMR spectrum of **2-Ph** in THF-$d_8$.

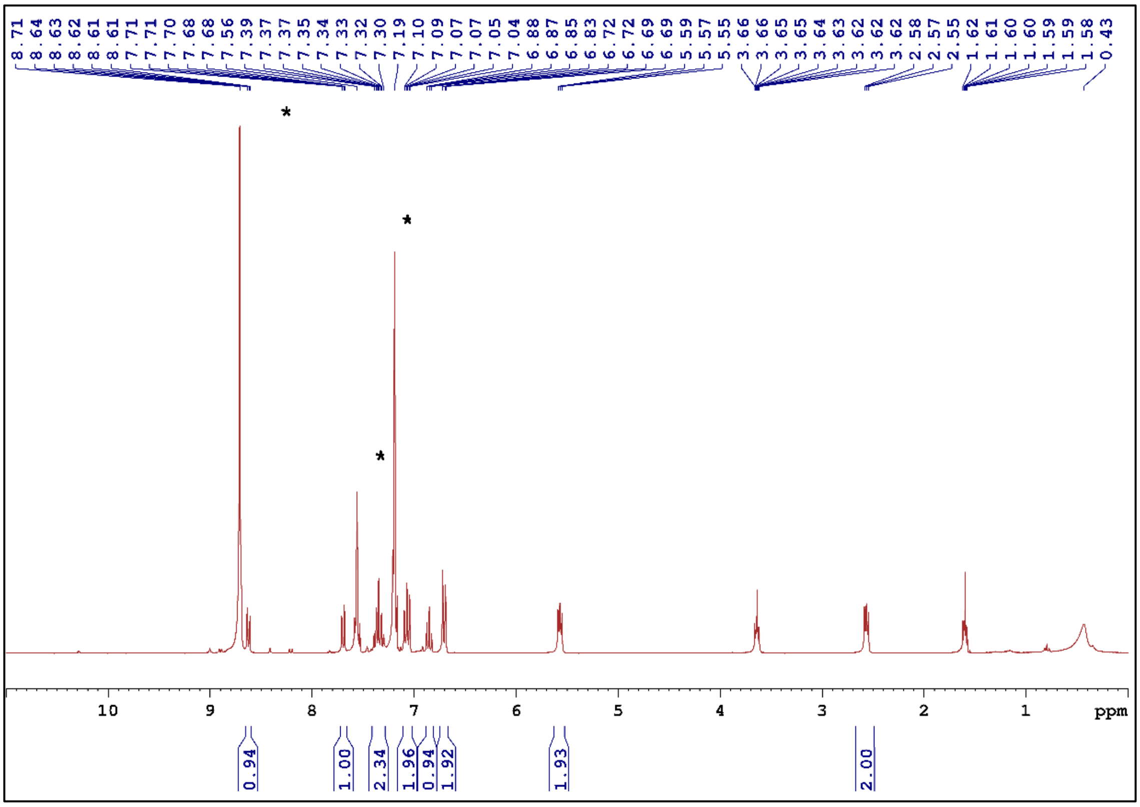


**Figure S4**: $^{1}$H NMR spectrum of **2-Ph** in pyridine-$d_5$ (solvent signals: *). Traces of toluene were detected. Peak near 0 ppm corresponds to impurity from perfluorinated oil used for XRD analysis.

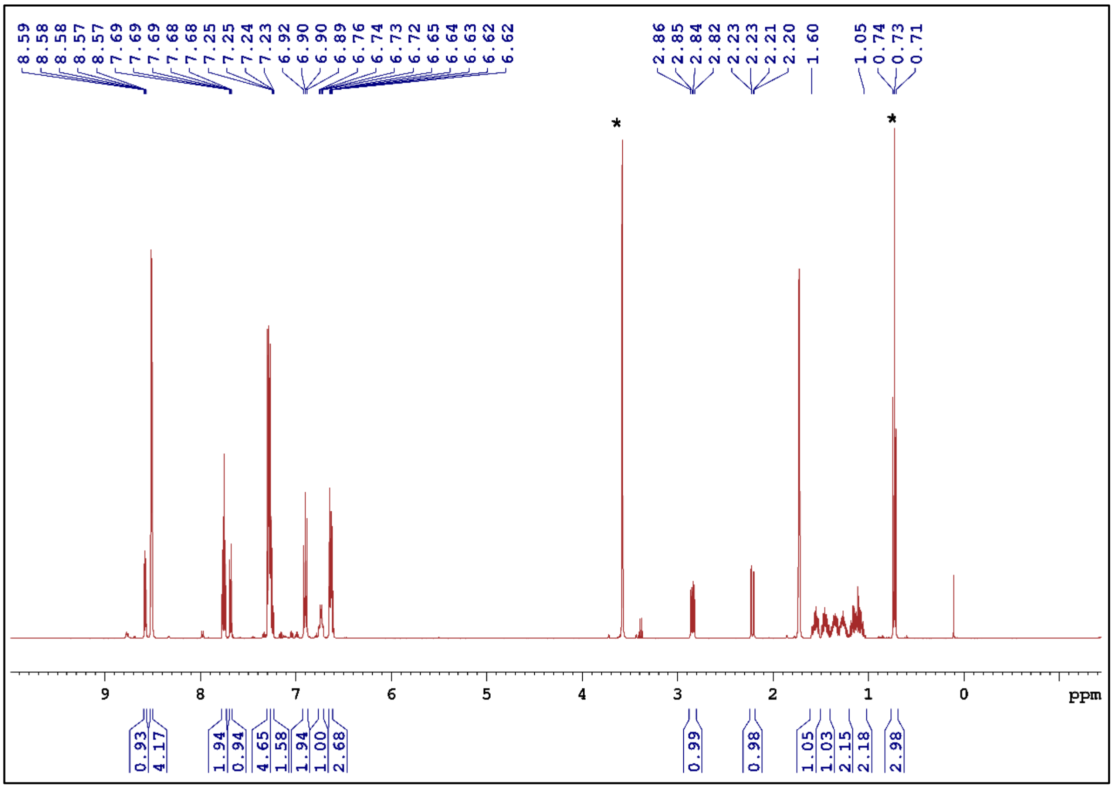


**Figure S5 (A)**: $^{1}$H NMR spectrum of **3-Ph** in THF-$d_8$ (solvent signals: *). Traces of diethyl ether were detected. Peak picking is not shown for the signals corresponding to pyridine and resonances between 1.05-1.60 ppm for clarity.

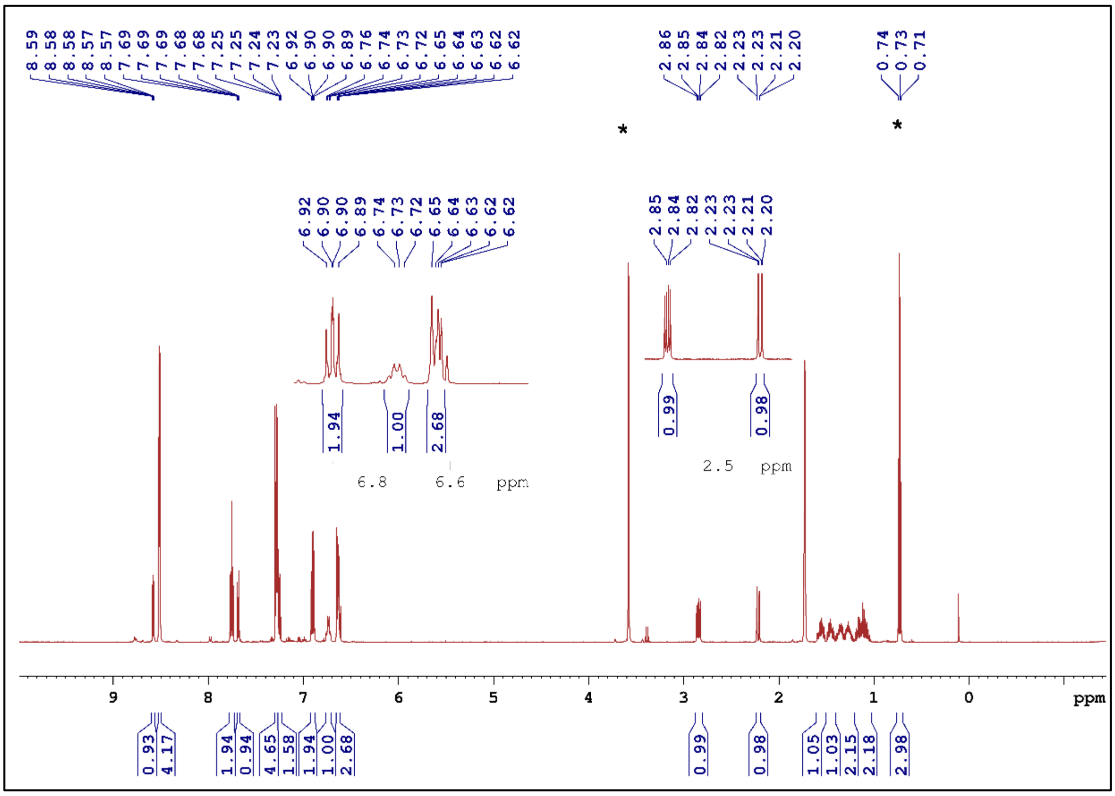


**Figure S5 (B)**: $^{1}$H NMR spectrum of **3-Ph** in THF-$d_8$ (solvent signals: *), with inlets showing zoom-in on signals for 1-$CH_2$ and 2-CH protons. Peak picking is not shown for the signals corresponding to pyridine and resonances between 1.05-1.60 ppm for clarity.

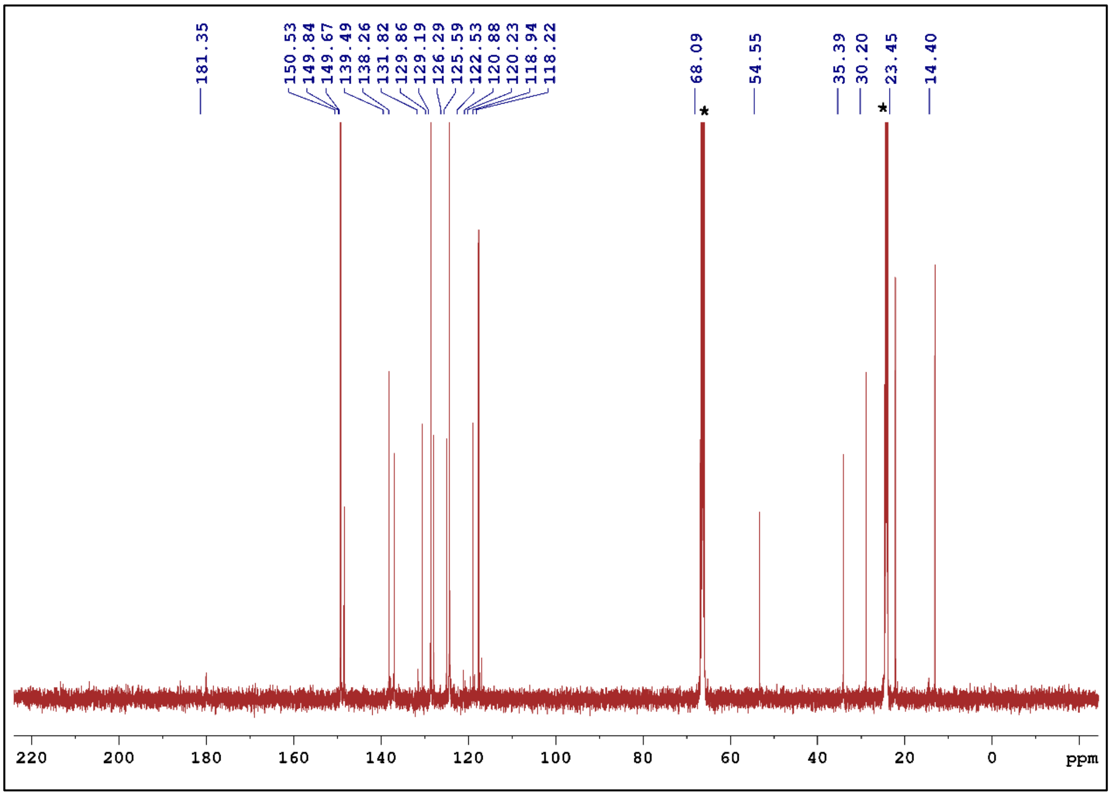


**Figure S6**: $^{13}$C NMR spectrum of **3-Ph** in THF-$d_8$ (solvent signals: *).

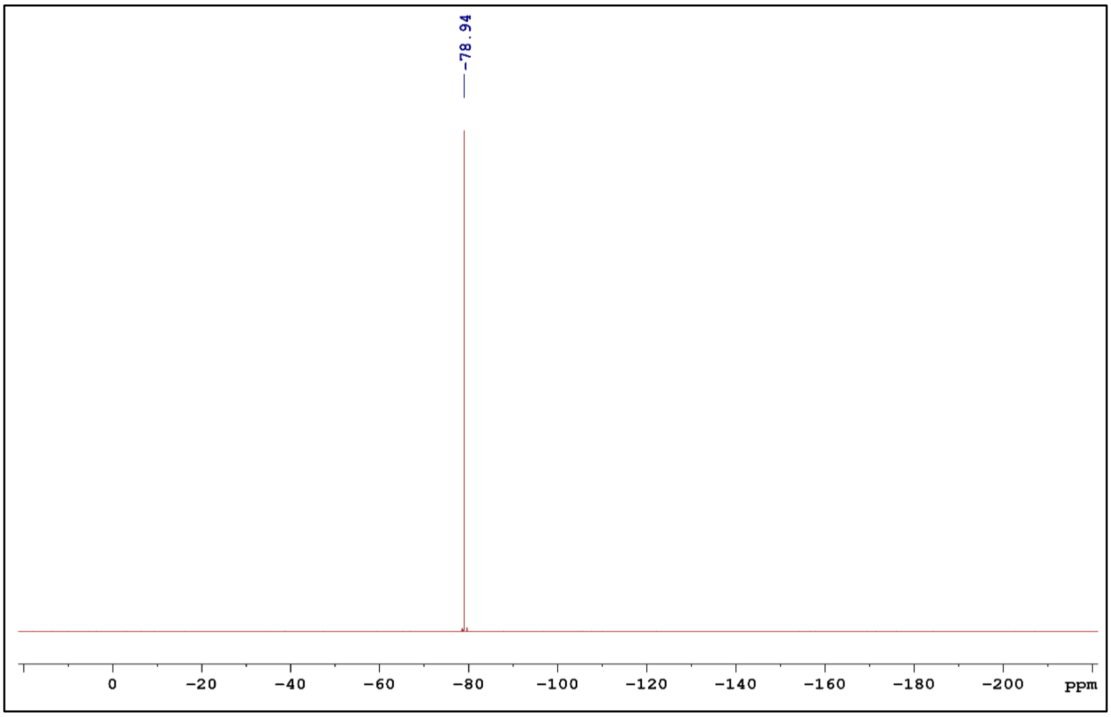


**Figure S7**: $^{19}$F NMR spectrum of **3-Ph** in THF-$d_8$.

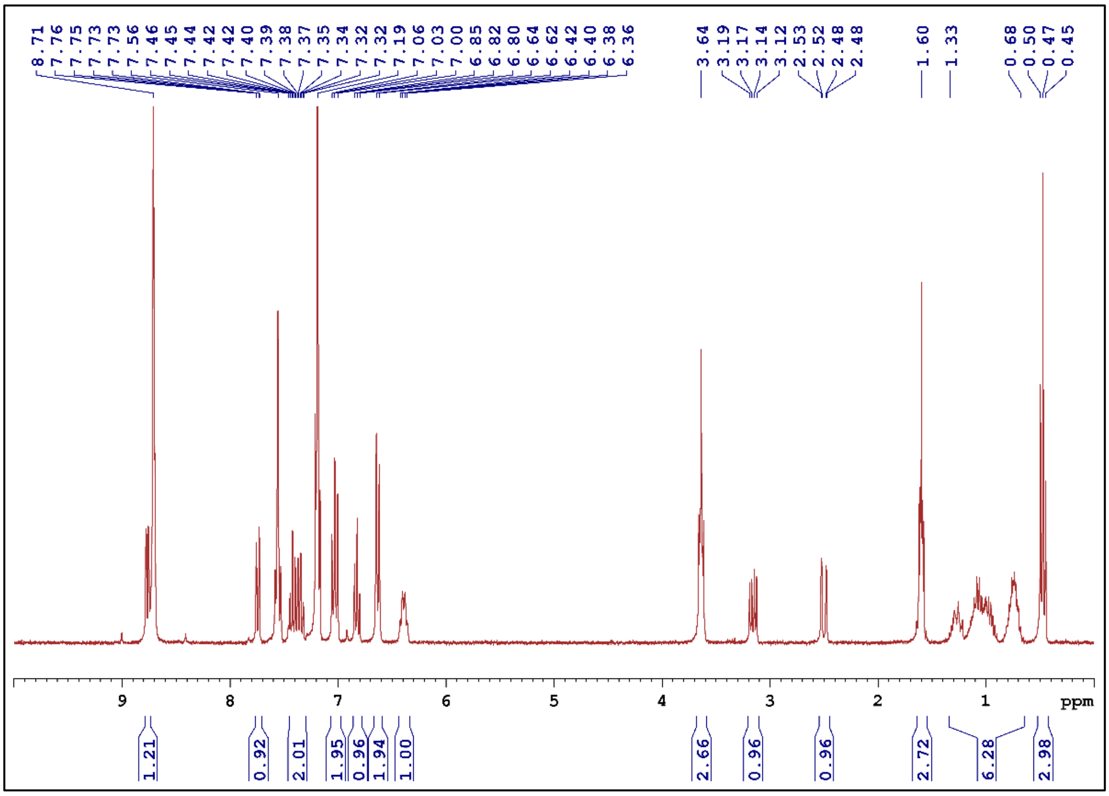


**Figure S8**: $^{1}$H NMR spectrum of **3-Ph** in pyridine-$d_5$ (solvent signals: *). Peak picking is not shown for the signals corresponding to pyridine and the resonances between 0.68-1.33 ppm for clarity.

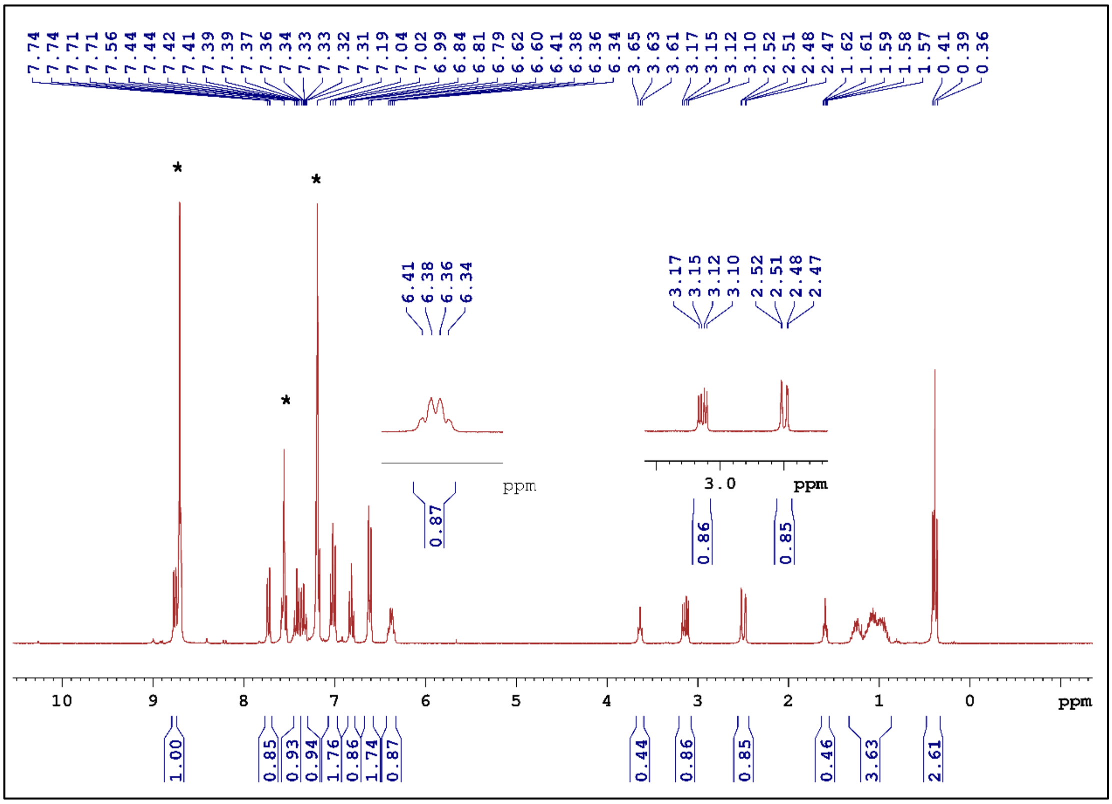


**Figure S9**: $^{1}$H NMR spectrum of **4-Ph** in pyridine-$d_5$ (solvent signals: *). Peak picking is not shown for the signals corresponding to pyridine and the resonances between 0.88-1.31 ppm for clarity. Traces of THF were detected.

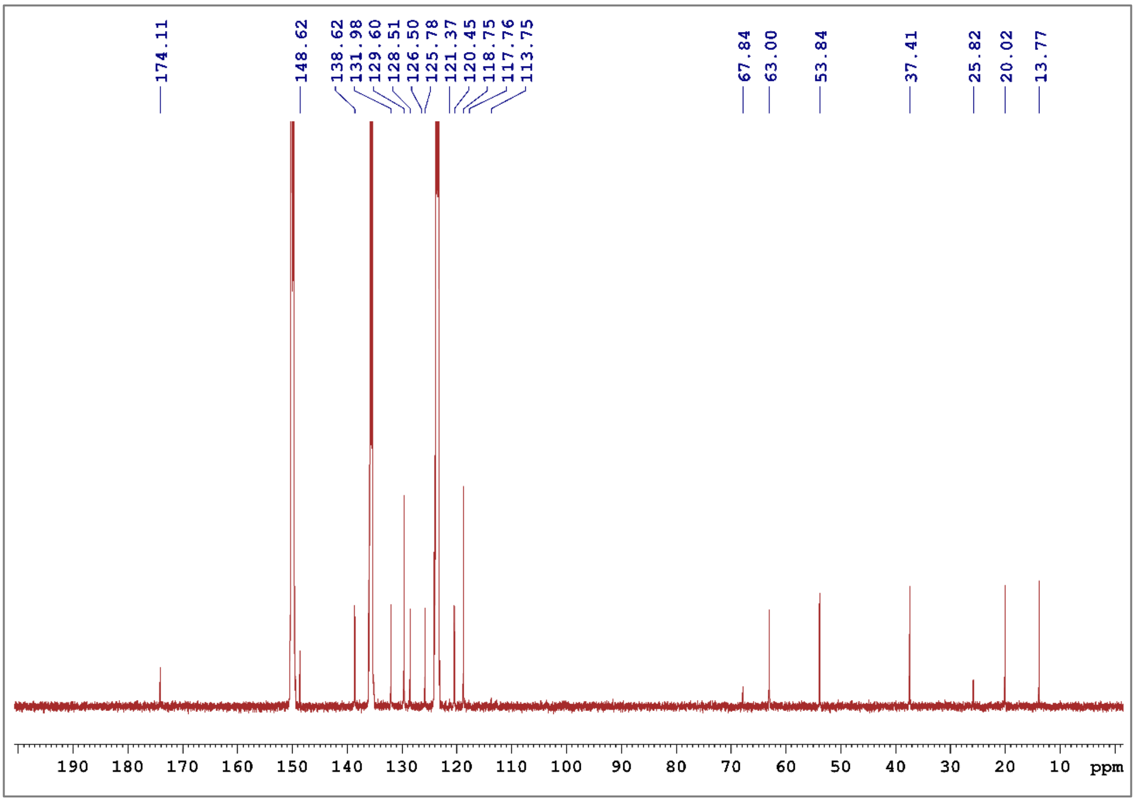


**Figure S10**: $^{13}$C NMR spectrum of **4-Ph** in pyridine-$d_5$ (solvent signals: *).

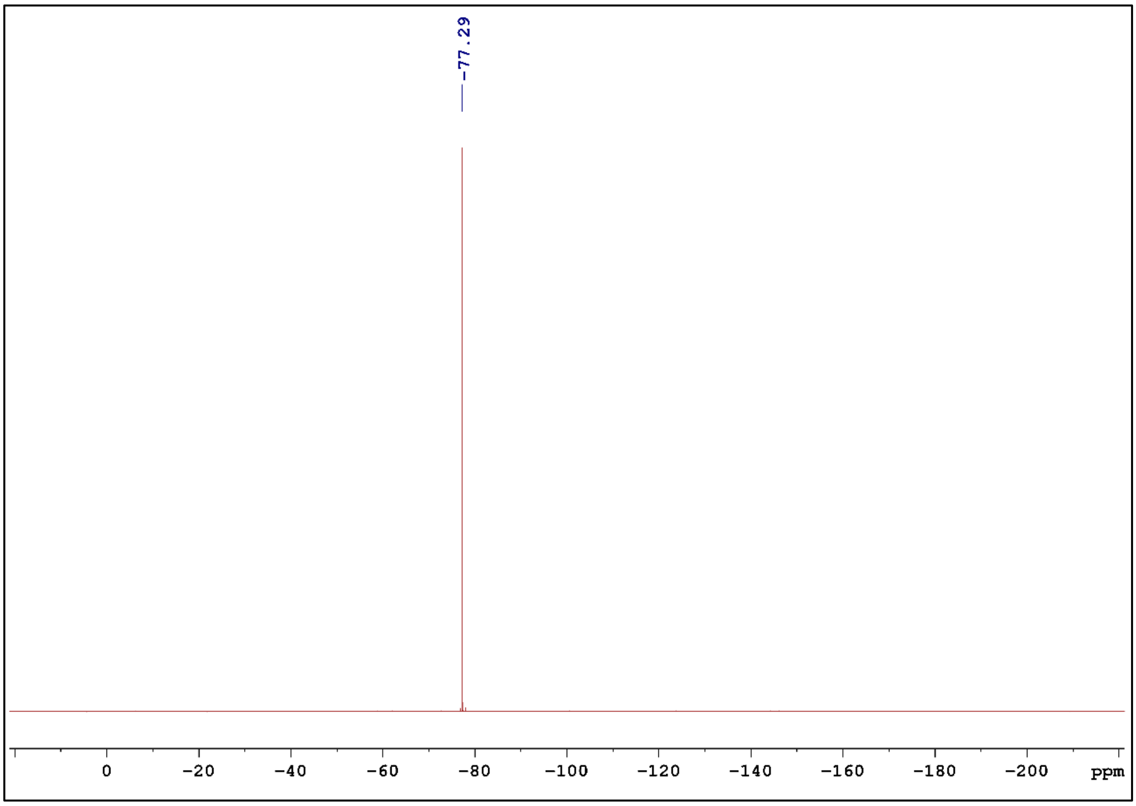


**Figure S11**: $^{19}$F NMR spectrum of **4-Ph** in pyridine-$d_5$.

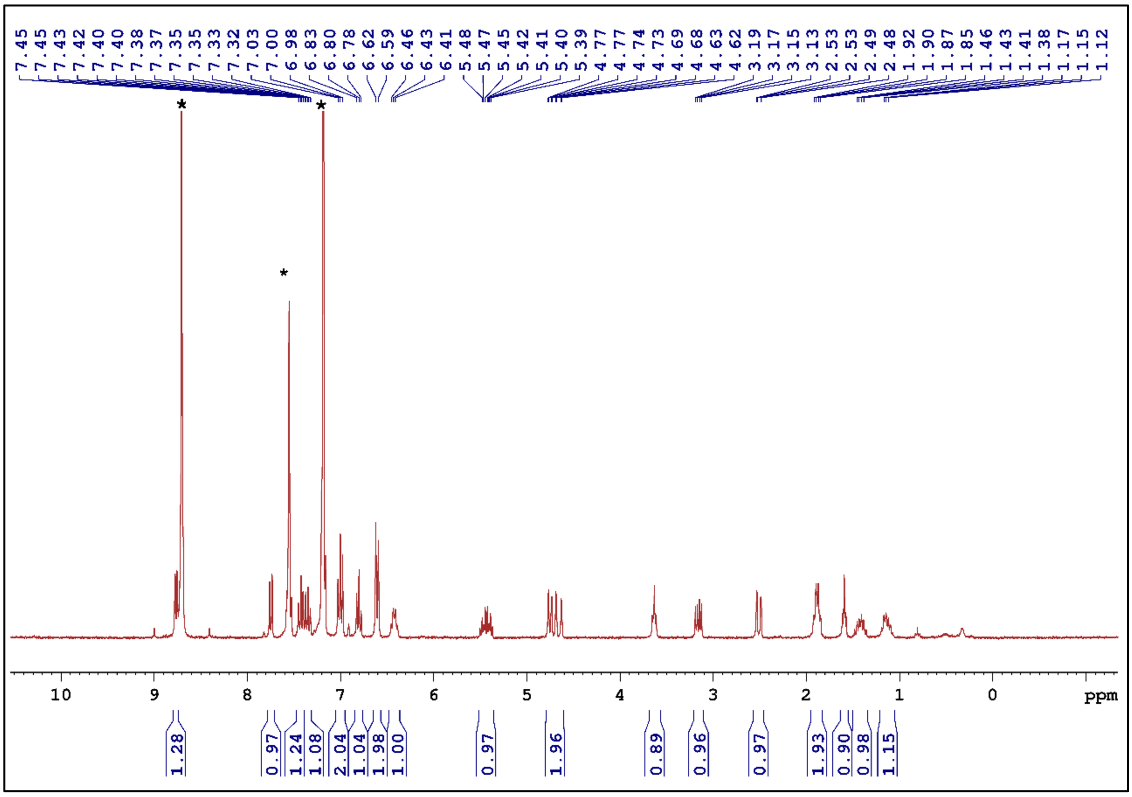


**Figure S12**: $^{1}$H NMR spectrum of **5-Ph** in pyridine-$d_5$ (solvent signals: *). Traces of THF and of pentane found.

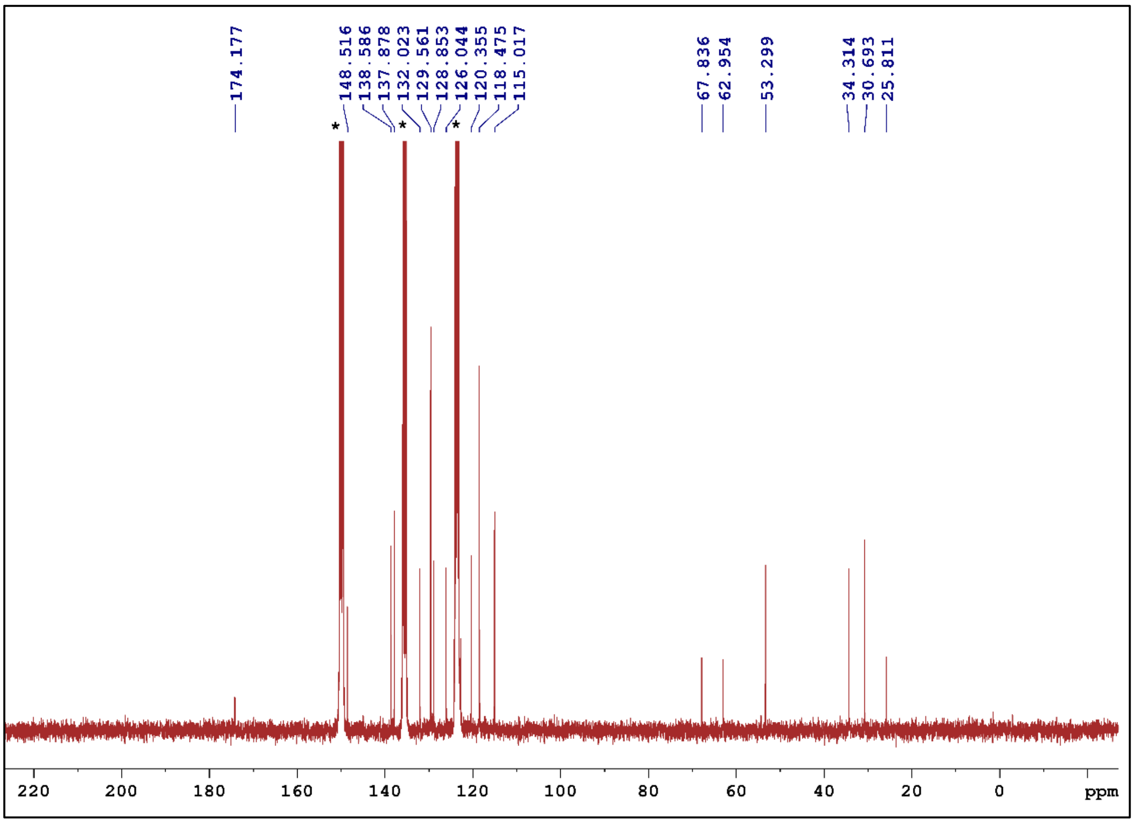


**Figure S13**: $^{13}$C NMR spectrum of **5-Ph** in pyridine-$d_5$ (solvent signals: *).

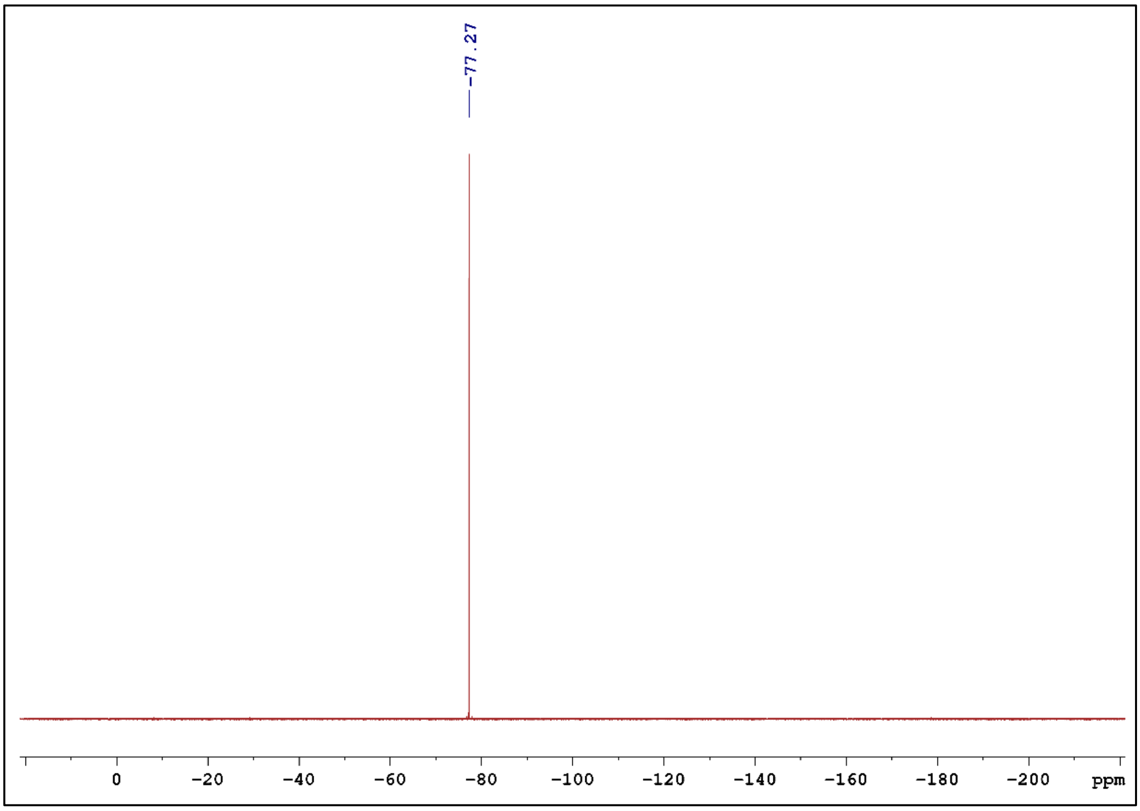


**Figure S14**: $^{19}$F NMR spectrum of **5-Ph** in pyridine-$d_5$.

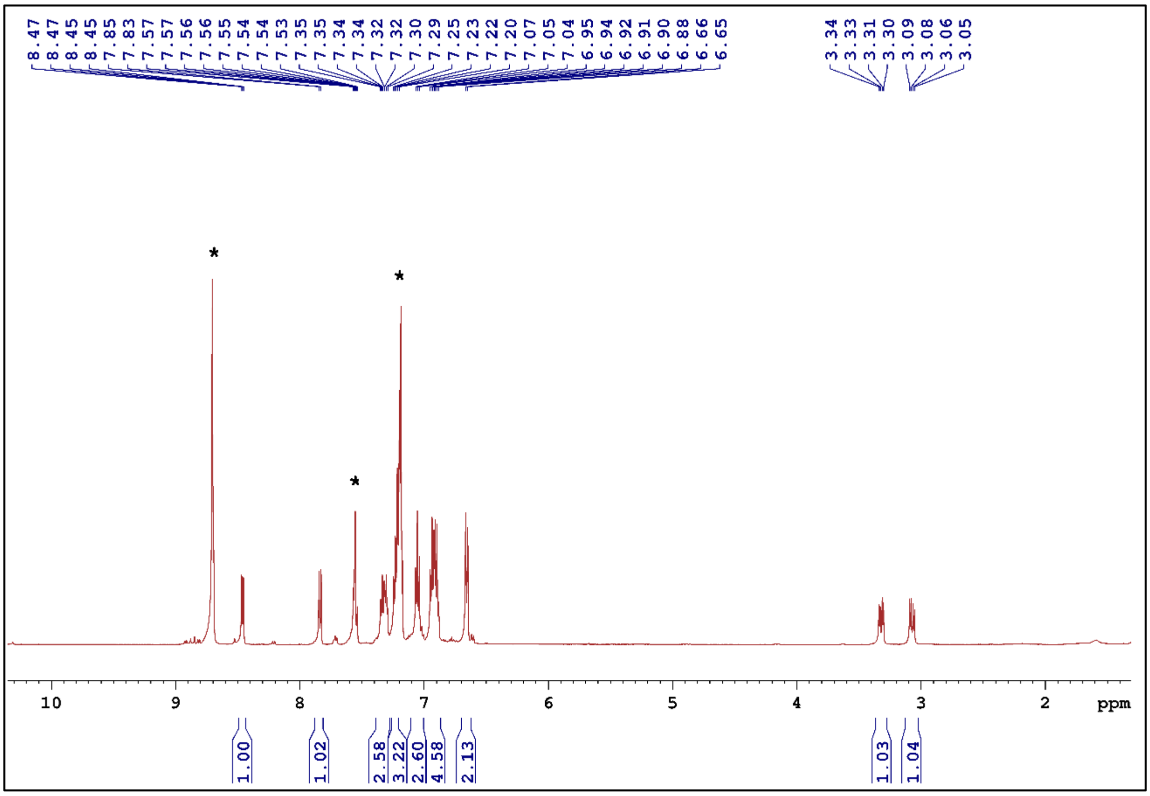


**Figure S15**: $^{1}$H NMR spectrum of **6-Ph** in pyridine-$d_5$ (solvent signals: *). Peak picking is not shown for the signals corresponding to pyridine for clarity.

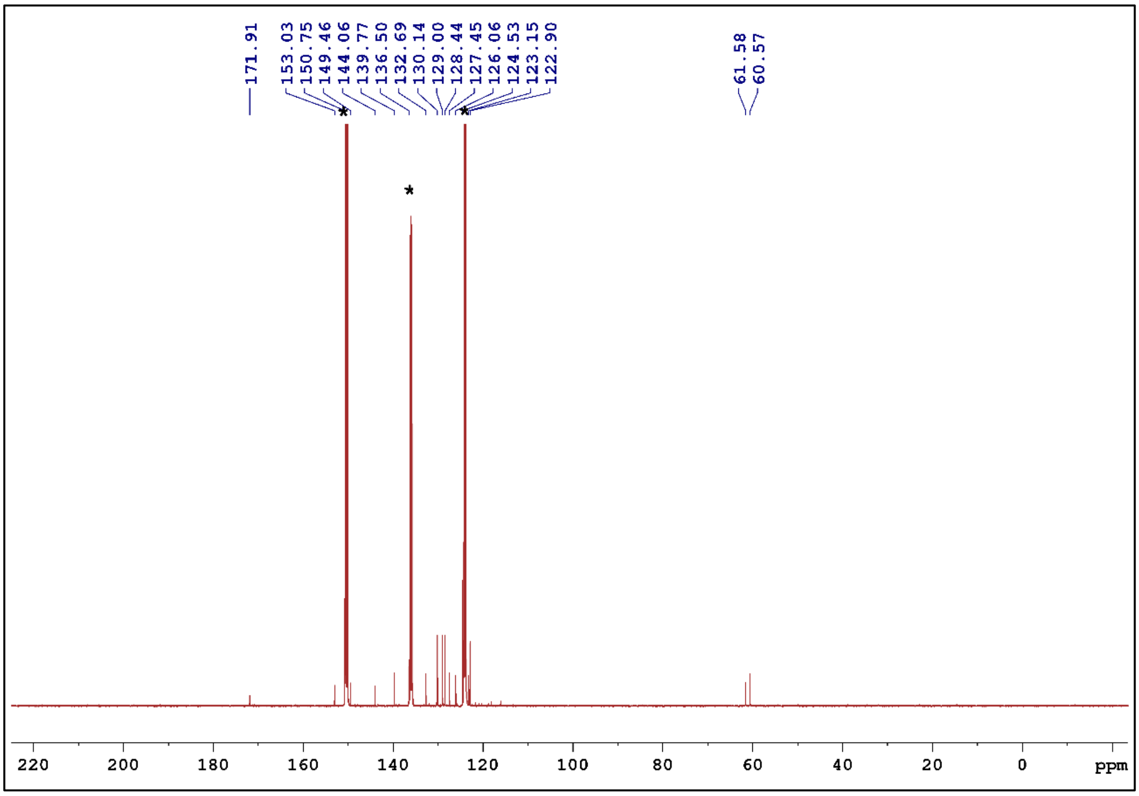


**Figure S16**: $^{13}$C NMR spectrum of **6-Ph** in pyridine-*d*$_5$ (solvent signals: *).

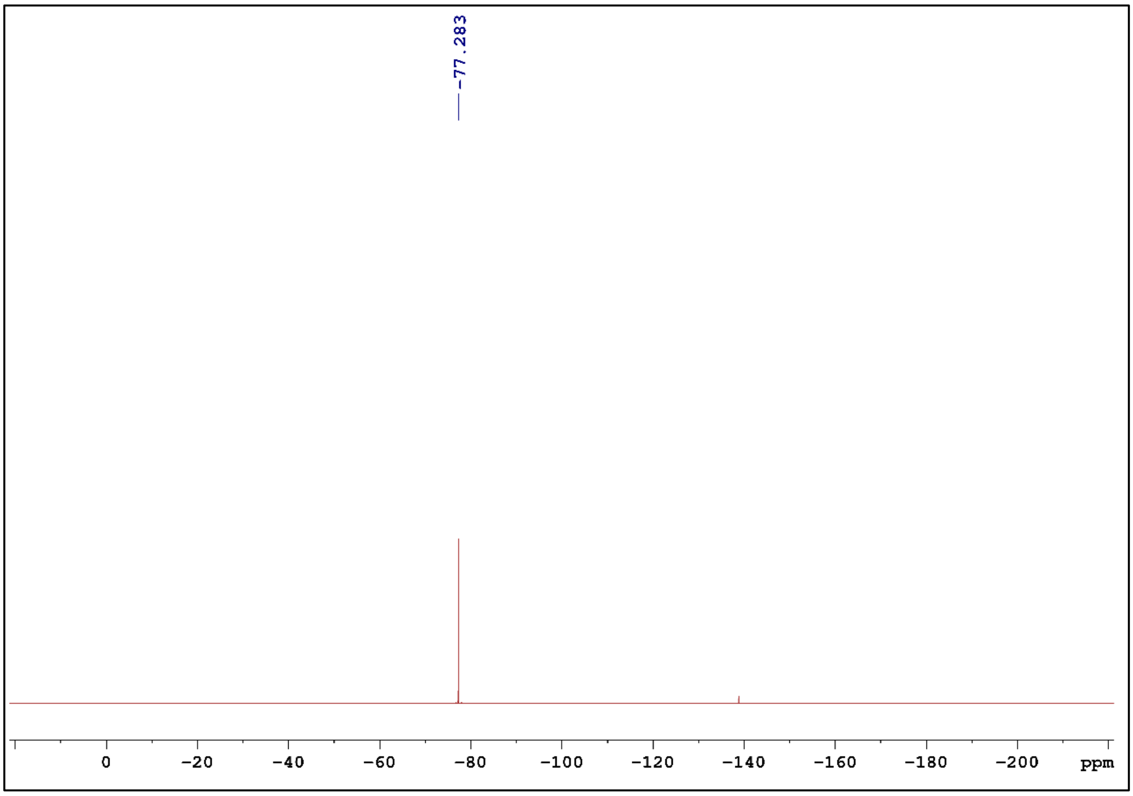


**Figure S17**: $^{19}$F NMR spectrum of **6-Ph** in pyridine-*d*$_5$.

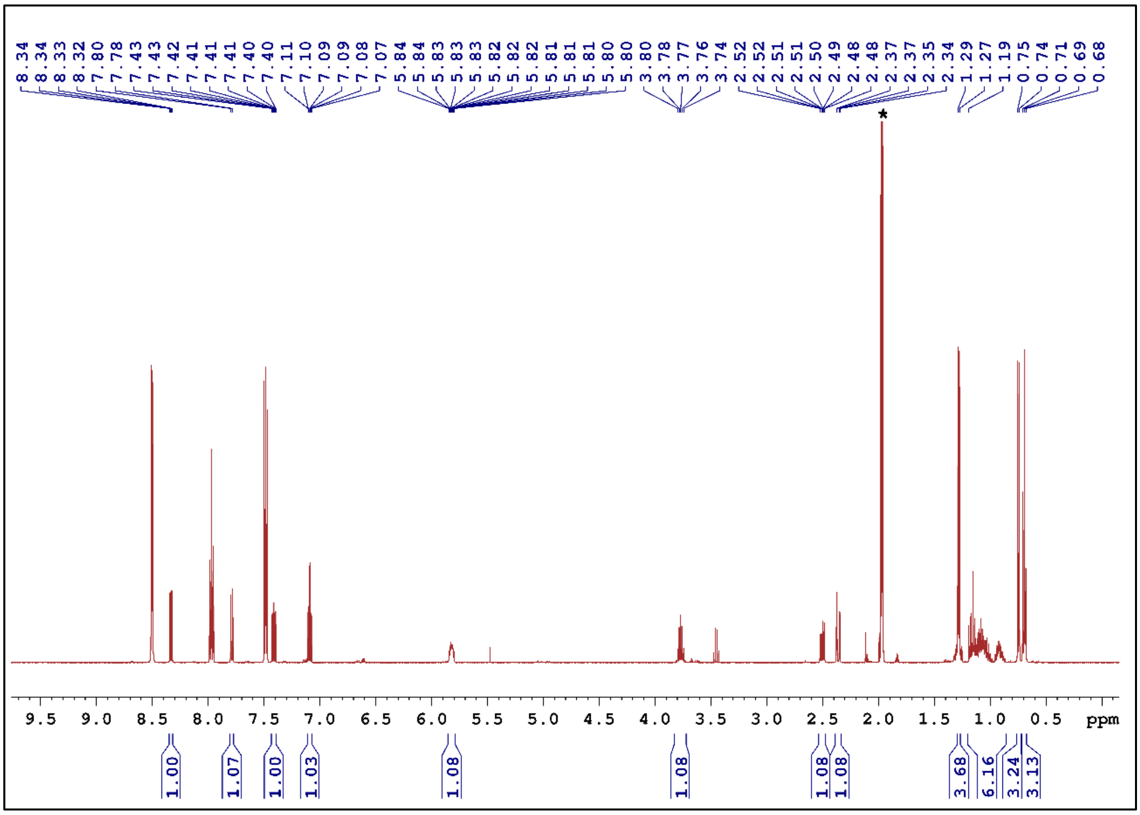


**Figure S18:** $^{1}$H NMR spectrum of **3-*i*Pr** in acetonitrile-$d_3$. (solvent signals: *). Peak picking is not shown for the signals corresponding to pyridine and the resonances between 0.86-1.19 ppm for clarity. Traces of diethyl ether were detected.

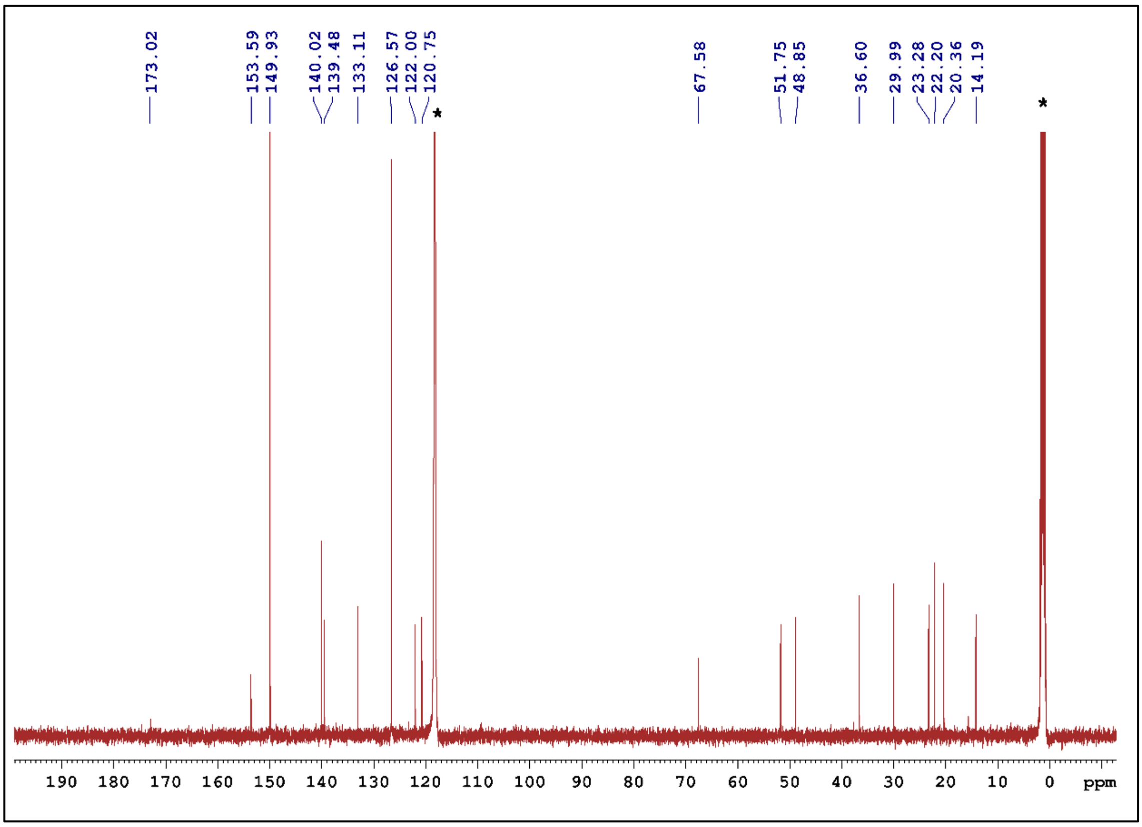


**Figure S19**: $^{13}$C NMR spectrum of **3-*i*Pr** in acetonitrile-$d_3$ (solvent signals: *).

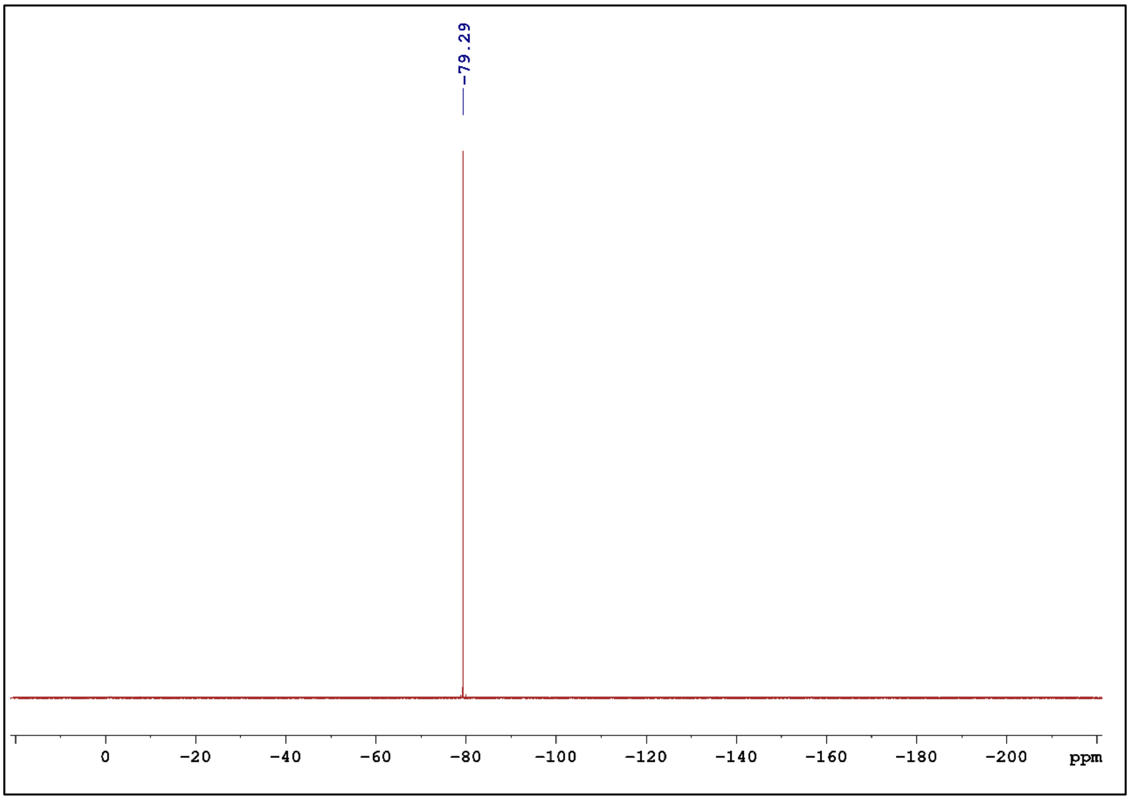


**Figure S20**: $^{19}$F NMR spectrum of **3-*i*Pr** in acetonitrile-$d_3$.

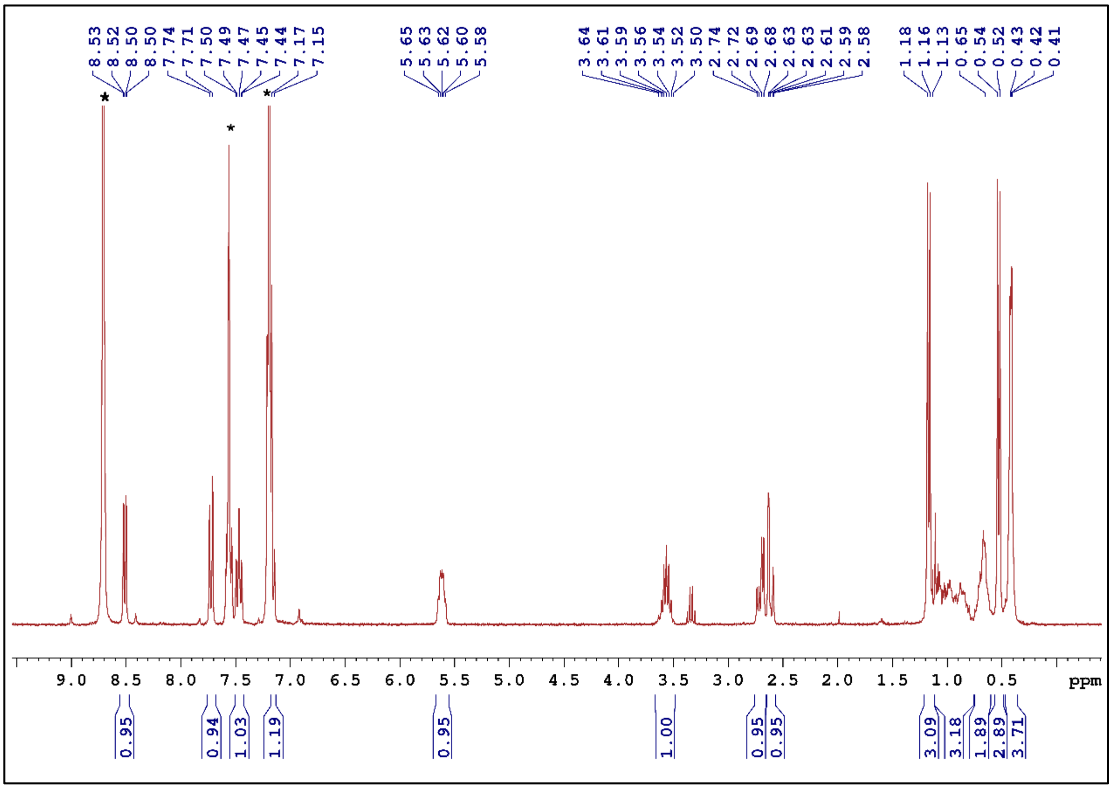


**Figure S21**: $^{1}$H NMR spectrum of **3-*i*Pr** in pyridine-$d_5$ (solvent signals: *). Peak picking is not shown for the signals corresponding to pyridine and the resonances between 0.65-1.13 ppm are clarity. Traces of diethyl ether were detected.

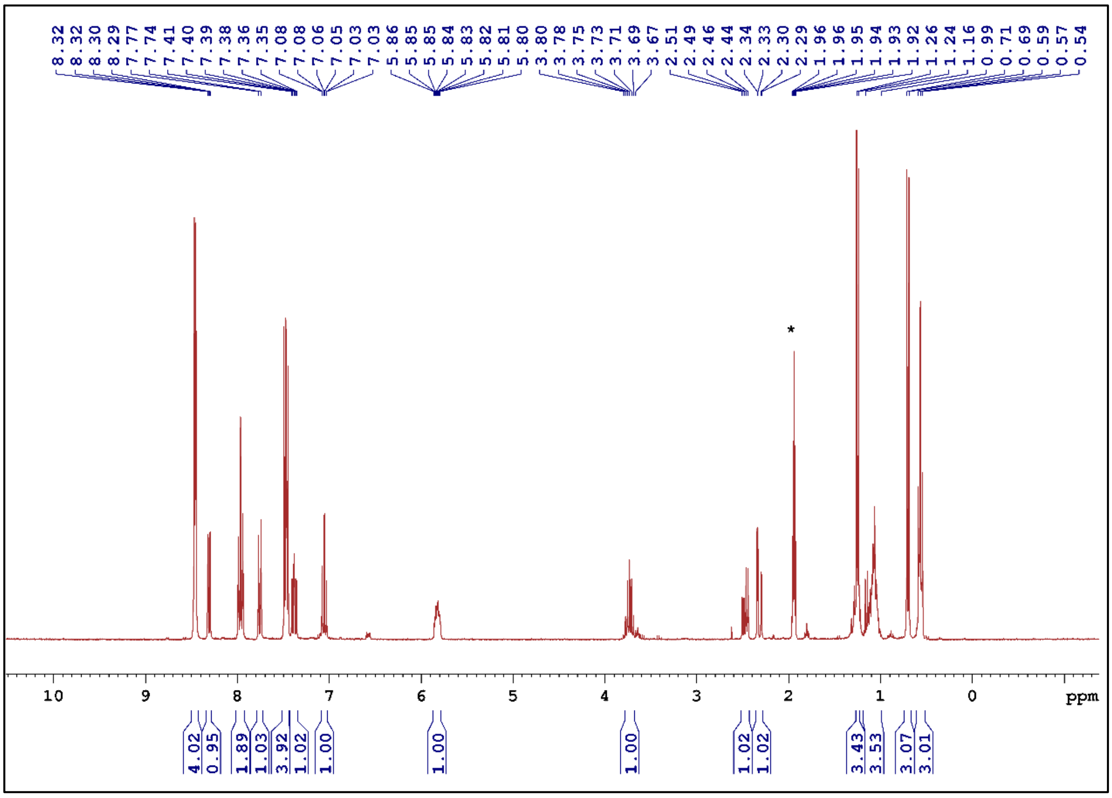


**Figure S22:** $^{1}$H NMR spectrum of **4-*i*Pr** in acetonitrile-$d_3$. (Solvent signals: *). Peak picking is not shown for the signals corresponding to pyridine and the resonances between 0.99-1.16 ppm for clarity. Traces of THF were detected.

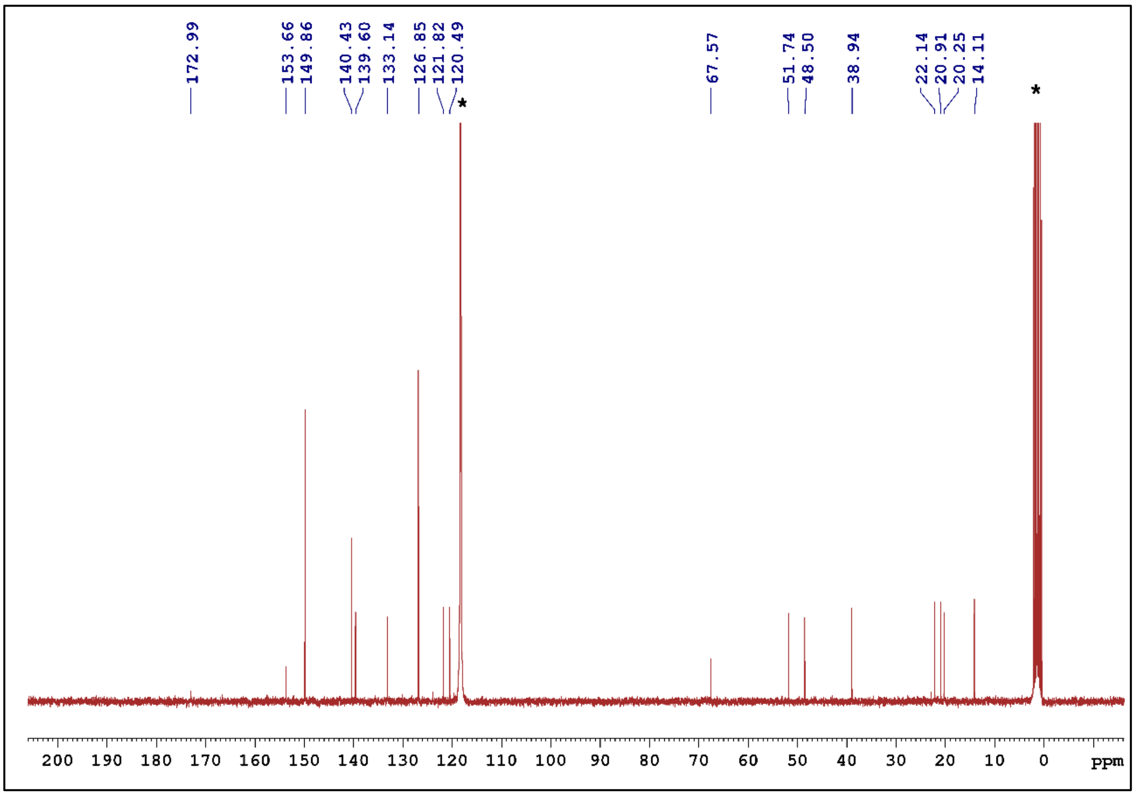


**Figure S23**: $^{13}$C NMR spectrum of **4-*i*Pr** in acetonitrile-$d_3$ (solvent signals: *).

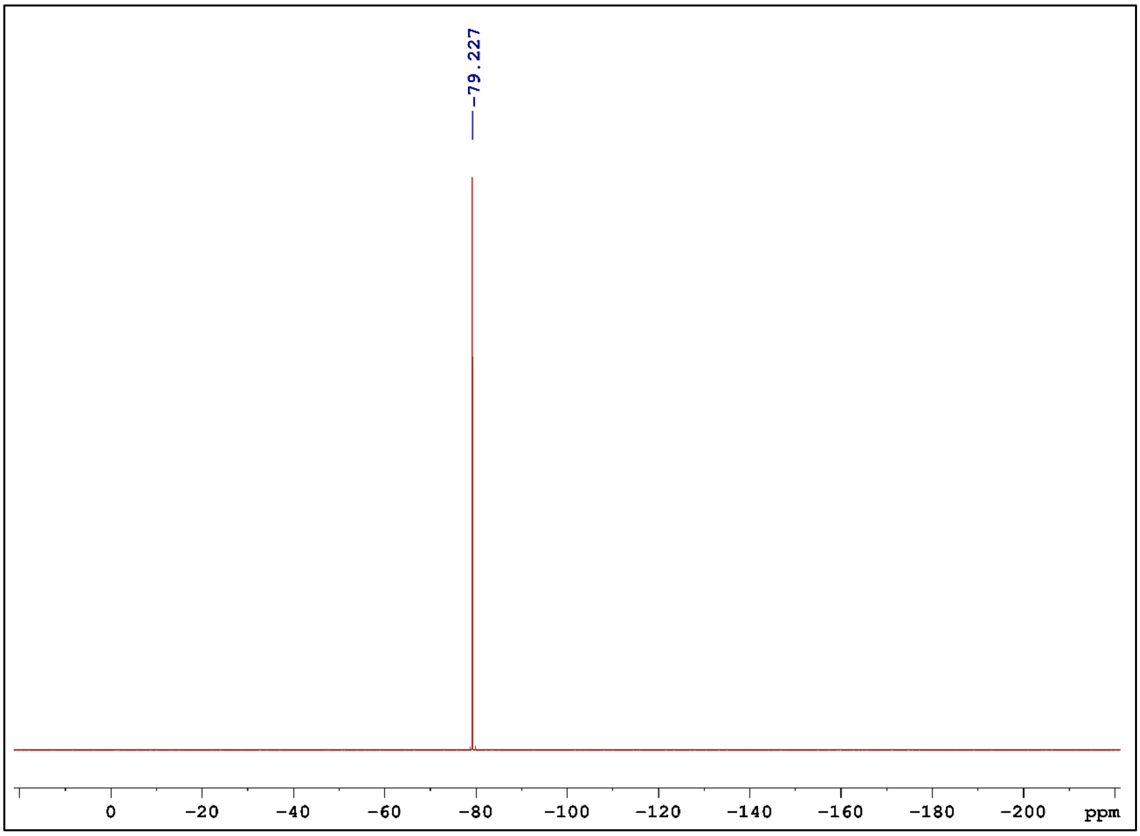


**Figure S24**: $^{19}F$ NMR spectrum of **4-*i*Pr** in acetonitrile-$d_3$.

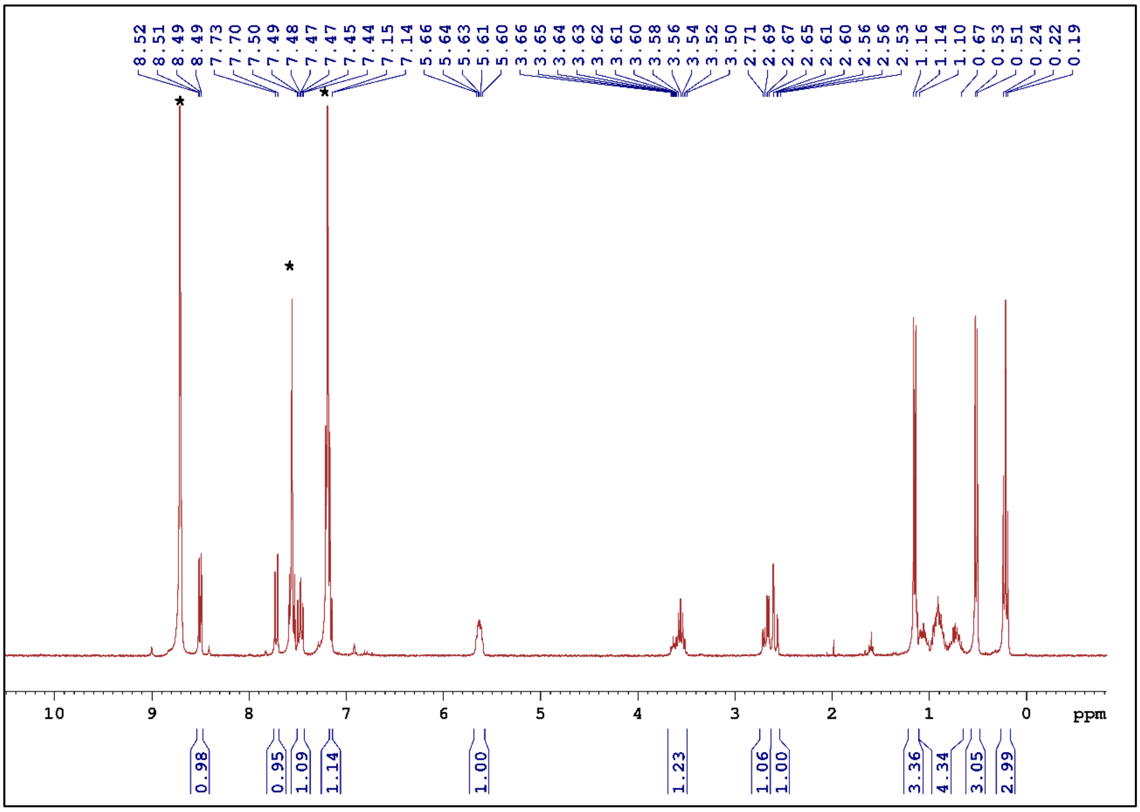


**Figure S25**: $^{1}H$ NMR spectrum of **4-*i*Pr** in pyridine-$d_5$ (solvent signals: *). Peak picking is not shown for the signals corresponding to pyridine and the resonances between 0.66-1.10 ppm for clarity.

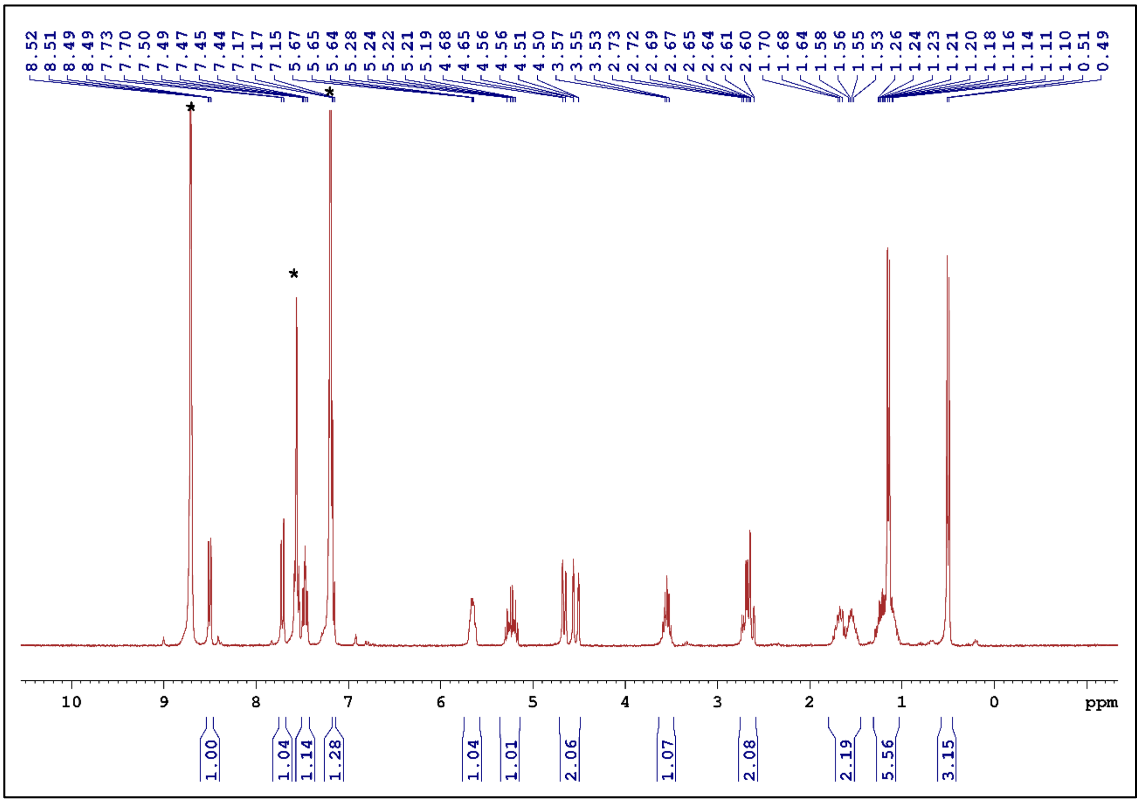


**Figure S26**: $^{1}$H NMR spectrum of **5-*i*Pr** in pyridine-$d_5$ (solvent signals: *). Peak picking is not shown for the signals corresponding to pyridine for clarity.

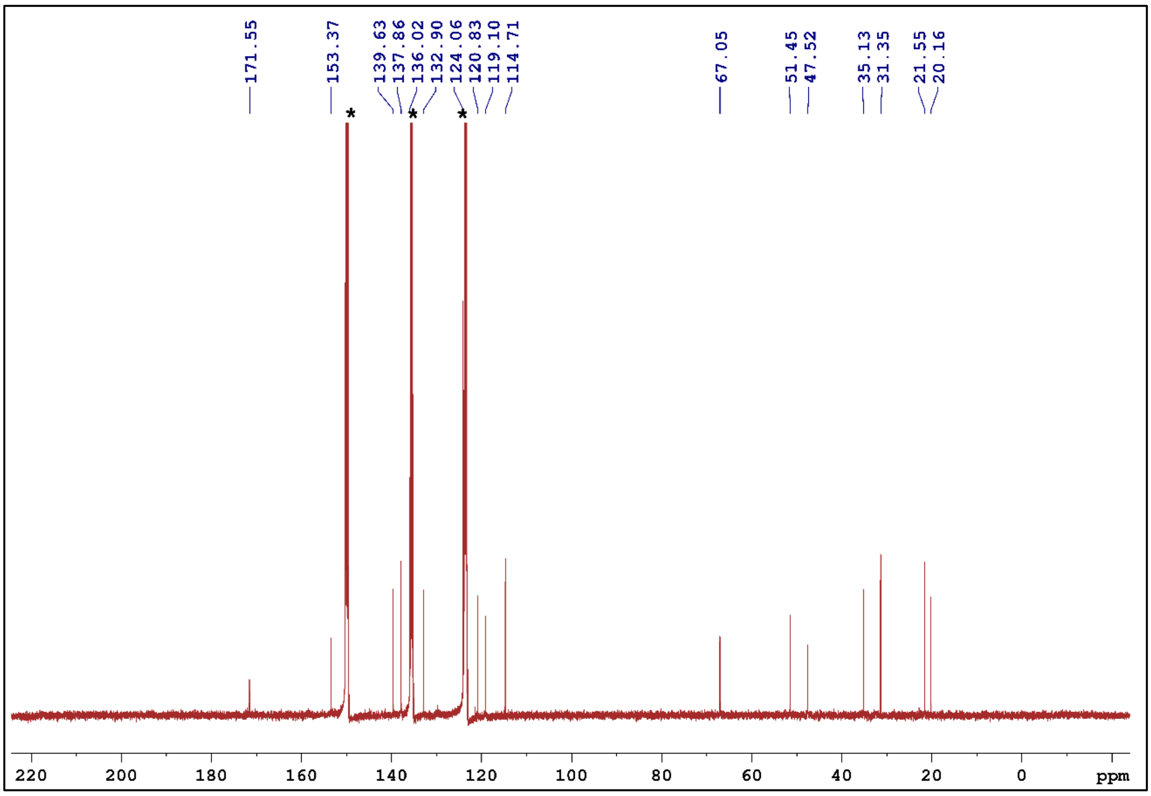


**Figure S27**: $^{13}$C NMR spectrum of **5-*i*Pr** in pyridine-$d_5$ (solvent signals: *).

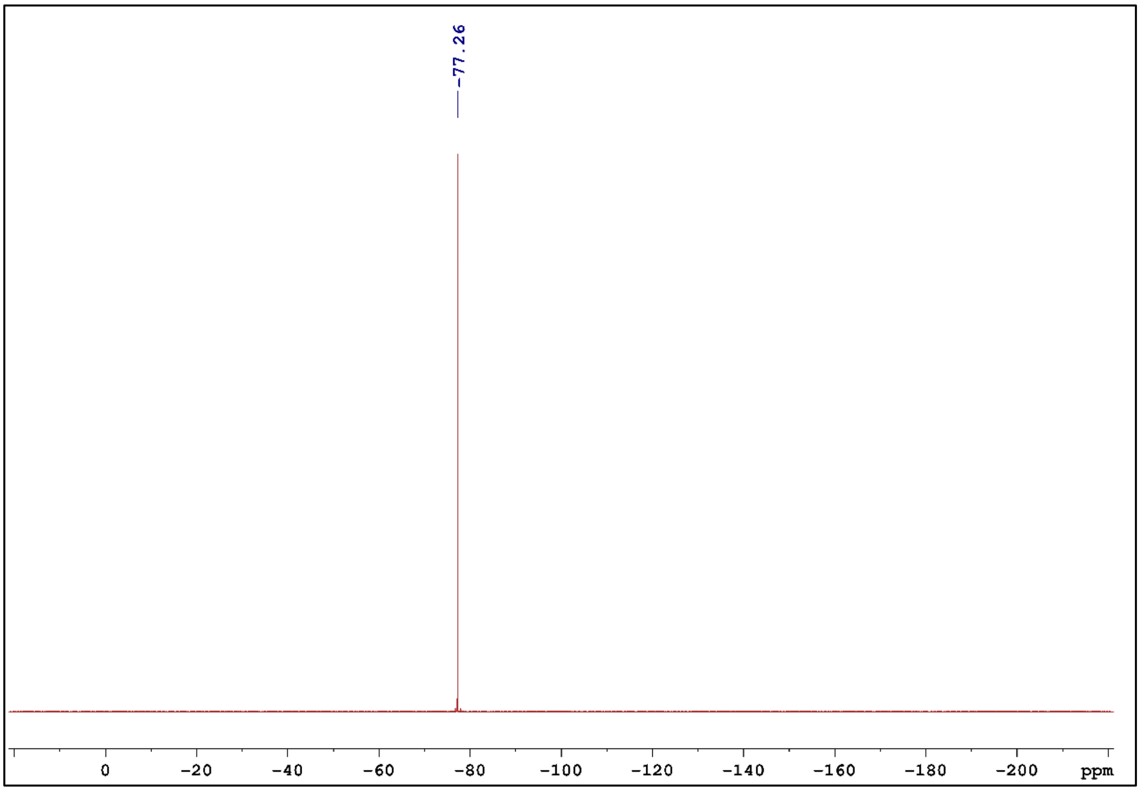


**Figure S28**: $^{19}$F NMR spectrum of **5-*i*Pr** in pyridine-$d_5$.

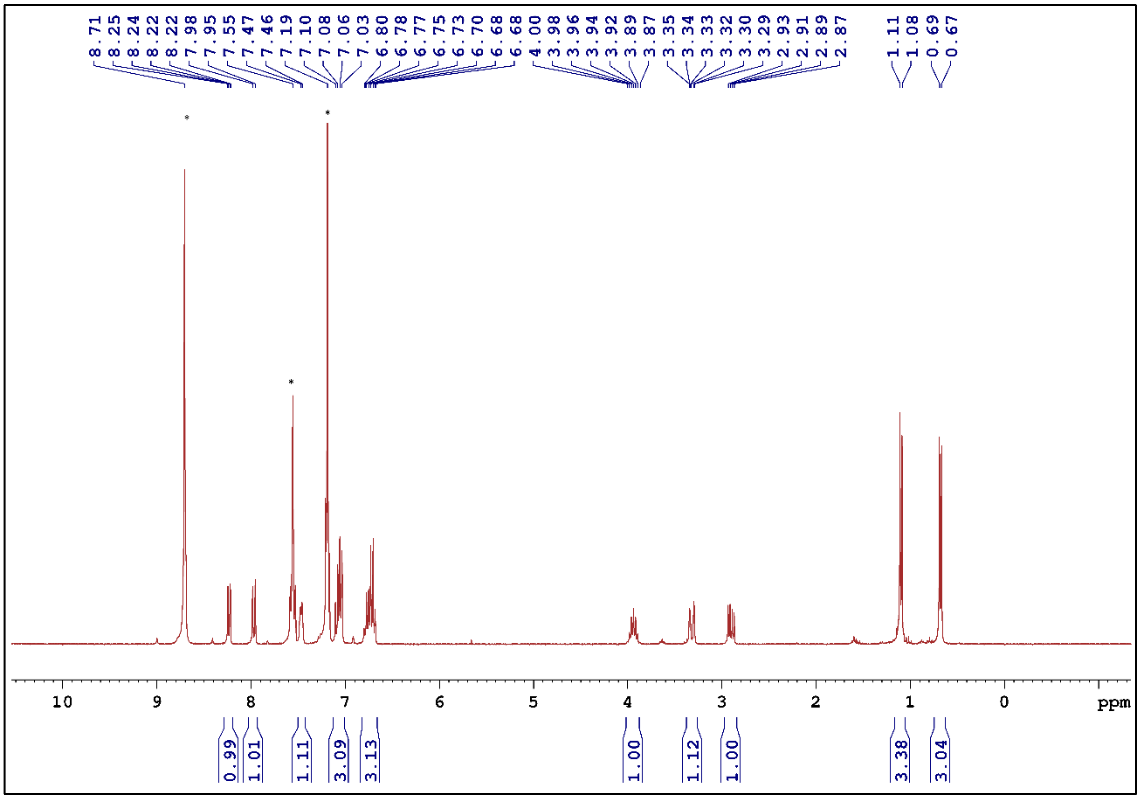


**Figure S29**: $^{1}$H NMR spectrum of **6-*i*Pr** in pyridine-$d_5$ (solvent signals: *). Peak picking is not shown for the signals corresponding to pyridine for clarity.

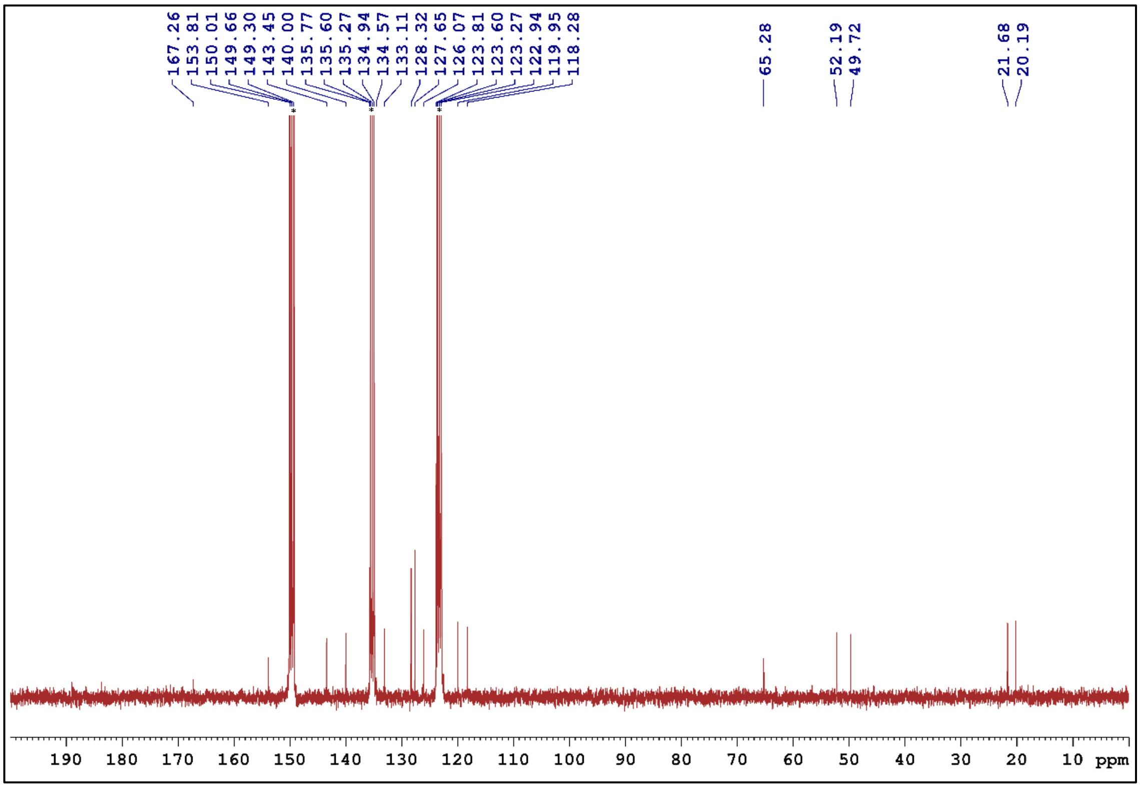


**Figure S30**: $^{13}C$ NMR spectrum of **6-*i*Pr** in pyridine-$d_5$ (solvent signals: *).

**Test reaction for olefin insertion with compound 1-Ph and 1-*i*Pr**

$[Bi_2(NPh(C_6H_4))_2(OTf)_2(thf)_3]$ (**1-Ph**) or $[Bi_2(NC_3H_7(C_6H_4))_2(OTf)_2(thf)_3]$ (**1-*i*Pr**) (5-6 mg) was dissolved in Pyridine-$d_5$ (0.4 mL) in a J-Young NMR tube and a 10-fold excess of olefin was added using a microlitre syringe. The reaction was monitored through $^1$H NMR spectroscopy. The reaction mixture was heated to 60 °C between the measurements in an NMR heating block. The substrates that showed an insertion reaction with compounds **1-Ph** and **1-*i*Pr** were scaled up through a lab-scale synthesis.

The successful formation of olefin-inserted cationic bismuth amides from compound **1-Ph** (with terminal olefins as the substrates) was accompanied by the formation of a small percentage of compound **A**[63] in the reaction mixture. The side products were successfully separated from desired insertion products through crystallisation. For reactions with compound **1-*i*Pr**, small fractions of *N*-*iso*propyl phenylamine and other unidentified side products was observed along with olefin insertion products. The side products were successfully separated from desired insertion products through crystallisation.

In reactions with internal olefins and sterically hindered olefins as substrates in pyridine-$d_5$, **1-Ph** underwent a tautomerization reaction upon heating, yielding the previously reported compound **A**.[63]

The substrates tested and the reactivity exhibited by them are summarized in the table below.

**Table S1.** Reactivity of olefin substrates with compounds **1-Ph** and **1-*i*Pr**.

| Compound | Substrate | Product |
|---|---|---|
| 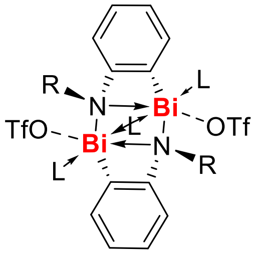 **1-R** **1-Ph**; R = Ph **1-*i*Pr**, R = *i*Pr | 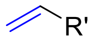 R' = H, $C_3H_7$, $C_4H_9$, $C_4H_7$, Ph | 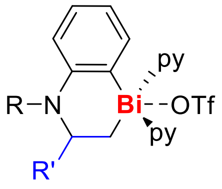 |
| 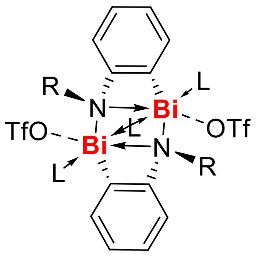 **1-R** **1-Ph**; R = Ph **1-*i*Pr**, R = *i*Pr | 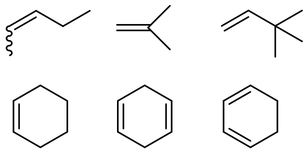 | For **1-Ph**; 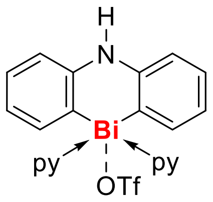 **(A)** For **1-*i*Pr;** 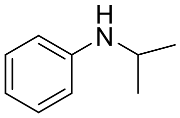 (suggested based on $^1$H NMR spectroscopy) + unidentified products |

**Tests for the release of olefins**

Isolated products from olefin insertion reactions were investigated in solution for their potential to release the olefin in the absence of other external reagents. Specifically, the products obtained from insertion reactions between an olefin and compound **1-Ph** were dissolved in a deuterated solvent of choice (0.5 mL) in a J-Young NMR tube. The course of the reaction was monitored by $^{1}$H NMR spectroscopy at temperatures of 60-70 °C with reaction times of up to several days. The experiment was continued until olefin release was completed. The experimental conditions and results are summarised in the table below.

**Table S2.** Reversibility experiments performed.

| **Insertion product** ( 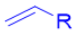 used ) | **Solvent, Temperature** | **Products observed** | **Time taken for full release** |
|---|---|---|---|
| **6-Ph** (styrene) | pyridine-$d_5$, r.t. | 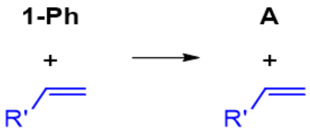  | 21 days |
| | THF-$d_8$, r.t. | A + styrene | 12 days |
| **2-Ph** (ethylene) | pyridine-$d_5$, 80 °C | No olefin release for 2 weeks | |
| | THF-$d_8$, 80 °C | A + ethylene + unidentified products | 14 days |
| **3-Ph** (1-hexene) | pyridine-$d_5$, 80 °C | A + 1-hexene | 10 days |
| | THF-$d_8$, 70 °C | A + 1-hexene | 1 day |
| **4-Ph** (1-pentene) | pyridine-$d_5$, 80 °C | A + 1-pentene | 10 days |
| | THF-$d_8$, 70 °C | A + 1-pentene | 1 day |
| **5-Ph** (1,5-hexadiene) | pyridine-$d_5$, 80 °C | A + 1,5-hexadiene | 10 days |

**Styrene insertion product (6-Ph)**

In pyridine-$d_5$, release of styrene starts within a day at room temperature. The release of olefin from the insertion product is accompanied by formation of the starting material **(1-Ph)**. Over the course of 21 days, the styrene insertion product was completely transformed into free styrene and compound **A**. The NMR spectrum points to formation of compound **1-Ph** *in-situ*, which tautomerizes into compound **A.**

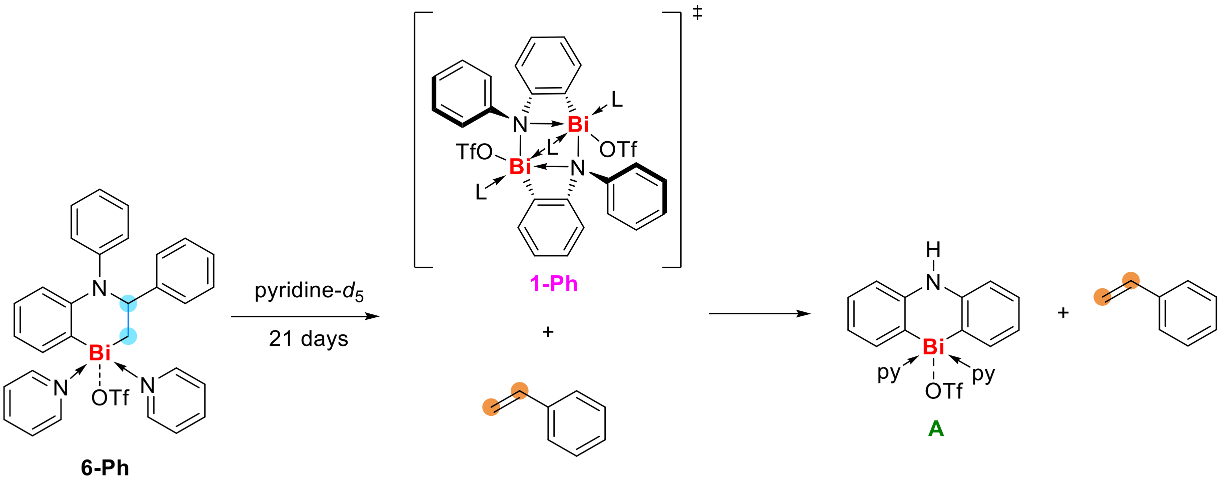


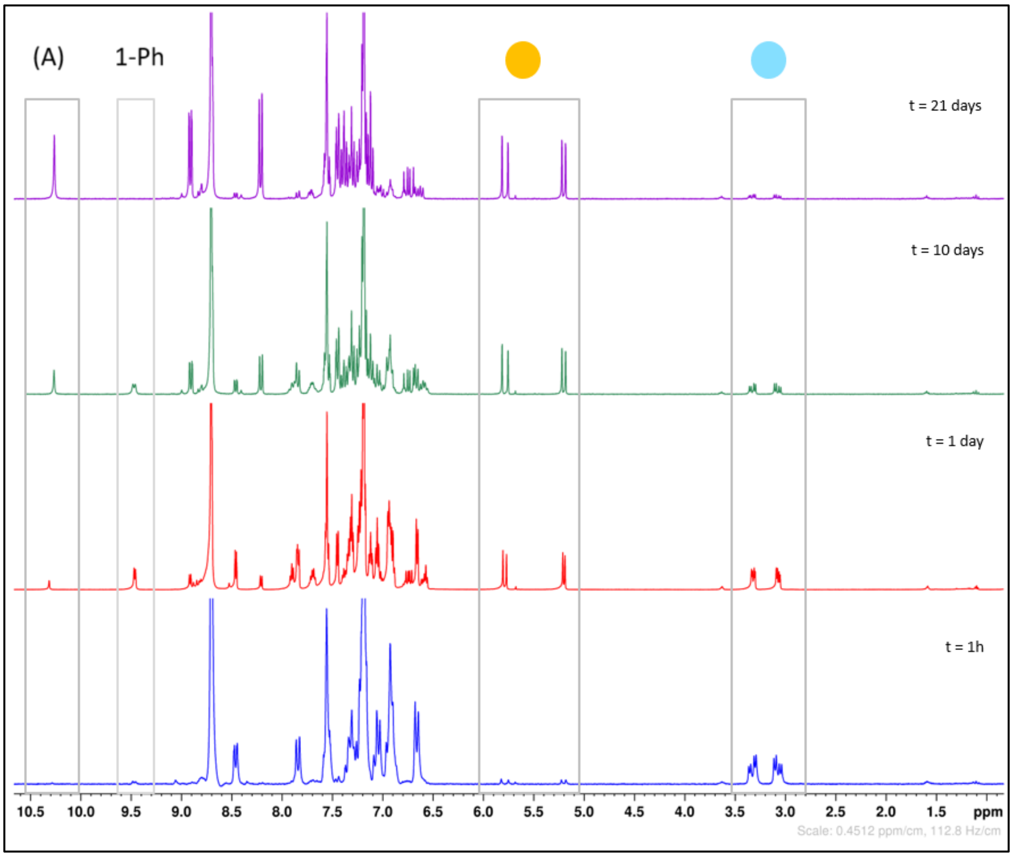


**Figure S31.** Reaction monitoring through $^{1}$H NMR spectroscopy of **6-Ph** in pyridine $d_5$. Characteristic peaks of compounds observed are denoted through following symbols: blue circle: compound **6-Ph**; orange circle: styrene; 1-Ph: compound **1-Ph**; (A): compound **A**.

In THF-$d_8$, a quantitative release of styrene from **6-Ph** could be observed within 12 days. Unlike in pyridine-$d_5$, the re-formation of the starting material **1-Ph** could not directly be observed in the $^1H$ NMR spectroscopic monitoring. Rather, the signals of the second CH activated product i.e., compound **A**, was observed.

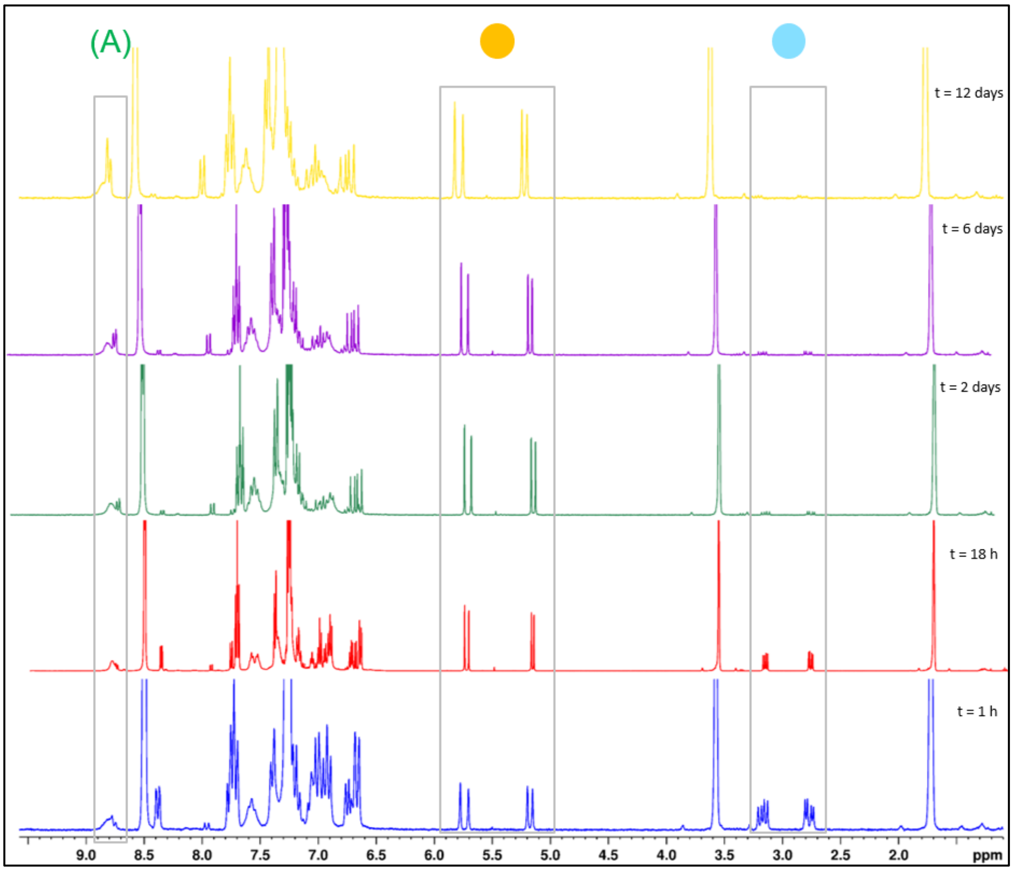


**Figure S32.** Reaction monitoring through $^1H$ NMR spectroscopy of **6-Ph** in THF $d_8$. Characteristic peaks of compounds observed are denoted through following symbols: blue circle: compound **6-Ph**; orange circle: styrene; (A): compound **A**.

**Ethene insertion product**

Heating a solution of compound **2-Ph** in pyridine-$d_5$ to 80 °C for 14 days did not release ethylene. No change in the $^{1}$H NMR spectrum was observed.

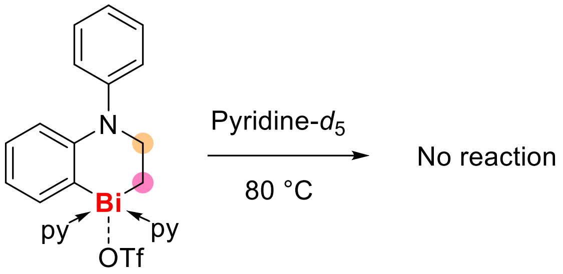


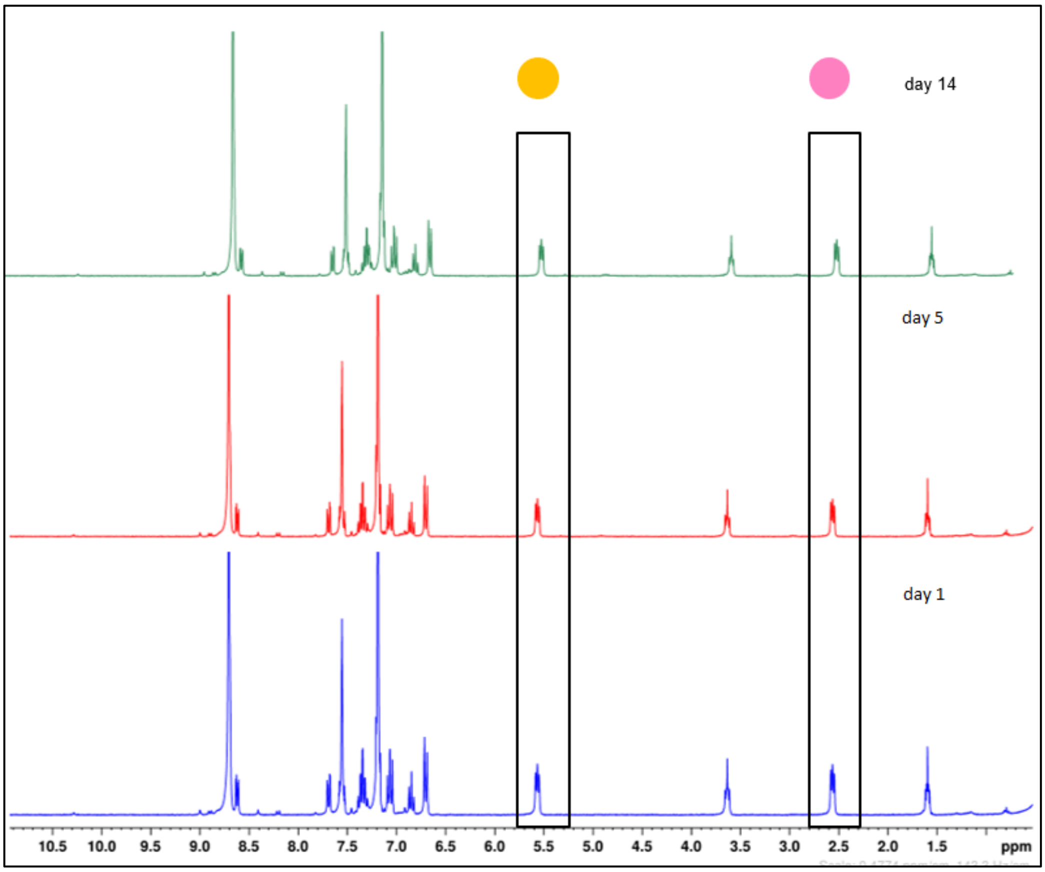


**Figure S33.** Reaction monitoring through $^{1}$H NMR spectroscopy of heating **2-Ph** in pyridine-$d_5$. Characteristic peaks of compounds observed are denoted through the following symbols: pink circle: 1-$CH_2$ protons of ethylene insertion product. orange circle: 2-CH proton of ethylene insertion product.

However, upon heating a THF-$d_8$ solution of **2-Ph** to 80 °C in a J-Young type NMR tube, ethylene release could be detected. In 14 days, a quantitative conversion with release of ethylene along with formation of compound **A** was observed via $^{1}$H NMR spectroscopy.

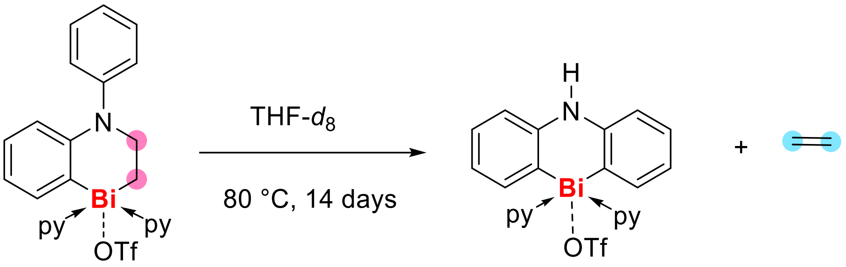


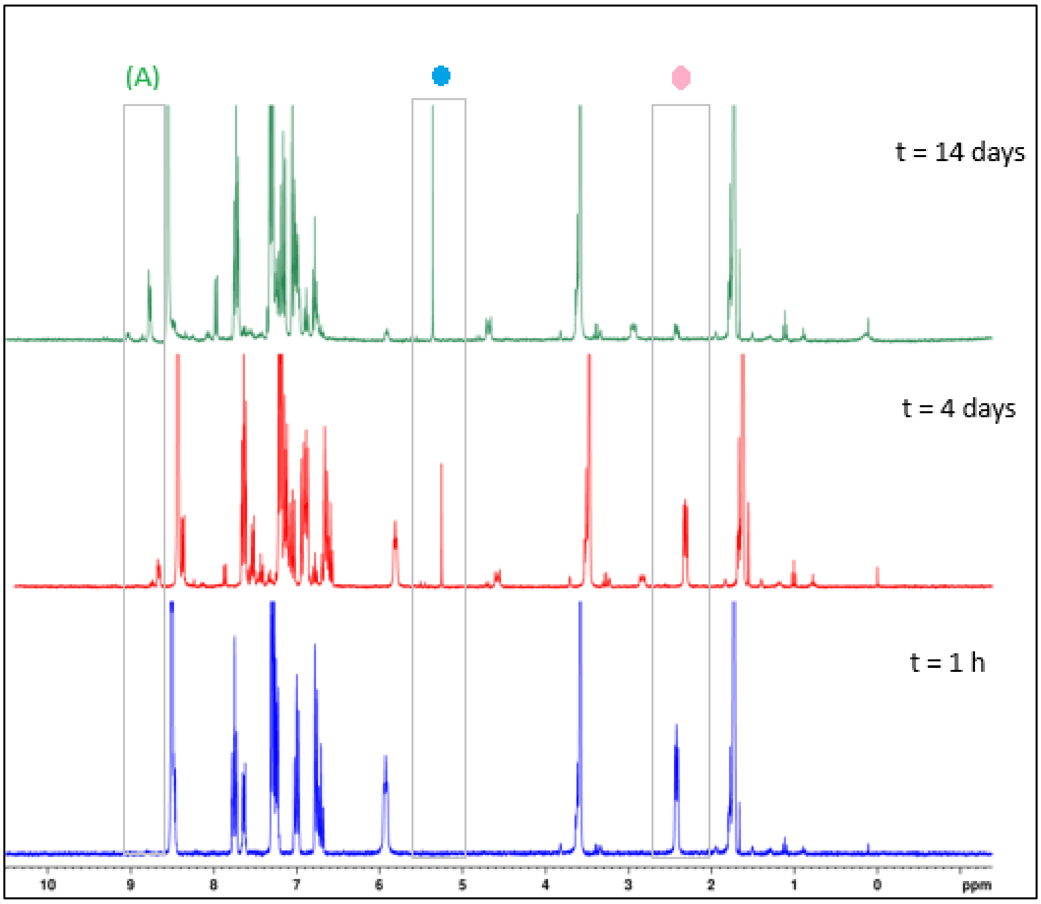


**Figure S34.** Reaction monitoring through $^{1}$H NMR spectroscopy of heating **2-Ph** in THF $d_8$. Characteristic peaks of compounds observed are denoted through the following symbols: pink circle: compound **2-Ph**; blue circle: ethylene; (A): compound **A**.

**1-hexene insertion product (3-Ph)**

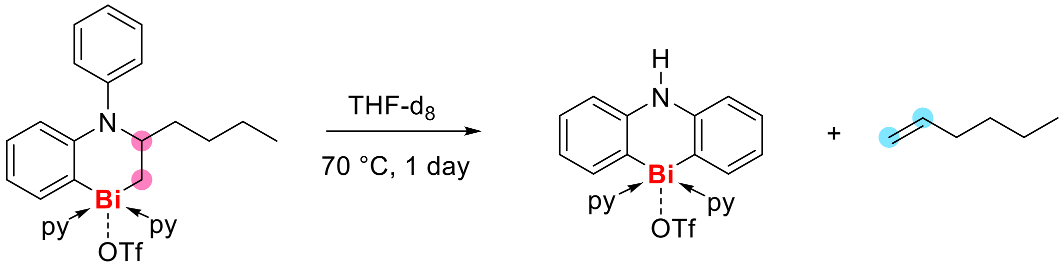


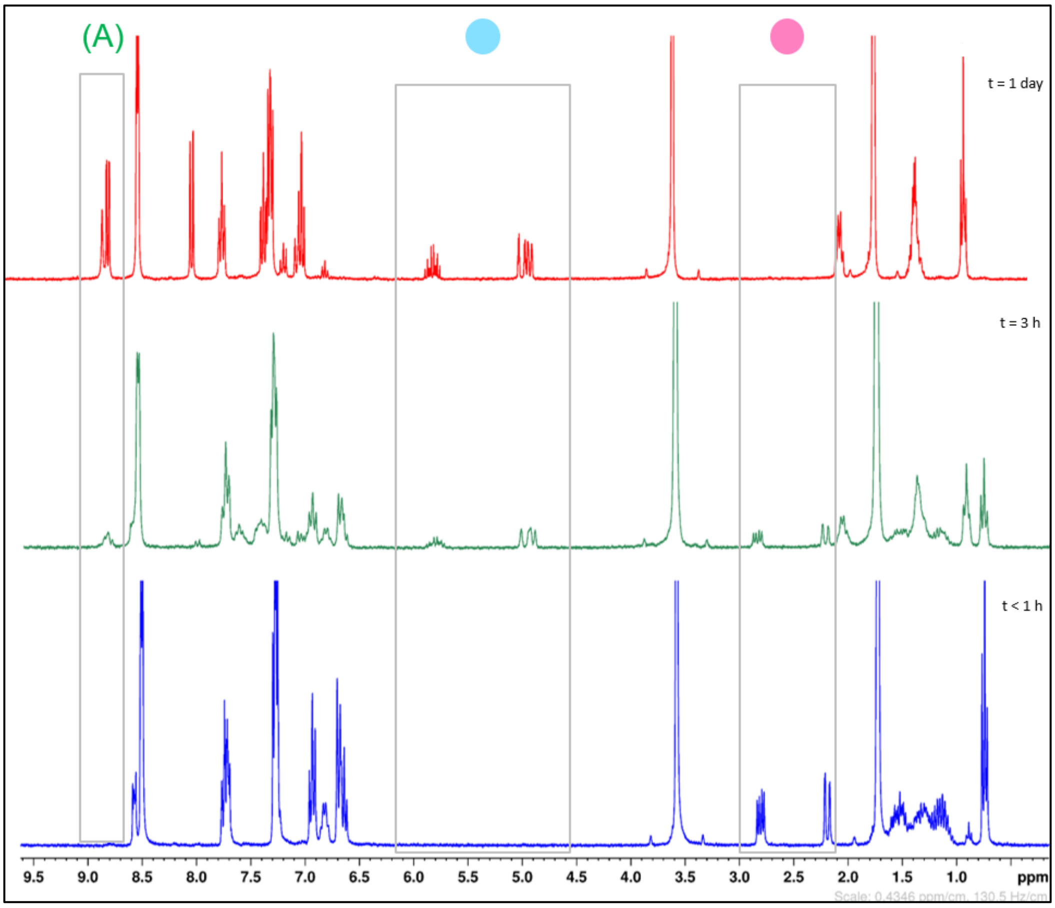


**Figure S35.** Reaction monitoring through $^{1}H$ NMR spectroscopy of heating **3-Ph** in THF $d_8$. Characteristic peaks of compounds observed are denoted through following symbols: pink circle: compound **3-Ph**; blue circle: hexene; (A): compound **A**.

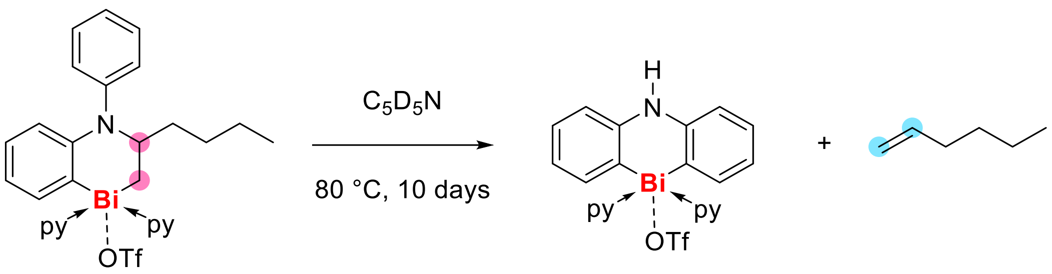


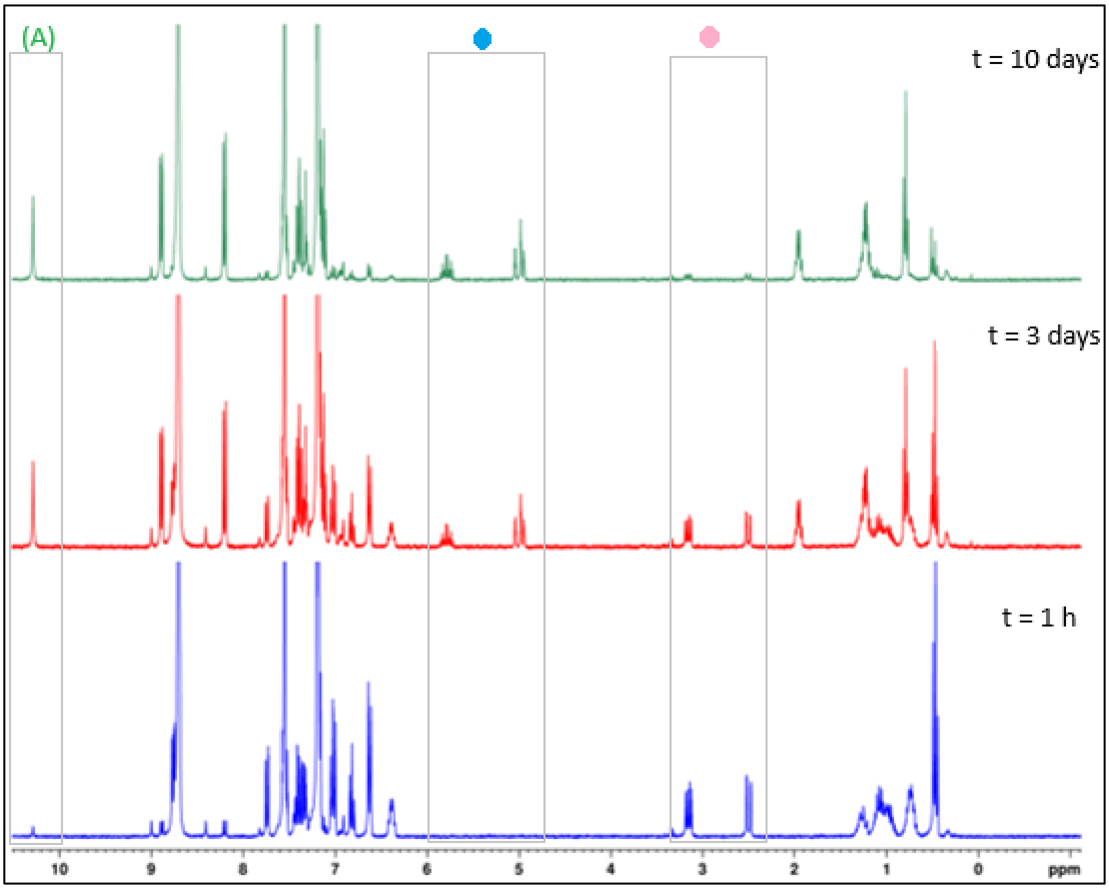


**Figure S36.** Reaction monitoring through $^{1}H$ NMR spectroscopy of heating **3-Ph** in pyridine-$d_5$. Characteristic peaks of compounds observed are denoted through following symbols: pink circle: compound **3-Ph**, blue circle: 1-hexene; (A): compound **A**.

**1-pentene insertion product (4-Ph)**

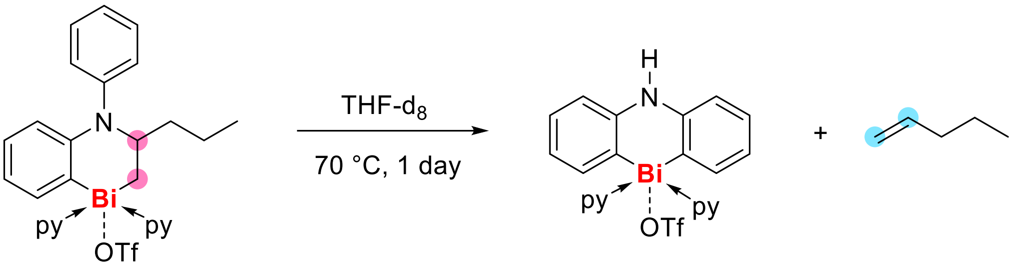


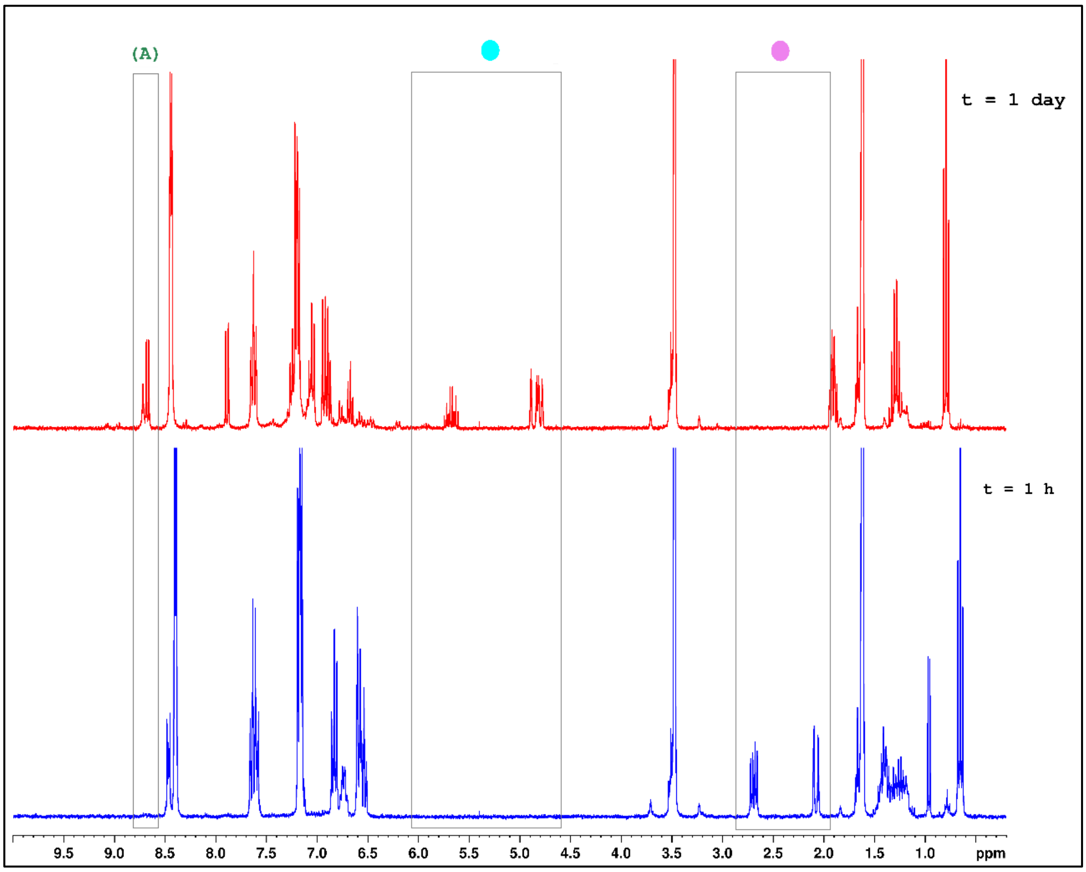


**Figure S37**: Reaction monitoring through $^{1}$H NMR spectroscopy of heating **4-Ph** in THF $d_8$. Characteristic peaks of compounds observed are denoted through following symbols: pink circle: compound **4-Ph**; blue circle: 1-pentene; (A): compound **A**.

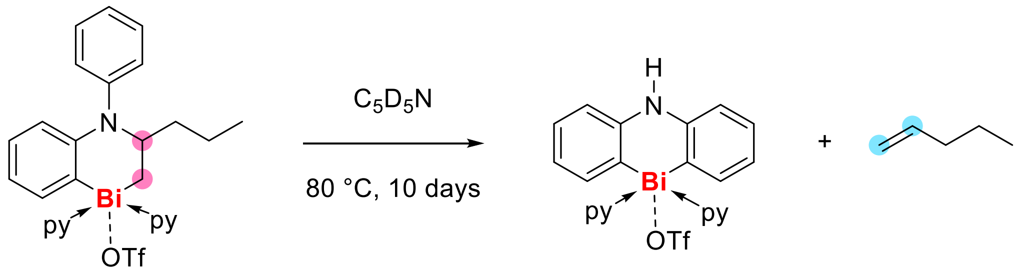


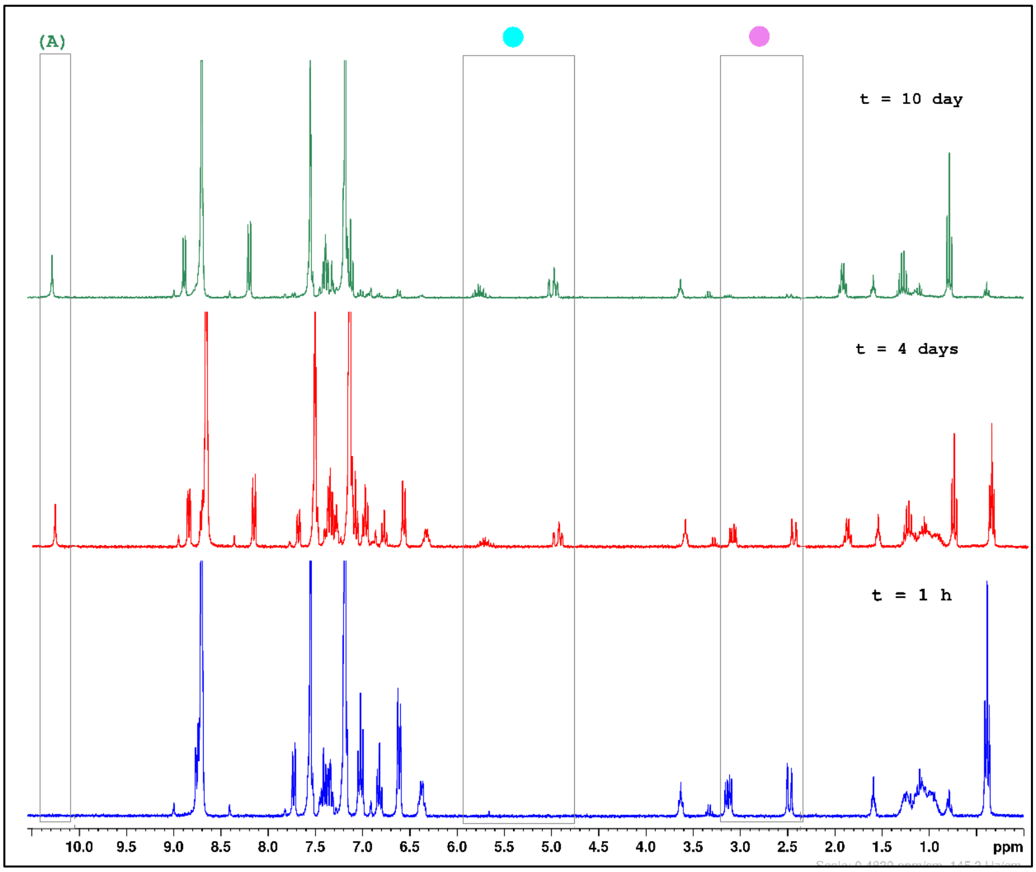


**Figure S38.** Reaction monitoring through $^1$H NMR spectroscopy of heating **4-Ph** in pyridine-$d_5$. Characteristic peaks of compounds observed are denoted through following symbols: pink circle: compound **4-Ph**, blue circle: 1-pentene and (A): compound **A**.

**1,5-hexadiene insertion product (5-Ph)**

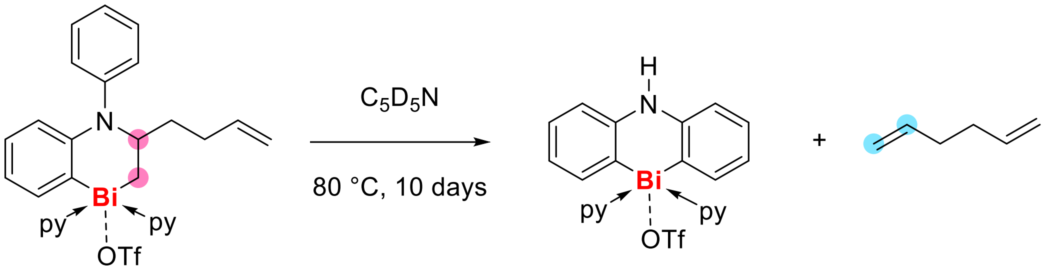


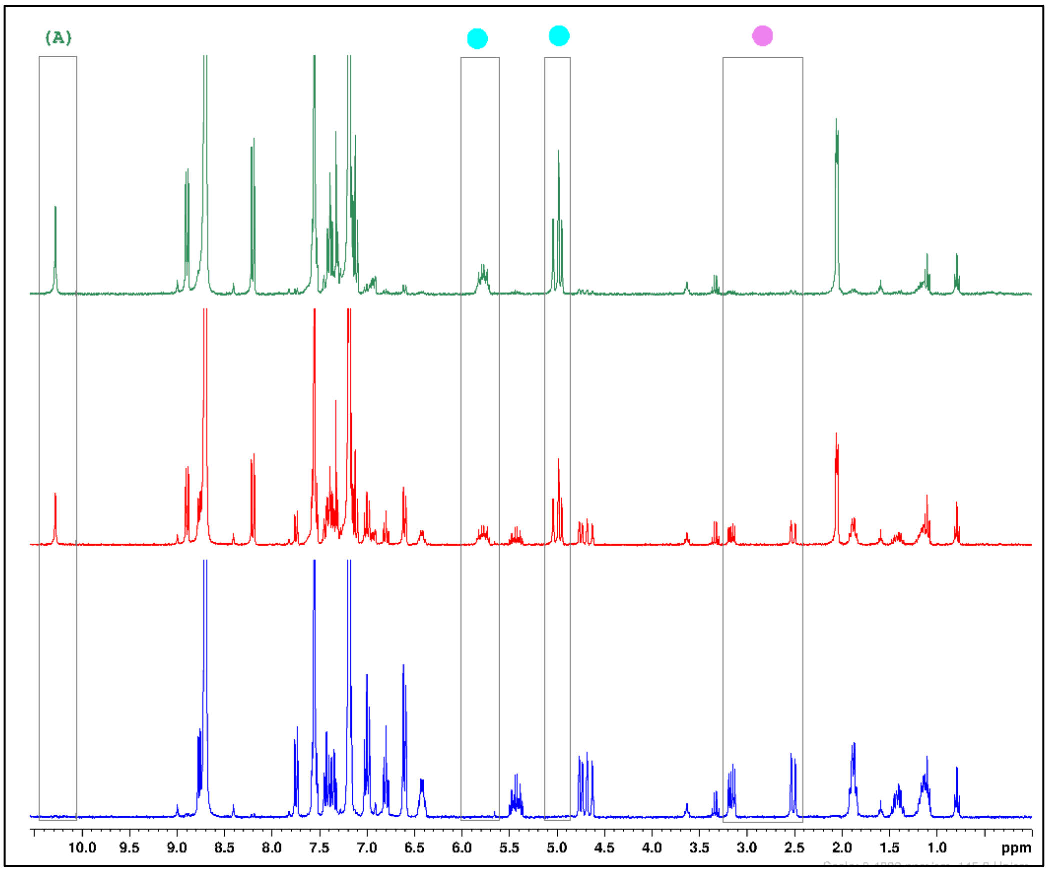


**Figure S39.** Reaction monitoring through $^{1}$H NMR spectroscopy of heating **5-Ph** in pyridine-$d_5$. Characteristic peaks of compounds observed are denoted through following symbols: pink circle: compound **5-Ph**, blue circle: 1,5-hexadiene and (A): compound **A**.

**Test for olefin release with 4-*i*Pr.**

At room temperature in solution (pyridine-$d_5$), the compound was found stable for several days. The isolated compound **4-*i*Pr** was then heated to 100 °C in pyridine-$d_5$ and monitored using $^1$H NMR spectroscopy. 1-pentene release (≈90 %) was observed after 2 days. All volatiles were distilled from the reaction mixture and collected, and were found to be the released olefin in pyridine-$d_5$. The remaining residue was again dissolved in pyridine-$d_5$ and analyzed by NMR spectroscopy. Signals were detected which match those of the isopropyl and phenyl group of the free amine HN*i*PrPh in multiplicity, chemical shift, and relative intensity. The signals of the residual product also matched with those detected after extensive heating of **1-*i*Pr** in pyridine-$d_5$, indicating a common decomposition pattern.

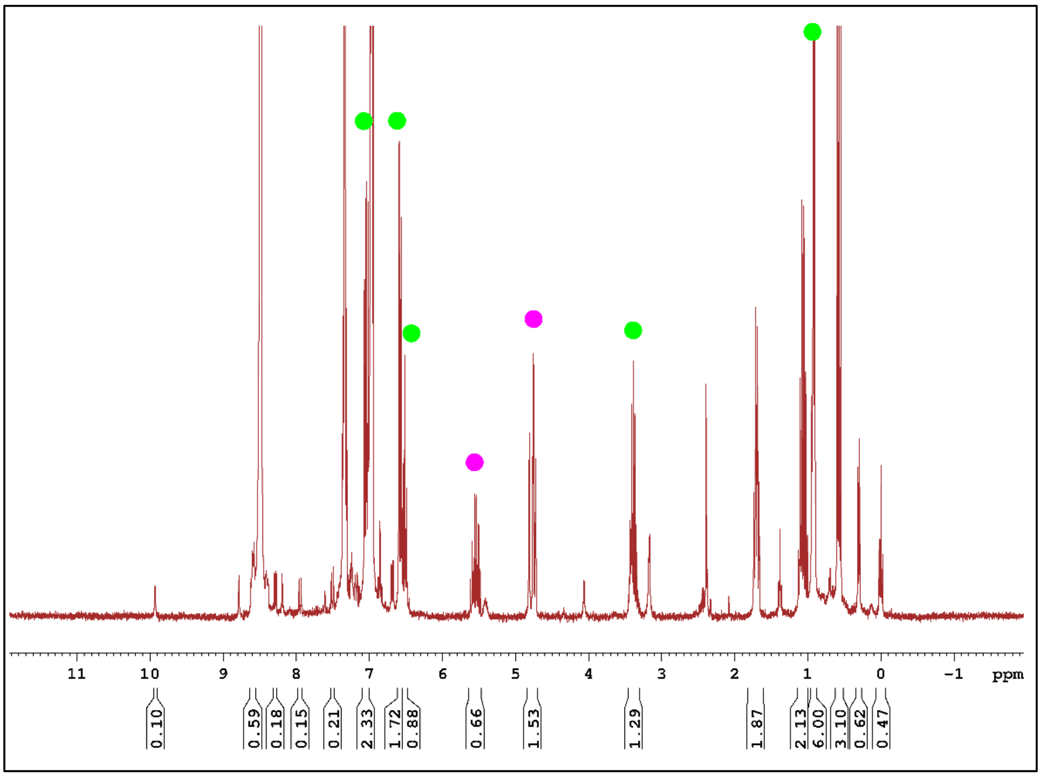


**Figure S40.** $^1$H NMR spectrum obtained after heating **4-*i*Pr** in pyridine-$d_5$ to 100 °C for 2 days. Pink circles denote free 1-pentene. Green circle denotes *N*-(isopropyl)phenyl group.

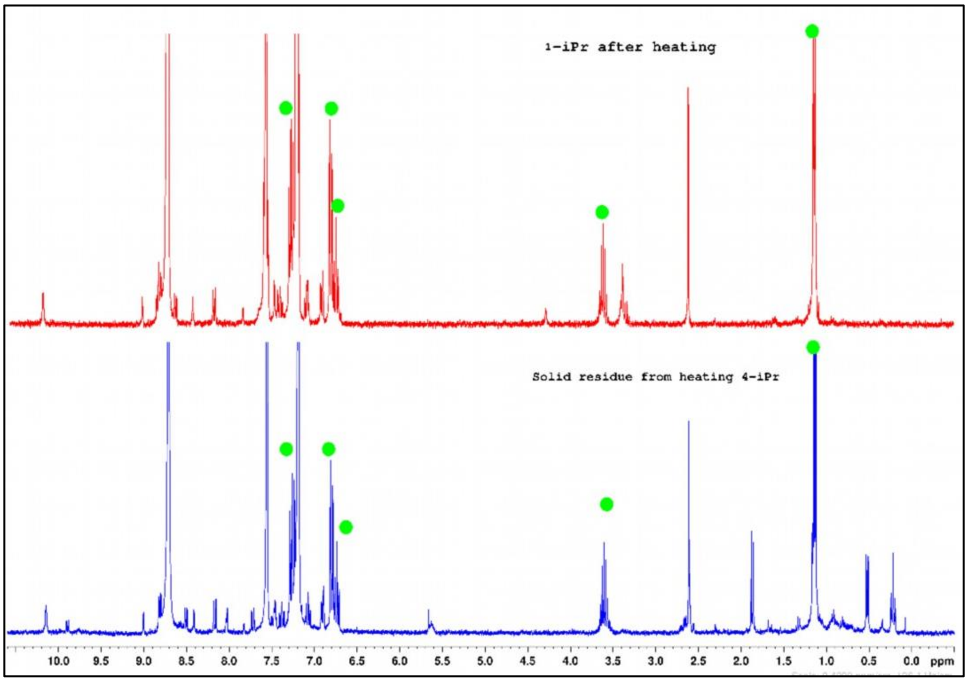


**Figure S41.** Comparison of $^{1}$H NMR spectra of the residue obtained after distilling off the volatiles from heating **4-*i*Pr** (blue spectrum) and heating **1-*i*Pr** at 80 °C for 2 days (red spectrum). Green circles indicate signals that could belong to a *N*-(isopropyl)phenyl group. Other unidentified signals can also be seen.

## Exchange experiments

Exchange experiments were tested between a defined olefin insertion product and an excess (ca. 100 equiv.) of another olefin. An exchange reaction between the olefins (forming the insertion product of the olefin added) would suggest that the starting material is formed *in-situ* and that the olefin insertion is reversible.

In a typical experimental approach, a defined amount of the product obtained from the insertion reaction between a specific olefin and compound **1-Ph**, is dissolved in pyridine-$d_5$ in a J-Young NMR tube. To the solution, a hundred-fold excess of another olefin is added. The reaction mixture is heated to defined temperatures of 50-100 °C (different temperatures used for different substrates, selected results given in the table below). The reaction was monitored periodically using $^{1}$H NMR spectroscopy. The substrates used, specific reaction conditions employed, and their outcomes are summarised in the table below.

**Table S3.** Substrates tested and outcomes for the tested exchange experiments (in pyridine-$d_5$).

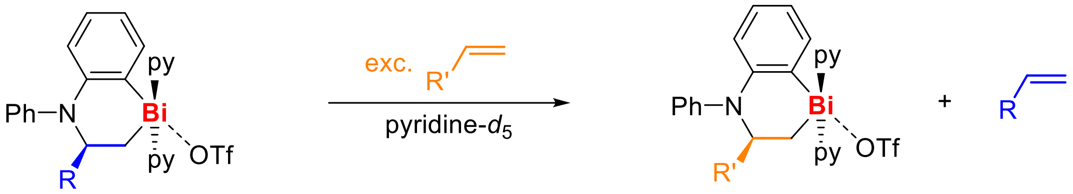


| Experiment no. | Bi complex (starting material) | R | R' | Conditions | Exchange (yes/no) |
|---|---|---|---|---|---|
| 1a | **6-Ph** | Ph | *n*Bu | 60 °C, 1 day | Yes |
| 1b | **6-Ph** | Ph | *n*Pr | 60 °C, 1 day | Yes |
| 1c | **6-Ph** | Ph | $C_2H_4CH{=}CH_2$ | 50 °C, 1 day | Yes |
| 2a | **3-Ph** | *n*Bu | *n*Pr | 80 °C, 7 days | No[a] |
| 2b | **3-Ph** | *n*Bu | $C_2H_4CH{=}CH_2$ | 80 °C, 7 days | No[a] |
| 3 | **2-Ph** | H | *n*Bu | 80 °C, 7 days | No |
| 4 | **2-Ph** | H | $C_2H_4CH{=}CH_2$ | 80 °C, 7 days | No |
| 5 | **5-Ph** | $C_2H_4CH{=}CH_2$ | *n*Bu | 80 °C, 8 days | No |

a: compound **A** was formed.

More detailed descriptions of the reaction conditions and reaction monitoring are given below.

**Styrene insertion product with excess olefins:**

A pyridine-$d_5$ solution of **6-Ph** was left at room temperature for one day. The *in-situ* formation of **1-Ph** (8%) was observed. The solutions were then reacted with an excess of 1-hexene (in reaction 1a), 1-pentene (in reaction 1b) and 1,5-hexadiene (in reaction 1c). After heating the reaction mixtures to 60 °C for one day, the formation of respective insertion products of 1-hexene: **3-Ph** (89%) and 1-pentene: **4-Ph** (84%) were observed. A small amount of the second-CH activated compound (**A**) was a side product for both reactions (reaction 1a: 11%, reaction 1b: 16%). For the exchange reaction with 1,5-hexadiene, a reaction at 60 °C only gave a 6.5% conversion. Lowering the reaction temperature to 50 °C showed the formation of **5-Ph** in 32 % yield.

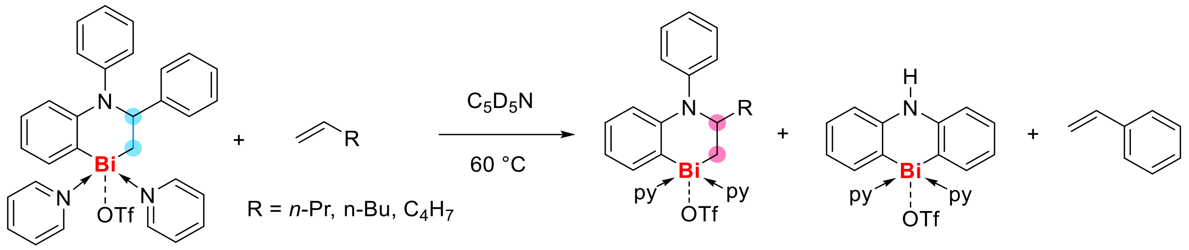


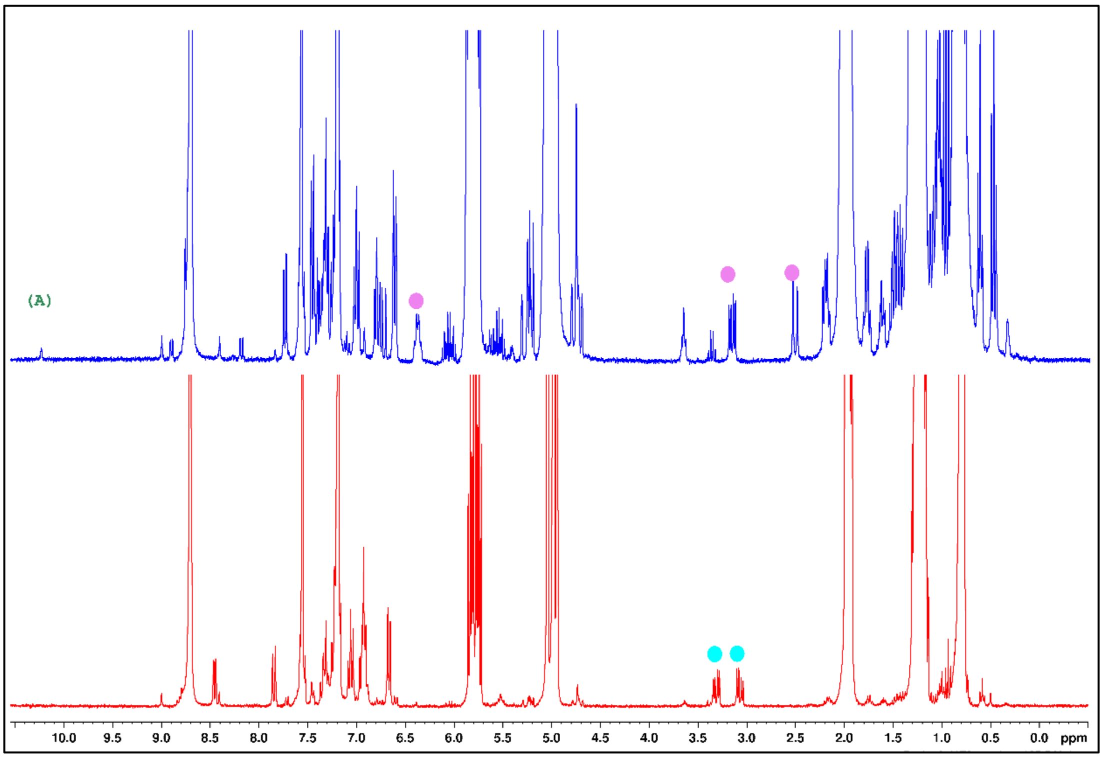


**Figure S42**: $^1$H NMR spectra for exchange experiment 1a. Bottom (red): spectrum obtained immediately after addition of a 100-fold excess of 1-hexene added to **6-Ph** in pyridine-$d_5$. Top (blue) spectrum of reaction mixture heated to 60 °C for 20 hours. Characteristic peaks of compounds observed are denoted through following symbols: blue circle- styrene insertion product, pink circle- hexene insertion product, **(A)**- compound **A**.

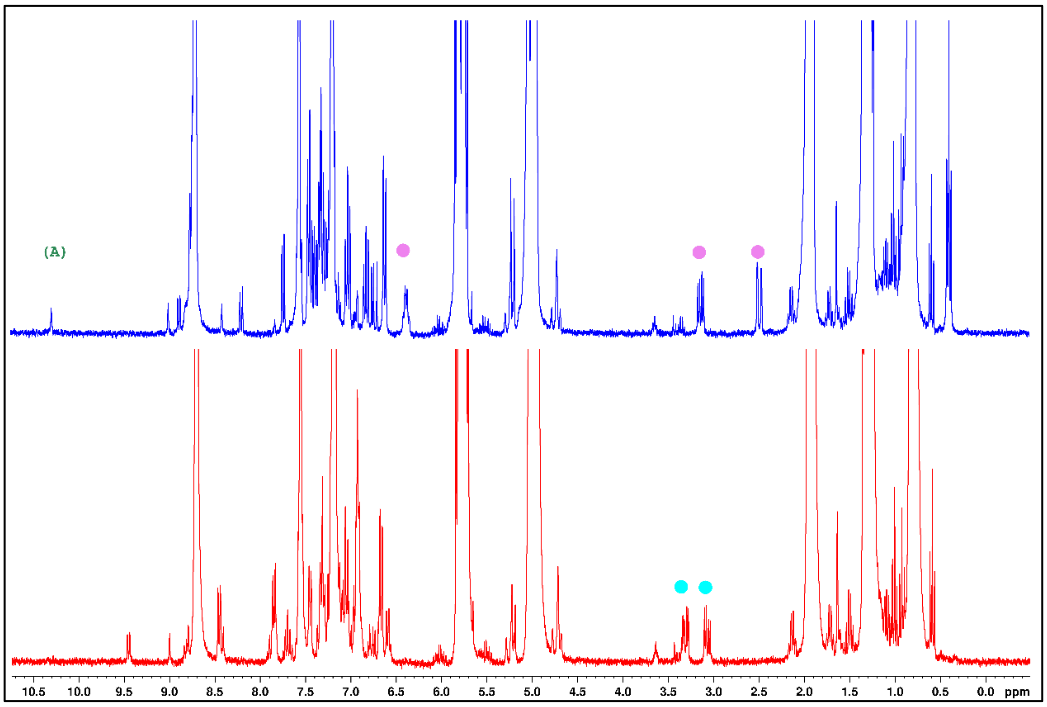


**Figure S43**: $^{1}$H NMR spectra for exchange experiment 1b; red spectrum (below) immediately after addition of a hundred-fold excess of 1-pentene added to styrene insertion product in pyridine-*d*5; blue spectrum (above) - reaction mixture heated to 60 °C for 20 hours. Characteristic peak of compounds observed are denoted through following symbols: blue circle- styrene insertion product, pink circle- 1-pentene insertion product, **(A)**- compound A.

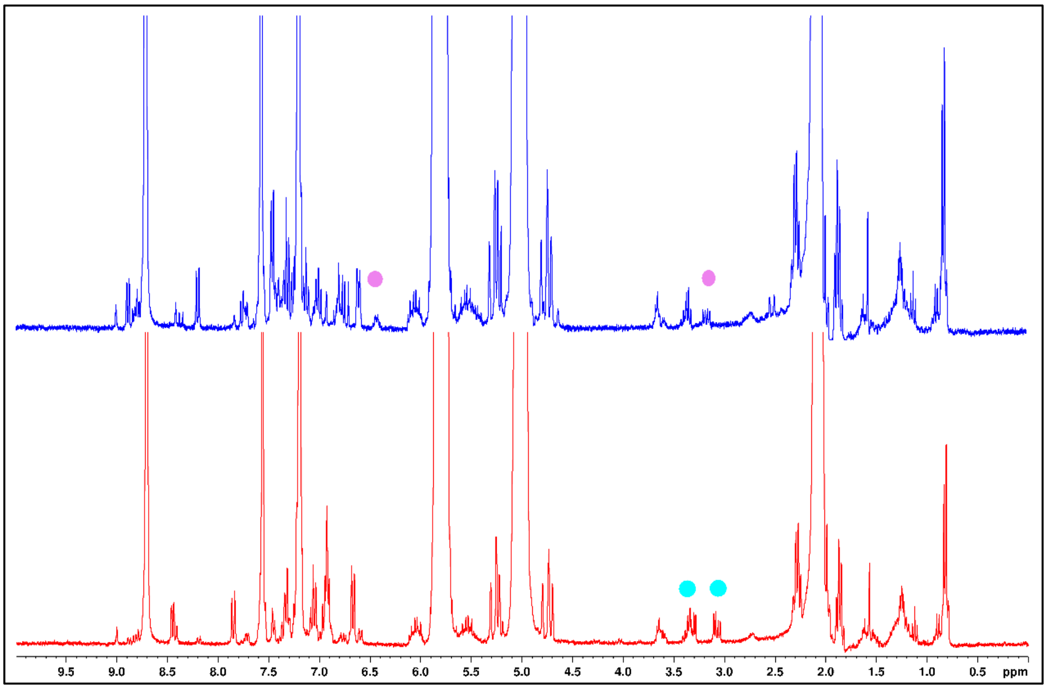


**Figure S44**: $^{1}$H NMR spectra for exchange experiment 1c; red spectrum (below) immediately after addition of a hundred-fold excess of 1,5-hexadiene added to styrene insertion product in pyridine-*d*5; blue spectrum (above)- reaction mixture heated to 50 °C for 20 hours. Characteristic peak of compounds observed are denoted through following symbols: blue circle- styrene insertion product, pink circle- 1,5-hexadiene insertion product, **(A)**- compound A.

**Competition experiments: C=C insertion or C=O insertion?**

Due to the polar nature of the carbonyl double bond, carbonyl species- ketones, aldehydes, esters and amides-are generally more reactive compared to the olefinic double bond. Compound **1-Ph** showed selective insertion of C=C bonds from different olefins. So, a competition experiment was conceptualised to test if **1-Ph** could selectively insert alkenes over carbonyl compounds.

In a J-Young NMR tube, 5 mg of **1-Ph** was dissolved in 0.5 mL pyridine-*d*5. To the solution 20 equivalents of 1-hexene and 20 equivalents of respective carbonyl compound were added. The reaction mixture was then heated to 60 °C, and reaction monitored periodically using $^{1}$H NMR spectroscopy. The results are summarized in the table below.

**Table S4**: carbonyl compounds tested in competition reaction experiment with **1-Ph**.

| Carbonyl compound | Reaction observed |
|---|---|
| 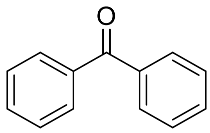 | 1-hexene insertion<br>(benzophenone unreacted) |
| 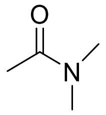 | 1-hexene insertion + second-CH activation<br>(N,N-dimethylacetamide unreacted) |
| 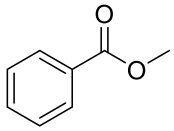 | 1-hexene insertion<br>(methyl benzoate unreacted) |
| 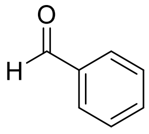 | Carbonyl bond insertion<br>(1-hexene unreacted) |
| 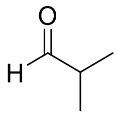 | Unselective reaction |

As pointed out the main part, the softly Lewis acidic nature of **1-Ph** is argued to be one factor for olefin insertion being favored over carbonyl insertion in the cases that do not represent an aromatic aldehyde. In addition, a combination of the electrophilicity of the carbonyl functional group, sterics, and the potential to engage in side reactions are likely to also impact the reactivity of **1-Ph** towards substrates with unsaturated functional groups such as olefins and carbonyl compounds.

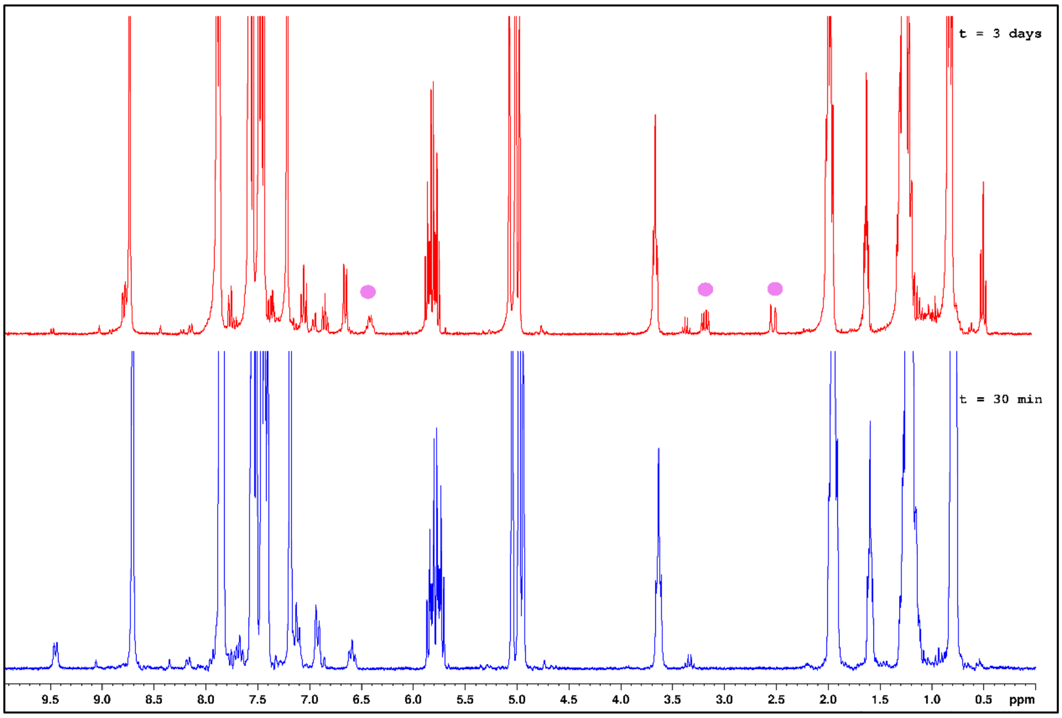


**Figure S45**: $^{1}H$ NMR spectrum for competitive reaction study of benzophenone and 1-hexene with **1-Ph** in pyridine-*d*5; blue spectrum (below)- 30 minutes after addition of benzophenone (7.8 µL, 20 eq.), 1-hexene (5.9 µL, 20 eq.) to **1-Ph** (3 mg, 1 eq.) in 0.5 mL pyridine-*d*5, red spectrum – after heating the reaction mixture to 60 °C for 3 days. Pink circle denotes the characteristic peak for 1- hexene insertion product.

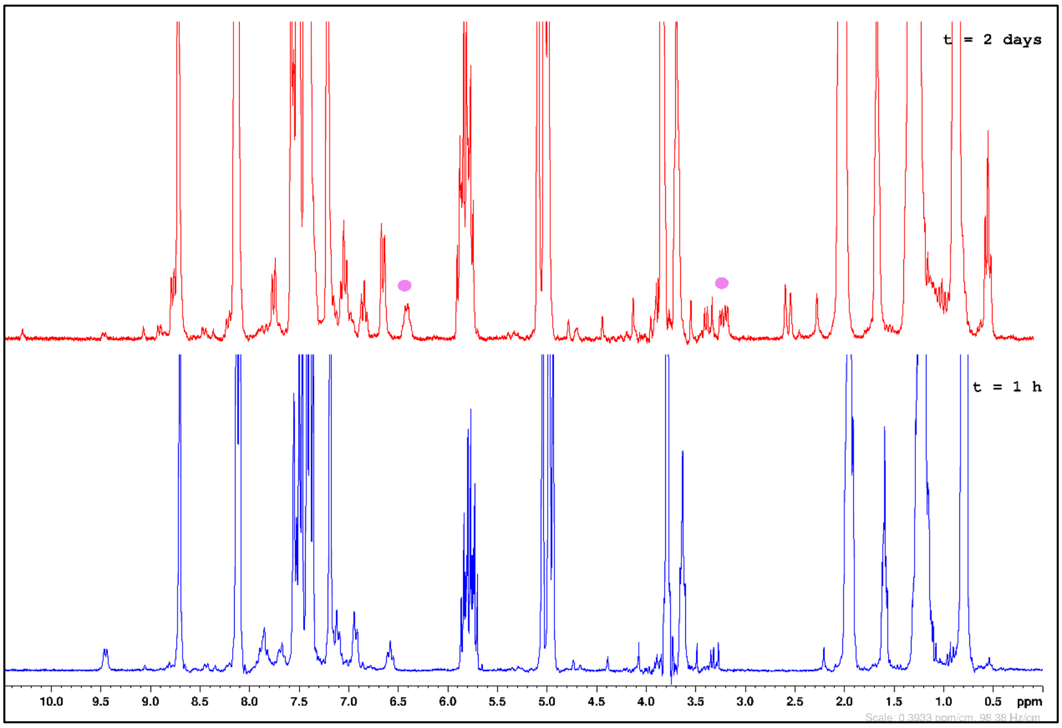


**Figure S46**: $^{1}H$ NMR spectrum for competitive reaction study of methyl benzoate and 1-hexene with **1-Ph** in pyridine-*d*5.; blue spectrum (below)- 30 minutes after addition of methyl benzoate (9.6 µL, 20 eq.), 1-hexene (9.9 µL, 20 eq.) to **1-Ph** (5 mg, 1 eq.) in 0.5 mL pyridine-*d*5, red spectrum – after heating the reaction mixture to 60 °C for 2 days. Pink circle denotes the characteristic peak for 1-hexene insertion product.

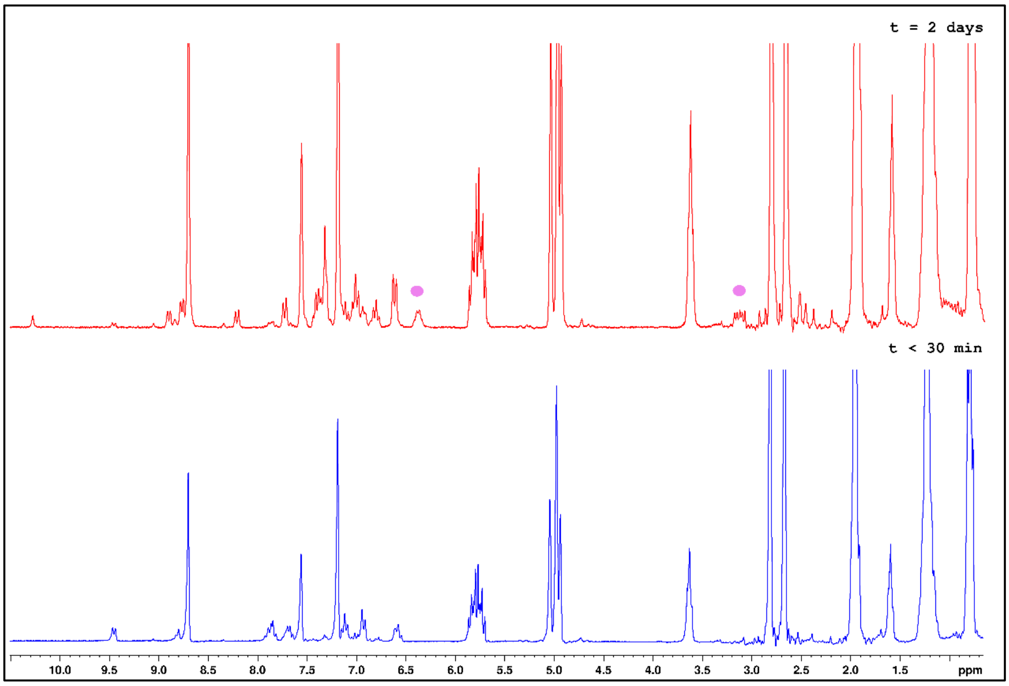


**Figure S47**: $^{1}$H NMR spectrum for competitive reaction study of N, N-dimethylacetamide and 1- hexene with **1-Ph**.; blue spectrum (below)- less than 30 minutes after addition of *N*,*N*-dimethylacetamide (7.3 µL, 20 eq.), 1-hexene (9.9 µL, 20 eq.) to **1-Ph** (5 mg, 1 eq.) in 0.5 mL pyridine-*d*5, red spectrum- after heating the reaction mixture to 60 °C for 3 days. Pink circle denotes the characteristic peak for 1-hexene insertion product.

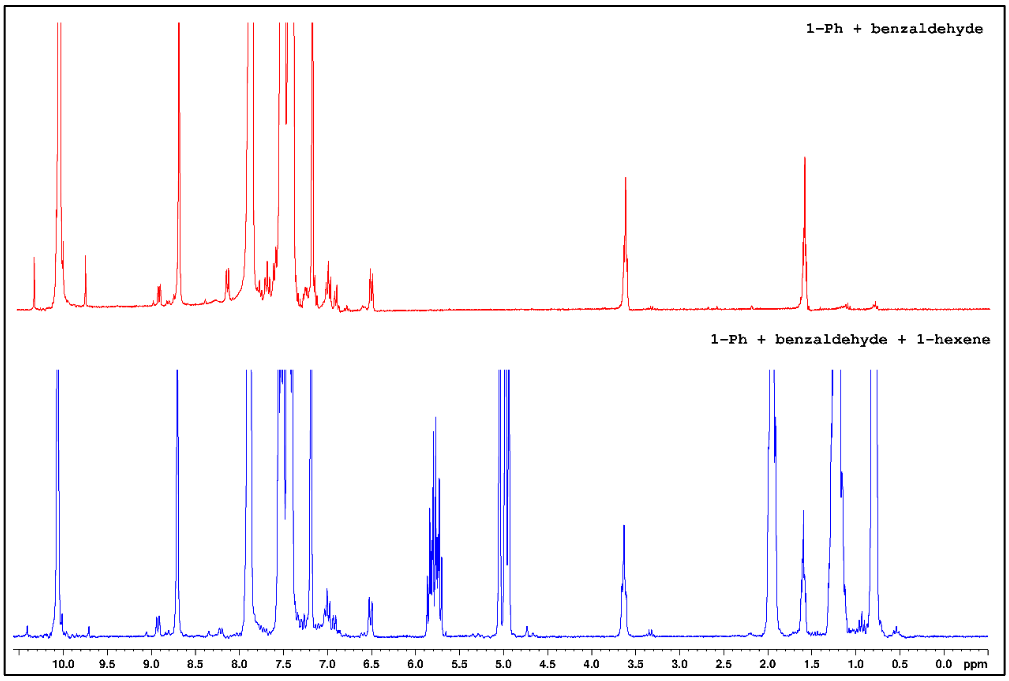


**Figure S48**: $^{1}$H NMR spectrum for competitive reaction study of benzaldehyde and 1-hexene with **1-Ph** in pyridine-*d*5; blue spectrum (below)- recorded immediately after the addition of benzaldehyde (12.8 µL, 20 eq.) to a pyridine-d5 solution of **1-Ph** (5 mg, 1 eq.) and 1-hexene (9.9 µL, 20 eq.). A new product formation is seen immediately as indicated by a change of color from red to colorless. The new signals formed do not match hexene inserted compound **3-Ph**. Red spectrum – separate reaction of **1-Ph** with benzaldehyde. The peaks match to that observed in blue spectrum, indicating benzaldehyde reacts with **1-Ph**.

## DFT calculations

All DFT calculations were performed with the Amsterdam Density Functional (ADF) program[88,89] using relativistic, dispersion-corrected density functional theory (DFT) at the ZORA-BLYPD3BJ/TZP level of theory for geometry optimizations and energy calculations, with the full electron model for all atoms (no frozen core).[88–97] Solvation in pyridine was simulated by using the conductor-like screening model (COSMO). All stationary points were verified to be minima on the potential energy surface through vibrational analysis. A concentration correction has been added to Gibbs energies (1.894 kcal mol$^{-1}$ for all computed systems, and 3.386 kcal mol$^{-1}$ for pyridine).[98,99] Voronoi deformation density (VDD) charges have also been computed at the same level of theory.[100]

## Mechanistic considerations

**Unfavored mechanism with mononuclear I-3 as the key intermediate.** In initial mechanistic considerations, splitting the dinuclear starting material **1-Ph** into mononuclear subunits (which appear as **I-3** in the mechanism discussed in the main part) was considered in concerted and coordination-insertion pathways. Among these two possibilities, the concerted pathway was favored by an energy difference of 5.1 kcal·mol$^{-1}$ (in the case of ethylene insertion), when comparing the Gibbs energies of the key transition states. However, the results obtained in the more detailed analyses of the concerted pathway were not in good agreement with important details of our experimental findings. Specifically, the reaction of "**1-Ph** + py → 2 **I-3**" is endergonic by $\Delta G$ = +14.6 kcal·mol$^{-1}$, i.e. $\Delta G$ = +7.3 kcal·mol$^{-1}$, when normalized to one equivalent of **I-3**. Obviously, this reaction is independent of the concentration of the olefin, which is not involved. This contrasts with to the formation of intermediate **I-1**, which will benefit from higher concentrations of the olefin. Experimentally, it has been shown that an excess of the olefins is necessary to drive the reactions to success in terms of sufficient spectroscopic and isolated yields. In the unfavored mechanism with only mononuclear key species, **I-3** would react through a concerted transition state with the olefin, which is related to an additional energy barrier of +15.6 kcal·mol$^{-1}$ relative to **I-3** (with ethylene as the substrate). It has to be stressed at this point that the concentration of **I-3** generated from **1-Ph** will be very low compared to that of **I-1** under the actual experimental conditions (**I-1** is thermodynamically favored compared to **I-3**, even more so in the presence of excess olefin, *vide supra*). Furthermore, the chemoselectivity of the olefin insertion reactions has been investigated with the mechanism starting from **I-3**. For example, the reaction pathways for the substrates 1-hexene and cyclopentene show very similar energy profiles (differing by only 0.5 kcal·mol$^{-1}$ in the key transition state). This is in contrast to experimental findings, which led to the isolation of the 1-hexene insertion product **3-Ph**, but only led to compound A (the product of a side reaction) in the case of cyclopentene. It was thus overall concluded that the mechanisms starting from **I-3** with mononuclear key intermediates and transition states do not offer a proper explanation of the experimental findings.

**Suggested mechanism involving dinuclear key species.** Thus, more demanding analyses with dinuclear species were conducted (as presented in the main part), based on the fact that dimerization of **I-3** is favorable by Δ$G$ = –14.6 kcal mol$^{-1}$ (2·**I-3** → **1-Ph-py** + **py**). The following olefins were considered in the final mechanism: ethylene, 1-hexene, styrene, *iso*-butene, cyclopentene, 1,3-cyclohexadiene, *neo*-hexene (see figure and table below).

The mechanism is in agreement with an excess of the olefin being necessary for **I1** being generated in sufficient amounts and in most cases also to facilitate the formation of the final product from a thermodynamic point of view. The inability of internal olefins is due to the high energy associated with **I-2** in these cases, while *neo*-hexene (a terminal olefin with a sterically demanding substituent) faced a high kinetic barrier of Δ$G$ (**I-1**→**TS-1**) = 28.9 kcal mol$^{-1}$ due to the relative stability of **I-1** and the high energy associated with **TS-1** in this case.

The mechanism shown in Figure S49 generates one equivalent of the desired product and one equivalent of the intermediate **I-3** from the dinuclear starting material **1-Ph-py**. The high-energy-intermediate **I-3** can be transformed into **1-Ph-py** (with elimination of pyridine) in an exothermic and exergonic reaction: **I3** → 0.5 **1-Ph-py** + 0.5 py (Δ$H$ = –10.6 kcal mol$^{-1}$; Δ$G$ = –7.3 kcal mol$^{-1}$) and thus be fed back into the synthetic cycle.

It is important to note that the overall reaction "**1-Ph-py** + py + 2 equiv. olefin → 2 equiv. product" is thermodynamically favorable in the majority of the cases (see last entry in table below). In the case of styrene as a substrate, the reaction is slightly endergonic, but could be driven to the product side by using an excess of the olefin in the experimental approach. Only in the case of *neo*-hexene as the substrate, the reaction is significantly endergonic.

The polar nature of the transition state (see main part) is in agreement with the polar solvent pyridine giving substantially higher yields than less polar solvents (e.g.: only small amounts (<5%) of **3-Ph** could be obtained from a reaction of **1-Ph** with 100 equiv. 1-hexene in THF at 60-70°C for 18 h).

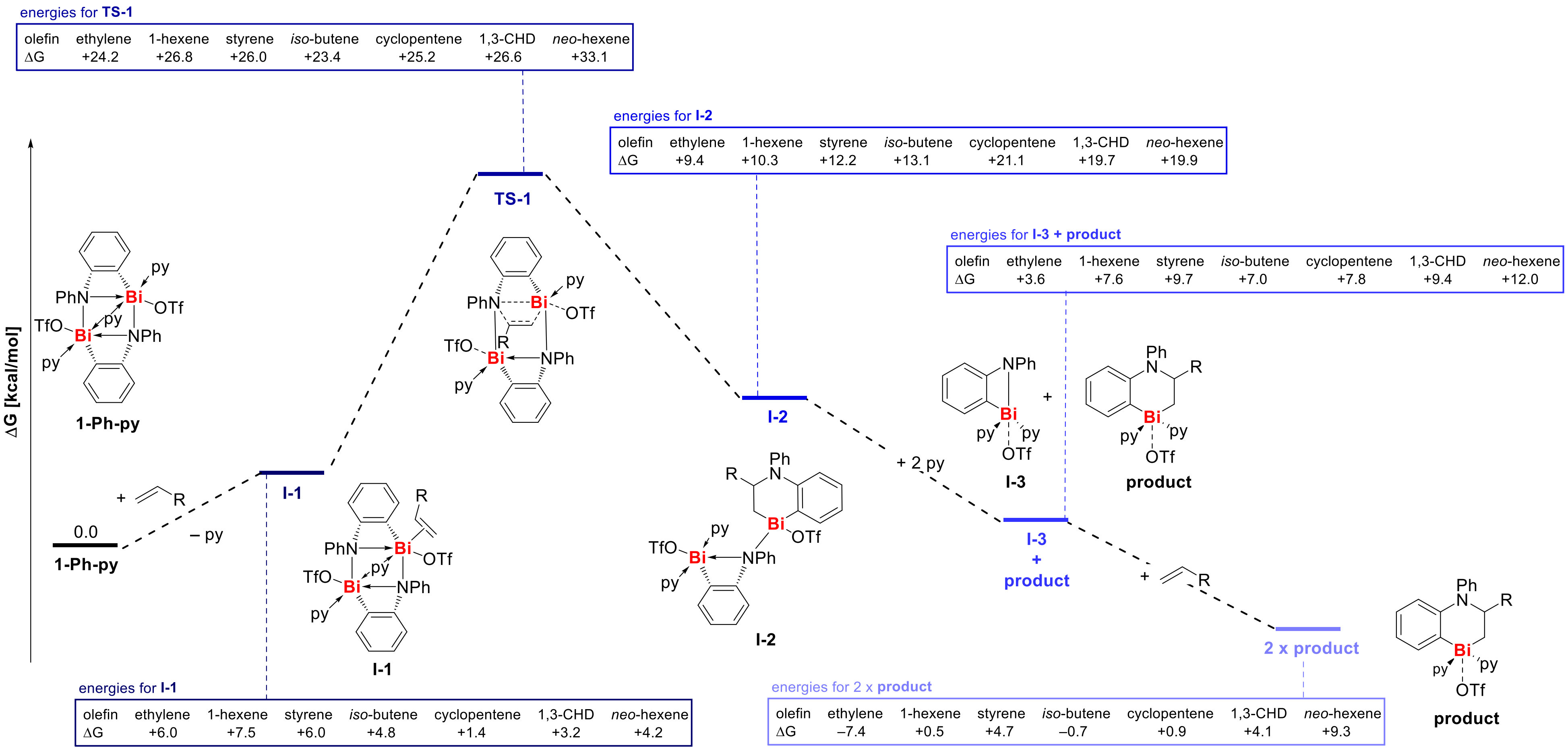


energies for **TS-1**

| olefin | ethylene | 1-hexene | styrene | *iso*-butene | cyclopentene | 1,3-CHD | *neo*-hexene |
|---|---|---|---|---|---|---|---|
| ΔG | +24.2 | +26.8 | +26.0 | +23.4 | +25.2 | +26.6 | +33.1 |

energies for **I-2**

| olefin | ethylene | 1-hexene | styrene | *iso*-butene | cyclopentene | 1,3-CHD | *neo*-hexene |
|---|---|---|---|---|---|---|---|
| ΔG | +9.4 | +10.3 | +12.2 | +13.1 | +21.1 | +19.7 | +19.9 |

energies for **I-3 + product**

| olefin | ethylene | 1-hexene | styrene | *iso*-butene | cyclopentene | 1,3-CHD | *neo*-hexene |
|---|---|---|---|---|---|---|---|
| ΔG | +3.6 | +7.6 | +9.7 | +7.0 | +7.8 | +9.4 | +12.0 |

energies for **I-1**

| olefin | ethylene | 1-hexene | styrene | *iso*-butene | cyclopentene | 1,3-CHD | *neo*-hexene |
|---|---|---|---|---|---|---|---|
| ΔG | +6.0 | +7.5 | +6.0 | +4.8 | +1.4 | +3.2 | +4.2 |

energies for 2 x **product**

| olefin | ethylene | 1-hexene | styrene | *iso*-butene | cyclopentene | 1,3-CHD | *neo*-hexene |
|---|---|---|---|---|---|---|---|
| ΔG | –7.4 | +0.5 | +4.7 | –0.7 | +0.9 | +4.1 | +9.3 |

**Figure S49.** Proposed mechanism for olefin insertion into Bi–N bond of **1-Ph-py**. Computed at ZORA-BLYP-D3(BJ)/TZP with a pyridine solvent model. A concentration correction has been performed (see general information in DFT calculations).

**Table S5**: Calculated enthalpies **Δ*H*** and Gibbs energies **Δ*G*** with respect to reactant **1-Ph-py** in the proposed mechanism for various olefins. All values are shown in kcal mol$^{-1}$. Computed at ZORA-BLYP-D3(BJ)/TZP in pyridine. A concentration correction has been performed (see general information in DFT calculations).

| Olefin | ***ethylene*** | | ***1-hexene*** | | ***styrene*** | | ***iso-butene*** | | ***cyclopentene*** | | ***1,3-CHD***[a] | | ***neo-hexene*** | |
|---|---|---|---|---|---|---|---|---|---|---|---|---|---|---|
| | **Δ*H*** | **Δ*G*** | **Δ*H*** | **Δ*G*** | **Δ*H*** | **Δ*G*** | **Δ*H*** | **Δ*G*** | **Δ*H*** | **Δ*G*** | **Δ*H*** | **Δ*G*** | **Δ*H*** | **Δ*G*** |
| **I-1** | +10.5 | +6.0 | +5.4 | +7.5 | +2.9 | +6.0 | +6.7 | +4.8 | +7.0 | +1.4 | +5.0 | +3.2 | +7.4 | +4.2 |
| **TS-1** | +23.2 | +24.2 | +21.4 | +26.8 | +19.4 | +26.0 | +25.0 | +23.4 | +22.6 | +25.2 | +23.9 | +26.6 | +33.2 | +33.1 |
| **I-2** | +7.3 | +9.4 | +8.6 | +10.3 | +7.3 | +12.2 | +9.1 | +13.1 | +15.8 | +21.1 | +16.0 | +19.7 | +15.2 | +19.9 |
| **product + I-3** | +0.8 | +3.6 | +0.7 | +7.6 | +4.3 | +9.7 | +1.5 | +7.0 | +4.2 | +7.8 | +6.0 | +9.4 | +6.9 | +12.0 |
| **2*product** | –19.6 | –7.4 | –19.8 | +0.5 | –12.5 | +4.7 | –18.1 | –0.7 | -12.7 | +0.9 | –9.2 | +4.1 | –7.4 | +9.3 |

For the following olefins, the products could be synthesized: ethylene (**2-Ph**), 1-hexene (**3-Ph**), styrene (**6-Ph**). For the remaining olefins, the synthesis was attempted, but unsuccessful to date. a: 1,3-CHD = 1,3-cyclohexadiene.

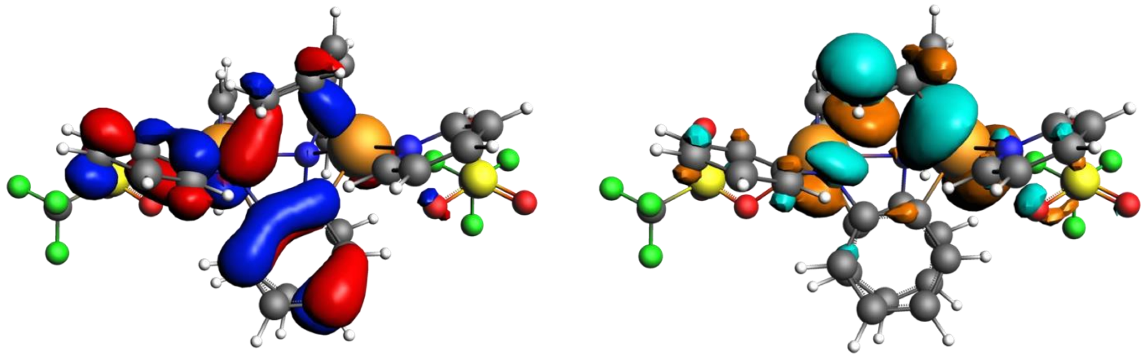

**Figure S50.** HOMO (left) and LUMO (right) of **TS-1** with R = H; isovalue = 0.03.

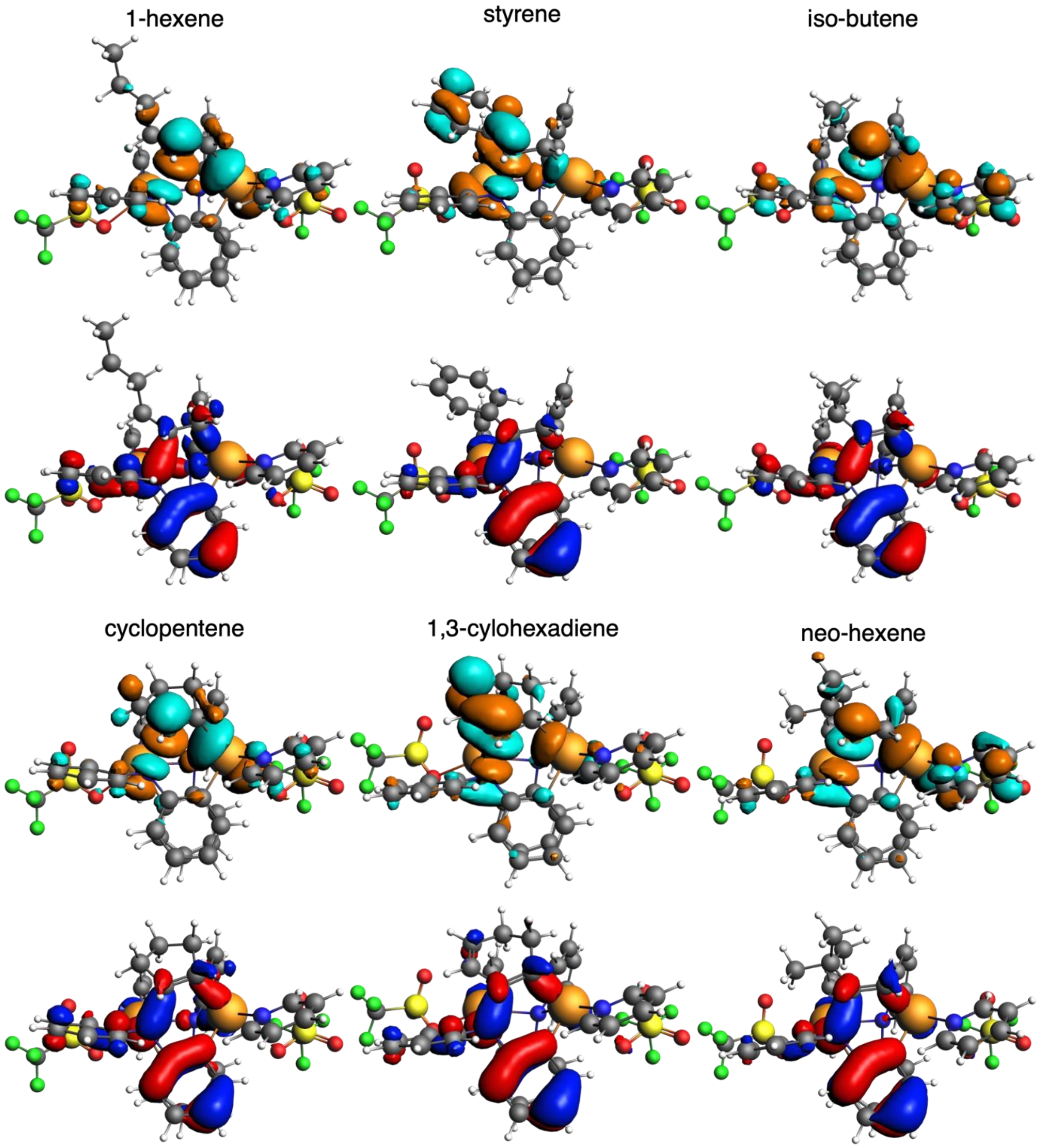


**Figure S51.** Frontier orbitals of **TS-1** with the olefins covered in this study (except for ethylene, *cf*. Figure S48). The HOMOs are shown red/blue, the LUMOs are shown in orange/turquoise; isovalue = 0.03.

**Table S6.** Voronoi deformation density (VDD) charges [au] for the atoms forming the $NC_2Bi$ four-membered ring in **TS-1**, which is highlighted by the grey box in the Lewis drawing below. a: 1,3-CHD = 1,3-cyclohexadiene.

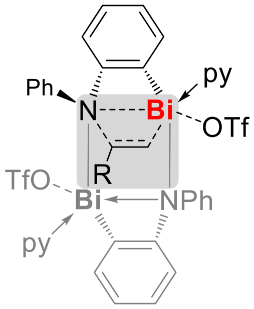


**TS-1**

| | *ethylene* | *1-hexene* | *styrene* | *iso-butene* | *cyclopentene* | *1,3-CHD*[a] | *neo-hexene* |
|---|---|---|---|---|---|---|---|
| **Bi** | +0.413 | +0.398 | +0.370 | +0.388 | +0.385 | +0.355 | +0.379 |
| **N** | –0.152 | –0.137 | –0.107 | –0.128 | –0.137 | –0.112 | –0.128 |
| **CHR** | +0.020 | +0.044 | +0.034 | +0.047 | +0.042 | +0.036 | +0.047 |
| **$CH_2$** | –0.136 | –0.150 | –0.157 | –0.153 | –0.134 | –0.139 | –0.152 |

A brief analysis of the VDD charges of the two carbon atoms, the nitrogen atom and the bismuth atom that form the four-membered ring in **TS-1** has been presented in the main part for ethylene as the olefinic substrate (R = H). The same qualitative result is obtained for all olefinic substrates covered in this work. Thus, the polarization of the olefin by the polar $RBi^+–NR_2$ functional group is a key feature in all cases. It has to be stressed that the polar nature of the $RBi^+–NR_2$ bond in the starting material **1-Ph-py**, but also in the intermediate **I-1** is decisively increased by the cationic nature of the bismuth center.

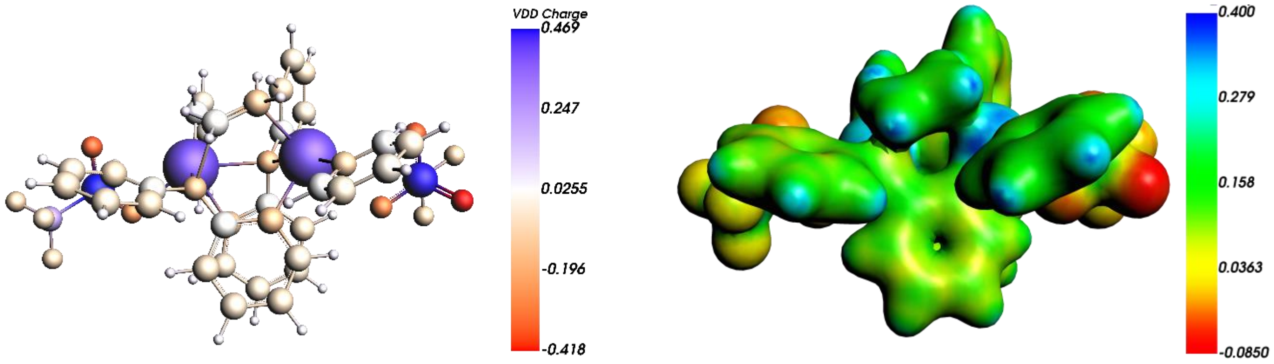


**Figure S52.** Map of the VDD charges (left) and isosurface map of the molecular electrostatic potential (right, isovalue = 0.03) for **TS-1** with ethylene as the substrate (i.e. R = H).

**Table S7.** Cartesian coordinates (in Å) and ADF electronic energies (in kcal mol$^{-1}$) of all systems under analysis. Computed at ZORA-BLYP-D3(BJ)/TZ2P level of theory in pyridine.

**1-Ph-py** (-13420.2)

| | | | | |
|---|---|---|---|---|
| 1 | Bi | 1.69540003 | -0.42719277 | 0.09022753 |
| 2 | S | 4.87244988 | 0.64087092 | -0.70637156 |
| 3 | N | -0.73692493 | -1.44830110 | -0.11140941 |
| 4 | H | 4.99286002 | -6.01744112 | 0.61181017 |
| 5 | O | 3.54332143 | 0.40663783 | -1.39396673 |
| 6 | C | -0.38347459 | -1.75851499 | -1.47552930 |
| 7 | C | 0.85874585 | -1.25122186 | -1.87514424 |
| 8 | O | 4.82455769 | 0.39061510 | 0.74912431 |
| 9 | O | 6.02023763 | 0.09702346 | -1.45171518 |
| 10 | C | -1.16328250 | -2.53816701 | 0.71242461 |
| 11 | F | 4.02741356 | 3.16180666 | -0.24473680 |
| 12 | C | -0.54385396 | -3.80231657 | 0.59337871 |
| 13 | H | 0.21918217 | -3.95140737 | -0.16446265 |
| 14 | F | 6.22757145 | 2.93215262 | -0.28855535 |
| 15 | C | -1.21241892 | -2.42412848 | -2.39102958 |
| 16 | H | -2.17988297 | -2.81298412 | -2.08059033 |
| 17 | C | 1.28693070 | -1.36178294 | -3.19837180 |
| 18 | H | 2.24601646 | -0.94944961 | -3.50654025 |
| 19 | C | 0.46061749 | -2.01850933 | -4.12425161 |
| 20 | H | 0.77489501 | -2.11498939 | -5.16252823 |
| 21 | H | -2.19041645 | 5.58607018 | 2.73312197 |
| 22 | H | -2.92895455 | 6.06442295 | -1.50487084 |
| 23 | N | 2.98116791 | -2.72251040 | 0.23650821 |
| 24 | C | -2.15346268 | -2.37570623 | 1.69780936 |
| 25 | H | -2.67891835 | -1.43139229 | 1.80625125 |
| 26 | C | -0.89607817 | -4.85528838 | 1.44029853 |
| 27 | H | -0.39726285 | -5.81759010 | 1.33175274 |
| 28 | C | -2.50158647 | -3.43056733 | 2.54572714 |
| 29 | H | -3.26972675 | -3.27264473 | 3.30059177 |
| 30 | C | -0.77426429 | -2.55165550 | -3.71548313 |
| 31 | H | -1.40873173 | -3.05767762 | -4.44192699 |
| 32 | C | -1.87622052 | -4.67707907 | 2.42769576 |
| 33 | H | -2.14736745 | -5.49632350 | 3.09100144 |
| 34 | C | 3.79306400 | -2.91437736 | 1.29996812 |
| 35 | C | 2.89219795 | -3.69028098 | -0.70168082 |
| 36 | C | 4.53055631 | -4.08597322 | 1.47282806 |
| 37 | C | 5.05845555 | 2.52596912 | -0.86311659 |
| 38 | C | 3.59968109 | -4.88889383 | -0.60183009 |
| 39 | F | 5.07098016 | 2.89774536 | -2.17657277 |
| 40 | Bi | -1.94644161 | 0.68534592 | 0.08158177 |
| 41 | S | -5.24186811 | -0.37724763 | -0.34241876 |
| 42 | N | 0.38660680 | 1.64419583 | -0.28561137 |
| 43 | H | -2.61769054 | 7.09995201 | 0.76755760 |
| 44 | O | -4.01840144 | -0.00309965 | -1.15613822 |
| 45 | C | 0.04259536 | 1.77552678 | -1.68331396 |
| 46 | C | -1.21650336 | 1.24291786 | -1.98922195 |
| 47 | O | -4.98879930 | -0.38527383 | 1.11272344 |
| 48 | O | -6.47752205 | 0.27032248 | -0.81427355 |
| 49 | C | 0.83618785 | 2.79537257 | 0.42916580 |

| | | | | |
|---|---|---|---|---|
| 50 | F | -4.34677129 | -2.92337168 | -0.52175529 |
| 51 | C | 0.71761501 | 4.10077166 | -0.08962194 |
| 52 | H | 0.30284398 | 4.25118292 | -1.08058965 |
| 53 | F | -6.51287598 | -2.73952202 | -0.11388441 |
| 54 | C | 0.87750472 | 2.25508194 | -2.70124819 |
| 55 | H | 1.86685619 | 2.64379409 | -2.47230157 |
| 56 | C | -1.66791342 | 1.15132024 | -3.30344730 |
| 57 | H | -2.63701950 | 0.71256544 | -3.53179468 |
| 58 | C | -0.83807001 | 1.63419008 | -4.32834097 |
| 59 | H | -1.16475708 | 1.57382587 | -5.36531010 |
| 60 | H | -0.55863583 | 1.08599238 | 3.01775871 |
| 61 | H | 1.45937720 | -2.53287344 | 2.82568577 |
| 62 | H | 0.29313897 | -3.39228838 | 4.84970038 |
| 63 | C | 1.36181567 | 2.63660000 | 1.72845328 |
| 64 | H | 1.47237344 | 1.64133401 | 2.15164912 |
| 65 | C | 1.10775058 | 5.20524551 | 0.67216378 |
| 66 | H | 0.99322925 | 6.20397216 | 0.25351553 |
| 67 | C | 1.74844978 | 3.74536648 | 2.48516660 |
| 68 | H | 2.15129970 | 3.59325015 | 3.48539112 |
| 69 | C | 0.41898107 | 2.18295566 | -4.02321645 |
| 70 | H | 1.05815372 | 2.54115902 | -4.82877141 |
| 71 | C | 1.62441758 | 5.04042792 | 1.96369054 |
| 72 | H | 1.92308831 | 5.90444275 | 2.55429897 |
| 73 | H | -1.84824060 | 0.36262554 | 5.02491847 |
| 74 | N | -2.45114703 | 3.25423967 | 0.30355912 |
| 75 | C | -2.67110620 | 4.06225324 | -0.75558863 |
| 76 | C | -5.46755699 | -2.20736830 | -0.81019284 |
| 77 | C | -2.28841660 | 3.80696853 | 1.52442712 |
| 78 | C | -2.74645995 | 5.44962885 | -0.62698139 |
| 79 | C | -2.33853248 | 5.18427381 | 1.73423979 |
| 80 | C | -2.57423520 | 6.02072689 | 0.63848379 |
| 81 | H | -2.77396292 | 3.57255949 | -1.72136999 |
| 82 | H | -2.10602510 | 3.12081760 | 2.35006962 |
| 83 | F | -5.72783811 | -2.32960894 | -2.14323715 |
| 84 | C | 4.43006384 | -5.09263679 | 0.50589080 |
| 85 | H | 3.85355004 | -2.09798329 | 2.01738420 |
| 86 | H | 2.23957347 | -3.47858259 | -1.54575935 |
| 87 | H | 3.49975268 | -5.64158825 | -1.37985829 |
| 88 | H | 5.17003258 | -4.19922290 | 2.34461487 |
| 89 | H | -1.40709963 | -1.92362358 | 5.98667888 |
| 90 | N | 0.50837249 | -0.68579110 | 2.82829489 |
| 91 | C | -0.40641755 | 0.09371968 | 3.44249191 |
| 92 | C | 0.73376697 | -1.91141267 | 3.34681444 |
| 93 | C | -1.11950499 | -0.30734635 | 4.57450224 |
| 94 | C | 0.07565578 | -2.39337650 | 4.48050603 |
| 95 | C | -0.86964711 | -1.57646708 | 5.10688880 |

**I-3** (-7485.4)

| | | | | |
|---|---|---|---|---|
| 1 | C | 4.98750506 | -1.53975024 | -0.24465503 |
| 2 | C | 1.31547482 | 3.62183110 | 2.33725239 |
| 3 | H | 6.01141905 | -1.57040869 | 0.12693510 |
| 4 | C | 0.35326017 | 1.43569530 | 1.87750037 |
| 5 | H | 1.23493548 | 4.61532538 | 2.77509528 |

| | | | | |
|---|---|---|---|---|
| 6 | Bi | 0.16138246 | -0.88663250 | -1.16086422 |
| 7 | S | -3.29445489 | 0.27438753 | -0.21684014 |
| 8 | N | 1.54635016 | -0.24120823 | 0.60218713 |
| 9 | C | -3.73871725 | 1.85074319 | 0.75151209 |
| 10 | O | -2.18990238 | -0.31487395 | 0.59148003 |
| 11 | C | 0.25349951 | 2.70902270 | 2.43936730 |
| 12 | C | 2.70288341 | -0.79457316 | -0.00834476 |
| 13 | C | 2.38065951 | -1.47882496 | -1.20357075 |
| 14 | H | -0.66257392 | 2.99398899 | 2.95484711 |
| 15 | O | -2.86603652 | 0.79681393 | -1.53834518 |
| 16 | O | -4.55396454 | -0.50256402 | -0.21032572 |
| 17 | C | 1.52202358 | 1.03850764 | 1.18553102 |
| 18 | F | -2.68015492 | 2.71479597 | 0.79075230 |
| 19 | C | 2.58174581 | 1.96876098 | 1.07529934 |
| 20 | H | 3.47510989 | 1.70663449 | 0.51734362 |
| 21 | H | -0.48251773 | 0.74236462 | 1.93159552 |
| 22 | C | 2.47526938 | 3.23886567 | 1.64994639 |
| 23 | H | 3.30099906 | 3.94145531 | 1.54094503 |
| 24 | F | -4.79085363 | 2.49796663 | 0.16457363 |
| 25 | C | 4.02370594 | -0.82067844 | 0.48009050 |
| 26 | H | 4.29587868 | -0.31425969 | 1.40345644 |
| 27 | C | 3.33918340 | -2.19505275 | -1.91258751 |
| 28 | H | 3.08290339 | -2.72169884 | -2.83197226 |
| 29 | C | 4.66302394 | -2.22278891 | -1.42869367 |
| 30 | H | 5.43289414 | -2.77103294 | -1.97000641 |
| 31 | F | -4.08884855 | 1.55174846 | 2.03891131 |
| 32 | H | -2.27637193 | -2.89634677 | -0.24698554 |
| 33 | H | -0.87048356 | 2.20345240 | -1.09278444 |
| 34 | N | -0.25201304 | -2.95421377 | 0.22580064 |
| 35 | C | 0.73481368 | -3.56162355 | 0.92202620 |
| 36 | C | 0.49103185 | -4.67205050 | 1.72939254 |
| 37 | C | -0.81313783 | -5.17150485 | 1.81835046 |
| 38 | C | -1.83176830 | -4.54055804 | 1.09663342 |
| 39 | C | -1.51405103 | -3.43158292 | 0.31283673 |
| 40 | H | 1.72945279 | -3.13695030 | 0.81683903 |
| 41 | H | 1.31151086 | -5.13054397 | 2.27566366 |
| 42 | H | -2.85947489 | -4.89238930 | 1.13988877 |
| 43 | N | 0.89139365 | 1.44120544 | -1.89046531 |
| 44 | C | 2.12963941 | 1.70655968 | -2.36520384 |
| 45 | C | 2.62935964 | 3.00616731 | -2.44038373 |
| 46 | C | 1.83290599 | 4.06535250 | -1.99177483 |
| 47 | C | 0.55764569 | 3.78832858 | -1.49129822 |
| 48 | C | 0.11787251 | 2.46567233 | -1.46048470 |
| 49 | H | 2.72195891 | 0.84840606 | -2.67220606 |
| 50 | H | 3.62857727 | 3.17661172 | -2.83346807 |
| 51 | H | -0.09043057 | 4.57917108 | -1.12285252 |
| 52 | H | 2.20337975 | 5.08755549 | -2.02591310 |
| 53 | H | -1.03208733 | -6.03589787 | 2.44150447 |

**Pyridine** (-1570.9)

| | | | | |
|---|---|---|---|---|
| 1 | C | -0.95542488 | 0.72478272 | 0.00000000 |
| 2 | H | 0.51471559 | 2.32552726 | 0.00000000 |
| 3 | C | -1.11283487 | -0.66565751 | 0.00000000 |

| | | | | |
|---|---|---|---|---|
| 4 | N | 1.29135225 | -0.97965712 | 0.00000000 |
| 5 | H | -0.06036216 | -2.55835527 | 0.00000000 |
| 6 | C | 1.42561963 | 0.36590816 | 0.00000000 |
| 7 | H | -2.10085483 | -1.12227853 | 0.00000000 |
| 8 | C | 0.03175450 | -1.47152371 | 0.00000000 |
| 9 | H | -1.82258564 | 1.38275015 | 0.00000000 |
| 10 | H | 2.44742955 | 0.74749816 | 0.00000000 |
| 11 | C | 0.34119086 | 1.25100569 | 0.00000000 |

**Ethylene** (-709.4)

| | | | | |
|---|---|---|---|---|
| 1 | C | -0.66862755 | -0.00000000 | 0.00000000 |
| 2 | C | 0.66862755 | 0.00000000 | 0.00000000 |
| 3 | H | -1.23996442 | -0.92912326 | 0.00000000 |
| 4 | H | -1.23996442 | 0.92912326 | 0.00000000 |
| 5 | H | 1.23996442 | -0.92912326 | 0.00000000 |
| 6 | H | 1.23996442 | 0.92912326 | 0.00000000 |

**I-1** (-12549.3)

| | | | | |
|---|---|---|---|---|
| 1 | Bi | 2.04379045 | -0.68567087 | 0.04305350 |
| 2 | S | 5.31123608 | 0.30561214 | -0.42584600 |
| 3 | N | -0.38562537 | -1.58074902 | -0.27916933 |
| 4 | H | 2.60859442 | -7.13598958 | 0.50671044 |
| 5 | O | 4.06201944 | -0.04109261 | -1.22278655 |
| 6 | C | -0.06267500 | -1.67531774 | -1.69193599 |
| 7 | C | 1.20780819 | -1.17910131 | -2.00503221 |
| 8 | O | 5.07630642 | 0.30130989 | 1.03195469 |
| 9 | O | 6.52191910 | -0.36008437 | -0.92957386 |
| 10 | C | -0.84788897 | -2.75873281 | 0.39630127 |
| 11 | F | 4.44886417 | 2.86245559 | -0.54707550 |
| 12 | C | -0.70224607 | -4.04077130 | -0.16910437 |
| 13 | H | -0.27551161 | -4.14954125 | -1.15995717 |
| 14 | F | 6.62357053 | 2.64235796 | -0.20809278 |
| 15 | C | -0.93142037 | -2.10507404 | -2.70261343 |
| 16 | H | -1.92793937 | -2.46563081 | -2.46169631 |
| 17 | C | 1.63783212 | -1.07580009 | -3.32670264 |
| 18 | H | 2.61778084 | -0.66741313 | -3.56445115 |
| 19 | C | 0.77387825 | -1.50641815 | -4.34563596 |
| 20 | H | 1.08511214 | -1.43462666 | -5.38652063 |
| 21 | H | 2.22452927 | -3.20684050 | 2.24106388 |
| 22 | H | 2.72438152 | -3.52963530 | -1.86736060 |
| 23 | N | 2.48232017 | -3.27463813 | 0.17829854 |
| 24 | C | -1.39181200 | -2.65173377 | 1.69112156 |
| 25 | H | -1.51502690 | -1.67490292 | 2.15239088 |
| 26 | C | -1.08403263 | -5.17784524 | 0.54795532 |
| 27 | H | -0.95080890 | -6.15841539 | 0.09406507 |
| 28 | C | -1.77194133 | -3.79291992 | 2.40175150 |
| 29 | H | -2.19053172 | -3.68222995 | 3.40071411 |
| 30 | C | -0.49509452 | -2.02080668 | -4.03063281 |
| 31 | H | -1.15934704 | -2.34138747 | -4.83139962 |
| 32 | C | -1.62008576 | -5.06622603 | 1.83673867 |
| 33 | H | -1.91457575 | -5.95488472 | 2.39168090 |
| 34 | C | 2.36028863 | -3.86646171 | 1.38564157 |
| 35 | C | 2.65091619 | -4.04953893 | -0.91491935 |

| | | | | |
|---|---|---|---|---|
| 36 | C | 2.39689852 | -5.25058553 | 1.54709737 |
| 37 | C | 5.55165495 | 2.13684994 | -0.87999322 |
| 38 | C | 2.71017248 | -5.44115590 | -0.83569264 |
| 39 | F | 5.77192492 | 2.27033007 | -2.21748495 |
| 40 | Bi | -1.56435923 | 0.43770907 | 0.17493675 |
| 41 | S | -4.76089427 | -0.61484797 | -0.56475223 |
| 42 | N | 0.82981905 | 1.44159332 | -0.06371353 |
| 43 | H | 2.85008667 | -6.02808722 | -1.73998490 |
| 44 | O | -3.41929982 | -0.42076960 | -1.24665985 |
| 45 | C | 0.44032943 | 1.81831597 | -1.40425527 |
| 46 | C | -0.82060529 | 1.34610568 | -1.78585324 |
| 47 | O | -4.69269486 | -0.43660453 | 0.89965824 |
| 48 | O | -5.87320497 | 0.03301318 | -1.27850826 |
| 49 | C | 1.22349725 | 2.50504946 | 0.81313739 |
| 50 | F | -4.06536642 | -3.20178344 | -0.23138521 |
| 51 | C | 0.49718598 | 3.71675128 | 0.81555070 |
| 52 | H | -0.31411874 | 3.85130384 | 0.10418918 |
| 53 | F | -6.24721660 | -2.83969485 | -0.27464696 |
| 54 | C | 1.25071472 | 2.51410822 | -2.31230583 |
| 55 | H | 2.23256125 | 2.87669460 | -2.01545853 |
| 56 | C | -1.29170573 | 1.52297940 | -3.08659626 |
| 57 | H | -2.26599919 | 1.13798621 | -3.38211658 |
| 58 | C | -0.48286000 | 2.20689349 | -4.00729747 |
| 59 | H | -0.82748967 | 2.35406158 | -5.02988675 |
| 60 | H | -1.26202242 | 2.20712815 | 3.06183747 |
| 61 | H | 0.77108134 | -1.40230184 | 2.88593959 |
| 62 | H | 2.20796213 | -0.79276894 | 4.83458810 |
| 63 | C | 2.27985489 | 2.35679317 | 1.72733586 |
| 64 | H | 2.88468663 | 1.45406727 | 1.73592634 |
| 65 | C | 0.81209461 | 4.73553615 | 1.71756277 |
| 66 | H | 0.23411085 | 5.65852246 | 1.70556397 |
| 67 | C | 2.58992272 | 3.37782579 | 2.63125980 |
| 68 | H | 3.41063048 | 3.23558449 | 3.33193611 |
| 69 | C | 0.77276633 | 2.70474179 | -3.61538001 |
| 70 | H | 1.39106650 | 3.23376380 | -4.33923693 |
| 71 | C | 1.85918944 | 4.57133012 | 2.63661132 |
| 72 | H | 2.10152440 | 5.36308233 | 3.34288833 |
| 73 | H | 0.03719424 | 2.94292372 | 5.05568248 |
| 74 | H | 2.28272944 | -5.68384837 | 2.53721144 |
| 75 | H | 1.82728053 | 1.42567232 | 5.96609852 |
| 76 | C | -5.05172805 | -2.47570769 | -0.82046948 |
| 77 | N | -0.29127368 | 0.37994880 | 2.87035461 |
| 78 | C | -0.49257530 | 1.56395816 | 3.48572347 |
| 79 | C | 0.66073184 | -0.43377308 | 3.37390688 |
| 80 | C | 0.23761848 | 1.97528959 | 4.60340449 |
| 81 | C | 1.44945804 | -0.10042895 | 4.47682635 |
| 82 | C | 1.23140936 | 1.13047203 | 5.10526696 |
| 83 | F | -5.07439890 | -2.77447518 | -2.15176504 |
| 84 | C | 2.57687071 | -6.05258398 | 0.41556472 |
| 85 | C | -3.39144332 | 3.02494051 | 0.90400734 |
| 86 | H | -2.70069083 | 3.58166435 | 1.53705416 |
| 87 | H | -4.10317785 | 2.36952761 | 1.40426727 |
| 88 | C | -3.37865715 | 3.16169426 | -0.43027375 |

| | | | | |
|---|---|---|---|---|
| 89 | H | -2.67367765 | 3.82098871 | -0.93304982 |
| 90 | H | -4.07298896 | 2.61494021 | -1.06694943 |

**TS-1** (-12535.8)

| | | | | |
|---|---|---|---|---|
| 1 | Bi | 1.84943181 | -0.38201249 | 0.20294549 |
| 2 | S | 5.28160685 | -0.53949333 | -0.33667626 |
| 3 | N | -0.40579013 | -1.39451074 | -0.01832690 |
| 4 | H | 3.31053024 | -6.62790874 | 0.50831984 |
| 5 | O | 4.03542210 | -0.17093415 | -1.11025274 |
| 6 | C | -0.18457557 | -1.47422385 | -1.45357370 |
| 7 | C | 1.02374537 | -0.88573020 | -1.84190816 |
| 8 | O | 5.14500846 | -0.30629664 | 1.11742048 |
| 9 | O | 5.89776827 | -1.80859718 | -0.75810982 |
| 10 | C | -0.61147093 | -2.60586943 | 0.71883245 |
| 11 | F | 6.05741170 | 2.03857396 | -0.63164022 |
| 12 | C | -0.56225997 | -3.88379836 | 0.13063269 |
| 13 | H | -0.38701386 | -3.98297884 | -0.93546421 |
| 14 | F | 7.71604198 | 0.60895507 | -0.31907584 |
| 15 | C | -1.08505062 | -1.95252051 | -2.41314400 |
| 16 | H | -2.03493655 | -2.39127316 | -2.12324751 |
| 17 | C | 1.36390880 | -0.73839904 | -3.18354869 |
| 18 | H | 2.30068568 | -0.26830438 | -3.47596237 |
| 19 | C | 0.46514747 | -1.21347085 | -4.15305961 |
| 20 | H | 0.70249868 | -1.10885045 | -5.21059026 |
| 21 | H | 2.83353143 | -5.28367234 | 2.58257837 |
| 22 | H | 0.84963588 | 1.90153863 | 2.31780479 |
| 23 | N | 2.59419026 | -2.82993211 | 0.27923851 |
| 24 | C | -0.80930903 | -2.51006219 | 2.11133103 |
| 25 | H | -0.81454264 | -1.53127879 | 2.58646994 |
| 26 | C | -0.71383881 | -5.02872740 | 0.91910452 |
| 27 | H | -0.66017208 | -6.00787078 | 0.44582305 |
| 28 | C | -0.96256348 | -3.65796251 | 2.89298757 |
| 29 | H | -1.10867884 | -3.55793137 | 3.96724298 |
| 30 | C | -0.74250297 | -1.81623451 | -3.76570079 |
| 31 | H | -1.43431463 | -2.17141589 | -4.52757938 |
| 32 | C | -0.91792327 | -4.92801750 | 2.30100181 |
| 33 | H | -1.03008692 | -5.82346318 | 2.90918633 |
| 34 | C | 2.59768150 | -3.45301042 | 1.47704866 |
| 35 | C | 2.84828884 | -3.54316934 | -0.83856847 |
| 36 | C | 2.84959622 | -4.81810178 | 1.60083083 |
| 37 | C | 6.50790908 | 0.78851785 | -0.92886020 |
| 38 | C | 3.11488027 | -4.91082892 | -0.79448475 |
| 39 | F | 6.68932593 | 0.70420674 | -2.27842015 |
| 40 | Bi | -2.26568681 | 0.25507875 | 0.33187043 |
| 41 | S | -5.31056732 | -1.33148583 | -0.93722057 |
| 42 | N | 0.89962602 | 1.95254229 | -0.03275020 |
| 43 | H | -0.26640778 | 3.19588395 | 1.65357665 |
| 44 | O | -4.13451909 | -0.65487781 | -1.57108658 |
| 45 | C | 0.07113592 | 2.09559188 | -1.18387973 |
| 46 | C | -1.22382879 | 1.52416032 | -1.26421990 |
| 47 | O | -5.33559028 | -1.23602863 | 0.54194887 |
| 48 | O | -6.59439313 | -1.10083396 | -1.63038527 |
| 49 | C | 1.81848870 | 3.02263986 | 0.22504085 |

| | | | | |
|---|---|---|---|---|
| 50 | F | -3.77384735 | -3.53340476 | -0.63708996 |
| 51 | C | 1.40452042 | 4.37022603 | 0.09762956 |
| 52 | H | 0.41297261 | 4.58890413 | -0.29039750 |
| 53 | F | -5.94345277 | -3.95460568 | -0.75140278 |
| 54 | C | 0.58886519 | 2.73917756 | -2.33705169 |
| 55 | H | 1.59846309 | 3.13922783 | -2.30575893 |
| 56 | C | -1.96135802 | 1.64104231 | -2.45819004 |
| 57 | H | -2.95229609 | 1.19759526 | -2.52278577 |
| 58 | C | -1.44259638 | 2.29519688 | -3.57696029 |
| 59 | H | -2.02780896 | 2.36121687 | -4.49247337 |
| 60 | C | -1.25342277 | 1.39571077 | 2.30396696 |
| 61 | H | -1.09220049 | 0.55427756 | 2.97616102 |
| 62 | H | -2.17482701 | 1.95149235 | 2.48114988 |
| 63 | C | 3.09980032 | 2.77354073 | 0.75117944 |
| 64 | H | 3.47180578 | 1.75970801 | 0.86973523 |
| 65 | C | 2.25150974 | 5.41939643 | 0.45686201 |
| 66 | H | 1.90736904 | 6.44638759 | 0.34372062 |
| 67 | C | 3.94447504 | 3.82849313 | 1.11895353 |
| 68 | H | 4.93330125 | 3.60232689 | 1.51292349 |
| 69 | C | -0.15091466 | 2.83542690 | -3.51161853 |
| 70 | H | 0.28916627 | 3.31954185 | -4.38223495 |
| 71 | C | 3.53058092 | 5.15638613 | 0.97115359 |
| 72 | H | 4.19093921 | 5.97467504 | 1.25236119 |
| 73 | C | -0.13157411 | 2.15699102 | 1.93221987 |
| 74 | F | -4.82020369 | -3.42504125 | -2.58391660 |
| 75 | C | 3.11263811 | -5.56018488 | 0.44494418 |
| 76 | C | -4.94229666 | -3.17030325 | -1.24699559 |
| 77 | H | 2.38066496 | -2.83761030 | 2.34755017 |
| 78 | H | 2.82709426 | -2.99258671 | -1.77532401 |
| 79 | H | 3.31614356 | -5.45132496 | -1.71591489 |
| 80 | N | -3.92678454 | 2.25267377 | 0.42422755 |
| 81 | C | -5.18185540 | 2.05351692 | 0.88706346 |
| 82 | C | -3.55168617 | 3.50004939 | 0.06242830 |
| 83 | C | -6.10324588 | 3.09570878 | 1.00041563 |
| 84 | H | -5.44040446 | 1.03188161 | 1.15423057 |
| 85 | C | -4.41495729 | 4.59143080 | 0.15159043 |
| 86 | H | -2.53802317 | 3.60338191 | -0.31772655 |
| 87 | C | -5.71481210 | 4.38652143 | 0.62812441 |
| 88 | H | -7.10418166 | 2.89065128 | 1.37206991 |
| 89 | H | -4.07142502 | 5.57763907 | -0.15067154 |
| 90 | H | -6.41218055 | 5.21780076 | 0.70562900 |

**I-2** (-12553.8)

| | | | | |
|---|---|---|---|---|
| 1 | Bi | 1.82693504 | -0.50236459 | -1.12732070 |
| 2 | S | 4.94378154 | -0.77025907 | -2.55197064 |
| 3 | N | -0.26311220 | -1.37617827 | -0.12844738 |
| 4 | H | 3.83870622 | -5.91515258 | 1.23722696 |
| 5 | O | 3.49441670 | -0.96451533 | -2.96055553 |
| 6 | C | -0.58947759 | -1.95646792 | -1.41681099 |
| 7 | C | 0.38907247 | -1.69962396 | -2.38947770 |
| 8 | O | 5.08739610 | -0.39460758 | -1.12956085 |
| 9 | O | 5.84329812 | -1.80659791 | -3.08080332 |
| 10 | C | -0.13488125 | -2.24416794 | 0.99595828 |

| | | | | |
|---|---|---|---|---|
| 11 | F | 4.59331878 | 1.83769541 | -3.13063434 |
| 12 | C | -0.39773349 | -3.62928760 | 0.94822427 |
| 13 | H | -0.73894590 | -4.08616094 | 0.02495219 |
| 14 | F | 6.69613599 | 1.15489724 | -3.24709633 |
| 15 | C | -1.77452978 | -2.61356162 | -1.77445133 |
| 16 | H | -2.56050606 | -2.79169730 | -1.04441315 |
| 17 | C | 0.22823406 | -2.08368982 | -3.71940832 |
| 18 | H | 0.99155792 | -1.86760975 | -4.46474125 |
| 19 | C | -0.94759439 | -2.76566102 | -4.07233076 |
| 20 | H | -1.10085105 | -3.08814455 | -5.10083265 |
| 21 | H | -5.00463132 | 5.05377897 | 1.71768636 |
| 22 | H | -4.08177901 | 5.02102783 | -2.50955507 |
| 23 | N | 2.72353582 | -2.55090021 | -0.29626279 |
| 24 | C | 0.35394547 | -1.70361574 | 2.20547689 |
| 25 | H | 0.62029838 | -0.65241386 | 2.24519062 |
| 26 | C | -0.19943641 | -4.42913107 | 2.08004788 |
| 27 | H | -0.40124178 | -5.49736603 | 2.01320671 |
| 28 | C | 0.54102839 | -2.50427294 | 3.33296262 |
| 29 | H | 0.92558196 | -2.05603565 | 4.24778302 |
| 30 | C | -1.93415317 | -3.02067091 | -3.10621293 |
| 31 | H | -2.84985468 | -3.52897997 | -3.39839563 |
| 32 | C | 0.26100448 | -3.87723761 | 3.28172372 |
| 33 | H | 0.41451922 | -4.50616225 | 4.15634556 |
| 34 | C | 3.21475700 | -2.59027219 | 0.96454084 |
| 35 | C | 2.63114708 | -3.68911960 | -1.02473954 |
| 36 | C | 3.62046518 | -3.78690765 | 1.54728475 |
| 37 | C | 5.40489594 | 0.80729972 | -3.50671979 |
| 38 | C | 3.03419164 | -4.91424697 | -0.50364039 |
| 39 | F | 5.26661769 | 0.61568728 | -4.84828660 |
| 40 | Bi | -2.05522135 | 0.59824173 | 0.06881880 |
| 41 | S | -4.75276218 | 0.51990907 | -2.73298853 |
| 42 | H | -0.69729822 | 5.06409104 | -0.81846029 |
| 43 | H | -5.19645380 | 6.13750308 | -0.54639496 |
| 44 | O | -3.27432745 | 0.40135232 | -2.81858651 |
| 45 | H | 0.92046906 | 1.33929617 | 1.51587933 |
| 46 | H | 0.57088587 | 2.44180236 | 2.82597297 |
| 47 | O | -5.28938467 | 0.65909373 | -1.35826540 |
| 48 | O | -5.36439701 | 1.41093879 | -3.74843002 |
| 49 | C | -0.41258421 | 1.76964176 | -1.08607436 |
| 50 | F | -5.00667096 | -2.15641559 | -2.33614522 |
| 51 | C | -1.17811204 | 1.36321072 | 2.09157372 |
| 52 | C | -0.18235260 | 1.48808654 | -2.45156787 |
| 53 | F | -6.73535895 | -1.22042104 | -3.34652627 |
| 54 | C | 1.57251057 | 3.16099534 | -2.54301148 |
| 55 | H | 0.97704040 | 1.93080439 | -4.22526876 |
| 56 | H | 1.91692730 | 4.24732960 | -0.70360875 |
| 57 | C | -0.50079663 | 4.36970186 | 1.22932059 |
| 58 | H | 2.35076279 | 3.68705874 | -3.09138754 |
| 59 | C | -0.87650562 | 5.29527483 | 0.22598180 |
| 60 | C | -1.43086944 | 5.90599118 | 2.89122220 |
| 61 | H | -0.55410951 | 4.01343950 | 3.37878903 |
| 62 | C | -1.78735791 | 6.82045430 | 1.89260662 |
| 63 | C | 0.34327042 | 2.79501886 | -0.46959001 |

| | | | | |
|---|---|---|---|---|
| 64 | C | 0.17073593 | 2.05026564 | 1.88193341 |
| 65 | H | -1.91404673 | 2.05678038 | 2.50790831 |
| 66 | H | -1.10447580 | 0.50371284 | 2.76332521 |
| 67 | C | 0.81007478 | 2.16957915 | -3.17675154 |
| 68 | H | -0.79229125 | 0.74358475 | -2.95549408 |
| 69 | C | -0.80088436 | 4.69829622 | 2.57402358 |
| 70 | C | -1.50438748 | 6.49613048 | 0.55870834 |
| 71 | C | 1.33453945 | 3.47504430 | -1.20202467 |
| 72 | N | 0.13807906 | 3.15597198 | 0.90283082 |
| 73 | H | -1.79274623 | 7.17648121 | -0.24134240 |
| 74 | N | -3.23663681 | 2.82500886 | -0.09351337 |
| 75 | C | -3.32943038 | 3.40450667 | -1.31028034 |
| 76 | C | -5.36809734 | -1.20503756 | -3.25124753 |
| 77 | C | -3.83658338 | 3.41506027 | 0.96176218 |
| 78 | C | -4.02976445 | 4.59162682 | -1.51245537 |
| 79 | C | -4.54709705 | 4.60789586 | 0.83882036 |
| 80 | C | -4.64872284 | 5.20638211 | -0.42008603 |
| 81 | H | -2.83361349 | 2.89168058 | -2.12888740 |
| 82 | H | -3.74131523 | 2.91645715 | 1.92214079 |
| 83 | F | -4.85966957 | -1.57199939 | -4.46708381 |
| 84 | C | 3.52853794 | -4.96724059 | 0.80432936 |
| 85 | H | 3.27630551 | -1.64722480 | 1.49848764 |
| 86 | H | 2.22036727 | -3.59196370 | -2.02461359 |
| 87 | H | 2.95079566 | -5.80800249 | -1.11553194 |
| 88 | H | 3.99837948 | -3.78394210 | 2.56547813 |
| 89 | H | -1.65015222 | 6.12428307 | 3.93582018 |
| 90 | H | -2.28729343 | 7.75357373 | 2.14462255 |

**Product** (-8219.0)

| | | | | |
|---|---|---|---|---|
| 1 | C | 4.98631048 | -2.80281730 | -1.35650146 |
| 2 | C | 4.96534328 | 3.37096789 | 1.22432642 |
| 3 | H | 5.97632979 | -3.24470868 | -1.45872647 |
| 4 | C | 3.43491710 | 1.68466242 | 2.09469175 |
| 5 | H | 5.41471245 | 4.36022485 | 1.28578896 |
| 6 | Bi | 0.34557324 | -0.65193039 | -0.93944308 |
| 7 | S | -2.87940191 | 1.29723905 | -0.26790812 |
| 8 | N | 3.19387452 | -0.48892274 | 0.97121877 |
| 9 | C | -3.34669566 | 1.27800649 | 1.57682964 |
| 10 | O | -1.49854892 | 1.84341469 | -0.26079260 |
| 11 | C | 4.02740333 | 2.94896047 | 2.17458741 |
| 12 | C | 3.44873286 | -1.35394300 | -0.14818849 |
| 13 | C | 2.42646184 | -1.65843569 | -1.07061842 |
| 14 | H | 3.73885662 | 3.61226693 | 2.98943857 |
| 15 | O | -3.90650597 | 2.18087775 | -0.86705674 |
| 16 | O | -2.97600760 | -0.13668938 | -0.64728708 |
| 17 | C | 3.77461572 | 0.78794184 | 1.05008230 |
| 18 | F | -3.24286757 | 2.52760724 | 2.12336208 |
| 19 | C | 4.71292637 | 1.23177007 | 0.08541208 |
| 20 | H | 4.96923381 | 0.59025121 | -0.75072185 |
| 21 | H | 2.70097046 | 1.40620556 | 2.84378001 |
| 22 | C | 5.29363602 | 2.49670032 | 0.17840310 |
| 23 | H | 5.99926566 | 2.80772558 | -0.59091458 |
| 24 | F | -4.63387604 | 0.84682046 | 1.74910810 |

| | | | | |
|---|---|---|---|---|
| 25 | C | 4.72174497 | -1.93448330 | -0.29211090 |
| 26 | H | 5.49674698 | -1.70095514 | 0.43600377 |
| 27 | C | 2.70319936 | -2.54607401 | -2.12405037 |
| 28 | H | 1.92331898 | -2.79364006 | -2.84551013 |
| 29 | C | 3.97513679 | -3.11638335 | -2.27589443 |
| 30 | H | 4.17488326 | -3.79766655 | -3.10185288 |
| 31 | F | -2.52702620 | 0.44000151 | 2.28425295 |
| 32 | H | -2.33827359 | -2.05561716 | 0.36040099 |
| 33 | H | 0.87445993 | 2.59986743 | -0.02071297 |
| 34 | N | -0.46066420 | -2.89485201 | 0.06481881 |
| 35 | C | 0.33912073 | -3.97067843 | 0.23788201 |
| 36 | C | -0.10168738 | -5.13123900 | 0.87553967 |
| 37 | C | -1.41557116 | -5.18316603 | 1.35291074 |
| 38 | C | -2.24397291 | -4.07036964 | 1.17221224 |
| 39 | C | -1.73306964 | -2.94484971 | 0.52449383 |
| 40 | H | 1.35191259 | -3.88288106 | -0.14745238 |
| 41 | H | 0.57759803 | -5.97191715 | 0.99387238 |
| 42 | H | -3.27177111 | -4.06707993 | 1.52721417 |
| 43 | N | 1.67183201 | 1.43071694 | -1.54009209 |
| 44 | C | 2.50678266 | 1.36830481 | -2.60056551 |
| 45 | C | 3.25615703 | 2.46575515 | -3.02116062 |
| 46 | C | 3.14918566 | 3.66672080 | -2.31261614 |
| 47 | C | 2.29292430 | 3.72710876 | -1.21016008 |
| 48 | C | 1.56598521 | 2.59115439 | -0.85680227 |
| 49 | H | 2.57486429 | 0.40870384 | -3.10778471 |
| 50 | H | 3.91410928 | 2.37134274 | -3.88140443 |
| 51 | H | 2.18829395 | 4.63546130 | -0.62328220 |
| 52 | H | 3.72958961 | 4.53736576 | -2.60977515 |
| 53 | H | -1.78641826 | -6.07321650 | 1.85684444 |
| 54 | C | 1.99934760 | -0.81967188 | 1.77911640 |
| 55 | C | 0.71017673 | -0.15746727 | 1.29639265 |
| 56 | H | 1.89926428 | -1.90951722 | 1.75676645 |
| 57 | H | -0.16237242 | -0.52763721 | 1.84143759 |
| 58 | H | 2.20674171 | -0.55385506 | 2.82443615 |
| 59 | H | 0.73803937 | 0.93119979 | 1.37765590 |

**1-hexene** (-2172.2)

| | | | | |
|---|---|---|---|---|
| 1 | C | -0.56443467 | -0.01164420 | 0.08170322 |
| 2 | C | 0.68042695 | 0.16857856 | -0.37913417 |
| 3 | H | -1.88418790 | -3.60445862 | 3.72265993 |
| 4 | H | -1.12030647 | 0.85279092 | 0.45903939 |
| 5 | H | 1.27068821 | -0.66689251 | -0.75988746 |
| 6 | H | 1.14811908 | 1.15267864 | -0.39334780 |
| 7 | C | -1.28241738 | -1.33450765 | 0.15488183 |
| 8 | H | -2.21362536 | -1.27302040 | -0.43222015 |
| 9 | H | -0.66317402 | -2.11893602 | -0.30395354 |
| 10 | C | -1.64822759 | -1.73907719 | 1.60232502 |
| 11 | H | -0.72413832 | -1.82729575 | 2.19387486 |
| 12 | H | -2.24016261 | -0.93388285 | 2.06491898 |
| 13 | C | -2.43537897 | -3.05858270 | 1.67723861 |
| 14 | H | -3.35712357 | -2.96202234 | 1.08306311 |
| 15 | H | -1.84427615 | -3.85846344 | 1.20526043 |
| 16 | C | -2.79107538 | -3.46418677 | 3.11818782 |

| | | | | |
|---|---|---|---|---|
| 17 | H | -3.40105432 | -2.69065162 | 3.60524857 |
| 18 | H | -3.35870731 | -4.40362835 | 3.14014588 |

**I-1** (-14016.2)

| | | | | |
|---|---|---|---|---|
| 1 | Bi | 2.06120908 | -0.73583014 | 0.07014886 |
| 2 | S | 5.35425143 | 0.20565282 | -0.41171478 |
| 3 | N | -0.35802466 | -1.62261272 | -0.25588742 |
| 4 | H | 2.60863542 | -7.17655366 | 0.56725875 |
| 5 | O | 4.10298581 | -0.15124611 | -1.19910379 |
| 6 | C | -0.03489668 | -1.72595023 | -1.66694469 |
| 7 | C | 1.23598350 | -1.23049995 | -1.98006567 |
| 8 | O | 5.12371847 | 0.23160443 | 1.04663514 |
| 9 | O | 6.56182107 | -0.47461232 | -0.90400131 |
| 10 | C | -0.82605849 | -2.79251115 | 0.42889329 |
| 11 | F | 4.50254154 | 2.76350759 | -0.58474579 |
| 12 | C | -0.69123038 | -4.07974116 | -0.12678404 |
| 13 | H | -0.26702658 | -4.19959046 | -1.11748363 |
| 14 | F | 6.67666926 | 2.54196925 | -0.24268224 |
| 15 | C | -0.90171762 | -2.15929802 | -2.67782268 |
| 16 | H | -1.89863386 | -2.51916717 | -2.43826969 |
| 17 | C | 1.66937451 | -1.13281972 | -3.30096113 |
| 18 | H | 2.65050651 | -0.72678021 | -3.53791407 |
| 19 | C | 0.80671657 | -1.56578507 | -4.32007082 |
| 20 | H | 1.11948074 | -1.49757324 | -5.36073815 |
| 21 | H | 2.22123321 | -3.23713017 | 2.27747194 |
| 22 | H | 2.75151594 | -3.58321419 | -1.82525325 |
| 23 | N | 2.49479902 | -3.31697896 | 0.21716380 |
| 24 | C | -1.36525160 | -2.67082254 | 1.72437398 |
| 25 | H | -1.47857269 | -1.68910883 | 2.17781375 |
| 26 | C | -1.08123403 | -5.20812088 | 0.59975641 |
| 27 | H | -0.95717963 | -6.19323826 | 0.15313898 |
| 28 | C | -1.75306452 | -3.80328596 | 2.44468730 |
| 29 | H | -2.16842152 | -3.68168607 | 3.44374032 |
| 30 | C | -0.46314365 | -2.07821976 | -4.00538727 |
| 31 | H | -1.12644627 | -2.40027722 | -4.80640906 |
| 32 | C | -1.61379694 | -5.08207403 | 1.88859676 |
| 33 | H | -1.91500682 | -5.96383246 | 2.45088235 |
| 34 | C | 2.36229071 | -3.90181570 | 1.42687204 |
| 35 | C | 2.66954371 | -4.09798248 | -0.87069893 |
| 36 | C | 2.39441107 | -5.28504881 | 1.59599704 |
| 37 | C | 5.60208073 | 2.02644206 | -0.90310392 |
| 38 | C | 2.72457357 | -5.48925298 | -0.78341650 |
| 39 | F | 5.82193658 | 2.13126780 | -2.24335805 |
| 40 | Bi | -1.53741590 | 0.41034008 | 0.17685407 |
| 41 | S | -4.72928324 | -0.66884508 | -0.59904956 |
| 42 | N | 0.87297822 | 1.41049233 | -0.05134988 |
| 43 | H | 2.86962043 | -6.08137946 | -1.68350879 |
| 44 | O | -3.37915839 | -0.45462852 | -1.25782278 |
| 45 | C | 0.48195930 | 1.78601507 | -1.38973004 |
| 46 | C | -0.76184814 | 1.27735290 | -1.78439716 |
| 47 | O | -4.68741202 | -0.49799835 | 0.86720827 |
| 48 | O | -5.83641012 | -0.03005812 | -1.32842636 |
| 49 | C | 1.29164043 | 2.46662590 | 0.82290383 |

| | | | | |
|---|---|---|---|---|
| 50 | F | -4.00848978 | -3.24839931 | -0.26705808 |
| 51 | C | 0.57494255 | 3.68310743 | 0.84561973 |
| 52 | H | -0.25304077 | 3.82390748 | 0.15753944 |
| 53 | F | -6.19347591 | -2.91146768 | -0.34228645 |
| 54 | C | 1.26611971 | 2.52746765 | -2.28554162 |
| 55 | H | 2.23083569 | 2.92689417 | -1.97993747 |
| 56 | C | -1.23336322 | 1.45261364 | -3.08519026 |
| 57 | H | -2.19591701 | 1.04363310 | -3.38616111 |
| 58 | C | -0.45008056 | 2.18157107 | -3.99210680 |
| 59 | H | -0.79898098 | 2.33690298 | -5.01169984 |
| 60 | H | -1.24183472 | 2.21949261 | 2.98222527 |
| 61 | H | 0.79516781 | -1.39201590 | 2.94696867 |
| 62 | H | 2.18752967 | -0.72989445 | 4.91164750 |
| 63 | C | 2.36349486 | 2.30688481 | 1.71732109 |
| 64 | H | 2.96271528 | 1.40055746 | 1.71211580 |
| 65 | C | 0.91571623 | 4.69729313 | 1.74273382 |
| 66 | H | 0.34361127 | 5.62409244 | 1.74610032 |
| 67 | C | 2.69864090 | 3.32240821 | 2.61878033 |
| 68 | H | 3.53093322 | 3.17082593 | 3.30369540 |
| 69 | C | 0.78273433 | 2.72288827 | -3.58594675 |
| 70 | H | 1.37890618 | 3.29331953 | -4.29664367 |
| 71 | C | 1.97941626 | 4.52263141 | 2.64046019 |
| 72 | H | 2.24193310 | 5.31036028 | 3.34402344 |
| 73 | H | 0.01748284 | 3.01266560 | 4.97954900 |
| 74 | H | 2.27163143 | -5.71256249 | 2.58757431 |
| 75 | H | 1.78530545 | 1.51970708 | 5.97136795 |
| 76 | C | -4.99422685 | -2.53128764 | -0.86800486 |
| 77 | N | -0.26854863 | 0.38716462 | 2.86355651 |
| 78 | C | -0.48067293 | 1.58907296 | 3.43952619 |
| 79 | C | 0.67216096 | -0.41217323 | 3.40866089 |
| 80 | C | 0.22588199 | 2.03210511 | 4.55999666 |
| 81 | C | 1.43720669 | -0.04785736 | 4.51863539 |
| 82 | C | 1.20744020 | 1.20073568 | 5.10672736 |
| 83 | F | -4.99291651 | -2.82369541 | -2.20088606 |
| 84 | C | 2.58053685 | -6.09355266 | 0.47012708 |
| 85 | C | -3.47728019 | 2.94600879 | 0.74754219 |
| 86 | H | -2.98797912 | 3.61617371 | 1.45526484 |
| 87 | H | -4.09789877 | 2.15297716 | 1.16162646 |
| 88 | C | -3.36061898 | 3.12649660 | -0.58045544 |
| 89 | H | -3.50138465 | 5.76860575 | -5.10089743 |
| 90 | H | -3.87946354 | 2.43508494 | -1.25004749 |
| 91 | C | -2.56692095 | 4.21226002 | -1.25215979 |
| 92 | H | -2.27521857 | 4.97453771 | -0.51620672 |
| 93 | H | -1.64091788 | 3.77202280 | -1.65073791 |
| 94 | C | -3.31480253 | 4.86343280 | -2.43378783 |
| 95 | H | -3.63551342 | 4.07238500 | -3.12890989 |
| 96 | H | -4.23042910 | 5.35278343 | -2.06908480 |
| 97 | C | -2.44396145 | 5.88010158 | -3.19058776 |
| 98 | H | -2.10848558 | 6.66161708 | -2.49168338 |
| 99 | H | -1.53585855 | 5.37059711 | -3.54755472 |
| 100 | C | -3.17538420 | 6.52868444 | -4.37728998 |
| 101 | H | -4.06823674 | 7.07335785 | -4.04012952 |
| 102 | H | -2.52728714 | 7.23993750 | -4.90565881 |

**TS-1** (-14000.3)

| | | | | |
|---|---|---|---|---|
| 1 | Bi | 2.03914517 | -0.34411095 | -0.03453883 |
| 2 | S | 5.37749284 | -0.81530926 | -0.88094009 |
| 3 | N | -0.22466441 | -1.29760042 | 0.01507196 |
| 4 | H | 3.25705655 | -6.62278447 | 0.35637178 |
| 5 | O | 4.13601988 | -0.26297603 | -1.54518093 |
| 6 | C | -0.15678292 | -1.44896632 | -1.42984375 |
| 7 | C | 0.99224268 | -0.85651651 | -1.96885360 |
| 8 | O | 5.35442426 | -0.66990529 | 0.59028863 |
| 9 | O | 5.82608782 | -2.10843621 | -1.42327113 |
| 10 | C | -0.40042032 | -2.45050154 | 0.84563572 |
| 11 | F | 6.41492081 | 1.68048618 | -1.04354962 |
| 12 | C | -0.47804371 | -3.76363881 | 0.34712407 |
| 13 | H | -0.42884260 | -3.94160834 | -0.72189765 |
| 14 | F | 7.92229766 | 0.06328221 | -0.95848457 |
| 15 | C | -1.14277322 | -1.97887470 | -2.27056606 |
| 16 | H | -2.04775851 | -2.42235701 | -1.86917621 |
| 17 | C | 1.18571612 | -0.75361205 | -3.34248666 |
| 18 | H | 2.07955922 | -0.28628926 | -3.75056252 |
| 19 | C | 0.19517776 | -1.27358557 | -4.19277490 |
| 20 | H | 0.31480472 | -1.20221757 | -5.27261465 |
| 21 | H | 3.18706429 | -5.15803310 | 2.40244672 |
| 22 | H | 2.81563631 | 3.80513603 | 6.09225835 |
| 23 | N | 2.67161904 | -2.80981104 | 0.03611289 |
| 24 | C | -0.43922986 | -2.25239690 | 2.24145425 |
| 25 | H | -0.35828614 | -1.24241983 | 2.63983398 |
| 26 | C | -0.59003248 | -4.84495764 | 1.22735452 |
| 27 | H | -0.63591666 | -5.85445857 | 0.82204723 |
| 28 | C | -0.55272986 | -3.33636733 | 3.11520735 |
| 29 | H | -0.57486717 | -3.15908791 | 4.18915559 |
| 30 | C | -0.94996697 | -1.88275796 | -3.65629589 |
| 31 | H | -1.71300296 | -2.27590280 | -4.32587056 |
| 32 | C | -0.63069483 | -4.64316653 | 2.61257395 |
| 33 | H | -0.71353389 | -5.48980316 | 3.29121015 |
| 34 | C | 2.84902351 | -3.37347986 | 1.25022493 |
| 35 | C | 2.70577200 | -3.58759753 | -1.06683256 |
| 36 | C | 3.05861463 | -4.74182471 | 1.40694896 |
| 37 | C | 6.70519089 | 0.41526662 | -1.46410648 |
| 38 | C | 2.91906709 | -4.96302423 | -0.99040990 |
| 39 | F | 6.78916147 | 0.42401733 | -2.82514736 |
| 40 | Bi | -2.12040380 | 0.34087464 | 0.36827103 |
| 41 | S | -5.26771893 | -1.38061385 | -0.77083143 |
| 42 | N | 1.03459300 | 2.03059843 | -0.12039247 |
| 43 | H | 0.19840550 | 3.28944523 | 1.63721641 |
| 44 | O | -4.15817994 | -0.69956282 | -1.49850237 |
| 45 | C | 0.14030124 | 2.17423149 | -1.23456504 |
| 46 | C | -1.13888698 | 1.56812062 | -1.28126446 |
| 47 | O | -5.19811227 | -1.25599773 | 0.70599106 |
| 48 | O | -6.60693811 | -1.19513885 | -1.37187027 |
| 49 | C | 2.01435779 | 3.08844475 | -0.02113238 |
| 50 | F | -3.70508749 | -3.57328246 | -0.48656556 |
| 51 | C | 1.58550029 | 4.43145161 | 0.07183265 |

| | | | | |
|---|---|---|---|---|
| 52 | H | 0.52736377 | 4.66158123 | -0.02857280 |
| 53 | F | -5.87435034 | -4.00673224 | -0.51146616 |
| 54 | C | 0.59756615 | 2.84734603 | -2.39788316 |
| 55 | H | 1.59058541 | 3.28621340 | -2.40101786 |
| 56 | C | -1.91223807 | 1.66066770 | -2.45378142 |
| 57 | H | -2.89040585 | 1.18519052 | -2.48709928 |
| 58 | C | -1.44940328 | 2.33213728 | -3.58640114 |
| 59 | H | -2.06069211 | 2.37838063 | -4.48593919 |
| 60 | C | -1.08361622 | 1.63827715 | 2.11564640 |
| 61 | H | -1.08100347 | 0.91222009 | 2.93158130 |
| 62 | H | -1.89001486 | 2.36627045 | 2.21458726 |
| 63 | C | 3.39033207 | 2.82244961 | 0.07073301 |
| 64 | H | 3.77300501 | 1.81203056 | -0.04077238 |
| 65 | C | 2.50171379 | 5.46443129 | 0.27265723 |
| 66 | H | 2.14527811 | 6.49058794 | 0.34517002 |
| 67 | C | 4.31000167 | 3.85991700 | 0.27744908 |
| 68 | H | 5.36959449 | 3.62569171 | 0.34754915 |
| 69 | C | -0.17928126 | 2.92185653 | -3.55104604 |
| 70 | H | 0.21784589 | 3.43095445 | -4.42806996 |
| 71 | C | 3.87187709 | 5.18295364 | 0.38515949 |
| 72 | H | 4.58687516 | 5.98732666 | 0.54722848 |
| 73 | C | 0.20289759 | 2.22481236 | 1.84589017 |
| 74 | F | -4.82413022 | -3.50639611 | -2.39470879 |
| 75 | C | 3.09520817 | -5.55095746 | 0.26664966 |
| 76 | C | -4.89810704 | -3.22359691 | -1.05904246 |
| 77 | H | 2.81031453 | -2.70668373 | 2.10895676 |
| 78 | H | 2.55180480 | -3.08265202 | -2.01658869 |
| 79 | H | 2.94407333 | -5.55598514 | -1.90104972 |
| 80 | N | -3.84867879 | 2.27914339 | 0.38736762 |
| 81 | C | -5.10272679 | 2.05238974 | 0.83868996 |
| 82 | C | -3.51123867 | 3.52557005 | -0.01269077 |
| 83 | C | -6.06182383 | 3.06494292 | 0.90018530 |
| 84 | H | -5.32961352 | 1.03246036 | 1.13901560 |
| 85 | C | -4.41284439 | 4.58848481 | 0.02465706 |
| 86 | H | -2.49553303 | 3.65245745 | -0.38014061 |
| 87 | C | -5.71281367 | 4.35465574 | 0.48780223 |
| 88 | H | -7.06030020 | 2.83837653 | 1.26602355 |
| 89 | H | -4.09857496 | 5.57486961 | -0.30751488 |
| 90 | H | -6.43961348 | 5.16320437 | 0.52538296 |
| 91 | C | 1.37497727 | 1.74468659 | 2.65670528 |
| 92 | C | 1.42710873 | 2.47521074 | 4.02444148 |
| 93 | H | 2.32409250 | 1.93632937 | 2.14642947 |
| 94 | H | 1.28988386 | 0.66339230 | 2.83864442 |
| 95 | C | 2.60785387 | 1.99345474 | 4.88538991 |
| 96 | H | 1.51698060 | 3.55621994 | 3.84315057 |
| 97 | H | 0.48293433 | 2.31940367 | 4.56432552 |
| 98 | C | 2.68928217 | 2.72341894 | 6.23647191 |
| 99 | H | 3.54473457 | 2.14097695 | 4.32754015 |
| 100 | H | 2.51231248 | 0.91054097 | 5.05532720 |
| 101 | H | 3.53766935 | 2.36118285 | 6.83094060 |
| 102 | H | 1.77427128 | 2.56732025 | 6.82409557 |

**I-2** (-14016.6)

| | | | | |
|---|---|---|---|---|
| 1 | Bi | 1.83825204 | -0.70126331 | -1.23933338 |
| 2 | S | 4.99097465 | -0.98913982 | -2.60574511 |
| 3 | N | -0.30581060 | -1.51937369 | -0.22752586 |
| 4 | H | 3.73490650 | -6.15019561 | 1.12415629 |
| 5 | O | 3.53854372 | -1.14011347 | -3.03406206 |
| 6 | C | -0.65411079 | -2.01832140 | -1.54413912 |
| 7 | C | 0.34278717 | -1.81171697 | -2.50925136 |
| 8 | O | 5.16453108 | -1.00960503 | -1.14140002 |
| 9 | O | 5.91253053 | -1.79048322 | -3.42519528 |
| 10 | C | -0.23861507 | -2.48954795 | 0.81777222 |
| 11 | F | 4.51874152 | 1.65207017 | -2.39366979 |
| 12 | C | -0.30218911 | -3.88069230 | 0.57966408 |
| 13 | H | -0.44407186 | -4.25055175 | -0.43081298 |
| 14 | F | 6.63872423 | 1.13607478 | -2.76773807 |
| 15 | C | -1.87626021 | -2.59421350 | -1.91325290 |
| 16 | H | -2.66528961 | -2.74510234 | -1.18061303 |
| 17 | C | 0.15858886 | -2.15816950 | -3.84741987 |
| 18 | H | 0.93560756 | -1.98010254 | -4.58880652 |
| 19 | C | -1.05974583 | -2.75228897 | -4.21368003 |
| 20 | H | -1.23260048 | -3.04338099 | -5.24848668 |
| 21 | H | -4.97297211 | 5.02579706 | 1.76347582 |
| 22 | H | -4.07320861 | 4.87861875 | -2.46595921 |
| 23 | N | 2.65898632 | -2.76103376 | -0.37819965 |
| 24 | C | -0.02617154 | -2.05535889 | 2.14310356 |
| 25 | H | 0.04667497 | -0.99400206 | 2.34360739 |
| 26 | C | -0.16164485 | -4.79060611 | 1.63207693 |
| 27 | H | -0.20351537 | -5.85754122 | 1.41756137 |
| 28 | C | 0.11332030 | -2.96871928 | 3.19050318 |
| 29 | H | 0.28048780 | -2.59971520 | 4.20136178 |
| 30 | C | -2.06251345 | -2.96165770 | -3.25202131 |
| 31 | H | -3.00872467 | -3.40429717 | -3.55399341 |
| 32 | C | 0.04566523 | -4.34690437 | 2.94461769 |
| 33 | H | 0.15873985 | -5.06047887 | 3.75835384 |
| 34 | C | 2.99641859 | -2.84439621 | 0.93010553 |
| 35 | C | 2.69845222 | -3.86343125 | -1.16207237 |
| 36 | C | 3.38316629 | -4.05109820 | 1.50291924 |
| 37 | C | 5.35383455 | 0.81493809 | -3.08072947 |
| 38 | C | 3.09178914 | -5.09694038 | -0.65198053 |
| 39 | F | 5.16466632 | 1.01747421 | -4.41342222 |
| 40 | Bi | -1.94687439 | 0.56522292 | 0.22622074 |
| 41 | S | -4.83100487 | 0.46976573 | -2.40074117 |
| 42 | H | -0.64114073 | 4.95153087 | -0.97031891 |
| 43 | H | -5.20004338 | 6.03408956 | -0.53219559 |
| 44 | O | -3.36432682 | 0.32594742 | -2.58829709 |
| 45 | H | 3.00247218 | 2.69010124 | 2.49294855 |
| 46 | H | 0.49086330 | 2.79484225 | 2.91615827 |
| 47 | O | -5.27908098 | 0.51467585 | -0.98839541 |
| 48 | O | -5.48156211 | 1.44662736 | -3.30685385 |
| 49 | C | -0.35688806 | 1.71084698 | -1.00361790 |
| 50 | F | -5.12813611 | -2.22766037 | -2.20016257 |
| 51 | C | -1.03473637 | 1.45821572 | 2.20177093 |
| 52 | C | -0.17299270 | 1.39054581 | -2.36748814 |
| 53 | F | -6.89352371 | -1.17666160 | -3.01450786 |

| | | | | |
|---|---|---|---|---|
| 54 | C | 1.57562619 | 3.05728578 | -2.56515685 |
| 55 | H | 0.92035849 | 1.78581900 | -4.19409883 |
| 56 | H | 1.96541739 | 4.20579416 | -0.77696401 |
| 57 | C | -0.43651807 | 4.44407405 | 1.13194870 |
| 58 | H | 2.33410508 | 3.57030711 | -3.15204353 |
| 59 | C | -0.81979478 | 5.27414366 | 0.04902126 |
| 60 | C | -1.38300172 | 6.12852583 | 2.64005920 |
| 61 | H | -0.51236556 | 4.29850960 | 3.31019560 |
| 62 | C | -1.73937924 | 6.94689777 | 1.56234563 |
| 63 | C | 0.40708844 | 2.75782080 | -0.44088932 |
| 64 | C | 0.28999938 | 2.20981525 | 2.01192125 |
| 65 | H | -1.81504076 | 2.14607409 | 2.54179848 |
| 66 | H | -0.95440721 | 0.66332550 | 2.94904811 |
| 67 | C | 0.79276004 | 2.05080819 | -3.14627297 |
| 68 | H | -0.80543773 | 0.63888495 | -2.83159362 |
| 69 | C | -0.74859037 | 4.89833070 | 2.43823570 |
| 70 | C | -1.45301472 | 6.49809685 | 0.26625499 |
| 71 | C | 1.37772097 | 3.40872924 | -1.22731859 |
| 72 | N | 0.21557893 | 3.20966103 | 0.90960354 |
| 73 | H | -1.74268509 | 7.09743729 | -0.59578142 |
| 74 | N | -3.16601874 | 2.77951274 | 0.01284001 |
| 75 | C | -3.27696920 | 3.32030612 | -1.21988979 |
| 76 | C | -5.52293962 | -1.19663101 | -3.00961125 |
| 77 | C | -3.77494148 | 3.38900968 | 1.05175805 |
| 78 | C | -4.00634822 | 4.48319495 | -1.45586211 |
| 79 | C | -4.51006587 | 4.56288674 | 0.89620703 |
| 80 | C | -4.63167382 | 5.11934172 | -0.38020165 |
| 81 | H | -2.77379701 | 2.79453484 | -2.02508172 |
| 82 | H | -3.66896108 | 2.92125540 | 2.02666929 |
| 83 | F | -5.10433852 | -1.47466203 | -4.28240783 |
| 84 | C | 3.43423741 | -5.19508470 | 0.70069732 |
| 85 | H | 2.94934701 | -1.92831559 | 1.51048057 |
| 86 | H | 2.40424557 | -3.73293104 | -2.19885409 |
| 87 | H | 3.11857678 | -5.96203881 | -1.30844890 |
| 88 | H | 3.63649562 | -4.08544502 | 2.55828482 |
| 89 | H | -1.60715961 | 6.43881983 | 3.66009308 |
| 90 | H | -2.24245714 | 7.89792490 | 1.72546643 |
| 91 | C | 5.38922494 | 1.61941878 | 1.44590338 |
| 92 | H | 3.87081225 | 0.19012586 | 0.92086341 |
| 93 | C | 1.49694284 | 1.26772085 | 1.87731029 |
| 94 | H | 4.04721182 | 0.41457992 | 2.66030400 |
| 95 | H | 6.20104772 | 0.88205638 | 1.45384136 |
| 96 | H | 5.60546744 | 2.37495513 | 2.21346852 |
| 97 | H | 5.39995308 | 2.11771844 | 0.46759998 |
| 98 | C | 2.85834644 | 1.94932920 | 1.69239484 |
| 99 | H | 1.31441479 | 0.56223102 | 1.05647501 |
| 100 | H | 1.52016805 | 0.65590377 | 2.78884624 |
| 101 | C | 4.02907920 | 0.95121973 | 1.69973576 |
| 102 | H | 2.87085802 | 2.50880732 | 0.74933207 |

**Product** (-9681.3)

| | | | | |
|---|---|---|---|---|
| 1 | C | 4.92618294 | -2.91258040 | -0.88666290 |
| 2 | C | 4.83801106 | 3.33886117 | 1.38201312 |

| | | | | |
|---|---|---|---|---|
| 3 | H | 5.91007937 | -3.37842047 | -0.85965047 |
| 4 | C | 3.16275239 | 1.75687778 | 2.18155391 |
| 5 | H | 5.31995464 | 4.31299175 | 1.43732868 |
| 6 | Bi | 0.30638902 | -0.68575028 | -1.02142033 |
| 7 | S | -2.91153713 | 1.33627370 | -0.57339662 |
| 8 | N | 2.95058262 | -0.47482400 | 1.14905848 |
| 9 | C | -3.47451291 | 1.58452171 | 1.22762629 |
| 10 | O | -1.52491808 | 1.86711284 | -0.56997701 |
| 11 | C | 3.79722440 | 3.00110395 | 2.25498005 |
| 12 | C | 3.29703282 | -1.38941162 | 0.09344466 |
| 13 | C | 2.38039901 | -1.70553895 | -0.92993885 |
| 14 | H | 3.45688199 | 3.71714411 | 3.00251295 |
| 15 | O | -3.89244821 | 2.13411792 | -1.34612750 |
| 16 | O | -3.01009888 | -0.13608319 | -0.74945873 |
| 17 | C | 3.56302764 | 0.78714122 | 1.22527852 |
| 18 | F | -3.39530018 | 2.90138604 | 1.58927546 |
| 19 | C | 4.60402977 | 1.15187148 | 0.33239630 |
| 20 | H | 4.91245203 | 0.46122412 | -0.44440119 |
| 21 | H | 2.34666598 | 1.55779944 | 2.86753409 |
| 22 | C | 5.22465559 | 2.39779156 | 0.41726998 |
| 23 | H | 6.01027844 | 2.64066260 | -0.29728136 |
| 24 | F | -4.77072136 | 1.17760986 | 1.39385310 |
| 25 | C | 4.56073142 | -2.00591393 | 0.11359523 |
| 26 | H | 5.25389490 | -1.76502931 | 0.91733111 |
| 27 | C | 2.75666960 | -2.62506626 | -1.92274650 |
| 28 | H | 2.06369819 | -2.86978606 | -2.72887654 |
| 29 | C | 4.02288933 | -3.22781683 | -1.91087197 |
| 30 | H | 4.30029253 | -3.93487854 | -2.69157570 |
| 31 | F | -2.69545509 | 0.86319472 | 2.09219715 |
| 32 | H | -2.32174839 | -1.90112234 | 0.59251371 |
| 33 | H | 0.82281066 | 2.58632250 | -0.09281006 |
| 34 | N | -0.70233878 | -2.93566084 | -0.19457816 |
| 35 | C | -0.08679398 | -4.12865994 | -0.34433648 |
| 36 | C | -0.58753864 | -5.30224033 | 0.21942339 |
| 37 | C | -1.76718642 | -5.24157856 | 0.96931619 |
| 38 | C | -2.41020541 | -4.00819521 | 1.11665108 |
| 39 | C | -1.85010016 | -2.87865927 | 0.51780695 |
| 40 | H | 0.83849699 | -4.12712313 | -0.91516436 |
| 41 | H | -0.05529817 | -6.23932614 | 0.07586471 |
| 42 | H | -3.33143522 | -3.91502703 | 1.68658453 |
| 43 | N | 1.71085701 | 1.37499337 | -1.52615379 |
| 44 | C | 2.62251233 | 1.27881197 | -2.51889821 |
| 45 | C | 3.42145518 | 2.35494470 | -2.90094986 |
| 46 | C | 3.28466671 | 3.57030005 | -2.22253859 |
| 47 | C | 2.34868191 | 3.66604061 | -1.18977997 |
| 48 | C | 1.57547599 | 2.54974167 | -0.87355885 |
| 49 | H | 2.71126791 | 0.30876248 | -3.00268997 |
| 50 | H | 4.14083914 | 2.23292370 | -3.70683532 |
| 51 | H | 2.21754929 | 4.58654216 | -0.62776562 |
| 52 | H | 3.90270329 | 4.42477094 | -2.48911436 |
| 53 | H | -2.17744642 | -6.13817416 | 1.42904677 |
| 54 | C | 1.69953777 | -0.75799244 | 1.91341211 |
| 55 | C | 0.45825208 | -0.16463740 | 1.23283847 |

| | | | | |
|---|---|---|---|---|
| 56 | H | 4.67251840 | -4.66563670 | 3.87185137 |
| 57 | H | -0.46857804 | -0.50439613 | 1.70235333 |
| 58 | H | 1.82469049 | -0.25355363 | 2.87905353 |
| 59 | H | 0.46918596 | 0.92767275 | 1.25773818 |
| 60 | C | 1.56952605 | -2.25688844 | 2.24359252 |
| 61 | H | 0.59152561 | -2.40181705 | 2.72229366 |
| 62 | H | 1.55116776 | -2.84605420 | 1.32235070 |
| 63 | C | 2.67858184 | -2.78716557 | 3.16343882 |
| 64 | H | 3.65866665 | -2.56601029 | 2.71978385 |
| 65 | H | 2.64397574 | -2.25268619 | 4.12576969 |
| 66 | C | 2.56702419 | -4.30100204 | 3.41032399 |
| 67 | H | 1.58598525 | -4.52604901 | 3.85651363 |
| 68 | H | 2.59176299 | -4.82250917 | 2.44114776 |
| 69 | C | 3.68371030 | -4.84658439 | 4.31620604 |
| 70 | H | 3.66620670 | -4.35797901 | 5.30047605 |
| 71 | H | 3.57935814 | -5.92779934 | 4.47584910 |

**Styrene** (-2225.0)

| | | | | |
|---|---|---|---|---|
| 1 | C | -0.56718805 | -0.14196458 | -0.27018825 |
| 2 | C | 0.61252008 | 0.12411999 | 0.31933148 |
| 3 | H | -0.07252870 | -2.41543520 | 1.24766688 |
| 4 | H | -0.99611098 | 0.61634873 | -0.93008457 |
| 5 | H | 1.10702460 | -0.57794993 | 0.98916630 |
| 6 | H | 1.12061866 | 1.06948816 | 0.13995849 |
| 7 | C | -1.37898446 | -1.36525007 | -0.13413005 |
| 8 | C | -2.59202123 | -1.45992315 | -0.84971809 |
| 9 | C | -3.40032194 | -2.59915688 | -0.75798272 |
| 10 | C | -3.01138672 | -3.67129055 | 0.05517679 |
| 11 | C | -1.80688529 | -3.59241815 | 0.77408715 |
| 12 | C | -1.00104958 | -2.45580875 | 0.68165526 |
| 13 | H | -2.89912103 | -0.62819213 | -1.48320638 |
| 14 | H | -4.33199239 | -2.64861605 | -1.31979999 |
| 15 | H | -3.63639216 | -4.55971721 | 0.12969893 |
| 16 | H | -1.49783442 | -4.42214402 | 1.40836914 |

**I-1** (-14071.4)

| | | | | |
|---|---|---|---|---|
| 1 | Bi | 2.05092417 | -0.76802425 | -0.00377938 |
| 2 | S | 5.35540887 | 0.11053759 | -0.51916317 |
| 3 | N | -0.38067738 | -1.61468744 | -0.29505773 |
| 4 | H | 2.53382651 | -7.19619971 | 0.73370602 |
| 5 | O | 4.09048382 | -0.22773471 | -1.29279520 |
| 6 | C | -0.05933180 | -1.78566198 | -1.69995079 |
| 7 | C | 1.22053757 | -1.32757300 | -2.03292922 |
| 8 | O | 5.13836158 | 0.15759458 | 0.94037924 |
| 9 | O | 6.54353700 | -0.60223939 | -1.01363178 |
| 10 | C | -0.86548658 | -2.74958791 | 0.43700614 |
| 11 | F | 4.55918818 | 2.68466194 | -0.72754732 |
| 12 | C | -0.72288582 | -4.06109827 | -0.05698366 |
| 13 | H | -0.28272689 | -4.22550651 | -1.03411763 |
| 14 | F | 6.72811436 | 2.41982671 | -0.38398855 |
| 15 | C | -0.92844735 | -2.25729999 | -2.69160931 |
| 16 | H | -1.92887549 | -2.59507883 | -2.43849869 |

| | | | | |
|---|---|---|---|---|
| 17 | C | 1.66024249 | -1.30261697 | -3.35491181 |
| 18 | H | 2.64901272 | -0.92536250 | -3.60700646 |
| 19 | C | 0.79474471 | -1.77314018 | -4.35460346 |
| 20 | H | 1.11224241 | -1.76267894 | -5.39604910 |
| 21 | H | 2.20647844 | -3.18826677 | 2.29172474 |
| 22 | H | 2.70891254 | -3.70006551 | -1.79696718 |
| 23 | N | 2.46747232 | -3.35194982 | 0.23488575 |
| 24 | C | -1.42944623 | -2.56858550 | 1.71415778 |
| 25 | H | -1.55477884 | -1.56750313 | 2.11895782 |
| 26 | C | -1.12914951 | -5.15486343 | 0.71159174 |
| 27 | H | -0.99955314 | -6.15926631 | 0.31200511 |
| 28 | C | -1.83393996 | -3.66672011 | 2.47728337 |
| 29 | H | -2.26998564 | -3.49925616 | 3.46074718 |
| 30 | C | -0.48329881 | -2.25095171 | -4.01941748 |
| 31 | H | -1.14755152 | -2.60580901 | -4.80565262 |
| 32 | C | -1.68624606 | -4.96958047 | 1.98288070 |
| 33 | H | -2.00107730 | -5.82442039 | 2.57839111 |
| 34 | C | 2.33506046 | -3.88762639 | 1.46703684 |
| 35 | C | 2.62597411 | -4.17658671 | -0.82276362 |
| 36 | C | 2.35049064 | -5.26369208 | 1.68960239 |
| 37 | C | 5.64095044 | 1.91887154 | -1.03565347 |
| 38 | C | 2.66390637 | -5.56412260 | -0.68201774 |
| 39 | F | 5.86206273 | 1.99940637 | -2.37782332 |
| 40 | Bi | -1.52451305 | 0.45945361 | 0.06189543 |
| 41 | S | -4.66590041 | -0.82778474 | -0.62311864 |
| 42 | N | 0.91102880 | 1.39963896 | -0.20604964 |
| 43 | H | 2.79599131 | -6.19264086 | -1.55915645 |
| 44 | O | -3.37617879 | -0.45737701 | -1.33137328 |
| 45 | C | 0.52999204 | 1.72060885 | -1.56115093 |
| 46 | C | -0.72981497 | 1.23276704 | -1.93148344 |
| 47 | O | -4.52691979 | -0.86799750 | 0.84589979 |
| 48 | O | -5.84767412 | -0.14579898 | -1.17626948 |
| 49 | C | 1.39063531 | 2.47325118 | 0.61286869 |
| 50 | F | -3.83811341 | -3.39764345 | -0.71767773 |
| 51 | C | 0.79374868 | 3.74970864 | 0.52546264 |
| 52 | H | 0.01030815 | 3.92439245 | -0.20544425 |
| 53 | F | -6.03184497 | -3.14264728 | -0.59905094 |
| 54 | C | 1.34870743 | 2.36433399 | -2.50020371 |
| 55 | H | 2.32464508 | 2.74954666 | -2.21293488 |
| 56 | C | -1.17377888 | 1.31118111 | -3.25153037 |
| 57 | H | -2.14126537 | 0.90265084 | -3.53734878 |
| 58 | C | -0.35124252 | 1.93284997 | -4.20223527 |
| 59 | H | -0.67631479 | 2.00716475 | -5.23880972 |
| 60 | H | -1.16033863 | 2.41635928 | 2.73508052 |
| 61 | H | 0.72518670 | -1.27261195 | 2.92878603 |
| 62 | H | 2.08806467 | -0.57264583 | 4.90192773 |
| 63 | C | 2.40970477 | 2.27589953 | 1.56183938 |
| 64 | H | 2.91786238 | 1.31947385 | 1.64384530 |
| 65 | C | 1.20025721 | 4.78324696 | 1.37268224 |
| 66 | H | 0.71838776 | 5.75473199 | 1.29042825 |
| 67 | C | 2.81043652 | 3.31209940 | 2.40915206 |
| 68 | H | 3.59932641 | 3.12866574 | 3.13638278 |
| 69 | C | 0.89092813 | 2.46947963 | -3.81936317 |

| | | | | |
|---|---|---|---|---|
| 70 | H | 1.51764915 | 2.95757204 | -4.56432577 |
| 71 | C | 2.20860943 | 4.57281268 | 2.32382119 |
| 72 | H | 2.51995963 | 5.37892398 | 2.98560204 |
| 73 | H | 0.07548358 | 3.25384785 | 4.72931066 |
| 74 | H | 2.22792278 | -5.65110361 | 2.69759968 |
| 75 | H | 1.74889969 | 1.73854225 | 5.84374557 |
| 76 | C | -4.88594761 | -2.64248305 | -1.14283135 |
| 77 | N | -0.26559075 | 0.54035025 | 2.73610675 |
| 78 | C | -0.44150237 | 1.77686189 | 3.24642919 |
| 79 | C | 0.62786720 | -0.26868280 | 3.34262600 |
| 80 | C | 0.25258610 | 2.24574185 | 4.36393786 |
| 81 | C | 1.37669025 | 0.11856479 | 4.45604373 |
| 82 | C | 1.18219006 | 1.40194665 | 4.97841693 |
| 83 | F | -4.96984681 | -2.74252333 | -2.50082201 |
| 84 | C | 2.51928380 | -6.11746954 | 0.59484603 |
| 85 | C | -3.79376683 | 2.68325577 | 0.60847002 |
| 86 | H | -3.47927078 | 3.15362220 | 1.53927671 |
| 87 | H | -4.44760483 | 1.82049571 | 0.69881965 |
| 88 | C | -3.44250043 | 3.17767593 | -0.59865273 |
| 89 | H | -2.14627775 | 3.78065245 | -2.86572408 |
| 90 | H | -3.77322377 | 2.64584482 | -1.49289839 |
| 91 | C | -2.61685709 | 4.37036986 | -0.84053476 |
| 92 | C | -2.40708080 | 5.35747306 | 0.14766500 |
| 93 | C | -1.59288745 | 6.46100049 | -0.11255097 |
| 94 | C | -0.95898379 | 6.59592316 | -1.35760030 |
| 95 | C | -1.16577037 | 5.62803808 | -2.34920522 |
| 96 | C | -1.99874317 | 4.53441686 | -2.09746674 |
| 97 | H | -2.89498712 | 5.26887220 | 1.11609486 |
| 98 | H | -1.44911647 | 7.21826202 | 0.65623753 |
| 99 | H | -0.31516254 | 7.45170713 | -1.55258836 |
| 100 | H | -0.67790454 | 5.72244171 | -3.31749358 |

**TS-1** (-14055.0)

| | | | | |
|---|---|---|---|---|
| 1 | Bi | 2.00390360 | -0.29000769 | 0.12620587 |
| 2 | S | 5.38799418 | -0.60633615 | -0.66157081 |
| 3 | N | -0.29905261 | -1.28270257 | 0.08033036 |
| 4 | H | 3.55205293 | -6.47152140 | 0.48299173 |
| 5 | O | 4.11919226 | -0.24369753 | -1.39746463 |
| 6 | C | -0.16948622 | -1.35090883 | -1.36727264 |
| 7 | C | 0.99403412 | -0.73953002 | -1.84530291 |
| 8 | O | 5.34193035 | -0.24454362 | 0.77168835 |
| 9 | O | 5.92690232 | -1.93440766 | -1.00189850 |
| 10 | C | -0.54868945 | -2.50973939 | 0.77716189 |
| 11 | F | 6.24897747 | 1.89701252 | -1.24085412 |
| 12 | C | -0.30395655 | -3.77086244 | 0.19563224 |
| 13 | H | 0.04798542 | -3.83000888 | -0.82864805 |
| 14 | F | 7.86429858 | 0.43675627 | -0.84991973 |
| 15 | C | -1.12438756 | -1.85693975 | -2.25859980 |
| 16 | H | -2.03376645 | -2.32687911 | -1.89918501 |
| 17 | C | 1.22984055 | -0.58758109 | -3.20957213 |
| 18 | H | 2.13591391 | -0.10544218 | -3.57125843 |
| 19 | C | 0.27002265 | -1.07702780 | -4.10993524 |
| 20 | H | 0.42360583 | -0.96587276 | -5.18217519 |

| | | | | |
|---|---|---|---|---|
| 21 | H | 3.20213821 | -5.08468269 | 2.55388904 |
| 22 | H | 2.42029554 | 3.43860548 | 2.89155579 |
| 23 | N | 2.70445343 | -2.70646444 | 0.21440055 |
| 24 | C | -1.01588901 | -2.46927359 | 2.10619188 |
| 25 | H | -1.24944762 | -1.51552262 | 2.57337571 |
| 26 | C | -0.49570097 | -4.94285715 | 0.93001497 |
| 27 | H | -0.28700611 | -5.90297645 | 0.46086658 |
| 28 | C | -1.20323733 | -3.64434791 | 2.83990252 |
| 29 | H | -1.56410879 | -3.58156976 | 3.86515278 |
| 30 | C | -0.88947620 | -1.70974312 | -3.63210313 |
| 31 | H | -1.62796707 | -2.08313893 | -4.33969750 |
| 32 | C | -0.93946606 | -4.89122977 | 2.25825590 |
| 33 | H | -1.08312534 | -5.80681828 | 2.82876609 |
| 34 | C | 2.82029992 | -3.29424116 | 1.42490939 |
| 35 | C | 2.89310150 | -3.44084002 | -0.90291486 |
| 36 | C | 3.12340326 | -4.64651199 | 1.56264618 |
| 37 | C | 6.63923865 | 0.60370918 | -1.42830877 |
| 38 | C | 3.20176866 | -4.79849886 | -0.84367600 |
| 39 | F | 6.76170315 | 0.38247986 | -2.76884252 |
| 40 | Bi | -2.19940321 | 0.39075863 | 0.44862748 |
| 41 | S | -5.27416398 | -1.48954688 | -0.74128908 |
| 42 | N | 0.93532347 | 2.14166803 | 0.11033792 |
| 43 | H | 0.24678905 | 3.38315081 | 1.87553270 |
| 44 | O | -4.20921252 | -0.76092849 | -1.48442066 |
| 45 | C | 0.07497389 | 2.27418374 | -1.04793311 |
| 46 | C | -1.17606085 | 1.62422502 | -1.16537336 |
| 47 | O | -5.20344853 | -1.34873302 | 0.73438271 |
| 48 | O | -6.62823335 | -1.38195082 | -1.33127721 |
| 49 | C | 1.95300235 | 3.18012590 | 0.17947128 |
| 50 | F | -3.59485914 | -3.59689206 | -0.46505588 |
| 51 | C | 1.56027331 | 4.53274561 | 0.26369436 |
| 52 | H | 0.50584074 | 4.78859855 | 0.18832106 |
| 53 | F | -5.73863309 | -4.14006931 | -0.42940059 |
| 54 | C | 0.57358756 | 2.95715059 | -2.18757294 |
| 55 | H | 1.55019854 | 3.42709696 | -2.14729220 |
| 56 | C | -1.88624456 | 1.68770510 | -2.37768114 |
| 57 | H | -2.84225975 | 1.17414368 | -2.46106639 |
| 58 | C | -1.38699717 | 2.37242653 | -3.48672842 |
| 59 | H | -1.94780995 | 2.39401656 | -4.41941171 |
| 60 | C | -1.24285649 | 1.84175672 | 2.07196364 |
| 61 | H | -1.39639957 | 1.30202673 | 3.00813519 |
| 62 | H | -1.95300293 | 2.66617736 | 1.99652802 |
| 63 | C | 3.31707275 | 2.87318972 | 0.24865951 |
| 64 | H | 3.67136447 | 1.84963262 | 0.18422568 |
| 65 | C | 2.50788545 | 5.54261522 | 0.43038618 |
| 66 | H | 2.18240937 | 6.57906673 | 0.49903282 |
| 67 | C | 4.27105911 | 3.88775564 | 0.41355667 |
| 68 | H | 5.32365495 | 3.62235239 | 0.47211714 |
| 69 | C | -0.14207276 | 3.00275144 | -3.38266174 |
| 70 | H | 0.28799444 | 3.52225888 | -4.23766643 |
| 71 | C | 3.87252853 | 5.22269810 | 0.51269497 |
| 72 | H | 4.61310524 | 6.00853417 | 0.64860039 |
| 73 | C | 0.14641932 | 2.30393829 | 1.91913932 |

| | | | | |
|---|---|---|---|---|
| 74 | F | -4.76123739 | -3.61515033 | -2.34553066 |
| 75 | C | 3.31662885 | -5.41237395 | 0.40813534 |
| 76 | C | -4.81575160 | -3.31527140 | -1.01127097 |
| 77 | H | 2.66083762 | -2.65718063 | 2.29188873 |
| 78 | H | 2.78567703 | -2.91507675 | -1.84770754 |
| 79 | H | 3.34679255 | -5.35842920 | -1.76377154 |
| 80 | N | -4.02764019 | 2.24008716 | 0.32440067 |
| 81 | C | -5.28682764 | 1.95420633 | 0.72405823 |
| 82 | C | -3.74855327 | 3.48609202 | -0.11883489 |
| 83 | C | -6.30935059 | 2.90413304 | 0.68895487 |
| 84 | H | -5.46502820 | 0.93718096 | 1.06406400 |
| 85 | C | -4.71625350 | 4.48828489 | -0.18135340 |
| 86 | H | -2.72449936 | 3.66362836 | -0.43896084 |
| 87 | C | -6.02159127 | 4.19245276 | 0.22793630 |
| 88 | H | -7.30954913 | 2.62992421 | 1.01552451 |
| 89 | H | -4.44726557 | 5.47630115 | -0.54691301 |
| 90 | H | -6.79916682 | 4.95221083 | 0.18707104 |
| 91 | C | 1.18864762 | 1.66474336 | 2.76268176 |
| 92 | C | 1.07637828 | 0.31905050 | 3.17699374 |
| 93 | C | 2.08044966 | -0.28997868 | 3.93294738 |
| 94 | C | 3.22518386 | 0.43638591 | 4.29212080 |
| 95 | C | 3.33779827 | 1.77996037 | 3.91578551 |
| 96 | C | 2.32789301 | 2.39137545 | 3.16395121 |
| 97 | H | 0.19810149 | -0.25764448 | 2.91107857 |
| 98 | H | 1.96670354 | -1.32584943 | 4.24718604 |
| 99 | H | 4.01401247 | -0.03784393 | 4.87244153 |
| 100 | H | 4.21399275 | 2.35565717 | 4.20708189 |

**I-2** (-14069.3)

| | | | | |
|---|---|---|---|---|
| 1 | Bi | 1.78509213 | -0.63615568 | -0.68672541 |
| 2 | S | 4.85210560 | -0.34485374 | -2.39106009 |
| 3 | N | -0.37520238 | -1.63164744 | 0.05053846 |
| 4 | H | 4.19236114 | -6.28250343 | 0.65537720 |
| 5 | O | 3.39300528 | -0.67232236 | -2.64353592 |
| 6 | C | -0.56179506 | -2.10392377 | -1.30772961 |
| 7 | C | 0.48602370 | -1.75238209 | -2.16671931 |
| 8 | O | 5.23264864 | -0.45154792 | -0.97077283 |
| 9 | O | 5.75460816 | -0.93892189 | -3.39164018 |
| 10 | C | -0.42535959 | -2.61625515 | 1.07968100 |
| 11 | F | 3.98701610 | 2.17667389 | -1.97323829 |
| 12 | C | -0.22958762 | -3.99089574 | 0.81506118 |
| 13 | H | -0.07615268 | -4.32471940 | -0.20699944 |
| 14 | F | 6.13291843 | 2.02617019 | -2.49531960 |
| 15 | C | -1.69388560 | -2.76548719 | -1.80056531 |
| 16 | H | -2.51996844 | -3.02543451 | -1.14332028 |
| 17 | C | 0.44170965 | -2.04585086 | -3.52894656 |
| 18 | H | 1.25564751 | -1.75577997 | -4.19106473 |
| 19 | C | -0.68395793 | -2.72204824 | -4.02706221 |
| 20 | H | -0.74539131 | -2.96912045 | -5.08587942 |
| 21 | H | -5.38429810 | 4.75031730 | 1.39311777 |
| 22 | H | -4.01566403 | 4.70727850 | -2.71229522 |
| 23 | N | 2.75737323 | -2.78471119 | -0.14781978 |
| 24 | C | -0.61307451 | -2.21730745 | 2.41819737 |

| | | | | |
|---|---|---|---|---|
| 25 | H | -0.79182987 | -1.17108389 | 2.63827480 |
| 26 | C | -0.20772694 | -4.92021358 | 1.85823822 |
| 27 | H | -0.04456471 | -5.97215433 | 1.62855730 |
| 28 | C | -0.57712089 | -3.14908629 | 3.45949584 |
| 29 | H | -0.72038496 | -2.81057951 | 4.48454652 |
| 30 | C | -1.73975745 | -3.07084053 | -3.16678441 |
| 31 | H | -2.61614518 | -3.57408462 | -3.56985887 |
| 32 | C | -0.37187568 | -4.50917044 | 3.18817159 |
| 33 | H | -0.34606525 | -5.23559308 | 3.99813377 |
| 34 | C | 2.84941065 | -3.20205301 | 1.13439828 |
| 35 | C | 3.15687091 | -3.59963642 | -1.15324978 |
| 36 | C | 3.36496969 | -4.45370986 | 1.45988653 |
| 37 | C | 4.89396827 | 1.52065550 | -2.75624536 |
| 38 | C | 3.67868152 | -4.86391905 | -0.89602686 |
| 39 | F | 4.59083196 | 1.76613184 | -4.06089061 |
| 40 | Bi | -1.96197894 | 0.44864383 | 0.22723677 |
| 41 | S | -4.88863784 | 0.15963707 | -2.26924214 |
| 42 | H | -1.66067265 | 5.02813874 | -0.41341046 |
| 43 | H | -5.40949358 | 5.77576527 | -0.90751806 |
| 44 | O | -3.42416542 | 0.13572820 | -2.51278820 |
| 45 | H | 2.48037644 | 3.07364154 | 0.61872705 |
| 46 | H | 0.19575240 | 3.06644853 | 2.76059066 |
| 47 | O | -5.28613087 | 0.17687517 | -0.84051571 |
| 48 | O | -5.65372884 | 1.07243627 | -3.15217743 |
| 49 | C | -0.40388767 | 1.60093853 | -1.03694431 |
| 50 | F | -4.94432914 | -2.55202125 | -2.04849687 |
| 51 | C | -1.11502121 | 1.53900164 | 2.11708034 |
| 52 | C | -0.31661886 | 1.28012572 | -2.41415118 |
| 53 | F | -6.82241247 | -1.66436825 | -2.80390300 |
| 54 | C | 0.89115617 | 3.31250369 | -2.87465301 |
| 55 | H | 0.39926174 | 1.82393731 | -4.37843142 |
| 56 | H | 1.21010525 | 4.61928050 | -1.20078655 |
| 57 | C | -0.06460129 | 4.70822930 | 1.00409644 |
| 58 | H | 1.41837006 | 3.96956696 | -3.56420077 |
| 59 | C | -1.01422652 | 5.49472274 | 0.32281984 |
| 60 | C | 0.63815311 | 6.68604941 | 2.23919189 |
| 61 | H | 1.52700956 | 4.73559588 | 2.47681656 |
| 62 | C | -0.30544832 | 7.46451796 | 1.55706028 |
| 63 | C | 0.12008556 | 2.85147716 | -0.59434616 |
| 64 | C | 0.14535240 | 2.39017059 | 1.89824510 |
| 65 | H | -1.93726982 | 2.20458987 | 2.39857291 |
| 66 | H | -0.98974192 | 0.83112631 | 2.93849382 |
| 67 | C | 0.33549530 | 2.10804293 | -3.33026321 |
| 68 | H | -0.79389411 | 0.37571024 | -2.77451537 |
| 69 | C | 0.76850213 | 5.31925854 | 1.96028308 |
| 70 | C | -1.12696438 | 6.85963131 | 0.59242186 |
| 71 | C | 0.78026284 | 3.67917776 | -1.53636386 |
| 72 | N | 0.01252219 | 3.30758421 | 0.73731992 |
| 73 | H | -1.87166615 | 7.45012520 | 0.06074750 |
| 74 | N | -3.25642413 | 2.63811709 | -0.15724566 |
| 75 | C | -3.27006932 | 3.17994741 | -1.39473990 |
| 76 | C | -5.45580291 | -1.56444581 | -2.84636171 |
| 77 | C | -4.01233777 | 3.19807463 | 0.80982961 |

| | | | | |
|---|---|---|---|---|
| 78 | C | -4.03272430 | 4.30611794 | -1.70230882 |
| 79 | C | -4.79565365 | 4.32979904 | 0.58180999 |
| 80 | C | -4.80679125 | 4.89504567 | -0.69729798 |
| 81 | H | -2.65708354 | 2.68822810 | -2.14351880 |
| 82 | H | -3.98704304 | 2.72316408 | 1.78788783 |
| 83 | F | -5.05788209 | -1.81353983 | -4.13208067 |
| 84 | C | 3.78823838 | -5.29845052 | 0.43038094 |
| 85 | H | 2.49185036 | -2.51776337 | 1.89592640 |
| 86 | H | 3.04920959 | -3.21325090 | -2.16201904 |
| 87 | H | 3.99190195 | -5.49143473 | -1.72585174 |
| 88 | H | 3.41974826 | -4.75528295 | 2.50196449 |
| 89 | H | 1.28968243 | 7.14301489 | 2.98254014 |
| 90 | H | -0.40118959 | 8.52710699 | 1.77301682 |
| 91 | C | 1.48427360 | 1.64599619 | 1.90155005 |
| 92 | C | 1.67744327 | 0.49314698 | 2.68270925 |
| 93 | C | 2.93766192 | -0.11585054 | 2.76904752 |
| 94 | C | 4.02693273 | 0.40498957 | 2.06107713 |
| 95 | C | 3.85050185 | 1.56126655 | 1.29106310 |
| 96 | C | 2.59679087 | 2.17839601 | 1.22061584 |
| 97 | H | 0.85831124 | 0.07249268 | 3.25432679 |
| 98 | H | 3.06700868 | -0.98830384 | 3.40647918 |
| 99 | H | 4.99864755 | -0.08072833 | 2.10765332 |
| 100 | H | 4.68637912 | 1.97553072 | 0.73371189 |

**Product** (-9730.7)

| | | | | |
|---|---|---|---|---|
| 1 | C | 4.87629930 | -2.24635038 | -1.92174783 |
| 2 | C | 5.87706879 | 2.55203039 | 1.98118343 |
| 3 | H | 5.90815922 | -2.49755457 | -2.16449229 |
| 4 | C | 4.47882769 | 0.62902844 | 2.49848878 |
| 5 | H | 6.58862333 | 3.31349861 | 2.29563277 |
| 6 | Bi | 0.11819428 | -0.68533834 | -1.10999862 |
| 7 | S | -3.19332160 | 1.15525737 | -0.36829595 |
| 8 | N | 3.06588824 | -0.38305164 | 0.74651373 |
| 9 | C | -3.57868081 | 0.95269234 | 1.48527328 |
| 10 | O | -1.82361100 | 1.72886275 | -0.36760815 |
| 11 | C | 5.38909660 | 1.61407738 | 2.90113534 |
| 12 | C | 3.28276808 | -1.12527327 | -0.44067544 |
| 13 | C | 2.22337122 | -1.57470217 | -1.26450266 |
| 14 | H | 5.72962646 | 1.63539216 | 3.93553701 |
| 15 | O | -4.26088214 | 2.06961843 | -0.83625169 |
| 16 | O | -3.28151111 | -0.23972323 | -0.87211503 |
| 17 | C | 4.02355703 | 0.58239297 | 1.16558185 |
| 18 | F | -3.45667566 | 2.14216035 | 2.14738346 |
| 19 | C | 4.51569513 | 1.53010300 | 0.24537486 |
| 20 | H | 4.16689227 | 1.50133845 | -0.78226860 |
| 21 | H | 4.13357507 | -0.11395782 | 3.21435739 |
| 22 | C | 5.43774366 | 2.49786637 | 0.64857000 |
| 23 | H | 5.80109896 | 3.22613154 | -0.07538455 |
| 24 | F | -4.85277839 | 0.49195556 | 1.67210589 |
| 25 | C | 4.61081197 | -1.46931663 | -0.79490917 |
| 26 | H | 5.43299303 | -1.13220190 | -0.16799525 |
| 27 | C | 2.51299746 | -2.37132055 | -2.38615865 |
| 28 | H | 1.69800472 | -2.70738855 | -3.02920959 |

| | | | | |
|---|---|---|---|---|
| 29 | C | 3.82623787 | -2.72100727 | -2.72333800 |
| 30 | H | 4.02816711 | -3.33729917 | -3.59794497 |
| 31 | F | -2.72224716 | 0.05530534 | 2.07129288 |
| 32 | H | -2.54251029 | -2.11876447 | 0.21247832 |
| 33 | H | 0.34289662 | 2.69626888 | -0.60675601 |
| 34 | N | -0.67002632 | -2.92497177 | -0.18628428 |
| 35 | C | 0.13067410 | -4.01145539 | -0.14416306 |
| 36 | C | -0.30702066 | -5.23332220 | 0.36422370 |
| 37 | C | -1.61468259 | -5.33583795 | 0.84788681 |
| 38 | C | -2.44159541 | -4.20852437 | 0.80596055 |
| 39 | C | -1.93721596 | -3.02003527 | 0.27817966 |
| 40 | H | 1.14472581 | -3.87404942 | -0.50852510 |
| 41 | H | 0.37622067 | -6.07771030 | 0.39242054 |
| 42 | H | -3.46388055 | -4.24350067 | 1.17454317 |
| 43 | N | 1.40645343 | 1.43596675 | -1.86718634 |
| 44 | C | 2.37958964 | 1.32505192 | -2.79841107 |
| 45 | C | 3.13450184 | 2.41984370 | -3.22069883 |
| 46 | C | 2.88182766 | 3.67380100 | -2.65354675 |
| 47 | C | 1.87208981 | 3.78897470 | -1.69323926 |
| 48 | C | 1.15061571 | 2.64974739 | -1.33188339 |
| 49 | H | 2.56051023 | 0.32754129 | -3.19137376 |
| 50 | H | 3.90841038 | 2.28380905 | -3.97199888 |
| 51 | H | 1.63909620 | 4.74359394 | -1.22812366 |
| 52 | H | 3.46107495 | 4.54399394 | -2.95471178 |
| 53 | H | -1.98148174 | -6.27439737 | 1.25789289 |
| 54 | C | 1.88273977 | -0.60834701 | 1.62145610 |
| 55 | C | 0.60240226 | 0.00544706 | 1.03526537 |
| 56 | H | 3.73903313 | -2.66243129 | 1.53032518 |
| 57 | H | -0.27632620 | -0.19187634 | 1.64971846 |
| 58 | H | 2.10535778 | -0.01168946 | 2.51498673 |
| 59 | H | 0.71473237 | 1.08926886 | 0.95418828 |
| 60 | C | 1.75447896 | -2.05297910 | 2.12350981 |
| 61 | C | 0.59050673 | -2.46708348 | 2.79771770 |
| 62 | C | 0.49341912 | -3.74864763 | 3.34858885 |
| 63 | C | 1.56113794 | -4.64877602 | 3.23639496 |
| 64 | C | 2.72791904 | -4.24553561 | 2.57740318 |
| 65 | C | 2.82413895 | -2.95839459 | 2.03236774 |
| 66 | H | -0.25322168 | -1.78992635 | 2.89892648 |
| 67 | H | -0.42223613 | -4.04630649 | 3.85656739 |
| 68 | H | 1.48307684 | -5.65021665 | 3.65664908 |
| 69 | H | 3.56893758 | -4.93139822 | 2.48450075 |

***iso*-butene** (-1807.9)

| | | | | |
|---|---|---|---|---|
| 1 | H | 1.58127048 | -3.51766373 | 3.46347706 |
| 2 | H | 2.49222087 | -3.89780101 | -0.23774789 |
| 3 | H | 3.52884535 | -4.29375675 | 1.14861761 |
| 4 | C | 0.78770859 | -2.11595541 | 1.16421053 |
| 5 | C | -0.11686742 | -2.68926690 | 0.35879131 |
| 6 | H | 0.53637840 | -1.15726758 | 1.62933541 |
| 7 | H | 2.88831826 | -1.89269877 | 1.21115834 |
| 8 | H | 1.82251561 | -4.77991760 | 1.15192977 |
| 9 | C | 2.51824734 | -3.98280366 | 0.85584779 |
| 10 | C | 2.27624617 | -2.76000460 | 3.07601594 |

| | | | | |
|---|---|---|---|---|
| 11 | H | 2.04160568 | -1.80379126 | 3.56115197 |
| 12 | H | -1.07857491 | -2.21397347 | 0.16851524 |
| 13 | H | 0.06714628 | -3.64137973 | -0.13729922 |
| 14 | C | 2.15488158 | -2.65211608 | 1.53555275 |
| 15 | H | 3.29524285 | -3.04912329 | 3.36364874 |

**I-1** (-13651.7)

| | | | | |
|---|---|---|---|---|
| 1 | Bi | 2.07686860 | -0.73236153 | 0.06156261 |
| 2 | S | 5.37428521 | 0.16634959 | -0.40902487 |
| 3 | N | -0.36311149 | -1.56845278 | -0.25468540 |
| 4 | H | 2.57064112 | -7.18565825 | 0.49406130 |
| 5 | O | 4.12183993 | -0.16874414 | -1.20523485 |
| 6 | C | -0.04914980 | -1.67184940 | -1.66846365 |
| 7 | C | 1.23023948 | -1.20225809 | -1.98633068 |
| 8 | O | 5.13965988 | 0.17062127 | 1.04871213 |
| 9 | O | 6.57734455 | -0.51548356 | -0.90990376 |
| 10 | C | -0.83602663 | -2.73814345 | 0.42792473 |
| 11 | F | 4.53924081 | 2.73130476 | -0.54881203 |
| 12 | C | -0.70419778 | -4.02362426 | -0.13253918 |
| 13 | H | -0.28149665 | -4.14022436 | -1.12422234 |
| 14 | F | 6.70996719 | 2.49236579 | -0.19779722 |
| 15 | C | -0.92838566 | -2.08799369 | -2.67569511 |
| 16 | H | -1.92955087 | -2.43156195 | -2.43167014 |
| 17 | C | 1.65922440 | -1.11086236 | -3.30911165 |
| 18 | H | 2.64718022 | -0.72424578 | -3.55021079 |
| 19 | C | 0.78368290 | -1.52561004 | -4.32483385 |
| 20 | H | 1.09325007 | -1.46237646 | -5.36678043 |
| 21 | H | 2.23853043 | -3.25761921 | 2.24254873 |
| 22 | H | 2.72239657 | -3.57273294 | -1.86821701 |
| 23 | N | 2.49057582 | -3.32228062 | 0.17908837 |
| 24 | C | -1.37590761 | -2.61965952 | 1.72298325 |
| 25 | H | -1.49005175 | -1.63949574 | 2.17929457 |
| 26 | C | -1.09746315 | -5.15363728 | 0.58924518 |
| 27 | H | -0.97619436 | -6.13733064 | 0.13880084 |
| 28 | C | -1.76762047 | -3.75375429 | 2.43858606 |
| 29 | H | -2.18448820 | -3.63468630 | 3.43729493 |
| 30 | C | -0.49397725 | -2.01484149 | -4.00504572 |
| 31 | H | -1.16616603 | -2.32508987 | -4.80327372 |
| 32 | C | -1.63065462 | -5.03085614 | 1.87812643 |
| 33 | H | -1.93510881 | -5.91384553 | 2.43673826 |
| 34 | C | 2.36558488 | -3.91637717 | 1.38495065 |
| 35 | C | 2.64633239 | -4.09520352 | -0.91736049 |
| 36 | C | 2.38635629 | -5.30137801 | 1.54166912 |
| 37 | C | 5.63621693 | 1.99272692 | -0.87157335 |
| 38 | C | 2.68804911 | -5.48768602 | -0.84310356 |
| 39 | F | 5.86454366 | 2.11586948 | -2.20895329 |
| 40 | Bi | -1.50377685 | 0.47635201 | 0.20618279 |
| 41 | S | -4.67447626 | -0.70529213 | -0.50083472 |
| 42 | N | 0.93622831 | 1.43014685 | -0.05634091 |
| 43 | H | 2.81684688 | -6.07332870 | -1.74990108 |
| 44 | O | -3.36395856 | -0.38749193 | -1.19811566 |
| 45 | C | 0.53142279 | 1.81124767 | -1.38943991 |
| 46 | C | -0.73917413 | 1.35273295 | -1.75731670 |

| | | | | |
|---|---|---|---|---|
| 47 | O | -4.56039886 | -0.69323630 | 0.97097388 |
| 48 | O | -5.83016287 | -0.01988547 | -1.10106546 |
| 49 | C | 1.37752141 | 2.47976603 | 0.81166570 |
| 50 | F | -3.89204506 | -3.29049516 | -0.49423526 |
| 51 | C | 0.73204019 | 3.73494352 | 0.78105979 |
| 52 | H | -0.05068039 | 3.90954168 | 0.04906659 |
| 53 | F | -6.08247305 | -2.99374942 | -0.41488404 |
| 54 | C | 1.33172846 | 2.50845077 | -2.30666437 |
| 55 | H | 2.31885684 | 2.86591277 | -2.02143257 |
| 56 | C | -1.22433650 | 1.53557143 | -3.05275387 |
| 57 | H | -2.20478373 | 1.15868233 | -3.33740011 |
| 58 | C | -0.42621479 | 2.22064569 | -3.97982489 |
| 59 | H | -0.78432035 | 2.37770352 | -4.99604653 |
| 60 | H | -1.17516657 | 2.29324270 | 2.95326180 |
| 61 | H | 0.80509364 | -1.35119532 | 2.97514608 |
| 62 | H | 2.13525147 | -0.71831666 | 4.99209938 |
| 63 | C | 2.39983562 | 2.27956840 | 1.75592217 |
| 64 | H | 2.94637518 | 1.34134853 | 1.79376739 |
| 65 | C | 1.08722708 | 4.74517755 | 1.67736038 |
| 66 | H | 0.56856825 | 5.70196284 | 1.63817394 |
| 67 | C | 2.75090413 | 3.29171140 | 2.65395628 |
| 68 | H | 3.54281226 | 3.10757235 | 3.37767433 |
| 69 | C | 0.83625231 | 2.71130149 | -3.60075598 |
| 70 | H | 1.44593491 | 3.24457777 | -4.32871116 |
| 71 | C | 2.09774981 | 4.52959090 | 2.62567632 |
| 72 | H | 2.37206389 | 5.31452695 | 3.32778164 |
| 73 | H | 0.02604051 | 3.05989755 | 4.99643996 |
| 74 | H | 2.26990942 | -5.73663327 | 2.53065498 |
| 75 | H | 1.73185261 | 1.53385567 | 6.04534039 |
| 76 | C | -4.92016614 | -2.53314958 | -0.95912152 |
| 77 | N | -0.22899427 | 0.44448285 | 2.86436052 |
| 78 | C | -0.44155170 | 1.64820724 | 3.43589418 |
| 79 | C | 0.67985156 | -0.37187007 | 3.43734618 |
| 80 | C | 0.23284046 | 2.07711300 | 4.58112979 |
| 81 | C | 1.41044403 | -0.02304512 | 4.57515637 |
| 82 | C | 1.18028462 | 1.22742855 | 5.15926329 |
| 83 | F | -4.98816239 | -2.67823535 | -2.31410888 |
| 84 | C | 2.55232421 | -6.10160177 | 0.40676501 |
| 85 | C | -3.70589568 | 2.86469481 | 0.69036979 |
| 86 | H | -3.38604673 | 3.54747266 | 1.47590818 |
| 87 | H | -4.28391189 | 1.99806748 | 1.00604816 |
| 88 | C | -3.44767928 | 3.09580548 | -0.60873358 |
| 89 | H | -3.42138018 | 5.85826705 | 0.12707678 |
| 90 | H | -3.80824598 | 2.37041501 | -1.34208751 |
| 91 | C | -2.70749051 | 4.27624648 | -1.19756698 |
| 92 | H | -1.73034660 | 3.89438139 | -1.53548630 |
| 93 | C | -3.44033797 | 4.76979549 | -2.46756214 |
| 94 | H | -3.56732774 | 3.95269787 | -3.18811868 |
| 95 | H | -2.86680764 | 5.56791154 | -2.95421637 |
| 96 | H | -4.43370486 | 5.16436626 | -2.21502335 |
| 97 | C | -2.46791134 | 5.42580906 | -0.20393130 |
| 98 | H | -1.92262919 | 5.09611525 | 0.68737503 |
| 99 | H | -1.88007791 | 6.21971976 | -0.68001491 |

**TS-1** (-13634.7)

| | | | | |
|---|---|---|---|---|
| 1 | Bi | 1.96866414 | -0.32875400 | 0.07905541 |
| 2 | S | 5.36322898 | -0.67591144 | -0.59394693 |
| 3 | N | -0.29676601 | -1.32714927 | 0.00461232 |
| 4 | H | 3.33578388 | -6.55646087 | 0.46546609 |
| 5 | O | 4.12339882 | -0.25588403 | -1.35145566 |
| 6 | C | -0.16219370 | -1.41082386 | -1.44044897 |
| 7 | C | 1.00747038 | -0.80036927 | -1.90790675 |
| 8 | O | 5.27439793 | -0.38528742 | 0.85365187 |
| 9 | O | 5.89092023 | -1.99392316 | -0.98355560 |
| 10 | C | -0.51642994 | -2.53142812 | 0.74751427 |
| 11 | F | 6.28205035 | 1.83889646 | -1.00299488 |
| 12 | C | -0.44315457 | -3.81409008 | 0.17140931 |
| 13 | H | -0.23834417 | -3.91936585 | -0.88879520 |
| 14 | F | 7.86122902 | 0.32783882 | -0.66160386 |
| 15 | C | -1.11111967 | -1.90953279 | -2.34147067 |
| 16 | H | -2.02983787 | -2.37019634 | -1.99308792 |
| 17 | C | 1.25829822 | -0.64599711 | -3.26821292 |
| 18 | H | 2.16677200 | -0.16194142 | -3.62068891 |
| 19 | C | 0.30628994 | -1.13482497 | -4.17788943 |
| 20 | H | 0.47151760 | -1.02407927 | -5.24834857 |
| 21 | H | 3.00275552 | -5.15051660 | 2.52663878 |
| 22 | H | 0.46520136 | 0.52559872 | 4.27346815 |
| 23 | N | 2.64430933 | -2.76078963 | 0.17365787 |
| 24 | C | -0.76718103 | -2.42687237 | 2.13104866 |
| 25 | H | -0.81671630 | -1.44482099 | 2.59559883 |
| 26 | C | -0.60980884 | -4.95413135 | 0.96277929 |
| 27 | H | -0.53748789 | -5.93630464 | 0.49845182 |
| 28 | C | -0.93343162 | -3.57026398 | 2.91691184 |
| 29 | H | -1.12178450 | -3.46323376 | 3.98389890 |
| 30 | C | -0.86032242 | -1.76294962 | -3.71274182 |
| 31 | H | -1.59278172 | -2.13270873 | -4.42828792 |
| 32 | C | -0.85499024 | -4.84428909 | 2.33754138 |
| 33 | H | -0.98008749 | -5.73592018 | 2.94880926 |
| 34 | C | 2.71539744 | -3.34933432 | 1.38721276 |
| 35 | C | 2.82082103 | -3.50615877 | -0.93823616 |
| 36 | C | 2.96140167 | -4.71255997 | 1.53312405 |
| 37 | C | 6.65492094 | 0.55212641 | -1.25799415 |
| 38 | C | 3.07569884 | -4.87467747 | -0.87087645 |
| 39 | F | 6.80566095 | 0.40743713 | -2.60549839 |
| 40 | Bi | -2.20975547 | 0.30124496 | 0.38146744 |
| 41 | S | -5.31727043 | -1.45274635 | -0.89209357 |
| 42 | N | 0.92699113 | 2.06448840 | 0.03361086 |
| 43 | H | -0.06058897 | 3.32012933 | 1.65507753 |
| 44 | O | -4.22100115 | -0.73444786 | -1.60078721 |
| 45 | C | 0.06678464 | 2.19636666 | -1.11796434 |
| 46 | C | -1.19750177 | 1.56891068 | -1.21669779 |
| 47 | O | -5.28259997 | -1.32255703 | 0.58585180 |
| 48 | O | -6.65241489 | -1.31891707 | -1.51731048 |
| 49 | C | 1.90781360 | 3.13087263 | 0.13019631 |
| 50 | F | -3.67530177 | -3.58310025 | -0.58320287 |
| 51 | C | 1.47579922 | 4.47342817 | 0.19339482 |

| | | | | |
|---|---|---|---|---|
| 52 | H | 0.41916996 | 4.70163348 | 0.07706478 |
| 53 | F | -5.82559898 | -4.10026880 | -0.62206290 |
| 54 | C | 0.56078205 | 2.87337445 | -2.26359094 |
| 55 | H | 1.54748679 | 3.32394549 | -2.23295621 |
| 56 | C | -1.92806215 | 1.65624746 | -2.41655128 |
| 57 | H | -2.89553886 | 1.16335553 | -2.48794627 |
| 58 | C | -1.43330887 | 2.33698616 | -3.52975810 |
| 59 | H | -2.01069182 | 2.37691187 | -4.45172324 |
| 60 | C | -1.20336282 | 1.57916904 | 2.13022343 |
| 61 | H | -1.17198953 | 0.86133641 | 2.94930921 |
| 62 | H | -2.03619858 | 2.27484974 | 2.25237909 |
| 63 | C | 3.28130934 | 2.86708081 | 0.24046359 |
| 64 | H | 3.66569913 | 1.85413407 | 0.16593658 |
| 65 | C | 2.38846311 | 5.51129761 | 0.38324362 |
| 66 | H | 2.03011004 | 6.53800656 | 0.43377267 |
| 67 | C | 4.19849197 | 3.90970514 | 0.43495799 |
| 68 | H | 5.25699955 | 3.67735875 | 0.52272228 |
| 69 | C | -0.17219597 | 2.93932056 | -3.44606286 |
| 70 | H | 0.25361117 | 3.45261507 | -4.30695701 |
| 71 | C | 3.75779333 | 5.23352070 | 0.51327364 |
| 72 | H | 4.47030599 | 6.04171107 | 0.66696946 |
| 73 | C | 0.05471330 | 2.26731154 | 1.88939476 |
| 74 | F | -4.78082239 | -3.56969423 | -2.49991232 |
| 75 | C | 3.14435634 | -5.48890570 | 0.38414526 |
| 76 | C | -4.87553202 | -3.28176820 | -1.16589636 |
| 77 | H | 2.56180910 | -2.70771311 | 2.25225341 |
| 78 | H | 2.74832429 | -2.98052359 | -1.88644154 |
| 79 | H | 3.21360878 | -5.44269100 | -1.78718120 |
| 80 | N | -3.95113309 | 2.22939335 | 0.37278863 |
| 81 | C | -5.22779718 | 1.98056419 | 0.74121318 |
| 82 | C | -3.60366494 | 3.48800608 | 0.02306440 |
| 83 | C | -6.20022440 | 2.98210038 | 0.76533774 |
| 84 | H | -5.46108900 | 0.95183408 | 1.00415426 |
| 85 | C | -4.51705208 | 4.54145382 | 0.02937156 |
| 86 | H | -2.56896074 | 3.63321934 | -0.27878465 |
| 87 | C | -5.84077032 | 4.28433819 | 0.40479732 |
| 88 | H | -7.21730226 | 2.73706318 | 1.06141621 |
| 89 | H | -4.19354292 | 5.53844819 | -0.25948452 |
| 90 | H | -6.57744233 | 5.08474412 | 0.41516143 |
| 91 | C | 1.22831942 | 1.99673650 | 2.82845981 |
| 92 | H | 2.16324885 | 2.11281853 | 2.27127166 |
| 93 | C | 1.24142877 | 3.11354724 | 3.90778770 |
| 94 | H | 1.35323529 | 4.10113318 | 3.44642537 |
| 95 | H | 2.08202744 | 2.95342250 | 4.59360196 |
| 96 | H | 0.31351262 | 3.10383096 | 4.49368730 |
| 97 | C | 1.23950346 | 0.61048919 | 3.50167380 |
| 98 | H | 1.07941538 | -0.21160717 | 2.79364734 |
| 99 | H | 2.20922436 | 0.44463358 | 3.98350710 |

**I-2** (-13650.3)

| | | | | |
|---|---|---|---|---|
| 1 | Bi | 1.72961598 | -0.51670429 | -1.10635184 |
| 2 | S | 4.90695702 | -0.49135706 | -2.39012109 |
| 3 | N | -0.36539475 | -1.55183234 | -0.16452413 |

| | | | | |
|---|---|---|---|---|
| 4 | H | 4.03533601 | -5.70004605 | 1.50823623 |
| 5 | O | 3.51195082 | -0.87919970 | -2.85307981 |
| 6 | C | -0.62149026 | -2.06646692 | -1.49462583 |
| 7 | C | 0.38350931 | -1.76312564 | -2.42357987 |
| 8 | O | 4.97831224 | -0.25535771 | -0.93380460 |
| 9 | O | 5.96763111 | -1.29826568 | -3.01169477 |
| 10 | C | -0.27923207 | -2.51993908 | 0.87809543 |
| 11 | F | 4.12095274 | 2.06739934 | -2.67309193 |
| 12 | C | -0.12703956 | -3.90232157 | 0.62561680 |
| 13 | H | -0.11468881 | -4.26605929 | -0.39706936 |
| 14 | F | 6.31007993 | 1.76820156 | -2.81797937 |
| 15 | C | -1.77873050 | -2.74178143 | -1.90418911 |
| 16 | H | -2.57200724 | -2.96795662 | -1.19556745 |
| 17 | C | 0.26835491 | -2.11069090 | -3.76964272 |
| 18 | H | 1.04791542 | -1.85783869 | -4.48622255 |
| 19 | C | -0.88341340 | -2.80166371 | -4.17890517 |
| 20 | H | -0.99926476 | -3.09367059 | -5.22143045 |
| 21 | H | -5.45863287 | 4.72662805 | 1.50178575 |
| 22 | H | -4.08874895 | 4.81682493 | -2.60263703 |
| 23 | N | 2.71729339 | -2.47687439 | -0.15764455 |
| 24 | C | -0.27503538 | -2.08827573 | 2.21961792 |
| 25 | H | -0.39166075 | -1.03330124 | 2.43227010 |
| 26 | C | 0.03174956 | -4.80444501 | 1.68097679 |
| 27 | H | 0.16022291 | -5.86268339 | 1.45818007 |
| 28 | C | -0.11211283 | -2.99338372 | 3.27108289 |
| 29 | H | -0.10693523 | -2.62622867 | 4.29624547 |
| 30 | C | -1.89404231 | -3.10717285 | -3.25111276 |
| 31 | H | -2.79038078 | -3.62407706 | -3.58648207 |
| 32 | C | 0.04418111 | -4.36129528 | 3.01074713 |
| 33 | H | 0.17435013 | -5.06829754 | 3.82763791 |
| 34 | C | 2.95613038 | -2.50145734 | 1.17411687 |
| 35 | C | 2.93884269 | -3.58055806 | -0.90784635 |
| 36 | C | 3.42765655 | -3.64669246 | 1.80642883 |
| 37 | C | 5.09879158 | 1.24073700 | -3.14816239 |
| 38 | C | 3.42247880 | -4.75478248 | -0.33765003 |
| 39 | F | 4.99994754 | 1.19055667 | -4.50536759 |
| 40 | Bi | -2.06059108 | 0.45796002 | 0.17680849 |
| 41 | S | -4.89137379 | 0.27787511 | -2.54929879 |
| 42 | H | -1.55990645 | 4.90609287 | -0.36153567 |
| 43 | H | -5.46195289 | 5.84206941 | -0.75643197 |
| 44 | O | -3.41363569 | 0.18746326 | -2.66066841 |
| 45 | H | 1.84096087 | 1.56250218 | 4.11350782 |
| 46 | H | 0.17205638 | 2.85583107 | 2.88577851 |
| 47 | O | -5.41163944 | 0.38032176 | -1.16502451 |
| 48 | O | -5.53800869 | 1.17354627 | -3.53894699 |
| 49 | C | -0.49448721 | 1.65540825 | -1.02077699 |
| 50 | F | -5.05490020 | -2.41612568 | -2.21812509 |
| 51 | C | -1.25953792 | 1.47745114 | 2.12892366 |
| 52 | C | -0.38242846 | 1.39611969 | -2.41111507 |
| 53 | F | -6.84450889 | -1.50612650 | -3.14397821 |
| 54 | C | 1.05534622 | 3.31186473 | -2.70744224 |
| 55 | H | 0.47489486 | 1.96609795 | -4.31296580 |
| 56 | H | 1.44051277 | 4.47811607 | -0.94323544 |

| | | | | |
|---|---|---|---|---|
| 57 | C | -0.08549449 | 4.57645695 | 1.18222379 |
| 58 | H | 1.67621760 | 3.94446973 | -3.33890181 |
| 59 | C | -0.99546878 | 5.36262372 | 0.44529661 |
| 60 | C | 0.43075360 | 6.53022227 | 2.54550856 |
| 61 | H | 1.36021625 | 4.60415053 | 2.79948398 |
| 62 | C | -0.47251148 | 7.30624015 | 1.80720465 |
| 63 | C | 0.14939592 | 2.80495453 | -0.48425150 |
| 64 | C | 0.07740618 | 2.22017179 | 1.99857703 |
| 65 | H | -2.05207536 | 2.19318659 | 2.36953713 |
| 66 | H | -1.24001538 | 0.73944273 | 2.93492899 |
| 67 | C | 0.39678255 | 2.19754102 | -3.25288688 |
| 68 | H | -0.94041305 | 0.56876667 | -2.83944450 |
| 69 | C | 0.63315812 | 5.18016763 | 2.23322722 |
| 70 | C | -1.17977464 | 6.71219131 | 0.75025066 |
| 71 | C | 0.92821219 | 3.61190085 | -1.35484318 |
| 72 | N | 0.06595732 | 3.19354986 | 0.87054662 |
| 73 | H | -1.89414479 | 7.29738726 | 0.17286937 |
| 74 | N | -3.36402652 | 2.64120001 | -0.12731832 |
| 75 | C | -3.37002559 | 3.22883267 | -1.34372620 |
| 76 | C | -5.47553720 | -1.45073017 | -3.09305395 |
| 77 | C | -4.11242233 | 3.17523719 | 0.86012589 |
| 78 | C | -4.11281701 | 4.37855304 | -1.60834628 |
| 79 | C | -4.87616804 | 4.32798164 | 0.67509424 |
| 80 | C | -4.87567100 | 4.94291909 | -0.58076719 |
| 81 | H | -2.76613111 | 2.75542708 | -2.11165522 |
| 82 | H | -4.09612454 | 2.66305208 | 1.81924174 |
| 83 | F | -4.99805926 | -1.77022095 | -4.33503348 |
| 84 | C | 3.66650270 | -4.79117991 | 1.03927480 |
| 85 | H | 2.75661234 | -1.58796345 | 1.72672566 |
| 86 | H | 2.71735944 | -3.49917525 | -1.96772695 |
| 87 | H | 3.59489178 | -5.62314413 | -0.96723612 |
| 88 | H | 3.59910993 | -3.63576424 | 2.87870324 |
| 89 | H | 0.99663719 | 6.97824993 | 3.36108872 |
| 90 | H | -0.62391844 | 8.35633155 | 2.05068834 |
| 91 | C | 1.31974638 | 1.28248238 | 2.01834461 |
| 92 | H | 1.08867149 | 0.41982366 | 1.37222961 |
| 93 | C | 2.60478498 | 1.95871203 | 1.50863861 |
| 94 | H | 2.52050938 | 2.29654628 | 0.47226264 |
| 95 | H | 3.45153438 | 1.26399963 | 1.57009247 |
| 96 | H | 2.85019741 | 2.83350662 | 2.12345042 |
| 97 | C | 1.55056700 | 0.74134213 | 3.44471222 |
| 98 | H | 0.66209779 | 0.26367920 | 3.86783953 |
| 99 | H | 2.36241758 | 0.00409495 | 3.45472571 |

**Product** (-9316.7)

| | | | | |
|---|---|---|---|---|
| 1 | C | 5.09661841 | -2.56052018 | 2.62936573 |
| 2 | C | 5.14217423 | 2.94125157 | -1.08204846 |
| 3 | H | 5.92949588 | -2.61087580 | 3.32905580 |
| 4 | C | 3.06584113 | 2.25655689 | -0.00107833 |
| 5 | H | 5.71744644 | 3.67317450 | -1.64560626 |
| 6 | Bi | 1.25154481 | -2.30011396 | -0.74420564 |
| 7 | S | -1.49026688 | -1.47361250 | -3.30503241 |
| 8 | N | 2.89579532 | 0.05948480 | 1.11442480 |

| | | | | |
|---|---|---|---|---|
| 9 | C | -2.74399371 | -0.23048835 | -2.59313730 |
| 10 | O | -0.19048632 | -0.77380129 | -3.14626605 |
| 11 | C | 3.81623354 | 3.19712097 | -0.71411234 |
| 12 | C | 3.46316991 | -1.23105630 | 1.40300691 |
| 13 | C | 2.93987036 | -2.40580087 | 0.82530184 |
| 14 | H | 3.34369428 | 4.13869350 | -0.99246112 |
| 15 | O | -1.94704506 | -1.65044821 | -4.70330327 |
| 16 | O | -1.66779818 | -2.65646702 | -2.42296861 |
| 17 | C | 3.62855395 | 1.01192011 | 0.38559499 |
| 18 | F | -2.66926155 | 0.97072904 | -3.24249395 |
| 19 | C | 4.96628005 | 0.75757785 | -0.01458653 |
| 20 | H | 5.42436172 | -0.19737720 | 0.21627167 |
| 21 | H | 2.03638645 | 2.49996820 | 0.23747132 |
| 22 | C | 5.70014086 | 1.70489995 | -0.72747944 |
| 23 | H | 6.71944081 | 1.46135165 | -1.02486946 |
| 24 | F | -4.02289814 | -0.70066167 | -2.71928803 |
| 25 | C | 4.53599113 | -1.32000968 | 2.30853411 |
| 26 | H | 4.92950590 | -0.40920227 | 2.75556867 |
| 27 | C | 3.50332450 | -3.64649738 | 1.16619021 |
| 28 | H | 3.11223779 | -4.56195429 | 0.72032960 |
| 29 | C | 4.57856993 | -3.73282940 | 2.06200225 |
| 30 | H | 5.00745804 | -4.70223401 | 2.31210720 |
| 31 | F | -2.51200348 | -0.00443980 | -1.26211767 |
| 32 | H | -1.94457950 | -2.84252345 | -0.19285464 |
| 33 | H | 1.89421703 | 0.49636800 | -2.64195642 |
| 34 | N | -0.34290097 | -3.30770641 | 1.04427180 |
| 35 | C | 0.06319449 | -3.80984632 | 2.23024024 |
| 36 | C | -0.83783077 | -4.24312437 | 3.20319184 |
| 37 | C | -2.20923485 | -4.15698142 | 2.94035801 |
| 38 | C | -2.62986666 | -3.65016950 | 1.70644098 |
| 39 | C | -1.66862170 | -3.23712567 | 0.78289632 |
| 40 | H | 1.13734965 | -3.84919140 | 2.39479193 |
| 41 | H | -0.46553531 | -4.63374607 | 4.14700041 |
| 42 | H | -3.68529331 | -3.57030414 | 1.45797432 |
| 43 | N | 3.05034702 | -1.14970492 | -2.12640890 |
| 44 | C | 4.23791809 | -1.78178341 | -2.25170417 |
| 45 | C | 5.27782211 | -1.26391318 | -3.02166596 |
| 46 | C | 5.08928065 | -0.04124826 | -3.67390760 |
| 47 | C | 3.86453809 | 0.61663781 | -3.53542247 |
| 48 | C | 2.86657730 | 0.02953079 | -2.75880207 |
| 49 | H | 4.34813982 | -2.71777942 | -1.70873818 |
| 50 | H | 6.21613556 | -1.80782346 | -3.09611124 |
| 51 | H | 3.67888093 | 1.57443658 | -4.01348191 |
| 52 | H | 5.88605364 | 0.39460294 | -4.27250735 |
| 53 | H | -2.93594698 | -4.47931696 | 3.68290583 |
| 54 | C | 1.42286487 | 0.22261153 | 1.31288585 |
| 55 | C | 0.63937011 | -0.21743935 | 0.06721451 |
| 56 | H | -0.87354881 | 0.78730946 | 2.78143799 |
| 57 | H | -0.43983349 | -0.23446961 | 0.22417390 |
| 58 | H | 1.26153662 | 1.30051322 | 1.43696336 |
| 59 | H | 0.83995651 | 0.45380961 | -0.77116176 |
| 60 | C | 0.96681552 | -0.39353210 | 2.66433728 |
| 61 | H | 1.21829965 | -1.45936098 | 2.66907478 |

| | | | | |
|---|---|---|---|---|
| 62 | C | 1.70491154 | 0.28208553 | 3.83764707 |
| 63 | H | 2.79075599 | 0.19498561 | 3.74215815 |
| 64 | H | 1.40746978 | -0.17571043 | 4.78933417 |
| 65 | H | 1.45272629 | 1.35116387 | 3.88386887 |
| 66 | C | -0.55620765 | -0.26184016 | 2.86215272 |
| 67 | H | -1.12572539 | -0.84647713 | 2.13490397 |
| 68 | H | -0.83300226 | -0.61942225 | 3.86137421 |

**Cyclopentene** (-1633.8)

| | | | | |
|---|---|---|---|---|
| 1 | C | -3.17905447 | 0.47400188 | 0.19835138 |
| 2 | C | -2.91630669 | -0.81010242 | -0.08733495 |
| 3 | C | -1.42616363 | -1.10519321 | -0.07487663 |
| 4 | C | -0.78143350 | 0.31613300 | -0.03690926 |
| 5 | C | -1.91277191 | 1.27338241 | 0.45406021 |
| 6 | H | -1.81632692 | 1.50225220 | 1.52830258 |
| 7 | H | -1.90548006 | 2.23755573 | -0.07251299 |
| 8 | H | -1.10179382 | -1.69070917 | -0.94601432 |
| 9 | H | -1.16178929 | -1.69737749 | 0.81681611 |
| 10 | H | -0.47748048 | 0.60390167 | -1.05139659 |
| 11 | H | 0.10939613 | 0.35684316 | 0.59946583 |
| 12 | H | -4.17656102 | 0.90431390 | 0.28400832 |
| 13 | H | -3.66880118 | -1.57730202 | -0.26805731 |

**I-1** (-13478.5)

| | | | | |
|---|---|---|---|---|
| 1 | Bi | 2.08497074 | -0.71243687 | 0.03563072 |
| 2 | S | 5.36930536 | 0.20875054 | -0.42007489 |
| 3 | N | -0.36205196 | -1.54213504 | -0.27715141 |
| 4 | H | 2.56715572 | -7.16854794 | 0.47934251 |
| 5 | O | 4.12377563 | -0.12370947 | -1.22882304 |
| 6 | C | -0.04776464 | -1.64951870 | -1.69033395 |
| 7 | C | 1.23317126 | -1.18489113 | -2.00982714 |
| 8 | O | 5.12455393 | 0.18854492 | 1.03576194 |
| 9 | O | 6.57973570 | -0.45766326 | -0.92397242 |
| 10 | C | -0.84122004 | -2.71018533 | 0.40451348 |
| 11 | F | 4.51830711 | 2.77054351 | -0.52396066 |
| 12 | C | -0.71014610 | -3.99665572 | -0.15435686 |
| 13 | H | -0.28557568 | -4.11514445 | -1.14497715 |
| 14 | F | 6.68835737 | 2.53909524 | -0.16466821 |
| 15 | C | -0.92748985 | -2.06752733 | -2.69657520 |
| 16 | H | -1.93047117 | -2.40545382 | -2.45135692 |
| 17 | C | 1.66338643 | -1.10167538 | -3.33284531 |
| 18 | H | 2.65254819 | -0.71886176 | -3.57513519 |
| 19 | C | 0.78802990 | -1.52011693 | -4.34710377 |
| 20 | H | 1.09885659 | -1.46367974 | -5.38906605 |
| 21 | H | 2.24804456 | -3.23797013 | 2.22370739 |
| 22 | H | 2.70383052 | -3.55957162 | -1.88979096 |
| 23 | N | 2.48433621 | -3.30544513 | 0.15839279 |
| 24 | C | -1.38405352 | -2.59041892 | 1.69832332 |
| 25 | H | -1.49612403 | -1.60993726 | 2.15422932 |
| 26 | C | -1.10421987 | -5.12583481 | 0.56844701 |
| 27 | H | -0.98236278 | -6.11006016 | 0.11928863 |
| 28 | C | -1.77578427 | -3.72365679 | 2.41521752 |
| 29 | H | -2.19340974 | -3.60319144 | 3.41345790 |

| | | | | |
|---|---|---|---|---|
| 30 | C | -0.49155353 | -2.00341496 | -4.02584430 |
| 31 | H | -1.16391990 | -2.31596539 | -4.82303181 |
| 32 | C | -1.63846631 | -5.00151981 | 1.85672756 |
| 33 | H | -1.94296120 | -5.88385732 | 2.41635723 |
| 34 | C | 2.36753107 | -3.89779326 | 1.36596723 |
| 35 | C | 2.63319007 | -4.08038546 | -0.93762512 |
| 36 | C | 2.38889134 | -5.28247735 | 1.52486801 |
| 37 | C | 5.62155678 | 2.04378861 | -0.85234696 |
| 38 | C | 2.67605122 | -5.47272239 | -0.86126258 |
| 39 | F | 5.85657361 | 2.19011143 | -2.18621653 |
| 40 | Bi | -1.50101038 | 0.50764135 | 0.18108185 |
| 41 | S | -4.68092509 | -0.63795200 | -0.48328335 |
| 42 | N | 0.93459751 | 1.44854172 | -0.07888914 |
| 43 | H | 2.80012124 | -6.05970276 | -1.76784540 |
| 44 | O | -3.35698749 | -0.40855333 | -1.19059429 |
| 45 | C | 0.53817142 | 1.82328981 | -1.41712932 |
| 46 | C | -0.73709769 | 1.37546295 | -1.78293160 |
| 47 | O | -4.57379170 | -0.52792372 | 0.98499522 |
| 48 | O | -5.81088440 | 0.04128896 | -1.13764609 |
| 49 | C | 1.37071466 | 2.50102962 | 0.78702852 |
| 50 | F | -3.99134456 | -3.24294375 | -0.28618047 |
| 51 | C | 0.76433558 | 3.77401332 | 0.71333823 |
| 52 | H | 0.02021852 | 3.96227650 | -0.05478619 |
| 53 | F | -6.17024525 | -2.86625141 | -0.26900595 |
| 54 | C | 1.35222134 | 2.49499206 | -2.34073834 |
| 55 | H | 2.34373755 | 2.84122884 | -2.05709815 |
| 56 | C | -1.21610160 | 1.54890194 | -3.08196617 |
| 57 | H | -2.20093175 | 1.18169430 | -3.36462765 |
| 58 | C | -0.40332647 | 2.20672795 | -4.01645816 |
| 59 | H | -0.75406118 | 2.35089300 | -5.03726171 |
| 60 | H | -1.14836630 | 2.23466285 | 3.08411986 |
| 61 | H | 0.83333776 | -1.40189360 | 2.89212559 |
| 62 | H | 2.22048764 | -0.85450139 | 4.89529892 |
| 63 | C | 2.35235243 | 2.28714153 | 1.77150196 |
| 64 | H | 2.86613990 | 1.33269889 | 1.84482999 |
| 65 | C | 1.11696477 | 4.78738176 | 1.60806427 |
| 66 | H | 0.62870048 | 5.75816808 | 1.53560395 |
| 67 | C | 2.70117446 | 3.30247088 | 2.66545332 |
| 68 | H | 3.46233703 | 3.10818291 | 3.41872717 |
| 69 | C | 0.86568729 | 2.68308360 | -3.64046532 |
| 70 | H | 1.48737866 | 3.19301423 | -4.37495136 |
| 71 | C | 2.08492704 | 4.55810065 | 2.59581479 |
| 72 | H | 2.35730101 | 5.34614013 | 3.29518447 |
| 73 | H | 0.09686738 | 2.90766861 | 5.13242726 |
| 74 | H | 2.27961618 | -5.71594888 | 2.51545936 |
| 75 | H | 1.83876449 | 1.34501334 | 6.06210488 |
| 76 | C | -4.98292685 | -2.48421423 | -0.82050239 |
| 77 | N | -0.20086224 | 0.39647899 | 2.88317578 |
| 78 | C | -0.40105644 | 1.57024605 | 3.51598415 |
| 79 | C | 0.72242477 | -0.44259817 | 3.39780165 |
| 80 | C | 0.29929263 | 1.94783197 | 4.66423367 |
| 81 | C | 1.48363135 | -0.14322503 | 4.52944521 |
| 82 | C | 1.26554252 | 1.07800456 | 5.17702536 |

| | | | | |
|---|---|---|---|---|
| 83 | F | -5.03380522 | -2.71917751 | -2.16390227 |
| 84 | C | 2.54755009 | -6.08466827 | 0.39026436 |
| 85 | H | -3.29907461 | 4.59430814 | 2.22454798 |
| 86 | H | -1.79182055 | 3.82828021 | 1.73056561 |
| 87 | C | -3.59863685 | 3.04914410 | -0.57572194 |
| 88 | C | -2.88010467 | 4.27120102 | -1.11205738 |
| 89 | H | -4.08768425 | 2.32165176 | -1.21936113 |
| 90 | C | -3.54701330 | 2.98118552 | 0.76914558 |
| 91 | C | -2.64351163 | 5.13602483 | 0.16476110 |
| 92 | H | -3.46994540 | 4.79568278 | -1.87469506 |
| 93 | H | -1.93957787 | 3.97156548 | -1.59882039 |
| 94 | C | -2.78046772 | 4.14959465 | 1.36598034 |
| 95 | H | -4.00984481 | 2.20082985 | 1.37117298 |
| 96 | H | -3.42632154 | 5.90037890 | 0.23603928 |
| 97 | H | -1.67953878 | 5.65422394 | 0.15185444 |

**TS-1** (-13461.6)

| | | | | |
|---|---|---|---|---|
| 1 | Bi | 1.93744339 | -0.25757674 | 0.17520279 |
| 2 | S | 5.36027161 | -0.58279999 | -0.42184214 |
| 3 | N | -0.32370507 | -1.25930465 | 0.03423989 |
| 4 | H | 3.31292781 | -6.48606980 | 0.64612087 |
| 5 | O | 4.12197070 | -0.21595862 | -1.20730367 |
| 6 | C | -0.15309037 | -1.33119683 | -1.40810852 |
| 7 | C | 1.04635050 | -0.75487010 | -1.83831290 |
| 8 | O | 5.25974712 | -0.21561327 | 1.00785258 |
| 9 | O | 5.90344261 | -1.91489375 | -0.73545453 |
| 10 | C | -0.54736029 | -2.48195910 | 0.74684064 |
| 11 | F | 6.26465478 | 1.91411474 | -0.95989486 |
| 12 | C | -0.46027122 | -3.75001079 | 0.13977944 |
| 13 | H | -0.24104743 | -3.82870120 | -0.91962421 |
| 14 | F | 7.85091142 | 0.43454992 | -0.52818670 |
| 15 | C | -1.08727181 | -1.80562679 | -2.33699905 |
| 16 | H | -2.03086421 | -2.23465178 | -2.01495641 |
| 17 | C | 1.34636739 | -0.61341774 | -3.19012767 |
| 18 | H | 2.27766878 | -0.15316084 | -3.51363250 |
| 19 | C | 0.41300060 | -1.08355363 | -4.12819797 |
| 20 | H | 0.61688586 | -0.98384159 | -5.19310203 |
| 21 | H | 2.86042836 | -5.08186332 | 2.68561047 |
| 22 | H | -6.76258319 | 5.00215307 | 0.36857952 |
| 23 | N | 2.62726427 | -2.69197465 | 0.31691082 |
| 24 | C | -0.80953602 | -2.41702534 | 2.12913852 |
| 25 | H | -0.85771848 | -1.44931611 | 2.61929172 |
| 26 | C | -0.63528420 | -4.91031786 | 0.89879809 |
| 27 | H | -0.55330067 | -5.87961909 | 0.40968383 |
| 28 | C | -0.98359762 | -3.57983967 | 2.88386698 |
| 29 | H | -1.18226662 | -3.49998385 | 3.95140018 |
| 30 | C | -0.78654804 | -1.67516178 | -3.69956871 |
| 31 | H | -1.50518606 | -2.02669019 | -4.43800510 |
| 32 | C | -0.89889963 | -4.83796480 | 2.27254841 |
| 33 | H | -1.02978988 | -5.74560309 | 2.85845461 |
| 34 | C | 2.63158723 | -3.28110278 | 1.53205316 |
| 35 | C | 2.86774752 | -3.43667928 | -0.78323995 |
| 36 | C | 2.87376638 | -4.64365346 | 1.69143868 |

| | | | | |
|---|---|---|---|---|
| 37 | C | 6.64636748 | 0.61883001 | -1.14284458 |
| 38 | C | 3.12232497 | -4.80453498 | -0.70235232 |
| 39 | F | 6.80734842 | 0.40243478 | -2.47998696 |
| 40 | Bi | -2.17894227 | 0.42835347 | 0.45009884 |
| 41 | S | -5.23323070 | -1.42061733 | -0.69183088 |
| 42 | N | 0.94701443 | 2.13173010 | -0.04209235 |
| 43 | H | -4.45126160 | 5.50953990 | -0.49341238 |
| 44 | O | -4.14268846 | -0.71602987 | -1.42740930 |
| 45 | C | 0.08348149 | 2.22718246 | -1.18434026 |
| 46 | C | -1.20373706 | 1.63600375 | -1.22146876 |
| 47 | O | -5.13321953 | -1.32569598 | 0.78533607 |
| 48 | O | -6.58560507 | -1.22945925 | -1.26074299 |
| 49 | C | 1.93870614 | 3.17740875 | 0.04400289 |
| 50 | F | -3.63319745 | -3.59650574 | -0.53378121 |
| 51 | C | 1.54754061 | 4.53320359 | -0.05268474 |
| 52 | H | 0.51612156 | 4.77566325 | -0.29618933 |
| 53 | F | -5.79541235 | -4.05726152 | -0.44900103 |
| 54 | C | 0.56825171 | 2.83212095 | -2.37274051 |
| 55 | H | 1.57238520 | 3.24472417 | -2.38496811 |
| 56 | C | -1.96385900 | 1.69329916 | -2.40459226 |
| 57 | H | -2.94626957 | 1.22640649 | -2.43133607 |
| 58 | C | -1.47907010 | 2.31248955 | -3.55768780 |
| 59 | H | -2.08101353 | 2.33279130 | -4.46443558 |
| 60 | C | -1.24161357 | 1.85982318 | 2.18560589 |
| 61 | H | -7.24076445 | 2.68726759 | 1.23567699 |
| 62 | C | -5.98824606 | 4.23806796 | 0.37108480 |
| 63 | C | 3.28391651 | 2.89806591 | 0.33763847 |
| 64 | H | 3.64569735 | 1.87630644 | 0.40248248 |
| 65 | C | 2.46816445 | 5.56262166 | 0.14456401 |
| 66 | H | 2.13879184 | 6.59748521 | 0.06621162 |
| 67 | C | 4.20718995 | 3.93266136 | 0.54070120 |
| 68 | H | 5.24148461 | 3.68355226 | 0.76659902 |
| 69 | C | -0.19576928 | 2.87353925 | -3.53567672 |
| 70 | H | 0.22242898 | 3.33020349 | -4.43152124 |
| 71 | C | 3.80629357 | 5.26853117 | 0.44900044 |
| 72 | H | 4.52435751 | 6.07114386 | 0.60671171 |
| 73 | C | -0.01750208 | 2.54143115 | 1.82506220 |
| 74 | F | -4.85466811 | -3.50813125 | -2.37666160 |
| 75 | C | 3.12311232 | -5.41902654 | 0.55444984 |
| 76 | C | -4.85730512 | -3.25228745 | -1.03262932 |
| 77 | H | 2.42653633 | -2.64047809 | 2.38699909 |
| 78 | H | 2.84690544 | -2.91087688 | -1.73387951 |
| 79 | H | 3.31252775 | -5.37191738 | -1.60972914 |
| 80 | N | -4.00272132 | 2.27606111 | 0.37188709 |
| 81 | C | -5.24030820 | 1.99894413 | 0.84076058 |
| 82 | C | -3.74069304 | 3.51794511 | -0.09357131 |
| 83 | C | -6.25807931 | 2.95396432 | 0.85454228 |
| 84 | H | -5.40576588 | 0.98473240 | 1.19584199 |
| 85 | C | -4.70529485 | 4.52480337 | -0.10897936 |
| 86 | H | -2.73405781 | 3.68691582 | -0.46845899 |
| 87 | H | -1.55146927 | 0.15100951 | 3.59909697 |
| 88 | C | 1.06213618 | 2.17386207 | 2.82254451 |
| 89 | H | -0.07283610 | 3.55622327 | 1.45268595 |

| | | | | |
|---|---|---|---|---|
| 90 | H | -1.27613801 | 1.71870671 | 4.34927575 |
| 91 | C | 0.54566865 | 0.88541973 | 3.50135133 |
| 92 | H | 2.07207052 | 2.10872809 | 2.40847092 |
| 93 | H | 1.09369723 | 2.99113239 | 3.56144444 |
| 94 | C | -0.98492872 | 1.08334121 | 3.50033159 |
| 95 | H | -2.11467453 | 2.51628970 | 2.18261688 |
| 96 | H | 0.81450432 | 0.00306182 | 2.90129715 |
| 97 | H | 0.96363317 | 0.73311685 | 4.50060353 |

**I-2** (-13469.2)

| | | | | |
|---|---|---|---|---|
| 1 | Bi | 1.76839727 | -0.64374319 | -0.82295215 |
| 2 | S | 5.05260570 | -0.34026573 | -1.70401190 |
| 3 | N | -0.39375827 | -1.70161957 | -0.24714841 |
| 4 | H | 3.83376037 | -6.13972292 | 1.29528650 |
| 5 | O | 3.76437154 | -0.78891088 | -2.37302254 |
| 6 | C | -0.52528883 | -2.04751838 | -1.64736817 |
| 7 | C | 0.59764787 | -1.72152736 | -2.42181767 |
| 8 | O | 4.92895994 | -0.25525593 | -0.23359049 |
| 9 | O | 6.25177520 | -0.98488043 | -2.25904626 |
| 10 | C | -0.44441395 | -2.80140171 | 0.65824805 |
| 11 | F | 4.05070933 | 2.15337661 | -1.84389748 |
| 12 | C | -0.23128422 | -4.13604134 | 0.24218350 |
| 13 | H | -0.06302742 | -4.35273808 | -0.80824579 |
| 14 | F | 6.26080481 | 2.07043690 | -1.72729927 |
| 15 | C | -1.66848773 | -2.58377913 | -2.25113886 |
| 16 | H | -2.55039742 | -2.81955163 | -1.66264272 |
| 17 | C | 0.61497607 | -1.90681128 | -3.80380396 |
| 18 | H | 1.48151341 | -1.62756213 | -4.40046283 |
| 19 | C | -0.52383193 | -2.46110237 | -4.41027276 |
| 20 | H | -0.53884246 | -2.62820184 | -5.48606075 |
| 21 | H | -5.31672788 | 4.65113670 | 1.86133082 |
| 22 | H | -4.37739997 | 4.59083103 | -2.36302858 |
| 23 | N | 2.68420870 | -2.69981037 | -0.02897686 |
| 24 | C | -0.65606185 | -2.56275830 | 2.02887388 |
| 25 | H | -0.83953005 | -1.54995182 | 2.36594948 |
| 26 | C | -0.21256147 | -5.17646423 | 1.17404854 |
| 27 | H | -0.03211105 | -6.19362293 | 0.82941643 |
| 28 | C | -0.63184783 | -3.60508017 | 2.95936693 |
| 29 | H | -0.79524639 | -3.38507859 | 4.01330364 |
| 30 | C | -1.65256866 | -2.78775372 | -3.63697161 |
| 31 | H | -2.53623248 | -3.19524893 | -4.12460746 |
| 32 | C | -0.40582648 | -4.92245014 | 2.53932460 |
| 33 | H | -0.38304871 | -5.73647217 | 3.26112929 |
| 34 | C | 2.77963795 | -2.91438525 | 1.30425588 |
| 35 | C | 2.99214814 | -3.68917703 | -0.89993439 |
| 36 | C | 3.18782106 | -4.14260102 | 1.81360852 |
| 37 | C | 5.16061711 | 1.47364722 | -2.26234759 |
| 38 | C | 3.41779500 | -4.93665806 | -0.45315419 |
| 39 | F | 5.23273018 | 1.55990233 | -3.61890390 |
| 40 | Bi | -1.94152466 | 0.45058074 | 0.35011647 |
| 41 | S | -5.06645800 | 0.08939773 | -1.85228073 |
| 42 | H | -1.64396533 | 5.03881407 | -0.71326037 |
| 43 | H | -5.61740757 | 5.63806498 | -0.43668842 |

| | | | | |
|---|---|---|---|---|
| 44 | O | -3.63292032 | 0.09893386 | -2.24308911 |
| 45 | C | -0.24435999 | 0.73499852 | 3.28898951 |
| 46 | H | 0.11960133 | 3.32954543 | 2.64164938 |
| 47 | O | -5.31617237 | 0.09928747 | -0.39070491 |
| 48 | O | -5.93827826 | 0.98098954 | -2.65300927 |
| 49 | C | -0.51178477 | 1.59119035 | -1.05576301 |
| 50 | F | -5.00614265 | -2.62118322 | -1.63853611 |
| 51 | C | -0.95764687 | 1.56118013 | 2.20953005 |
| 52 | C | -0.46719383 | 1.20200779 | -2.41561045 |
| 53 | F | -6.98683229 | -1.79938543 | -2.17774764 |
| 54 | C | 0.89821212 | 3.10782879 | -2.97821590 |
| 55 | H | 0.25989672 | 1.60379949 | -4.41027680 |
| 56 | H | 1.32183715 | 4.46659600 | -1.36704175 |
| 57 | C | -0.06678603 | 4.80821953 | 0.74223316 |
| 58 | H | 1.47644077 | 3.68298154 | -3.69937423 |
| 59 | C | -0.98628260 | 5.55532071 | -0.02111087 |
| 60 | C | 0.68846581 | 6.87578628 | 1.78657502 |
| 61 | H | 1.51696235 | 4.93192881 | 2.21808781 |
| 62 | C | -0.22677769 | 7.61316200 | 1.02473500 |
| 63 | C | 0.09007021 | 2.82761868 | -0.68070662 |
| 64 | C | 0.13711170 | 2.58709288 | 1.83794193 |
| 65 | H | -1.84286510 | 2.06607398 | 2.61038231 |
| 66 | H | 1.91353774 | 0.25805507 | 3.46744950 |
| 67 | C | 0.23431104 | 1.93804631 | -3.37533213 |
| 68 | H | -1.01408070 | 0.31995578 | -2.72977487 |
| 69 | C | 0.77879377 | 5.48485672 | 1.64197712 |
| 70 | C | -1.06002728 | 6.94236916 | 0.11493402 |
| 71 | C | 0.82106238 | 3.54712312 | -1.65870430 |
| 72 | N | -0.04074573 | 3.38694085 | 0.60919469 |
| 73 | H | -1.78328135 | 7.50056053 | -0.47798814 |
| 74 | N | -3.28432207 | 2.59249920 | 0.12505019 |
| 75 | C | -3.44382335 | 3.11432838 | -1.11119072 |
| 76 | C | -5.63937062 | -1.65103202 | -2.37137838 |
| 77 | C | -3.95654429 | 3.13991033 | 1.15931992 |
| 78 | C | -4.27534327 | 4.20679071 | -1.35145961 |
| 79 | C | -4.79911584 | 4.23973853 | 0.99861331 |
| 80 | C | -4.96294765 | 4.78377644 | -0.27921840 |
| 81 | H | -2.89379260 | 2.63175501 | -1.91279266 |
| 82 | H | -3.81537048 | 2.68090468 | 2.13451258 |
| 83 | F | -5.37609205 | -1.88627104 | -3.69348277 |
| 84 | C | 3.51274715 | -5.17006754 | 0.92280772 |
| 85 | H | 2.51895763 | -2.08664851 | 1.95652968 |
| 86 | H | 2.88642777 | -3.45755460 | -1.95521806 |
| 87 | H | 3.66144136 | -5.70832233 | -1.17796713 |
| 88 | H | 3.24446164 | -4.28342140 | 2.88890533 |
| 89 | H | 1.34980135 | 7.38288053 | 2.48767967 |
| 90 | H | -0.29131773 | 8.69405698 | 1.13659296 |
| 91 | H | 1.89993591 | 1.63215357 | 0.93787310 |
| 92 | H | 2.23368222 | 2.41936373 | 2.46760481 |
| 93 | H | 1.12522026 | -0.35273535 | 2.01291752 |
| 94 | H | -0.76154872 | -0.18193415 | 3.58475995 |
| 95 | H | -0.15626140 | 1.34456001 | 4.20341236 |
| 96 | C | 1.16859140 | 0.50257633 | 2.70393261 |

| | | | | |
|---|---|---|---|---|
| 97 | C | 1.49118141 | 1.81360144 | 1.93763095 |

**Product** (-9140.7)

| | | | | |
|---|---|---|---|---|
| 1 | C | 5.01261555 | -2.80354192 | -1.02381031 |
| 2 | C | 5.04054166 | 3.50690088 | 1.02857577 |
| 3 | H | 6.00515290 | -3.24881351 | -1.07286041 |
| 4 | C | 3.44821784 | 1.94730065 | 2.01755410 |
| 5 | H | 5.51836335 | 4.48441978 | 1.00996732 |
| 6 | Bi | 0.32800562 | -0.72862488 | -0.75213740 |
| 7 | S | -2.88548835 | 1.28214145 | -0.23364267 |
| 8 | N | 3.17448726 | -0.32500613 | 1.08994732 |
| 9 | C | -3.29120690 | 2.08351886 | 1.44406426 |
| 10 | O | -1.52640801 | 1.80258431 | -0.53098241 |
| 11 | C | 4.07759836 | 3.19591556 | 1.99543654 |
| 12 | C | 3.44504221 | -1.28079972 | 0.04881639 |
| 13 | C | 2.44334962 | -1.65576324 | -0.86793473 |
| 14 | H | 3.79284062 | 3.93731144 | 2.74137841 |
| 15 | O | -3.96239832 | 1.78735829 | -1.11789656 |
| 16 | O | -2.93990731 | -0.17188572 | 0.06276553 |
| 17 | C | 3.77975727 | 0.94431521 | 1.06926577 |
| 18 | F | -3.28025403 | 3.44904245 | 1.34720326 |
| 19 | C | 4.73787017 | 1.28267948 | 0.07915526 |
| 20 | H | 4.98737403 | 0.56625217 | -0.69536988 |
| 21 | H | 2.68731768 | 1.77193486 | 2.77003268 |
| 22 | C | 5.35112287 | 2.53520639 | 0.06681875 |
| 23 | H | 6.07202491 | 2.75686854 | -0.71935494 |
| 24 | F | -4.53134095 | 1.70204166 | 1.88091089 |
| 25 | C | 4.71936796 | -1.87051003 | -0.02387486 |
| 26 | H | 5.47566693 | -1.58916590 | 0.70676346 |
| 27 | C | 2.74888877 | -2.60187740 | -1.86072883 |
| 28 | H | 1.98585517 | -2.89932361 | -2.58100597 |
| 29 | C | 4.02656752 | -3.17200580 | -1.95003704 |
| 30 | H | 4.24843816 | -3.89912568 | -2.73001743 |
| 31 | F | -2.38027204 | 1.72212710 | 2.39659744 |
| 32 | H | -2.40308758 | -2.31773778 | 0.19597300 |
| 33 | H | 0.82530085 | 2.55450255 | 0.15093410 |
| 34 | N | -0.50144307 | -3.11335127 | -0.06893198 |
| 35 | C | 0.28325386 | -4.21109751 | 0.00370575 |
| 36 | C | -0.19823226 | -5.44652082 | 0.43627625 |
| 37 | C | -1.54297575 | -5.55553517 | 0.80750678 |
| 38 | C | -2.36007526 | -4.42417357 | 0.71992746 |
| 39 | C | -1.80604261 | -3.22262347 | 0.27492360 |
| 40 | H | 1.32185325 | -4.08033999 | -0.28868677 |
| 41 | H | 0.47327676 | -6.30026366 | 0.48354766 |
| 42 | H | -3.41167642 | -4.46302591 | 0.99322394 |
| 43 | N | 1.58818952 | 1.37806763 | -1.38071825 |
| 44 | C | 2.38351275 | 1.31770378 | -2.47156511 |
| 45 | C | 3.09253749 | 2.42345689 | -2.93617232 |
| 46 | C | 2.98927535 | 3.63188658 | -2.23942438 |
| 47 | C | 2.17431124 | 3.69069977 | -1.10645173 |
| 48 | C | 1.48483478 | 2.54565128 | -0.71024531 |
| 49 | H | 2.45195151 | 0.35226646 | -2.96774503 |
| 50 | H | 3.71940400 | 2.32986088 | -3.81936499 |

| | | | | |
|---|---|---|---|---|
| 51 | H | 2.07533178 | 4.60417160 | -0.52673905 |
| 52 | H | 3.54065393 | 4.50927393 | -2.56992104 |
| 53 | H | -1.94571932 | -6.50408864 | 1.15613704 |
| 54 | C | 2.04120574 | -0.59843608 | 1.99736964 |
| 55 | C | 0.63865502 | -0.18072338 | 1.51128866 |
| 56 | H | 0.49693913 | 0.90314569 | 1.49798652 |
| 57 | H | 2.25133419 | -0.04393693 | 2.92000916 |
| 58 | H | 0.46950105 | -2.16933663 | 4.09440281 |
| 59 | H | -0.05964293 | -3.07668576 | 2.68110788 |
| 60 | C | -0.31525277 | -0.88274990 | 2.49486179 |
| 61 | C | 0.44216387 | -2.15790715 | 2.99908781 |
| 62 | C | 1.87892039 | -2.07850484 | 2.41432020 |
| 63 | H | 2.65180652 | -2.37644615 | 3.13122417 |
| 64 | H | 1.97550915 | -2.72852266 | 1.54131492 |
| 65 | H | -1.29257486 | -1.11249755 | 2.06144773 |
| 66 | H | -0.50716942 | -0.20830772 | 3.34088989 |

**1,3-cyclohexadiene** (-1822.6)

| | | | | |
|---|---|---|---|---|
| 1 | C | -1.43358253 | 1.24285198 | -0.16635965 |
| 2 | C | -0.10026747 | 3.20795166 | 0.44587755 |
| 3 | C | -1.37636596 | 2.48162759 | 0.36891822 |
| 4 | C | -0.18727087 | 0.59246492 | -0.73348492 |
| 5 | C | 1.09525973 | 1.07343174 | -0.01551245 |
| 6 | C | 1.07174930 | 2.56562644 | 0.25041953 |
| 7 | H | -2.38694636 | 0.72203693 | -0.26289824 |
| 8 | H | -0.11810907 | 4.27038499 | 0.69110939 |
| 9 | H | -2.28033390 | 2.97463537 | 0.72808462 |
| 10 | H | -0.12655756 | 0.83808328 | -1.80974060 |
| 11 | H | -0.26204459 | -0.50021527 | -0.67870563 |
| 12 | H | 1.98031846 | 0.79894147 | -0.60192555 |
| 13 | H | 1.19936218 | 0.54977499 | 0.95261117 |
| 14 | H | 2.01985621 | 3.09529134 | 0.35043906 |

**I-1** (-13668.1)

| | | | | |
|---|---|---|---|---|
| 1 | Bi | 2.05510249 | -0.65205222 | 0.05971338 |
| 2 | S | 5.34012995 | 0.24205508 | -0.44659485 |
| 3 | N | -0.40654509 | -1.50074716 | -0.22874313 |
| 4 | H | 2.67605956 | -7.09850148 | 0.59075415 |
| 5 | O | 4.08279183 | -0.09450976 | -1.23444685 |
| 6 | C | -0.08240144 | -1.64558918 | -1.63613210 |
| 7 | C | 1.19485612 | -1.18006182 | -1.96769454 |
| 8 | O | 5.11097753 | 0.26045431 | 1.01192330 |
| 9 | O | 6.53756622 | -0.45076351 | -0.94593068 |
| 10 | C | -0.91081903 | -2.65200765 | 0.46408415 |
| 11 | F | 4.52279766 | 2.81213549 | -0.59906515 |
| 12 | C | -0.68161110 | -3.95414421 | -0.02547366 |
| 13 | H | -0.16379682 | -4.09189887 | -0.96803752 |
| 14 | F | 6.69432055 | 2.55795205 | -0.26290033 |
| 15 | C | -0.95496661 | -2.10559422 | -2.63063603 |
| 16 | H | -1.95290011 | -2.45068061 | -2.37545175 |
| 17 | C | 1.62655443 | -1.13345149 | -3.29217729 |
| 18 | H | 2.61335042 | -0.75070825 | -3.54396371 |
| 19 | C | 0.75681924 | -1.58919606 | -4.29489008 |

| | | | | |
|---|---|---|---|---|
| 20 | H | 1.06870144 | -1.56087659 | -5.33770089 |
| 21 | H | 2.35981561 | -3.14578094 | 2.28627123 |
| 22 | H | 2.69577923 | -3.52327264 | -1.83318022 |
| 23 | N | 2.53034843 | -3.24102626 | 0.21626148 |
| 24 | C | -1.58443366 | -2.50571918 | 1.69120852 |
| 25 | H | -1.78450926 | -1.51599572 | 2.09260920 |
| 26 | C | -1.10380725 | -5.06984597 | 0.70106370 |
| 27 | H | -0.90575517 | -6.06473734 | 0.30570149 |
| 28 | C | -2.00454545 | -3.62576097 | 2.41339532 |
| 29 | H | -2.52674129 | -3.48333514 | 3.35796080 |
| 30 | C | -0.51804346 | -2.07670191 | -3.96056526 |
| 31 | H | -1.18546626 | -2.42127964 | -4.74872705 |
| 32 | C | -1.76634319 | -4.91720292 | 1.92586620 |
| 33 | H | -2.09476317 | -5.78869592 | 2.48908412 |
| 34 | C | 2.45893497 | -3.81743355 | 1.43500586 |
| 35 | C | 2.65958700 | -4.03068032 | -0.87182638 |
| 36 | C | 2.50444459 | -5.19938466 | 1.61374164 |
| 37 | C | 5.61132070 | 2.06280590 | -0.92513011 |
| 38 | C | 2.72391446 | -5.42117600 | -0.77678083 |
| 39 | F | 5.83035436 | 2.17441419 | -2.26504291 |
| 40 | Bi | -1.52208298 | 0.57545186 | 0.16552428 |
| 41 | S | -4.68330844 | -0.64556347 | -0.47991417 |
| 42 | N | 0.91990931 | 1.50320770 | -0.11172457 |
| 43 | H | 2.83044507 | -6.01988174 | -1.67792412 |
| 44 | O | -3.36917960 | -0.37658568 | -1.19044033 |
| 45 | C | 0.52475039 | 1.84599319 | -1.45869044 |
| 46 | C | -0.74371163 | 1.37775161 | -1.82089710 |
| 47 | O | -4.55755011 | -0.63013493 | 0.99062357 |
| 48 | O | -5.82176548 | 0.07531752 | -1.07272532 |
| 49 | C | 1.35117337 | 2.57433247 | 0.73349172 |
| 50 | F | -3.97469539 | -3.25506674 | -0.50353684 |
| 51 | C | 0.71096850 | 3.83116903 | 0.65730504 |
| 52 | H | -0.06162417 | 3.99083381 | -0.08993642 |
| 53 | F | -6.15186797 | -2.89496886 | -0.35923097 |
| 54 | C | 1.33733705 | 2.50399878 | -2.39309894 |
| 55 | H | 2.32378277 | 2.86649898 | -2.11203465 |
| 56 | C | -1.21614237 | 1.51380430 | -3.12627564 |
| 57 | H | -2.19576870 | 1.13030275 | -3.40545046 |
| 58 | C | -0.40486536 | 2.15776174 | -4.07157609 |
| 59 | H | -0.75104937 | 2.27398736 | -5.09756052 |
| 60 | H | -0.90291503 | 2.29873460 | 3.12178143 |
| 61 | H | 0.64037777 | -1.52984215 | 2.75604080 |
| 62 | H | 2.14996411 | -1.20616116 | 4.72255230 |
| 63 | C | 2.35599784 | 2.39345140 | 1.70053790 |
| 64 | H | 2.89331389 | 1.45164418 | 1.77451237 |
| 65 | C | 1.05620161 | 4.86079389 | 1.53592449 |
| 66 | H | 0.54446939 | 5.81941293 | 1.46290406 |
| 67 | C | 2.69748547 | 3.42670078 | 2.57781647 |
| 68 | H | 3.47635333 | 3.25814957 | 3.31927894 |
| 69 | C | 0.85636595 | 2.65732690 | -3.69942140 |
| 70 | H | 1.47680035 | 3.15710424 | -4.44200599 |
| 71 | C | 2.04901587 | 4.66569766 | 2.50737018 |
| 72 | H | 2.31576126 | 5.46780743 | 3.19290657 |

| | | | | |
|---|---|---|---|---|
| 73 | H | 0.46044655 | 2.75318232 | 5.15455570 |
| 74 | H | 2.43262282 | -5.61949705 | 2.61358820 |
| 75 | H | 2.04792517 | 0.97754034 | 5.97308185 |
| 76 | C | -4.99251080 | -2.46555866 | -0.93501726 |
| 77 | N | -0.17767986 | 0.37457525 | 2.83364250 |
| 78 | C | -0.22799803 | 1.53840491 | 3.51153007 |
| 79 | C | 0.66035442 | -0.58086982 | 3.29154013 |
| 80 | C | 0.54144856 | 1.79398858 | 4.64983636 |
| 81 | C | 1.48493983 | -0.40647507 | 4.40456032 |
| 82 | C | 1.42216052 | 0.80716395 | 5.09980023 |
| 83 | F | -5.10377464 | -2.60225538 | -2.28818796 |
| 84 | C | 2.63934776 | -6.01639401 | 0.48662605 |
| 85 | C | -2.36843715 | 5.23047189 | -0.55355027 |
| 86 | C | -2.95474137 | 3.92964737 | 1.54099033 |
| 87 | H | -2.05871803 | 5.91126783 | 1.47378275 |
| 88 | H | -3.73844224 | 5.88102122 | 0.95374365 |
| 89 | H | -1.81587827 | 6.06124733 | -0.99177816 |
| 90 | C | -2.72957274 | 4.17272074 | -1.30888807 |
| 91 | C | -3.66578522 | 2.96314617 | 0.61196962 |
| 92 | H | -3.48481407 | 4.00658638 | 2.49681384 |
| 93 | H | -1.95263031 | 3.53022116 | 1.77893998 |
| 94 | C | -3.53173307 | 3.08872821 | -0.73115012 |
| 95 | H | -2.45643032 | 4.11359548 | -2.36026502 |
| 96 | H | -4.27918326 | 2.17104104 | 1.03917356 |
| 97 | H | -4.02251918 | 2.38635511 | -1.40262774 |
| 98 | C | -2.78569315 | 5.32418679 | 0.89990403 |

**TS-1** (-13649.4)

| | | | | |
|---|---|---|---|---|
| 1 | Bi | 1.88150010 | -0.30025544 | 0.21752001 |
| 2 | S | 5.31262490 | -0.91577697 | 0.29726942 |
| 3 | N | -0.35259670 | -1.27965625 | -0.11519030 |
| 4 | H | 3.22132663 | -6.52546721 | 0.60099365 |
| 5 | O | 4.33059169 | -0.35207947 | -0.70791971 |
| 6 | C | -0.10783042 | -1.25325024 | -1.54928629 |
| 7 | C | 1.14148333 | -0.71325087 | -1.88104588 |
| 8 | O | 4.79427730 | -0.90392942 | 1.68124279 |
| 9 | O | 5.99183080 | -2.13893747 | -0.16147313 |
| 10 | C | -0.62122475 | -2.55097379 | 0.49130174 |
| 11 | F | 6.21320764 | 1.58550659 | 0.79335688 |
| 12 | C | -0.48465639 | -3.76996151 | -0.20099306 |
| 13 | H | -0.19037533 | -3.76749214 | -1.24506486 |
| 14 | F | 7.71757311 | -0.00667533 | 1.09580415 |
| 15 | C | -1.01835191 | -1.61765378 | -2.54760968 |
| 16 | H | -2.00069898 | -2.00732888 | -2.29290840 |
| 17 | C | 1.51328748 | -0.50914988 | -3.20819384 |
| 18 | H | 2.47753745 | -0.07374837 | -3.46409822 |
| 19 | C | 0.60854488 | -0.87926249 | -4.21635325 |
| 20 | H | 0.87241155 | -0.73059660 | -5.26223399 |
| 21 | H | 2.61437222 | -5.19008883 | 2.64644901 |
| 22 | H | -6.87116477 | 4.81781605 | 0.90620805 |
| 23 | N | 2.50611887 | -2.73261816 | 0.34116526 |
| 24 | C | -0.98924113 | -2.59111362 | 1.85115762 |
| 25 | H | -1.09051864 | -1.66580454 | 2.41166258 |

| | | | | |
|---|---|---|---|---|
| 26 | C | -0.70752665 | -4.98424754 | 0.45299452 |
| 27 | H | -0.58442955 | -5.91367191 | -0.10052480 |
| 28 | C | -1.21271361 | -3.80815094 | 2.50041427 |
| 29 | H | -1.49441695 | -3.80885037 | 3.55203807 |
| 30 | C | -0.64198593 | -1.42624117 | -3.88357433 |
| 31 | H | -1.33892466 | -1.69249691 | -4.67641515 |
| 32 | C | -1.07300161 | -5.01591715 | 1.80483774 |
| 33 | H | -1.24177916 | -5.96524702 | 2.30924093 |
| 34 | C | 2.44116516 | -3.35758302 | 1.53635974 |
| 35 | C | 2.82798298 | -3.43984818 | -0.76281461 |
| 36 | C | 2.68720713 | -4.72152227 | 1.66891328 |
| 37 | C | 6.68418335 | 0.40181582 | 0.30511974 |
| 38 | C | 3.09780456 | -4.80578445 | -0.70592924 |
| 39 | F | 7.16147422 | 0.61502408 | -0.95491442 |
| 40 | Bi | -2.20464012 | 0.34494703 | 0.48095227 |
| 41 | S | -5.28462637 | -1.51098770 | -0.70595801 |
| 42 | N | 0.82318126 | 2.13390072 | -0.15888465 |
| 43 | H | -4.59835680 | 5.44262915 | 0.01924907 |
| 44 | O | -4.24936295 | -0.75077355 | -1.46091084 |
| 45 | C | -0.11798164 | 2.21999896 | -1.25085911 |
| 46 | C | -1.40129858 | 1.62696851 | -1.21302086 |
| 47 | O | -5.11317365 | -1.48825624 | 0.76749734 |
| 48 | O | -6.67083116 | -1.32441641 | -1.19259694 |
| 49 | C | 1.85758633 | 3.15813466 | -0.25724598 |
| 50 | F | -3.62188836 | -3.64423565 | -0.77946376 |
| 51 | C | 1.50091553 | 4.52138745 | -0.19786858 |
| 52 | H | 0.45254395 | 4.80320036 | -0.14632179 |
| 53 | F | -5.75664106 | -4.17377074 | -0.54001084 |
| 54 | C | 0.28515345 | 2.84280233 | -2.46109209 |
| 55 | H | 1.28409353 | 3.25604658 | -2.54534651 |
| 56 | C | -2.25057655 | 1.72331050 | -2.32971949 |
| 57 | H | -3.23400650 | 1.26064566 | -2.29054066 |
| 58 | C | -1.85032863 | 2.36751861 | -3.50160951 |
| 59 | H | -2.52040762 | 2.41860654 | -4.35809014 |
| 60 | C | -1.06308597 | 1.69732407 | 2.10749840 |
| 61 | H | -7.27308310 | 2.43495744 | 1.61230410 |
| 62 | C | -6.07976977 | 4.07499961 | 0.83298660 |
| 63 | C | 3.21064424 | 2.82310954 | -0.38520421 |
| 64 | H | 3.53008285 | 1.78921053 | -0.47963953 |
| 65 | C | 2.47862121 | 5.51652987 | -0.22974452 |
| 66 | H | 2.18173512 | 6.56244939 | -0.17461520 |
| 67 | C | 4.19317080 | 3.82322294 | -0.42567114 |
| 68 | H | 5.23741438 | 3.53975735 | -0.52934107 |
| 69 | C | -0.56189259 | 2.91052346 | -3.56472689 |
| 70 | H | -0.20485270 | 3.38289180 | -4.47866244 |
| 71 | C | 3.83416782 | 5.17124148 | -0.33911708 |
| 72 | H | 4.59812370 | 5.94598486 | -0.36617851 |
| 73 | C | 0.09428884 | 2.50148829 | 1.64908005 |
| 74 | F | -4.99312535 | -3.51129037 | -2.51031348 |
| 75 | C | 3.02327954 | -5.45851834 | 0.52855960 |
| 76 | C | -4.89020362 | -3.31406737 | -1.15904731 |
| 77 | H | 2.18170127 | -2.74488014 | 2.39610784 |
| 78 | H | 2.86041390 | -2.88621708 | -1.69703555 |

| | | | | |
|---|---|---|---|---|
| 79 | H | 3.35715413 | -5.34154337 | -1.61524538 |
| 80 | N | -4.05155368 | 2.16753099 | 0.63708635 |
| 81 | C | -5.26886448 | 1.82641367 | 1.11588437 |
| 82 | C | -3.83174992 | 3.44514985 | 0.25483956 |
| 83 | C | -6.30730253 | 2.75270051 | 1.22702876 |
| 84 | H | -5.40154019 | 0.78410350 | 1.39605422 |
| 85 | C | -4.81826413 | 4.42704139 | 0.33905377 |
| 86 | H | -2.84197707 | 3.66468077 | -0.13910991 |
| 87 | C | 0.54325359 | 0.34585194 | 3.59690041 |
| 88 | C | 1.57191168 | 1.40633440 | 3.31085334 |
| 89 | C | -0.87020013 | 0.92848321 | 3.43411385 |
| 90 | H | 0.68957307 | -0.51435388 | 2.91727575 |
| 91 | H | 0.68093926 | -0.06039819 | 4.60559861 |
| 92 | H | 2.52585914 | 1.36287352 | 3.83532269 |
| 93 | C | 1.33848066 | 2.40630242 | 2.43814266 |
| 94 | H | -1.91791172 | 2.37365337 | 2.20375157 |
| 95 | H | -1.62636833 | 0.14184912 | 3.55279766 |
| 96 | H | -1.03331224 | 1.63077061 | 4.26546920 |
| 97 | H | -0.16687793 | 3.51055832 | 1.34759050 |
| 98 | H | 2.09633194 | 3.16764646 | 2.27942854 |

**I-2** (-13658.1)

| | | | | |
|---|---|---|---|---|
| 1 | Bi | 1.69086153 | -0.61253945 | -0.72910791 |
| 2 | S | 4.97329638 | -0.24629201 | -1.56480446 |
| 3 | N | -0.50128103 | -1.67697933 | -0.16253616 |
| 4 | H | 3.93063231 | -6.09783167 | 1.30285435 |
| 5 | O | 3.70868745 | -0.70308477 | -2.27075451 |
| 6 | C | -0.58707779 | -2.06962572 | -1.55715882 |
| 7 | C | 0.53845757 | -1.73496011 | -2.32228206 |
| 8 | O | 4.83050235 | -0.23463033 | -0.09332406 |
| 9 | O | 6.20008759 | -0.82427711 | -2.13320140 |
| 10 | C | -0.65306626 | -2.75831170 | 0.76189361 |
| 11 | F | 3.89100893 | 2.21742659 | -1.59934775 |
| 12 | C | -0.24050635 | -4.07068712 | 0.43322764 |
| 13 | H | 0.16236489 | -4.27225075 | -0.55454845 |
| 14 | F | 6.09974113 | 2.20044086 | -1.44199991 |
| 15 | C | -1.69710617 | -2.67850831 | -2.15475641 |
| 16 | H | -2.57653743 | -2.92931852 | -1.56863333 |
| 17 | C | 0.58292286 | -1.97903983 | -3.69552355 |
| 18 | H | 1.45136528 | -1.69802911 | -4.28919774 |
| 19 | C | -0.52385999 | -2.59781221 | -4.29868503 |
| 20 | H | -0.51412609 | -2.80892823 | -5.36693458 |
| 21 | H | -5.00629588 | 5.01829149 | 1.79205026 |
| 22 | H | -4.45026231 | 4.53391902 | -2.47234563 |
| 23 | N | 2.63101620 | -2.67919082 | 0.05555335 |
| 24 | C | -1.19708655 | -2.53948963 | 2.04123614 |
| 25 | H | -1.56750469 | -1.55512303 | 2.31068690 |
| 26 | C | -0.33384401 | -5.10396368 | 1.36631829 |
| 27 | H | 0.00626027 | -6.10138898 | 1.09218815 |
| 28 | C | -1.28771074 | -3.57606439 | 2.97662339 |
| 29 | H | -1.71171730 | -3.37268036 | 3.95872714 |
| 30 | C | -1.65166558 | -2.93816827 | -3.52992809 |
| 31 | H | -2.51049437 | -3.40044720 | -4.01294125 |

| | | | | |
|---|---|---|---|---|
| 32 | C | -0.84893269 | -4.86454141 | 2.64926646 |
| 33 | H | -0.91582389 | -5.67160680 | 3.37631026 |
| 34 | C | 2.75289503 | -2.91627436 | 1.38185239 |
| 35 | C | 2.96885270 | -3.64044217 | -0.83591664 |
| 36 | C | 3.21745935 | -4.13530309 | 1.86504050 |
| 37 | C | 5.03085944 | 1.59603989 | -2.02966888 |
| 38 | C | 3.44350921 | -4.88049844 | -0.41779022 |
| 39 | F | 5.12505242 | 1.75564098 | -3.37789316 |
| 40 | Bi | -1.97545099 | 0.46440429 | 0.32884622 |
| 41 | S | -5.00901577 | 0.05602080 | -2.03259022 |
| 42 | H | -1.68609947 | 4.97727473 | -0.69079632 |
| 43 | H | -5.45195026 | 5.82252744 | -0.55309717 |
| 44 | O | -3.55373927 | 0.03111196 | -2.33164956 |
| 45 | H | 0.63866734 | -1.06203348 | 2.64845184 |
| 46 | H | 0.23580089 | 3.20613599 | 2.66603292 |
| 47 | O | -5.34902066 | 0.19766239 | -0.59654878 |
| 48 | O | -5.82181127 | 0.87404187 | -2.96413175 |
| 49 | C | -0.52177335 | 1.59436025 | -1.06381135 |
| 50 | F | -5.00651080 | -2.62143481 | -1.55471569 |
| 51 | C | -0.92875271 | 1.50688688 | 2.20272959 |
| 52 | C | -0.45724838 | 1.22393425 | -2.42785533 |
| 53 | F | -6.92590529 | -1.83942654 | -2.32376158 |
| 54 | C | 0.86174870 | 3.17506939 | -2.95097908 |
| 55 | H | 0.27644732 | 1.67583495 | -4.40907841 |
| 56 | H | 1.24345830 | 4.51816965 | -1.31378590 |
| 57 | C | -0.17365144 | 4.75746593 | 0.83459779 |
| 58 | H | 1.42558344 | 3.77777622 | -3.66088514 |
| 59 | C | -1.09018892 | 5.49303780 | 0.05483971 |
| 60 | C | 0.42766198 | 6.81858452 | 1.98991194 |
| 61 | H | 1.33983031 | 4.90913134 | 2.38527925 |
| 62 | C | -0.48913931 | 7.54126463 | 1.21651632 |
| 63 | C | 0.05901419 | 2.83214807 | -0.66376261 |
| 64 | C | 0.19448863 | 2.49442642 | 1.83046934 |
| 65 | H | -1.79520054 | 2.09741912 | 2.52297415 |
| 66 | H | 1.27936072 | -0.40373161 | 4.13183582 |
| 67 | C | 0.23457670 | 1.99256574 | -3.36905454 |
| 68 | H | -0.98029279 | 0.33382050 | -2.75900566 |
| 69 | C | 0.59648020 | 5.44084663 | 1.79680011 |
| 70 | C | -1.24115932 | 6.86697095 | 0.24101783 |
| 71 | C | 0.76885399 | 3.59074877 | -1.62421279 |
| 72 | N | -0.07711809 | 3.34759723 | 0.64775802 |
| 73 | H | -1.96415090 | 7.41123461 | -0.36489473 |
| 74 | N | -3.26795743 | 2.68028451 | 0.08847736 |
| 75 | C | -3.50329516 | 3.10745296 | -1.17177524 |
| 76 | C | -5.56522837 | -1.72259226 | -2.42590302 |
| 77 | C | -3.81035378 | 3.36098296 | 1.11875180 |
| 78 | C | -4.28472774 | 4.22998484 | -1.44203966 |
| 79 | C | -4.59633561 | 4.49836723 | 0.92997816 |
| 80 | C | -4.84050981 | 4.94111372 | -0.37343631 |
| 81 | H | -3.05547941 | 2.52364684 | -1.97044828 |
| 82 | H | -3.60680373 | 2.98400040 | 2.11795010 |
| 83 | F | -5.20565518 | -2.08804667 | -3.69409521 |
| 84 | C | 3.56788037 | -5.13473428 | 0.95238088 |

| | | | | |
|---|---|---|---|---|
| 85 | H | 2.46743659 | -2.11362003 | 2.05342606 |
| 86 | H | 2.84419990 | -3.39423523 | -1.88586399 |
| 87 | H | 3.70567527 | -5.62941632 | -1.15977440 |
| 88 | H | 3.29705235 | -4.29106374 | 2.93706530 |
| 89 | H | 1.03120038 | 7.32777308 | 2.73999008 |
| 90 | H | -0.61530491 | 8.61185419 | 1.36782628 |
| 91 | H | 2.86529509 | 0.29286290 | 2.31554531 |
| 92 | C | 1.55592750 | 1.83581913 | 1.75599814 |
| 93 | H | -1.26829771 | -0.03417452 | 3.71637089 |
| 94 | H | -0.32035516 | 1.33821092 | 4.25408237 |
| 95 | H | 2.32484875 | 2.37050883 | 1.19769834 |
| 96 | C | 1.84738256 | 0.68361518 | 2.37750908 |
| 97 | C | -0.47577130 | 0.65523720 | 3.40233987 |
| 98 | C | 0.84064490 | -0.11220741 | 3.16673441 |

**Product** (-9327.3)

| | | | | |
|---|---|---|---|---|
| 1 | C | 5.08385732 | -2.75731367 | -0.95924734 |
| 2 | C | 4.87026591 | 3.62955821 | 0.96136365 |
| 3 | H | 6.09483303 | -3.15145261 | -1.05096925 |
| 4 | C | 3.41144075 | 2.00175106 | 2.04152077 |
| 5 | H | 5.28987972 | 4.63210489 | 0.90603883 |
| 6 | Bi | 0.34657496 | -0.84213939 | -0.56789373 |
| 7 | S | -2.92768038 | 1.17697496 | -0.29828422 |
| 8 | N | 3.22746011 | -0.29642502 | 1.16010053 |
| 9 | C | -2.81335584 | 2.40002431 | 1.15660071 |
| 10 | O | -1.78900398 | 1.59130779 | -1.15497241 |
| 11 | C | 3.96630610 | 3.28366824 | 1.97227640 |
| 12 | C | 3.50139533 | -1.26902427 | 0.13758200 |
| 13 | C | 2.46949591 | -1.73984472 | -0.69687473 |
| 14 | H | 3.66986372 | 4.02160462 | 2.71699481 |
| 15 | O | -4.27615592 | 1.44050577 | -0.85444970 |
| 16 | O | -2.76206972 | -0.14653970 | 0.35665969 |
| 17 | C | 3.76053119 | 1.00272148 | 1.09605577 |
| 18 | F | -3.01705936 | 3.68434458 | 0.73217416 |
| 19 | C | 4.66003682 | 1.37340620 | 0.06438216 |
| 20 | H | 4.92056200 | 0.65566617 | -0.70541518 |
| 21 | H | 2.69516057 | 1.79724723 | 2.82992793 |
| 22 | C | 5.20045150 | 2.65780719 | 0.00625826 |
| 23 | H | 5.87763851 | 2.90400606 | -0.81077359 |
| 24 | F | -3.74669736 | 2.11858389 | 2.11581087 |
| 25 | C | 4.80174593 | -1.78977290 | 0.00997039 |
| 26 | H | 5.58379667 | -1.43239831 | 0.67758944 |
| 27 | C | 2.76445646 | -2.72224127 | -1.65717456 |
| 28 | H | 1.97699112 | -3.09503296 | -2.31294235 |
| 29 | C | 4.06299560 | -3.23050343 | -1.79682104 |
| 30 | H | 4.27672452 | -3.98807392 | -2.54967849 |
| 31 | F | -1.57656436 | 2.34790367 | 1.74714730 |
| 32 | H | -2.41919557 | -2.51388050 | -0.35072745 |
| 33 | H | 0.64537354 | 2.50594183 | 0.13631073 |
| 34 | N | -0.48878132 | -3.23333470 | -0.07820715 |
| 35 | C | 0.29705119 | -4.29602005 | 0.19833465 |
| 36 | C | -0.23154301 | -5.56453290 | 0.43968672 |
| 37 | C | -1.61888601 | -5.74010531 | 0.39174337 |

| | | | | |
|---|---|---|---|---|
| 38 | C | -2.43130482 | -4.63974003 | 0.09605536 |
| 39 | C | -1.82953717 | -3.40237268 | -0.13444947 |
| 40 | H | 1.36565700 | -4.10599215 | 0.23372559 |
| 41 | H | 0.43598184 | -6.39296899 | 0.66385792 |
| 42 | H | -3.51355226 | -4.73072549 | 0.04816065 |
| 43 | N | 1.52242095 | 1.29057203 | -1.30245640 |
| 44 | C | 2.32599482 | 1.22044695 | -2.38549424 |
| 45 | C | 2.94394277 | 2.34681496 | -2.92636226 |
| 46 | C | 2.73752427 | 3.58742030 | -2.31532089 |
| 47 | C | 1.91076876 | 3.65805809 | -1.19046418 |
| 48 | C | 1.31375824 | 2.49033081 | -0.71933403 |
| 49 | H | 2.47819644 | 0.23126744 | -2.81145020 |
| 50 | H | 3.58258239 | 2.24398890 | -3.80004293 |
| 51 | H | 1.72937161 | 4.59962589 | -0.67931736 |
| 52 | H | 3.21717564 | 4.48261141 | -2.70468142 |
| 53 | H | -2.05899748 | -6.71697043 | 0.58031179 |
| 54 | C | 2.17946163 | -2.07948771 | 2.52888300 |
| 55 | C | -0.28721331 | -2.12761970 | 3.02924454 |
| 56 | H | 1.18554066 | -3.77982787 | 3.25274629 |
| 57 | C | 2.14516479 | -0.61957893 | 2.12199207 |
| 58 | H | 3.14982706 | -2.57348273 | 2.50509962 |
| 59 | C | -0.31717583 | -0.61863599 | 2.69467660 |
| 60 | H | -0.95930222 | -2.68350652 | 2.36172436 |
| 61 | H | -0.69423437 | -2.28547163 | 4.03934946 |
| 62 | C | 0.73902622 | -0.19015864 | 1.66772376 |
| 63 | H | 2.38502888 | -0.04761395 | 3.03068227 |
| 64 | H | -1.31613166 | -0.33335150 | 2.35239398 |
| 65 | H | -0.12348692 | -0.05112899 | 3.61995202 |
| 66 | H | 0.70964774 | 0.89825360 | 1.55359306 |
| 67 | C | 1.09043001 | -2.73533657 | 2.95074666 |

***neo*-hexene** (-2174.1)

| | | | | |
|---|---|---|---|---|
| 1 | C | -2.07303312 | 1.58991267 | -0.15302339 |
| 2 | C | -0.81655310 | 1.83434387 | 0.23994023 |
| 3 | H | -1.11445448 | -1.06763102 | 0.02989311 |
| 4 | H | -2.64207074 | 2.40517470 | -0.61214017 |
| 5 | H | -0.19196481 | 1.07194180 | 0.70335364 |
| 6 | H | -0.37133327 | 2.82006598 | 0.10850457 |
| 7 | C | -2.84265363 | 0.28264915 | -0.04613443 |
| 8 | C | -4.12318465 | 0.54329111 | 0.79325450 |
| 9 | H | -4.73839870 | -0.36513103 | 0.84099535 |
| 10 | H | -3.86415860 | 0.83976743 | 1.81768389 |
| 11 | H | -4.72904001 | 1.34324644 | 0.34788070 |
| 12 | C | -3.26018248 | -0.15069447 | -1.47839574 |
| 13 | H | -3.84485221 | 0.63646096 | -1.97213826 |
| 14 | H | -3.87622991 | -1.05887120 | -1.43673161 |
| 15 | H | -2.37689880 | -0.35975764 | -2.09514516 |
| 16 | C | -2.01760736 | -0.84247672 | 0.61104544 |
| 17 | H | -1.71136102 | -0.57097259 | 1.62920940 |
| 18 | H | -2.61769558 | -1.75922006 | 0.67194794 |

**I-1** (-14017.8)

| | | | | |
|---|---|---|---|---|
| 1 | Bi | 2.18381162 | -0.79434680 | 0.13073860 |

| | | | | |
|---|---|---|---|---|
| 2 | S | 5.53665427 | -0.09272872 | -0.16330470 |
| 3 | N | -0.28709832 | -1.50342183 | -0.30342917 |
| 4 | H | 2.41367564 | -7.25604039 | 0.66396495 |
| 5 | O | 4.31256642 | -0.34147923 | -1.03225142 |
| 6 | C | 0.09916529 | -1.64913863 | -1.69435880 |
| 7 | C | 1.41730071 | -1.25562594 | -1.95095247 |
| 8 | O | 5.21799493 | -0.07070629 | 1.27830168 |
| 9 | O | 6.71651098 | -0.85760825 | -0.59446278 |
| 10 | C | -0.87065319 | -2.63504273 | 0.35795828 |
| 11 | F | 4.89289443 | 2.52455384 | -0.34736355 |
| 12 | C | -0.72156158 | -3.93948428 | -0.15450415 |
| 13 | H | -0.20196904 | -4.09390512 | -1.09359488 |
| 14 | F | 7.01635941 | 2.13354733 | 0.13305892 |
| 15 | C | -0.74722660 | -2.03921659 | -2.73967440 |
| 16 | H | -1.77741131 | -2.32348157 | -2.54240881 |
| 17 | C | 1.92033106 | -1.21788317 | -3.25021415 |
| 18 | H | 2.93916250 | -0.88962350 | -3.44487889 |
| 19 | C | 1.07852291 | -1.60672879 | -4.30386192 |
| 20 | H | 1.44590634 | -1.58275664 | -5.32850079 |
| 21 | H | 2.15968938 | -3.29673623 | 2.35392681 |
| 22 | H | 2.79297955 | -3.68106953 | -1.73040666 |
| 23 | N | 2.48624988 | -3.39638752 | 0.30249423 |
| 24 | C | -1.54523847 | -2.46693188 | 1.58205703 |
| 25 | H | -1.68684679 | -1.47274756 | 1.99759797 |
| 26 | C | -1.22430513 | -5.03802914 | 0.54684504 |
| 27 | H | -1.08713653 | -6.03647900 | 0.13503793 |
| 28 | C | -2.04543828 | -3.56991252 | 2.27867132 |
| 29 | H | -2.56596083 | -3.41122408 | 3.22159321 |
| 30 | C | -0.23960482 | -2.01886032 | -4.04463227 |
| 31 | H | -0.88466430 | -2.30887108 | -4.87233238 |
| 32 | C | -1.88754787 | -4.86507281 | 1.76827442 |
| 33 | H | -2.27725947 | -5.72358564 | 2.31193170 |
| 34 | C | 2.29280344 | -3.97045139 | 1.50905603 |
| 35 | C | 2.65887872 | -4.18844169 | -0.77778013 |
| 36 | C | 2.25692510 | -5.35319322 | 1.68213794 |
| 37 | C | 5.95230519 | 1.71005127 | -0.60526159 |
| 38 | C | 2.64717712 | -5.58047317 | -0.68622218 |
| 39 | F | 6.26895269 | 1.81380287 | -1.92618385 |
| 40 | Bi | -1.32019421 | 0.62146152 | 0.07005794 |
| 41 | S | -4.53390328 | -0.34082873 | -0.65389580 |
| 42 | N | 1.16299347 | 1.43231611 | -0.06388416 |
| 43 | H | 2.79199559 | -6.18172053 | -1.58026176 |
| 44 | O | -3.22738035 | -0.07509499 | -1.38050507 |
| 45 | C | 0.87207719 | 1.80167331 | -1.42953735 |
| 46 | C | -0.39489581 | 1.39969591 | -1.87250932 |
| 47 | O | -4.37576537 | -0.36118789 | 0.81432393 |
| 48 | O | -5.67800326 | 0.40108032 | -1.20480100 |
| 49 | C | 1.63242643 | 2.46989461 | 0.80435462 |
| 50 | F | -3.85326883 | -2.95775282 | -0.76205886 |
| 51 | C | 1.09204265 | 3.77136848 | 0.70583536 |
| 52 | H | 0.37055335 | 3.98978979 | -0.07631897 |
| 53 | F | -6.02070349 | -2.58057342 | -0.53658360 |
| 54 | C | 1.77877008 | 2.41439455 | -2.30658352 |

| | | | | |
|---|---|---|---|---|
| 55 | H | 2.76149053 | 2.72635552 | -1.95933975 |
| 56 | C | -0.76062179 | 1.54123177 | -3.21211251 |
| 57 | H | -1.72885168 | 1.19321471 | -3.56472996 |
| 58 | C | 0.14403002 | 2.13877095 | -4.10089725 |
| 59 | H | -0.12231540 | 2.25840620 | -5.14997619 |
| 60 | H | -0.80716151 | 2.39702796 | 3.02845713 |
| 61 | H | 0.61223078 | -1.49155387 | 2.80386932 |
| 62 | H | 2.03108794 | -1.18614286 | 4.83731591 |
| 63 | C | 2.58206053 | 2.21728247 | 1.80944029 |
| 64 | H | 3.04483031 | 1.23875849 | 1.90377779 |
| 65 | C | 1.47723004 | 4.77420852 | 1.59852488 |
| 66 | H | 1.04090171 | 5.76799389 | 1.50717211 |
| 67 | C | 2.96566530 | 3.22344399 | 2.70086704 |
| 68 | H | 3.70064358 | 2.99798967 | 3.47136718 |
| 69 | C | 1.39713349 | 2.58394793 | -3.64322895 |
| 70 | H | 2.09102476 | 3.04840018 | -4.34243795 |
| 71 | C | 2.41436448 | 4.50647516 | 2.60770380 |
| 72 | H | 2.71229503 | 5.28738650 | 3.30475942 |
| 73 | H | 0.46892618 | 2.83865196 | 5.11774698 |
| 74 | H | 2.08732994 | -5.77146100 | 2.67080074 |
| 75 | H | 1.94484672 | 1.02186434 | 6.04684145 |
| 76 | C | -4.87664687 | -2.14692250 | -1.14025934 |
| 77 | N | -0.14504353 | 0.43967075 | 2.81118872 |
| 78 | C | -0.18384090 | 1.61932594 | 3.46446418 |
| 79 | C | 0.63636219 | -0.53459225 | 3.32506146 |
| 80 | C | 0.53810261 | 1.86735632 | 4.63493975 |
| 81 | C | 1.41149579 | -0.36924680 | 4.47455984 |
| 82 | C | 1.35747074 | 0.85765829 | 5.14611694 |
| 83 | F | -5.03426947 | -2.25176288 | -2.49205694 |
| 84 | C | 2.43807254 | -6.17323554 | 0.56376647 |
| 85 | C | -2.75888545 | 3.25607356 | 1.06039421 |
| 86 | H | -1.81792795 | 3.80393037 | 1.06169570 |
| 87 | H | -2.99007207 | 2.66744579 | 1.94675604 |
| 88 | C | -3.65210011 | 3.35713006 | 0.06077947 |
| 89 | H | -3.29156013 | 2.47315177 | -2.52354640 |
| 90 | H | -4.58787625 | 2.79923927 | 0.15052774 |
| 91 | C | -3.54646944 | 4.20186321 | -1.19440077 |
| 92 | C | -4.56069981 | 5.37257580 | -1.04509036 |
| 93 | H | -4.57392478 | 5.97250648 | -1.96450227 |
| 94 | H | -5.57569929 | 4.99486102 | -0.86791077 |
| 95 | H | -4.28365072 | 6.02402060 | -0.20703170 |
| 96 | C | -2.12970557 | 4.76963993 | -1.41564678 |
| 97 | H | -1.81198821 | 5.40102099 | -0.57667554 |
| 98 | H | -2.11423966 | 5.38758045 | -2.32144753 |
| 99 | H | -1.39970363 | 3.96374087 | -1.54949148 |
| 100 | C | -3.96024007 | 3.33081526 | -2.40917756 |
| 101 | H | -4.97874117 | 2.94543262 | -2.28424863 |
| 102 | H | -3.92099213 | 3.92788084 | -3.32906394 |

**TS-1** (-13992.8)

| | | | | |
|---|---|---|---|---|
| 1 | Bi | 2.08524320 | -0.34793766 | -0.08864523 |
| 2 | S | 5.45461716 | -1.05310321 | -0.68367306 |
| 3 | N | -0.18656035 | -1.29180019 | -0.05900115 |

| | | | | |
|---|---|---|---|---|
| 4 | H | 3.29635872 | -6.61251028 | 0.17687766 |
| 5 | O | 4.32994311 | -0.42612696 | -1.47636234 |
| 6 | C | -0.13731882 | -1.34528949 | -1.51196458 |
| 7 | C | 1.02370625 | -0.76453753 | -2.03780412 |
| 8 | O | 5.26405592 | -0.94104397 | 0.77837411 |
| 9 | O | 5.90333064 | -2.35460225 | -1.20685388 |
| 10 | C | -0.41836082 | -2.51094606 | 0.65843276 |
| 11 | F | 6.66740152 | 1.36434220 | -0.56726654 |
| 12 | C | -0.39333583 | -3.77807412 | 0.04449385 |
| 13 | H | -0.21728306 | -3.85791410 | -1.02269611 |
| 14 | F | 8.04890047 | -0.35980428 | -0.47869918 |
| 15 | C | -1.14895071 | -1.80480380 | -2.36356534 |
| 16 | H | -2.06154727 | -2.23796025 | -1.96773214 |
| 17 | C | 1.20169076 | -0.59937844 | -3.40855199 |
| 18 | H | 2.10190378 | -0.13692547 | -3.80868134 |
| 19 | C | 0.18658578 | -1.04973557 | -4.26841040 |
| 20 | H | 0.29479798 | -0.93027882 | -5.34519723 |
| 21 | H | 3.12557529 | -5.21412798 | 2.26286245 |
| 22 | H | 3.26195711 | 1.49587103 | 1.83922286 |
| 23 | N | 2.66423023 | -2.80223253 | -0.04803083 |
| 24 | C | -0.63822612 | -2.44286565 | 2.04862560 |
| 25 | H | -0.65691973 | -1.47528287 | 2.54317114 |
| 26 | C | -0.57323274 | -4.93653174 | 0.80472367 |
| 27 | H | -0.53841093 | -5.90553704 | 0.30947995 |
| 28 | C | -0.81665354 | -3.60423856 | 2.80474585 |
| 29 | H | -0.98260155 | -3.52329867 | 3.87777647 |
| 30 | C | -0.97011576 | -1.64964256 | -3.74473932 |
| 31 | H | -1.75254278 | -1.98764818 | -4.42214199 |
| 32 | C | -0.78414397 | -4.86200242 | 2.18741556 |
| 33 | H | -0.92036143 | -5.76794423 | 2.77486213 |
| 34 | C | 2.80342884 | -3.39994205 | 1.15487828 |
| 35 | C | 2.75131611 | -3.54349843 | -1.17268040 |
| 36 | C | 3.02806451 | -4.76876354 | 1.27656954 |
| 37 | C | 6.90873816 | 0.11404999 | -1.05844776 |
| 38 | C | 2.98332679 | -4.91701085 | -1.13011257 |
| 39 | F | 7.11683369 | 0.21307036 | -2.40301922 |
| 40 | Bi | -2.02013034 | 0.40067767 | 0.52904916 |
| 41 | S | -5.15077951 | -1.46991242 | -0.49755234 |
| 42 | N | 1.09605971 | 2.10101486 | -0.08845129 |
| 43 | H | 0.53593990 | 3.46005475 | 1.64015107 |
| 44 | O | -4.10897841 | -0.74813404 | -1.28154294 |
| 45 | C | 0.13123527 | 2.24046734 | -1.16561829 |
| 46 | C | -1.13313326 | 1.59994349 | -1.17961550 |
| 47 | O | -4.92860237 | -1.46462832 | 0.96914034 |
| 48 | O | -6.54191148 | -1.22057137 | -0.93813772 |
| 49 | C | 2.13992253 | 3.11606108 | -0.19180910 |
| 50 | F | -3.60581636 | -3.69230969 | -0.58896053 |
| 51 | C | 1.82631419 | 4.47246542 | 0.02839544 |
| 52 | H | 0.79643307 | 4.76647809 | 0.21432709 |
| 53 | F | -5.76730387 | -4.10381217 | -0.36159484 |
| 54 | C | 0.51548886 | 2.94022594 | -2.34184832 |
| 55 | H | 1.48785257 | 3.41547841 | -2.39293760 |
| 56 | C | -1.94949781 | 1.67411157 | -2.32258525 |

| | | | | |
|---|---|---|---|---|
| 57 | H | -2.91000215 | 1.16299490 | -2.32158569 |
| 58 | C | -1.55239913 | 2.36514297 | -3.46762268 |
| 59 | H | -2.19462418 | 2.39336993 | -4.34605373 |
| 60 | C | -1.00136793 | 2.01032442 | 1.98965353 |
| 61 | H | -1.21220456 | 1.55245148 | 2.95619278 |
| 62 | H | -1.69474763 | 2.82867026 | 1.79430523 |
| 63 | C | 3.45612204 | 2.78458970 | -0.53925290 |
| 64 | H | 3.73325508 | 1.76269544 | -0.78560109 |
| 65 | C | 2.81597502 | 5.45633597 | -0.02984811 |
| 66 | H | 2.54977149 | 6.49588700 | 0.15337299 |
| 67 | C | 4.44883545 | 3.77072086 | -0.60464649 |
| 68 | H | 5.46267688 | 3.48894228 | -0.87576504 |
| 69 | C | -0.30436476 | 2.99666951 | -3.46712216 |
| 70 | H | 0.04785369 | 3.52712420 | -4.35067121 |
| 71 | C | 4.13857229 | 5.10782658 | -0.33564875 |
| 72 | H | 4.91253859 | 5.87155514 | -0.38231644 |
| 73 | C | 0.39322477 | 2.40569761 | 1.83755075 |
| 74 | F | -4.96196434 | -3.46517711 | -2.32278900 |
| 75 | C | 3.12097489 | -5.54097461 | 0.11384610 |
| 76 | C | -4.85391335 | -3.28759365 | -0.96999371 |
| 77 | H | 2.72681080 | -2.76026377 | 2.03112603 |
| 78 | H | 2.62837233 | -3.01049239 | -2.11143407 |
| 79 | H | 3.05150937 | -5.48057648 | -2.05691767 |
| 80 | N | -3.91346706 | 2.16919147 | 0.50831759 |
| 81 | C | -5.10690181 | 1.85089508 | 1.05727392 |
| 82 | C | -3.74382365 | 3.40020001 | -0.02343040 |
| 83 | C | -6.17294160 | 2.75135672 | 1.08613490 |
| 84 | H | -5.19937190 | 0.84681438 | 1.46328321 |
| 85 | C | -4.76116441 | 4.35382775 | -0.02957613 |
| 86 | H | -2.76914721 | 3.60560981 | -0.45947793 |
| 87 | C | -5.99947179 | 4.02379792 | 0.53293306 |
| 88 | H | -7.11775139 | 2.45185530 | 1.53299587 |
| 89 | H | -4.58125526 | 5.33133100 | -0.47021720 |
| 90 | H | -6.81341701 | 4.74549303 | 0.53929335 |
| 91 | C | 1.37896155 | 1.91679409 | 2.92385040 |
| 92 | C | 1.03338769 | 2.82621964 | 4.15603794 |
| 93 | H | 1.69686761 | 2.55527309 | 4.98590487 |
| 94 | H | 1.19456989 | 3.88427318 | 3.91850376 |
| 95 | H | -0.00260995 | 2.69547929 | 4.48326284 |
| 96 | C | 1.17069596 | 0.44771586 | 3.35062662 |
| 97 | H | 0.16902509 | 0.26600610 | 3.75109558 |
| 98 | H | 1.89100979 | 0.18707958 | 4.13367166 |
| 99 | H | 1.32840458 | -0.25517262 | 2.52485063 |
| 100 | C | 2.87675976 | 2.15214009 | 2.62300117 |
| 101 | H | 3.07508087 | 3.18552769 | 2.32660392 |
| 102 | H | 3.45173680 | 1.93940961 | 3.53166175 |

**I-2** (-14010.6)

| | | | | |
|---|---|---|---|---|
| 1 | Bi | 1.84690908 | -0.95051780 | -1.32858602 |
| 2 | S | 4.91636828 | -1.28910135 | -2.79436328 |
| 3 | N | -0.31885122 | -1.70467029 | -0.34393630 |
| 4 | H | 3.50515899 | -6.56563946 | 0.81292846 |
| 5 | O | 3.45927827 | -1.45558041 | -3.19180535 |

| | | | | |
|---|---|---|---|---|
| 6 | C | -0.69690715 | -2.14751430 | -1.67357548 |
| 7 | C | 0.30750587 | -1.95527550 | -2.63407554 |
| 8 | O | 5.08059032 | -0.99858225 | -1.35439497 |
| 9 | O | 5.80354093 | -2.29357851 | -3.39887920 |
| 10 | C | -0.27003988 | -2.71125935 | 0.66788112 |
| 11 | F | 4.55509702 | 1.34710283 | -3.20783972 |
| 12 | C | -0.46552680 | -4.08490351 | 0.40063917 |
| 13 | H | -0.69587538 | -4.41281505 | -0.60778779 |
| 14 | F | 6.65780910 | 0.67809095 | -3.39269523 |
| 15 | C | -1.94546319 | -2.65212760 | -2.05811340 |
| 16 | H | -2.74164867 | -2.78785450 | -1.33062011 |
| 17 | C | 0.10649113 | -2.25144562 | -3.98179112 |
| 18 | H | 0.89049940 | -2.08512517 | -4.71850695 |
| 19 | C | -1.13750684 | -2.77905465 | -4.36395195 |
| 20 | H | -1.32362975 | -3.03176812 | -5.40652917 |
| 21 | H | -4.57011233 | 4.95382298 | 2.13504623 |
| 22 | H | -4.40845207 | 4.74602236 | -2.18357033 |
| 23 | N | 2.60849751 | -3.07566087 | -0.57104341 |
| 24 | C | 0.06462726 | -2.34205030 | 1.98828052 |
| 25 | H | 0.25817456 | -1.30001960 | 2.20988589 |
| 26 | C | -0.34324957 | -5.03488519 | 1.41989904 |
| 27 | H | -0.48898799 | -6.08732429 | 1.18036957 |
| 28 | C | 0.18091845 | -3.29320028 | 3.00365417 |
| 29 | H | 0.44442293 | -2.97044538 | 4.00971468 |
| 30 | C | -2.14837372 | -2.97044847 | -3.40755784 |
| 31 | H | -3.11357623 | -3.36043016 | -3.72098658 |
| 32 | C | -0.02536091 | -4.65178206 | 2.72916318 |
| 33 | H | 0.07103985 | -5.39679150 | 3.51633948 |
| 34 | C | 2.98505283 | -3.21401157 | 0.72186102 |
| 35 | C | 2.55049123 | -4.15794427 | -1.38264518 |
| 36 | C | 3.31038022 | -4.45817068 | 1.25211302 |
| 37 | C | 5.36485016 | 0.34382222 | -3.65743938 |
| 38 | C | 2.87831606 | -5.42680364 | -0.91572409 |
| 39 | F | 5.20933953 | 0.23484796 | -5.00586921 |
| 40 | Bi | -1.93840502 | 0.42707046 | 0.13676493 |
| 41 | S | -4.93472799 | 0.36337716 | -2.39572161 |
| 42 | H | -0.94387103 | 4.68407283 | -1.21282624 |
| 43 | H | -5.16209276 | 5.94762856 | -0.10148064 |
| 44 | O | -3.47195968 | 0.26001108 | -2.63763526 |
| 45 | H | 2.37776497 | 3.80756693 | 1.55266315 |
| 46 | H | -0.34316294 | 3.05584042 | 2.86610163 |
| 47 | O | -5.33124880 | 0.36158244 | -0.96720006 |
| 48 | O | -5.63757360 | 1.34998235 | -3.25093026 |
| 49 | C | -0.36238549 | 1.61430711 | -1.03726202 |
| 50 | F | -5.18000350 | -2.34258444 | -2.25073731 |
| 51 | C | -0.95488136 | 1.18415286 | 2.10769270 |
| 52 | C | -0.05156038 | 1.26496296 | -2.36867441 |
| 53 | F | -6.98525549 | -1.30320329 | -2.98814521 |
| 54 | C | 1.69666258 | 2.93941272 | -2.43979756 |
| 55 | H | 1.20845647 | 1.62354130 | -4.09519839 |
| 56 | H | 1.87893274 | 4.16388018 | -0.67666194 |
| 57 | C | -0.49294337 | 4.56551942 | 0.90675109 |
| 58 | H | 2.50771510 | 3.44282410 | -2.96144555 |

| | | | | |
|---|---|---|---|---|
| 59 | C | -0.94611112 | 5.21652705 | -0.26849162 |
| 60 | C | -1.04426995 | 6.61013634 | 2.14028736 |
| 61 | H | -0.18514182 | 4.87915782 | 3.04529126 |
| 62 | C | -1.49326497 | 7.23733308 | 0.97263788 |
| 63 | C | 0.31576915 | 2.69579820 | -0.43423932 |
| 64 | C | 0.01921002 | 2.38941317 | 2.08481786 |
| 65 | H | -1.82885604 | 1.41895380 | 2.72384563 |
| 66 | H | -0.50424625 | 0.30701752 | 2.56728539 |
| 67 | C | 0.98124886 | 1.91036590 | -3.07028741 |
| 68 | H | -0.63863877 | 0.50220359 | -2.87285479 |
| 69 | C | -0.54290397 | 5.30415245 | 2.11457260 |
| 70 | C | -1.43138189 | 6.52257729 | -0.23173258 |
| 71 | C | 1.35363093 | 3.33573200 | -1.14669434 |
| 72 | N | -0.05537085 | 3.22215840 | 0.84707072 |
| 73 | H | -1.78803821 | 6.97621168 | -1.15549583 |
| 74 | N | -3.15677632 | 2.63718932 | 0.13195898 |
| 75 | C | -3.45700020 | 3.17444189 | -1.07143706 |
| 76 | C | -5.61565495 | -1.30024640 | -3.02400379 |
| 77 | C | -3.55975052 | 3.27532843 | 1.25155296 |
| 78 | C | -4.18511157 | 4.35533847 | -1.19436306 |
| 79 | C | -4.27747688 | 4.46922273 | 1.20747845 |
| 80 | C | -4.60156448 | 5.01800412 | -0.03655381 |
| 81 | H | -3.10498538 | 2.63224419 | -1.94361158 |
| 82 | H | -3.29589613 | 2.81599834 | 2.19942894 |
| 83 | F | -5.22991012 | -1.54072783 | -4.31450007 |
| 84 | C | 3.25658502 | -5.58193329 | 0.42231808 |
| 85 | H | 3.01894717 | -2.31154751 | 1.32486535 |
| 86 | H | 2.23097372 | -3.98076924 | -2.40477021 |
| 87 | H | 2.82615305 | -6.27483635 | -1.59264573 |
| 88 | H | 3.59676370 | -4.53608135 | 2.29666229 |
| 89 | H | -1.06983554 | 7.14126572 | 3.09117992 |
| 90 | H | -1.88176634 | 8.25356855 | 0.99915175 |
| 91 | C | 1.49408226 | 2.04096874 | 2.51978520 |
| 92 | C | 1.46071763 | 1.47112137 | 3.96025468 |
| 93 | H | 2.48492875 | 1.34251861 | 4.33154250 |
| 94 | H | 0.93644970 | 2.15478523 | 4.64087154 |
| 95 | H | 0.96474697 | 0.49582956 | 4.01555844 |
| 96 | C | 2.16617507 | 1.01226113 | 1.58617239 |
| 97 | H | 1.54067007 | 0.12012848 | 1.46852309 |
| 98 | H | 3.12922589 | 0.69179398 | 2.00276164 |
| 99 | H | 2.35725539 | 1.43907202 | 0.59824525 |
| 100 | C | 2.35203098 | 3.32750397 | 2.53523647 |
| 101 | H | 1.96492009 | 4.05406008 | 3.25907989 |
| 102 | H | 3.38228332 | 3.08414527 | 2.82311770 |

**Product** (-9677.6)

| | | | | |
|---|---|---|---|---|
| 1 | C | 4.75912710 | -3.02948489 | -1.03867712 |
| 2 | C | 5.45683165 | 2.95732818 | 1.15623577 |
| 3 | H | 5.73063886 | -3.51988604 | -0.99781617 |
| 4 | C | 3.78492495 | 1.49907662 | 2.16972721 |
| 5 | H | 6.10120514 | 3.83303247 | 1.20508105 |
| 6 | Bi | 0.21203874 | -0.70425405 | -1.17594902 |
| 7 | S | -3.02528437 | 1.32425028 | -0.66544473 |

| | | | | |
|---|---|---|---|---|
| 8 | N | 2.88304131 | -0.43322306 | 0.89305450 |
| 9 | C | -3.59246551 | 1.17753405 | 1.14524290 |
| 10 | O | -1.63141278 | 1.82228397 | -0.54462148 |
| 11 | C | 4.61776034 | 2.62250200 | 2.22498869 |
| 12 | C | 3.17507928 | -1.42631186 | -0.10355379 |
| 13 | C | 2.25448265 | -1.74915370 | -1.11905142 |
| 14 | H | 4.61029511 | 3.23392483 | 3.12686243 |
| 15 | O | -3.99223496 | 2.28501379 | -1.24653130 |
| 16 | O | -3.14284156 | -0.07361106 | -1.15610171 |
| 17 | C | 3.74975205 | 0.67093529 | 1.01987239 |
| 18 | F | -3.47432723 | 2.37415060 | 1.79714898 |
| 19 | C | 4.61063995 | 1.02110054 | -0.05275589 |
| 20 | H | 4.60187688 | 0.42681195 | -0.95955084 |
| 21 | H | 3.17428599 | 1.27751653 | 3.03813686 |
| 22 | C | 5.44258637 | 2.13622166 | 0.01909239 |
| 23 | H | 6.07071106 | 2.37880896 | -0.83720475 |
| 24 | F | -4.90105498 | 0.78402425 | 1.21782874 |
| 25 | C | 4.42598025 | -2.07589376 | -0.07446014 |
| 26 | H | 5.14204146 | -1.81491632 | 0.70162694 |
| 27 | C | 2.60270347 | -2.70694580 | -2.08575301 |
| 28 | H | 1.90535544 | -2.94543514 | -2.88966284 |
| 29 | C | 3.84602068 | -3.35249173 | -2.05330159 |
| 30 | H | 4.10203066 | -4.09012670 | -2.81254961 |
| 31 | F | -2.84038249 | 0.25391496 | 1.82169092 |
| 32 | H | -2.58522579 | -2.00317362 | -0.03910082 |
| 33 | H | 0.85519207 | 2.29657336 | 0.33911113 |
| 34 | N | -0.88158230 | -3.01975495 | -0.65846007 |
| 35 | C | -0.27247557 | -4.21567197 | -0.81034592 |
| 36 | C | -0.87712991 | -5.41501186 | -0.43156652 |
| 37 | C | -2.15664906 | -5.37854455 | 0.13169272 |
| 38 | C | -2.79043470 | -4.14089617 | 0.28621735 |
| 39 | C | -2.12477994 | -2.98513812 | -0.12440020 |
| 40 | H | 0.73023969 | -4.19729713 | -1.22819626 |
| 41 | H | -0.34565209 | -6.35328142 | -0.57070550 |
| 42 | H | -3.78620181 | -4.06525630 | 0.71637277 |
| 43 | N | 1.54520471 | 1.42942155 | -1.41637085 |
| 44 | C | 2.31774144 | 1.56305143 | -2.51670071 |
| 45 | C | 3.05483252 | 2.71846451 | -2.77116666 |
| 46 | C | 3.00300581 | 3.76777481 | -1.84825889 |
| 47 | C | 2.21104956 | 3.62437924 | -0.70571301 |
| 48 | C | 1.49260016 | 2.44385897 | -0.52627623 |
| 49 | H | 2.34077695 | 0.71581436 | -3.19918496 |
| 50 | H | 3.66226310 | 2.78303511 | -3.67035231 |
| 51 | H | 2.15150108 | 4.40868955 | 0.04398979 |
| 52 | H | 3.57596582 | 4.67748349 | -2.01255449 |
| 53 | H | -2.65053214 | -6.29598577 | 0.44463818 |
| 54 | C | 1.71022672 | -0.60028114 | 1.80547653 |
| 55 | C | 0.33104332 | -0.43855992 | 1.11741004 |
| 56 | H | 3.98874988 | -2.11847642 | 2.60733748 |
| 57 | H | -0.40480916 | -1.14806032 | 1.49875785 |
| 58 | H | 1.78329905 | 0.25387862 | 2.47654134 |
| 59 | H | -0.07738007 | 0.56101121 | 1.27871097 |
| 60 | C | 1.81089577 | -1.85176261 | 2.75818022 |

| | | | | |
|---|---|---|---|---|
| 61 | C | 0.80224367 | -1.64223604 | 3.91696000 |
| 62 | H | 0.88760620 | -2.46434012 | 4.63874933 |
| 63 | H | 1.00245750 | -0.70230147 | 4.44888870 |
| 64 | H | -0.23475007 | -1.61648867 | 3.56352452 |
| 65 | C | 1.48618200 | -3.18865933 | 2.05658783 |
| 66 | H | 0.47121008 | -3.18871768 | 1.64915496 |
| 67 | H | 1.55067118 | -4.00915713 | 2.78270698 |
| 68 | H | 2.17938819 | -3.40406369 | 1.23956938 |
| 69 | C | 3.22928323 | -1.92378824 | 3.36920464 |
| 70 | H | 3.49232893 | -0.98937046 | 3.88022842 |
| 71 | H | 3.27426621 | -2.73482637 | 4.10716214 |

**Non-favored mechanisms involving mononuclear species: concerted vs. coordination/insertion**
**TS concerted, ethylene insertion** (-8190.9)

| | | | | |
|---|---|---|---|---|
| 1 | C | 3.57503529 | -1.51175955 | 0.13830289 |
| 2 | C | 2.12192700 | 4.96416907 | 1.67974160 |
| 3 | H | 4.64538374 | -1.71524505 | 0.14041939 |
| 4 | C | 0.72084055 | 3.11147737 | 2.39534000 |
| 5 | H | 2.37576663 | 6.02225200 | 1.70917629 |
| 6 | Bi | -1.20056035 | -0.10322026 | 0.54237074 |
| 7 | S | -1.67792166 | -1.14616515 | -3.32120972 |
| 8 | N | 1.00013882 | 0.87548711 | 1.51024714 |
| 9 | C | -2.19291031 | -2.97745595 | -3.36729532 |
| 10 | O | -2.25680677 | -0.67145521 | -2.03015808 |
| 11 | C | 1.05669156 | 4.46498247 | 2.44461934 |
| 12 | C | 1.70144662 | -0.13175676 | 0.81148861 |
| 13 | C | 0.83494617 | -1.02357829 | 0.13954452 |
| 14 | H | 0.47143830 | 5.13760039 | 3.07061494 |
| 15 | O | -2.34071607 | -0.57873300 | -4.51939626 |
| 16 | O | -0.20293456 | -1.21525560 | -3.36811698 |
| 17 | C | 1.45291920 | 2.20353399 | 1.58986572 |
| 18 | F | -3.54984017 | -3.11235343 | -3.26299294 |
| 19 | C | 2.51732597 | 2.72213782 | 0.81528710 |
| 20 | H | 3.06231786 | 2.06860459 | 0.14244749 |
| 21 | H | -0.13745580 | 2.75119396 | 2.95800690 |
| 22 | C | 2.83798658 | 4.08205099 | 0.86025282 |
| 23 | H | 3.64826270 | 4.45590321 | 0.23529505 |
| 24 | F | -1.79966889 | -3.55937916 | -4.54225847 |
| 25 | C | 3.08976116 | -0.39419953 | 0.82545824 |
| 26 | H | 3.77339270 | 0.25504929 | 1.36781629 |
| 27 | C | 1.32436206 | -2.13635785 | -0.54762376 |
| 28 | H | 0.65111924 | -2.79739371 | -1.08850397 |
| 29 | C | 2.70572434 | -2.37830893 | -0.55228001 |
| 30 | H | 3.10820586 | -3.23603216 | -1.08971479 |
| 31 | F | -1.61813648 | -3.67681766 | -2.34158458 |
| 32 | H | -4.01229862 | -1.72948774 | -0.13789970 |
| 33 | H | -1.37434318 | 3.31623321 | 0.13525669 |
| 34 | N | -2.20138237 | -2.45777139 | 0.57382936 |
| 35 | C | -1.48997961 | -3.53844365 | 0.96309506 |
| 36 | C | -2.03334469 | -4.82269066 | 0.96891099 |
| 37 | C | -3.36168939 | -4.99522887 | 0.56454741 |
| 38 | C | -4.09844628 | -3.87541211 | 0.16636591 |
| 39 | C | -3.48305077 | -2.62373764 | 0.18113171 |

| | | | | |
|---|---|---|---|---|
| 40 | H | -0.46124187 | -3.35604217 | 1.26374389 |
| 41 | H | -1.42244674 | -5.66518019 | 1.28339924 |
| 42 | H | -5.13189359 | -3.96382196 | -0.15897469 |
| 43 | N | -0.34214059 | 1.91720663 | -1.00250052 |
| 44 | C | 0.55837491 | 1.70370310 | -1.98448125 |
| 45 | C | 1.17281579 | 2.75332402 | -2.67091465 |
| 46 | C | 0.85030026 | 4.06953592 | -2.32392513 |
| 47 | C | -0.08044632 | 4.29048641 | -1.30418933 |
| 48 | C | -0.65337952 | 3.18702231 | -0.66991794 |
| 49 | H | 0.77369810 | 0.66433158 | -2.21903031 |
| 50 | H | 1.89253957 | 2.53647258 | -3.45707074 |
| 51 | H | -0.35346165 | 5.29597880 | -0.99416976 |
| 52 | H | 1.31991525 | 4.90756964 | -2.83492737 |
| 53 | H | -3.81286081 | -5.98501947 | 0.55736903 |
| 54 | C | 0.38359346 | -0.01766771 | 3.31261594 |
| 55 | C | -0.87032249 | -0.61797955 | 3.09667220 |
| 56 | H | 1.27643834 | -0.63641233 | 3.34146890 |
| 57 | H | -0.92816799 | -1.70293626 | 3.04338694 |
| 58 | H | 0.45928913 | 0.92049396 | 3.85317086 |
| 59 | H | -1.76201174 | -0.11712952 | 3.47371369 |

**TS-concerted, 1-hexene insertion** (-9656.0)

| | | | | |
|---|---|---|---|---|
| 1 | C | 3.60785798 | -1.37880721 | -0.17725932 |
| 2 | C | 2.17394743 | 5.05027697 | 1.56492982 |
| 3 | H | 4.67950051 | -1.55046810 | -0.27248963 |
| 4 | C | 0.81710088 | 3.17665071 | 2.31059376 |
| 5 | H | 2.41879808 | 6.11041971 | 1.59594566 |
| 6 | Bi | -1.16014315 | -0.07425866 | 0.58358427 |
| 7 | S | -1.76724653 | -1.18858060 | -3.29228157 |
| 8 | N | 1.09616394 | 0.94600292 | 1.40764234 |
| 9 | C | -2.20686124 | -3.04045051 | -3.27489474 |
| 10 | O | -2.29992434 | -0.70912740 | -1.98564009 |
| 11 | C | 1.13988102 | 4.53330284 | 2.35987660 |
| 12 | C | 1.76422204 | -0.04349609 | 0.65376476 |
| 13 | C | 0.86621929 | -0.96408102 | 0.06669410 |
| 14 | H | 0.56853060 | 5.19393150 | 3.01099352 |
| 15 | O | -2.51151319 | -0.67935522 | -4.46995954 |
| 16 | O | -0.29484518 | -1.19778962 | -3.41544784 |
| 17 | C | 1.53332978 | 2.28057181 | 1.47709800 |
| 18 | F | -3.55329080 | -3.22793299 | -3.12042764 |
| 19 | C | 2.56038471 | 2.81954952 | 0.66682345 |
| 20 | H | 3.08670279 | 2.18059000 | -0.03351440 |
| 21 | H | -0.02132147 | 2.80689932 | 2.89616354 |
| 22 | C | 2.86469746 | 4.18342158 | 0.70881920 |
| 23 | H | 3.64675131 | 4.57085328 | 0.05675268 |
| 24 | F | -1.83049745 | -3.63878317 | -4.44760108 |
| 25 | C | 3.15444676 | -0.27043613 | 0.54478717 |
| 26 | H | 3.86714679 | 0.39659419 | 1.02295897 |
| 27 | C | 1.32326242 | -2.07219375 | -0.64817398 |
| 28 | H | 0.62355853 | -2.75593481 | -1.12248167 |
| 29 | C | 2.70500568 | -2.27679567 | -0.77645546 |
| 30 | H | 3.08077522 | -3.12803258 | -1.34299064 |
| 31 | F | -1.57138444 | -3.69135931 | -2.25233862 |

| | | | | |
|---|---|---|---|---|
| 32 | H | -3.93767122 | -1.80640136 | -0.02567089 |
| 33 | H | -1.35278817 | 3.41029492 | 0.11639391 |
| 34 | N | -2.09294778 | -2.44418725 | 0.68457751 |
| 35 | C | -1.33589312 | -3.48430844 | 1.09744699 |
| 36 | C | -1.83011087 | -4.78675434 | 1.15301081 |
| 37 | C | -3.15631297 | -5.02145056 | 0.77294889 |
| 38 | C | -3.94023371 | -3.94330868 | 0.35093239 |
| 39 | C | -3.37238422 | -2.66956321 | 0.31582533 |
| 40 | H | -0.30982391 | -3.25325099 | 1.37271805 |
| 41 | H | -1.18353898 | -5.59568483 | 1.48382557 |
| 42 | H | -4.97397815 | -4.08069731 | 0.04411840 |
| 43 | N | -0.38408064 | 1.99220832 | -1.05189726 |
| 44 | C | 0.47785498 | 1.76554506 | -2.06492738 |
| 45 | C | 1.08612059 | 2.80413473 | -2.77466244 |
| 46 | C | 0.79815139 | 4.12594441 | -2.41826391 |
| 47 | C | -0.09440948 | 4.36257620 | -1.36825922 |
| 48 | C | -0.66073995 | 3.26778399 | -0.71200221 |
| 49 | H | 0.66781502 | 0.72255563 | -2.30727313 |
| 50 | H | 1.77378685 | 2.57495905 | -3.58585641 |
| 51 | H | -0.34068264 | 5.37274555 | -1.05067971 |
| 52 | H | 1.26270511 | 4.95583283 | -2.94709755 |
| 53 | H | -3.57022121 | -6.02688403 | 0.80503691 |
| 54 | C | 0.57809205 | 0.07919736 | 3.25102166 |
| 55 | C | -0.72768971 | -0.46206199 | 3.04557488 |
| 56 | H | 2.95440462 | -2.05375026 | 7.41286905 |
| 57 | H | -0.82853076 | -1.54381956 | 3.13730548 |
| 58 | H | 0.65411424 | 1.05287716 | 3.72795851 |
| 59 | H | -1.56635404 | 0.09692979 | 3.46407663 |
| 60 | C | 1.74303675 | -0.83618829 | 3.50472931 |
| 61 | H | 1.71475176 | -1.67879190 | 2.80044363 |
| 62 | H | 2.68810298 | -0.30529763 | 3.33755057 |
| 63 | C | 1.72654680 | -1.38562069 | 4.95398842 |
| 64 | H | 1.73231577 | -0.54421510 | 5.66340922 |
| 65 | H | 0.78990771 | -1.93504924 | 5.12540101 |
| 66 | C | 2.92537907 | -2.30690148 | 5.24042506 |
| 67 | H | 2.92030486 | -3.13850894 | 4.51941402 |
| 68 | H | 3.85852313 | -1.75016925 | 5.06654694 |
| 69 | C | 2.91681849 | -2.86576835 | 6.67348071 |
| 70 | H | 2.00502058 | -3.44900133 | 6.86309612 |
| 71 | H | 3.77892883 | -3.52198704 | 6.85018256 |

**TS-concerted, cyclopentene insertion** (-9116.2)

| | | | | |
|---|---|---|---|---|
| 1 | C | 3.69649378 | -1.44166363 | 0.07770764 |
| 2 | C | 2.01610125 | 4.97390592 | 1.64203509 |
| 3 | H | 4.77370616 | -1.60006959 | 0.04315795 |
| 4 | C | 0.66069795 | 3.08263558 | 2.34363284 |
| 5 | H | 2.22640997 | 6.04178889 | 1.65521365 |
| 6 | Bi | -1.11462879 | -0.21897695 | 0.65373243 |
| 7 | S | -1.68597007 | -1.11528175 | -3.28086676 |
| 8 | N | 1.06875652 | 0.83558275 | 1.53724780 |
| 9 | C | -2.38910748 | -2.87050970 | -3.49658473 |
| 10 | O | -2.26256703 | -0.68961695 | -1.97565042 |
| 11 | C | 0.94151863 | 4.44930372 | 2.37509423 |

| | | | | |
|---|---|---|---|---|
| 12 | C | 1.78997109 | -0.14856959 | 0.82967371 |
| 13 | C | 0.93786029 | -1.07134477 | 0.17815903 |
| 14 | H | 0.30201380 | 5.11139875 | 2.95768363 |
| 15 | O | -2.23271572 | -0.38786484 | -4.45237682 |
| 16 | O | -0.22531462 | -1.34248462 | -3.28836647 |
| 17 | C | 1.46105791 | 2.18506206 | 1.59216689 |
| 18 | F | -3.75566272 | -2.87120631 | -3.41605303 |
| 19 | C | 2.53148608 | 2.73034343 | 0.84524953 |
| 20 | H | 3.12696833 | 2.08772742 | 0.20624230 |
| 21 | H | -0.21365917 | 2.70699993 | 2.87011753 |
| 22 | C | 2.79475311 | 4.10318823 | 0.86900161 |
| 23 | H | 3.61110240 | 4.49514989 | 0.26333232 |
| 24 | F | -2.04033679 | -3.38810126 | -4.71633598 |
| 25 | C | 3.18715727 | -0.35578511 | 0.79786334 |
| 26 | H | 3.86156205 | 0.31306915 | 1.32686949 |
| 27 | C | 1.45223544 | -2.14458042 | -0.55060719 |
| 28 | H | 0.79266705 | -2.81613159 | -1.09513096 |
| 29 | C | 2.84198498 | -2.32804941 | -0.60360473 |
| 30 | H | 3.26152615 | -3.15471150 | -1.17555794 |
| 31 | F | -1.91026789 | -3.71487414 | -2.53324984 |
| 32 | H | -3.98114633 | -1.68020627 | 0.17193353 |
| 33 | H | -1.38405892 | 3.25584816 | 0.08051732 |
| 34 | N | -2.12936613 | -2.55418011 | 0.52880498 |
| 35 | C | -1.42721015 | -3.70023828 | 0.66773887 |
| 36 | C | -2.02779778 | -4.95551790 | 0.57687243 |
| 37 | C | -3.40390230 | -5.02971486 | 0.33800013 |
| 38 | C | -4.13033059 | -3.84319639 | 0.19364030 |
| 39 | C | -3.45770655 | -2.62580451 | 0.29159583 |
| 40 | H | -0.36241736 | -3.59441805 | 0.85220604 |
| 41 | H | -1.42333283 | -5.85151114 | 0.69254673 |
| 42 | H | -5.20057579 | -3.85373431 | 0.00362663 |
| 43 | N | -0.31115720 | 1.83893889 | -0.99369241 |
| 44 | C | 0.60423256 | 1.61283676 | -1.95826547 |
| 45 | C | 1.20873068 | 2.64977436 | -2.67320151 |
| 46 | C | 0.85780008 | 3.97064539 | -2.37438465 |
| 47 | C | -0.09005091 | 4.20693881 | -1.37397860 |
| 48 | C | -0.64964368 | 3.11288902 | -0.71029833 |
| 49 | H | 0.84362297 | 0.57073665 | -2.15546761 |
| 50 | H | 1.94161028 | 2.42044686 | -3.44370999 |
| 51 | H | -0.38642759 | 5.21681659 | -1.10134861 |
| 52 | H | 1.31794289 | 4.80016575 | -2.90764301 |
| 53 | H | -3.90063355 | -5.99452437 | 0.26339564 |
| 54 | H | -1.67552741 | -0.05410595 | 3.50207733 |
| 55 | H | 1.22333866 | -3.15804431 | 4.10725038 |
| 56 | H | 1.09087492 | -2.63207847 | 2.41873057 |
| 57 | H | -0.93522995 | -2.16967990 | 4.67697261 |
| 58 | H | -1.27632442 | -2.76830387 | 3.05516133 |
| 59 | C | 0.48568188 | 0.04211402 | 3.39136859 |
| 60 | C | 1.52721717 | -0.98764329 | 3.77554338 |
| 61 | H | 0.56888071 | 1.04205296 | 3.80175636 |
| 62 | C | -0.78173013 | -0.59916715 | 3.18948110 |
| 63 | C | 0.86689884 | -2.35408761 | 3.45462375 |
| 64 | H | 2.49818982 | -0.84059103 | 3.29012766 |

| | | | | |
|---|---|---|---|---|
| 65 | H | 1.69409364 | -0.88479121 | 4.86058987 |
| 66 | C | -0.64731866 | -2.07220619 | 3.61722460 |

**Intermediate stepwise, ethylene insertion** (-6611.9)

| | | | | |
|---|---|---|---|---|
| 1 | C | 5.01992616 | -1.53921237 | -0.25961965 |
| 2 | C | 1.31147914 | 3.56308087 | 2.34173693 |
| 3 | H | 6.02006082 | -1.61608146 | 0.16581516 |
| 4 | C | 0.34769135 | 1.39598567 | 1.80246002 |
| 5 | H | 1.22801198 | 4.54303083 | 2.80843453 |
| 6 | Bi | 0.27790998 | -0.71128496 | -1.40661188 |
| 7 | S | -3.16028049 | 0.13785394 | -0.37343137 |
| 8 | N | 1.55627381 | -0.24647231 | 0.49320410 |
| 9 | C | -3.67233359 | 1.63754458 | 0.68033738 |
| 10 | O | -2.06872917 | -0.48611687 | 0.42722401 |
| 11 | C | 0.24446244 | 2.65272354 | 2.40079343 |
| 12 | C | 2.73669462 | -0.77911561 | -0.09593973 |
| 13 | C | 2.47693590 | -1.35820942 | -1.35850598 |
| 14 | H | -0.67721308 | 2.92591157 | 2.91232040 |
| 15 | O | -2.69349203 | 0.76221041 | -1.63909579 |
| 16 | O | -4.40263873 | -0.65837958 | -0.46874129 |
| 17 | C | 1.52585987 | 1.01655512 | 1.11769105 |
| 18 | F | -2.64494703 | 2.53383414 | 0.78232604 |
| 19 | C | 2.59100568 | 1.94442724 | 1.05054293 |
| 20 | H | 3.49396152 | 1.69144302 | 0.50329779 |
| 21 | H | -0.48868774 | 0.70173479 | 1.82460112 |
| 22 | C | 2.48129235 | 3.19670599 | 1.66233015 |
| 23 | H | 3.31200975 | 3.89798674 | 1.58993607 |
| 24 | F | -4.73903162 | 2.27996560 | 0.11692392 |
| 25 | C | 4.02507216 | -0.86214152 | 0.46332081 |
| 26 | H | 4.24848417 | -0.42868266 | 1.43589116 |
| 27 | C | 3.46533123 | -2.03439098 | -2.06797534 |
| 28 | H | 3.25729011 | -2.48012627 | -3.04058387 |
| 29 | C | 4.75618226 | -2.12309759 | -1.51070698 |
| 30 | H | 5.54936508 | -2.64138929 | -2.04770005 |
| 31 | F | -4.02505333 | 1.25057613 | 1.94238936 |
| 32 | N | 0.97533770 | 1.55406647 | -1.94505277 |
| 33 | H | -0.82298133 | 2.26157692 | -1.16931745 |
| 34 | C | 2.22093450 | 1.84421524 | -2.39045233 |
| 35 | C | 2.69320875 | 3.15373830 | -2.42762009 |
| 36 | C | 1.86743860 | 4.18800868 | -1.97389165 |
| 37 | C | 0.58724089 | 3.87961560 | -1.50527943 |
| 38 | C | 0.16757796 | 2.55180134 | -1.50814922 |
| 39 | H | 2.83173422 | 1.00146500 | -2.70078921 |
| 40 | H | 3.69643606 | 3.35021006 | -2.79638312 |
| 41 | H | -0.08282853 | 4.65125357 | -1.13648111 |
| 42 | H | 2.21932730 | 5.21696693 | -1.98085590 |
| 43 | C | 0.03193214 | -3.14283973 | 0.57999697 |
| 44 | C | -0.72392779 | -3.46592822 | -0.48357327 |
| 45 | H | -0.37050653 | -2.55496372 | 1.40174453 |
| 46 | H | 1.07298809 | -3.45024998 | 0.65522741 |
| 47 | H | -1.76741769 | -3.16242684 | -0.54616854 |
| 48 | H | -0.32811931 | -4.06438304 | -1.30346677 |

**TS stepwise** (-6603.0)

| | | | | |
|---|---|---|---|---|
| 1 | C | 5.15868328 | -1.53593879 | -0.58118921 |
| 2 | C | 1.53637536 | 3.37006085 | 2.30755362 |
| 3 | H | 6.16788758 | -1.60010504 | -0.17667336 |
| 4 | C | 0.54623574 | 1.22286774 | 1.74392556 |
| 5 | H | 1.47649641 | 4.33295117 | 2.81167963 |
| 6 | Bi | 0.32230801 | -0.97402887 | -1.70792383 |
| 7 | S | -3.08452766 | 0.33564499 | -0.63499340 |
| 8 | N | 1.68104703 | -0.35087765 | 0.26800165 |
| 9 | C | -3.66884523 | 1.80847716 | 0.41979374 |
| 10 | O | -1.79628396 | -0.02941786 | 0.02309891 |
| 11 | C | 0.47889272 | 2.45346913 | 2.39897975 |
| 12 | C | 2.86166250 | -0.83235822 | -0.37453751 |
| 13 | C | 2.57794307 | -1.39716318 | -1.63317148 |
| 14 | H | -0.41429602 | 2.70523926 | 2.96855661 |
| 15 | O | -2.93007093 | 0.91150499 | -1.99079260 |
| 16 | O | -4.16943035 | -0.65713477 | -0.47140534 |
| 17 | C | 1.68556834 | 0.87255062 | 0.98578708 |
| 18 | F | -2.75588595 | 2.82833131 | 0.38590202 |
| 19 | C | 2.74067750 | 1.80646556 | 0.88782763 |
| 20 | H | 3.60508724 | 1.58327181 | 0.27160741 |
| 21 | H | -0.30676808 | 0.55377894 | 1.77208438 |
| 22 | C | 2.66128826 | 3.03909563 | 1.54158360 |
| 23 | H | 3.48068868 | 3.74902347 | 1.43874904 |
| 24 | F | -4.86025760 | 2.29426863 | -0.04050792 |
| 25 | C | 4.15617859 | -0.91650922 | 0.17405185 |
| 26 | H | 4.37769867 | -0.51214838 | 1.15924482 |
| 27 | C | 3.58341506 | -2.02449339 | -2.37593914 |
| 28 | H | 3.36984439 | -2.46340960 | -3.35040531 |
| 29 | C | 4.88293564 | -2.08362224 | -1.84883724 |
| 30 | H | 5.68044369 | -2.56340794 | -2.41496414 |
| 31 | F | -3.83652784 | 1.43795767 | 1.72448171 |
| 32 | N | 0.94030700 | 1.50933058 | -1.97865016 |
| 33 | H | -0.73047972 | 2.12709312 | -0.91730048 |
| 34 | C | 2.10602593 | 1.85931187 | -2.56485454 |
| 35 | C | 2.55548010 | 3.17943459 | -2.58537826 |
| 36 | C | 1.77974597 | 4.16733059 | -1.96894006 |
| 37 | C | 0.57736326 | 3.80012057 | -1.35851986 |
| 38 | C | 0.18786850 | 2.46129656 | -1.38539719 |
| 39 | H | 2.68619008 | 1.05293166 | -3.00715351 |
| 40 | H | 3.49828614 | 3.41994287 | -3.07047433 |
| 41 | H | -0.05052261 | 4.53229965 | -0.85769674 |
| 42 | H | 2.11184598 | 5.20316119 | -1.95830895 |
| 43 | C | 0.84405916 | -2.02474777 | 1.17524454 |
| 44 | C | 0.05594174 | -2.66856670 | 0.19223278 |
| 45 | H | 0.36082975 | -1.45372693 | 1.96188529 |
| 46 | H | 1.82579291 | -2.41594466 | 1.42741986 |
| 47 | H | -1.02621282 | -2.59938876 | 0.28942569 |
| 48 | H | 0.43980710 | -3.58171876 | -0.26285394 |

### Single-crystal X-ray diffraction analyses

Square planar coordination polyhedra around the bismuth atoms (with varying extents of distortion) have been assigned in all cases. Importantly, the Bi–O interatomic distances vary, suggesting varying strengths of interactions in the solid state. In some cases, additional even weaker Bi–O interactions may be discussed based on distance criteria.

### Compound 2-Ph

Compound **2-Ph** crystallised from a difluorobenzene solution which was layered with *n*-pentane at –30 °C, over the period of 2 weeks in the monoclinic space group *C 2/c* with Z = 8. The unit cell parameters are a = 13.628(4) Å b = 16.811(5) Å c = 23.972(9) Å, α = 90° β = 94.89(2)° γ = 90°. Bi1-C1 bond length is 2.246(5) Å, C1– C2 bond length is 1.520(8) Å and C2–N1 bond length is 1.458(6) Å.

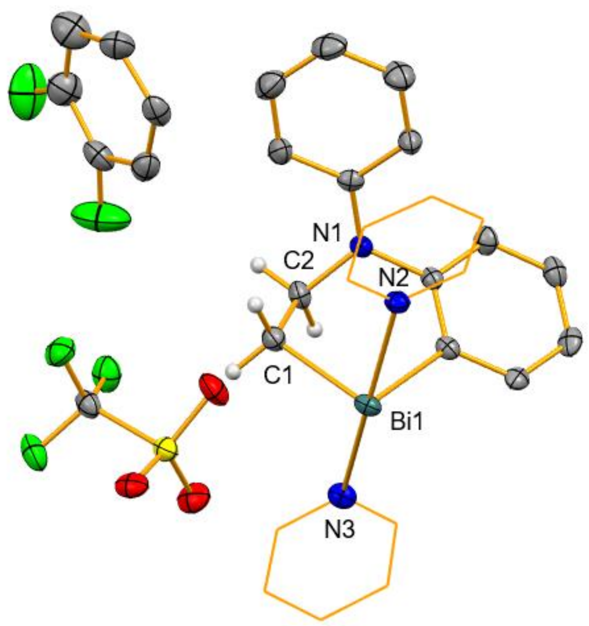


**Figure S53**: Molecular structure of compound **2-Ph** in the solid state. Displacement ellipsoids are drawn at the 50% probability level. Hydrogen atoms are omitted for clarity. Pyridine ligands are depicted in the wireframe model. The difulorobenzene molecule shows positional disorder and only one split position is shown.

From a pyridine solution of compound **2-Ph** which was layered with diethyl ether and stored at –30 °C for one week, a co-crystal of compound **2-Ph** and compound **A** (side-product) was obtained reproducibly. The crystal forms in the monoclinic space group *P* $2_1/c$ with Z = 4. The unit cell parameters are a = 21.8748(9) Å, b = 12.7068(5) Å, c = 18.7200(7) Å, α = 90°, β = 113.0080(10)°, γ = 90°. The bond lengths of compound **2-Ph** in the co-crystal slightly differ from those of crystal obtained from difluorobenzene. Bi1–C1 bond length is 2.268(3) Å, C1–C2 bond length is 1.521(5) Å and C2–N1 bond length is 1.472(5) Å.

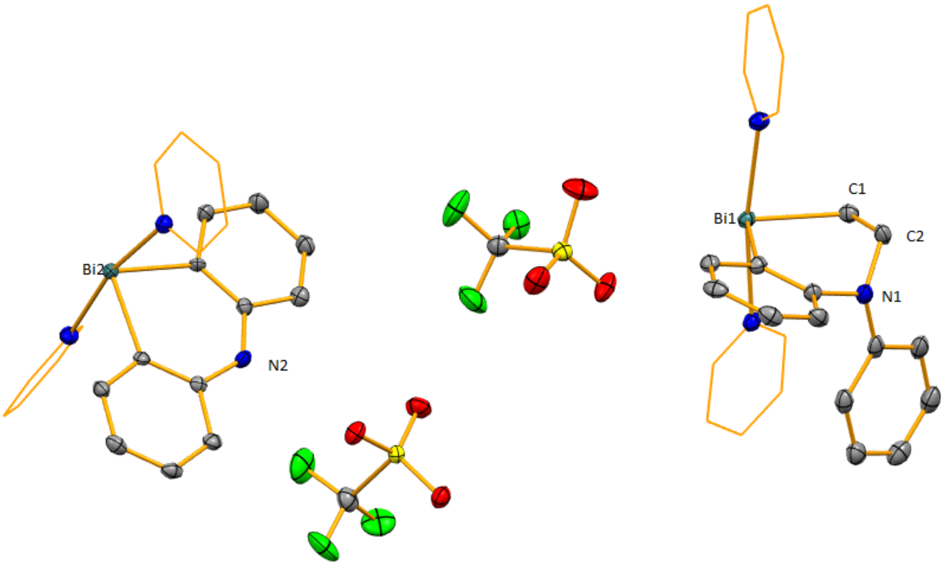


**Figure S54**: Crystal structure of co-crystallised compound **2-Ph** and compound **A**. Displacement ellipsoids are drawn at the 50% probability level. Hydrogen atoms are omitted for clarity. Pyridine ligands are depicted in the wireframe model.

Attempt to crystallise compound **2-Ph** from a THF solution layered with *n*-hexane, gave crystals of compound **2-Ph** that crystallised in a triclinic $P\bar{1}$ space group with Z= 1. The unit cell parameters are a = 10.8208(19) Å, b = 10.9387(15) Å, c = 13.304(4) Å, α = 98.939(12)°, β = 110.027(14)°, γ = 101.946(9)°. The structure is heavily disordered at multiple atoms hence not suitable for a detailed discussion of the bonding parameters.

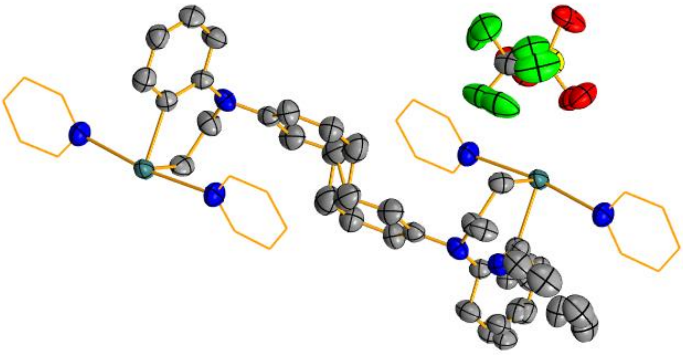

**Figure S55**: Crystal structure of compound **2-Ph**, with disorder. Displacement ellipsoids are drawn at the 50% probability level. Hydrogen atoms are omitted for clarity. Pyridine ligands are depicted in the wireframe model.

**Compound 3-Ph**

Compound **3-Ph** crystallised from a THF solution which was layered with n-hexane at –30 °C, over the period of 3 days in the triclinic space group $P\overline{1}$ with Z = 2. The unit cell parameters are a = 10.625(3) Å, b = 11.118(3) Å, c = 15.307(8) Å, α = 105.579(14)°, β = 94.128(19)°, γ = 102.187(8)°. Bi1–C1 bond length is 2.238(2) Å, the C1–C2 bond length is 1.521(3) Å, and the C2–N1 bond length is 1.485(3) Å.

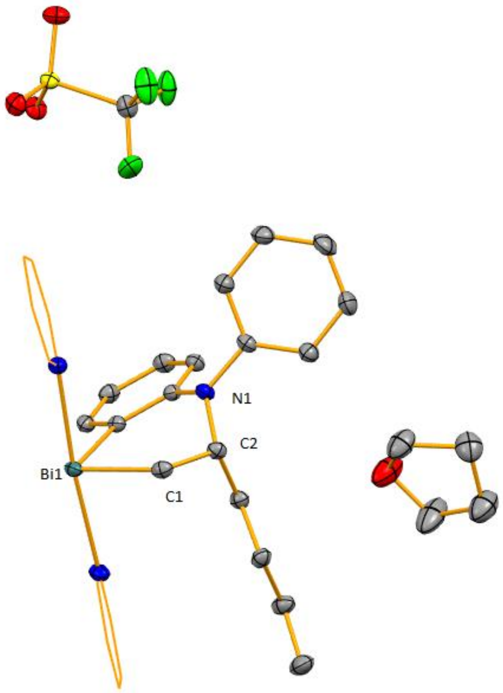


**Figure S56**: Molecular structure of compound **3-Ph** in the solid state. Displacement ellipsoids are drawn at the 50% probability level. Hydrogen atoms are omitted for clarity. Pyridine ligands are depicted in the wireframe model.

**Compound 3-*i*Pr**

Compound **3-*i*Pr** crystallised from a pyridine solution which was layered with diethyl ether at –30 °C, over the period of 2 weeks in the monoclinic space group $P\ 2_1/n$ with Z = 4. The unit cell parameters are a = 12.191(2) Å, b = 16.831(5) Å, c = 13.922(3) Å, α = 90° β = 93.548(7)° γ = 90°. Bi1–C1 bond length is 2.247(2) Å, the C1–C2 bond length is 1.526(3) Å, and the C2–N1 bond length is 1.483(3) Å.

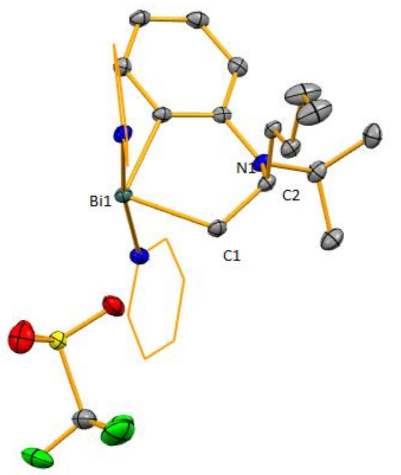


**Figure S57**: Molecular structure of compound **3-*i*Pr** in the solid state. Displacement ellipsoids are drawn at the 50% probability level. Hydrogen atoms are omitted for clarity. Pyridine ligands are depicted in the wireframe model.

**Compound 5-*i*Pr**

Compound **5-*i*Pr** crystallised from a pyridine solution which was layered with *n*-pentane at –30 °C, over the period of 2 weeks in the monoclinic space group $P\ 2_1/n$ with Z = 4. The unit cell parameters are a = 12.7890(4) Å, b = 16.3837(5) Å, c = 13.3016(4) Å, α = 90° β = 90.5710(10)° γ = 90°. Bi1–C1 bond length is 2.235(4) Å, the C1–C2 bond length is 1.520(6) Å, and the C2–N1 bond length is 1.479(4) Å.

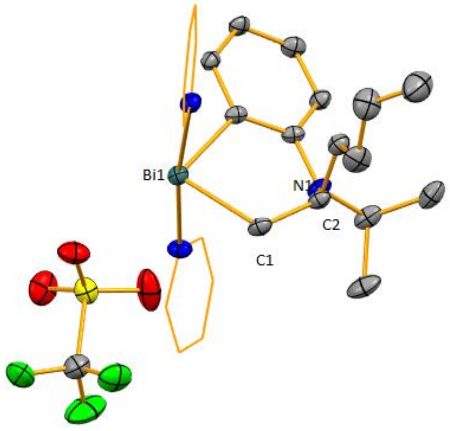


**Figure S58**: Molecular structure of compound **5-*i*Pr** in the solid state. Displacement ellipsoids are drawn at the 50% probability level. Hydrogen atoms are omitted for clarity. Pyridine ligands are depicted in the wireframe model.

**Table S8.** Crystallographic data for **2-Ph**, **3-Ph**, **3-*i*Pr**, and **5-*i*Pr**.

| Compound | **2-Ph** | **3-Ph** | **3-*i*Pr** | **5-*i*Pr** |
|---|---|---|---|---|
| Empirical formula | $C_{28}H_{25}BiF_4N_3O_3S$ | $C_{33}H_{39}BiF_3N_3O_4S$ | $C_{26}H_{33}BiF_3N_3O_3S$ | $C_{26}H_{31}BiF_3N_3O_3S$ |
| Formula weight | 768.55 | 839.737 | 730.588 | 731.58 |
| Temperature [K] | 100.00 | 100.00 | 100(2) | 100(2) |
| Crystal system | monoclinic | triclinic | monoclinic | monoclinic |
| Space group (number) | $C2/c$ (15) | $P\overline{1}$ (2) | $P2_1/n$ (14) | $P2_1/n$ (14) |
| *a* [Å] | 13.628(4) | 10.625(3) | 12.191(2) | 12.7890(4) |
| *b* [Å] | 16.811(5) | 11.118(3) | 16.830(5) | 16.3837(5) |
| *c* [Å] | 23.972(9) | 15.307(8) | 13.922(3) | 13.3016(4) |
| α [°] | 90 | 105.579(14) | 90 | 90 |
| β [°] | 94.89(2) | 94.128(19) | 93.548(7) | 90.5710(10) |
| γ [°] | 90 | 102.187(8) | 90 | 90 |
| Volume [$Å^3$] | 5472(3) | 1686.6(11) | 2851.1(12) | 2786.96(15) |
| *Z* | 8 | 2 | 4 | 4 |
| $\rho_{calc}$ [$gcm^{-3}$] | 1.866 | 1.654 | 1.709 | 1.744 |
| $\mu$ [$mm^{-1}$] | 6.583 | 5.345 | 6.308 | 6.453 |
| *F*(000) | 2984 | 832 | 1440 | 1432 |
| Crystal size [$mm^3$] | 0.145×0.074×0.059 | 0.188×0.166×0.098 | 0.173×0.128×0.080 | 0.289×0.144×0.061 |
| Crystal color | colorless | yellow | colorless | colorless |
| Crystal shape | block | block | block | block |
| Radiation | Mo$K_\alpha$ (λ=0.71073 Å) | Mo $K_\alpha$ (λ=0.71073 Å) | Mo $K_\alpha$ (λ=0.71073 Å) | Mo$K_\alpha$ (λ=0.71073 Å) |
| 2θ range [°] | 3.86 to 52.92 (0.80 Å) | 3.92 to 52.74 (0.80 Å) | 3.80 to 67.24 (0.64 Å) | 3.94 to 56.58 (0.75 Å) |
| Index ranges | −17 ≤ h ≤ 17<br>−20 ≤ k ≤ 20<br>−30 ≤ l ≤ 30 | −15 ≤ h ≤ 14<br>−13≤ k ≤ 13<br>−19 ≤ l ≤ 19 | −18 ≤ h ≤ 17<br>−26 ≤ k ≤ 25<br>−21 ≤ l ≤ 17 | −17 ≤ h ≤ 16<br>−21 ≤ k ≤ 21<br>−14 ≤ l ≤ 17 |
| Reflections collected | 68467 | 52429 | 80185 | 37675 |
| Independent reflections | 5623<br>$R_{int}$ = 0.0440<br>$R_{sigma}$ = 0.0194 | 6894<br>$R_{int}$ = 0.0300<br>$R_{sigma}$ = 0.0177 | 10329<br>$R_{int}$ = 0.0437<br>$R_{sigma}$ = 0.0332 | 6893<br>$R_{int}$ = 0.0488<br>$R_{sigma}$ = 0.0380 |
| Completeness to θ = 25.2417° | 100.0 % | 100.0 % | 100.0 % | 100.0 % |
| Data / Restraints / Parameters | 5623/375/369 | 6894/0/407 | 10329/0/380 | 6893/0/344 |
| Absorption correction $T_{min}/T_{max}$ (method) | 0.6648/0.7454 (multi-scan) | 0.6187/0.7466 (multi-scan) | 0.5825/0.7466 (multi-scan) | 0.6689/0.7457 (multi-scan) |
| Goodness-of-fit on $F^2$ | 1.336 | 1.060 | 0.947 | 1.092 |
| Final *R* indexes [$I$≥2σ($I$)] | $R_1$ = 0.0339<br>w$R_2$ = 0.0620 | $R_1$ = 0.0254<br>w$R_2$ = 0.0340 | $R_1$ = 0.0281<br>w$R_2$ = 0.0482 | $R_1$ = 0.0329<br>w$R_2$ = 0.0527 |
| Final *R* indexes [all data] | $R_1$ = 0.0384<br>w$R_2$ = 0.0630 | $R_1$ = 0.0172<br>w$R_2$ = 0.0346 | $R_1$ = 0.0400<br>w$R_2$ = 0.0516 | $R_1$ = 0.0446<br>w$R_2$ = 0.0552 |
| Largest peak/hole [$eÅ^{-3}$] | 1.13/−1.90 | 0.77/−0.55 | 0.91/−1.30 | 1.01/−1.70 |
| CCDC number | 2428765 | 2428764 | 2428763 | 2428762 |

**Acknowledgements**

Funding by the Deutsche Forschungsgemeinschaft (DFG, grant numbers LI2860/3-1, LI2860/5-1), the LOEWE program (LOEWE/4b//519/05/01.002(0002)/85), the Spanish Ministerio de Ciencia, Innovación y Universidades (projects PID2022-138861NB-I00 and CEX2021-001202-M), the Generalitat de Catalunya (project 2021SGR442), and the Dutch Research Council (NWO) is gratefully acknowledged. This project has received funding from the European Research Council (ERC) under the European Union's Horizon 2020 research and innovation program (grant agreement No 946184).
